# A Genome-Wide Association Study for Culm Cellulose Content in Barley Reveals Candidate Genes Co-Expressed with Members of the *CELLULOSE SYNTHASE A* Gene Family

**DOI:** 10.1371/journal.pone.0130890

**Published:** 2015-07-08

**Authors:** Kelly Houston, Rachel A. Burton, Beata Sznajder, Antoni J. Rafalski, Kanwarpal S. Dhugga, Diane E. Mather, Jillian Taylor, Brian J. Steffenson, Robbie Waugh, Geoffrey B. Fincher

**Affiliations:** 1 The James Hutton Institute, Invergowrie, Dundee, DD2 5DA, Scotland, United Kingdom; 2 ARC Centre of Excellence in Plant Cell Walls, School of Agriculture, Food & Wine, The University of Adelaide, Waite Campus, Glen Osmond, SA 5064, Australia; 3 Australian Centre for Plant Functional Genomics, The University of Adelaide, Waite Campus, Glen Osmond, SA 5064, Australia; 4 Genetic Discovery Group, DuPont Agricultural Biotechnology, DuPont Pioneer, DuPont Experimental Station, Building E353, Wilmington, DE, 19803, United States of America; 5 Genetic Discovery Group, DuPont Agricultural Biotechnology, DuPont Pioneer, 7300 NW 62nd Avenue, Johnston, IA, 50131, United States of America; 6 Department of Plant Pathology, University of Minnesota, St. Paul, MN, 55108, United States of America; 7 Division of Plant Sciences, University of Dundee at The James Hutton Institute, Invergowrie, Scotland, Dundee, DD2 5DA, United Kingdom; Iowa State University, UNITED STATES

## Abstract

Cellulose is a fundamentally important component of cell walls of higher plants. It provides a scaffold that allows the development and growth of the plant to occur in an ordered fashion. Cellulose also provides mechanical strength, which is crucial for both normal development and to enable the plant to withstand both abiotic and biotic stresses. We quantified the cellulose concentration in the culm of 288 two – rowed and 288 six – rowed spring type barley accessions that were part of the USDA funded barley Coordinated Agricultural Project (CAP) program in the USA. When the population structure of these accessions was analysed we identified six distinct populations, four of which we considered to be comprised of a sufficient number of accessions to be suitable for genome-wide association studies (GWAS). These lines had been genotyped with 3072 SNPs so we combined the trait and genetic data to carry out GWAS. The analysis allowed us to identify regions of the genome containing significant associations between molecular markers and cellulose concentration data, including one region cross-validated in multiple populations. To identify candidate genes we assembled the gene content of these regions and used these to query a comprehensive RNA-seq based gene expression atlas. This provided us with gene annotations and associated expression data across multiple tissues, which allowed us to formulate a supported list of candidate genes that regulate cellulose biosynthesis. Several regions identified by our analysis contain genes that are co-expressed with *CELLULOSE SYNTHASE A (HvCesA)* across a range of tissues and developmental stages. These genes are involved in both primary and secondary cell wall development. In addition, genes that have been previously linked with cellulose synthesis by biochemical methods, such as *HvCOBRA*, a gene of unknown function, were also associated with cellulose levels in the association panel. Our analyses provide new insights into the genes that contribute to cellulose content in cereal culms and to a greater understanding of the interactions between them.

## Background

Cellulose is crucially important to plant cell walls for several reasons. Through its presence in the primary cell wall, cellulose provides a strong yet flexible scaffold allowing growth of cells, while maintaining a defined structure and three-dimensional shape for the cell. In the secondary cell wall, which is produced once cells have stopped enlarging, cellulose provides the mechanical strength necessary to support the plant against the forces of gravity and to help it withstand biotic and abiotic stresses. For example, cellulose content per unit length of the culm has been shown to be a major determinant of straw strength [1]. A weaker culm makes a plant more prone to lodging, which leads to reduced grain yields. In secondary cell walls, cellulose and non-cellulosic wall polysaccharides are often embedded in an amorphous lignin matrix, together forming an abundant but relatively intractable bio-composite (lignocellulose) that has great potential as a raw material in second generation biofuel production. Understanding more about the genes regulating cellulose content will therefore contribute to several key global challenges related to crop yields and renewable bioenergy.

Cellulose is a long unbranched polysaccharide comprised of a chain of glucosyl residues joined by glycosidic linkages between the first and fourth carbon atoms of a series of glucosyl monomers, (1→4)- β-glucan. A sturdy and strong scaffold is constructed by multiple cellulosic chains aligning to form a crystalline microfibril comprised of between 18 and 36 glucan chains [2,3,4]. A single microfibril is believed to be synthesised by a complex of particle rosettes located at the plasma membrane [5,6,7].

Previous studies have identified the *CELLULOSE SYNTHASE A* (*CesA*) genes as being crucial in the synthesis of cellulose. The first *CesA* genes to be identified were *GsCesA1* and *GsCesA2* in cotton [8]. In barley eight *CesA* genes have been described, six of which form two groups of closely co-expressed genes [9]. Thus, *HvCesA1*, *HvCesA2*, and *HvCesA6* comprise group one, and group two is composed of *HvCesA4*, *HvCesA7*, and *HvCesA8*, with *HvCesA3* and *HvCesA5* falling into neither cluster. Group one has been implicated in primary cell wall development, whereas group two has a clear role in secondary cell wall construction [9], based on the types and developmental stages of the tissues in which they are transcribed. This means that to some degree the expression patterns of these two groups of genes differ both spatially and temporally. A recent study by Zhang et al [10] illustrated this at a very localised and detailed scale by comparing expression patterns of the maize orthologs of these genes along an elongating internode at stage V16 (when the collar of the 16^th^ leaf is visible) [11]. When expression levels of genes from group one and two are compared across sections of the internode it is only in section 1, the meristematic zone, that group one genes (*ZmCesA2*, *ZmCesA5*, *ZmCesA7*, and *ZmCesA8*) have a higher level of expression than group two genes (*ZmCesA10*, *ZmCesA11*, and *ZmCesA13*). This corresponds with increasing cellulose content from low levels in section 1 (20% by weight), to section 10, the upper maturation zone where cellulose content reaches its maximum of 42% by weight.

Mutation studies of genes involved in both primary and secondary cell wall development have also helped to characterise and typify these processes. Mutations in genes such as *PROCUSTE1* (*PRC1)*, which encodes *AtCesA6* and *RSW1* (*AtCesA1*), result in lower levels of cellulose, coupled with gaps between cells and abnormally shaped cells due to restricted cell elongation [12]. Mechanical strength for the plant overall is provided primarily by secondary cell walls. The fragile stem locus described by Burton et al.[13], *fs2*, is the result of mutations in *HvCesA4*. Two independent *fs2* mutants cause reductions of crystalline cellulose by 40% and 60% when compared with their respective wild type parental lines. As variation in cellulose content can be characteristic of both primary and/or secondary wall development, we may expect to identify genes involved in either or both of these processes by carrying out a GWAS for this trait.

In addition to genes responsible for the synthesis of cellulose, several genes have been shown to be involved in cellulose breakdown. These include members of several glycoside hydrolase families [14]. There is also emerging evidence that some genes that encode polysaccharide hydrolases from these families are actually involved in the synthesis of cellulose, especially with regards to plant development. The GH9 family includes endo-(1,4)-β-glucanases, also referred to as cellulases [15]. One member of this gene family, known as *KORRIGAN* (*KOR*) (*HvCel1* in barley), has been studied comprehensively to demonstrate its role in cellulose synthesis. In *Arabidopsis*, a reduction in crystalline cellulose coupled with an increase in the amount of pectin and non-crystalline cellulose has been observed in mutant and T-DNA insertion lines of *kor*. These lines also exhibit reduced cell elongation, have a dwarf phenotype and reduced wall separation between cells [16–21].

Here we describe the results of a GWAS carried out to identify regions of the barley genome associated with variation in cellulose concentration of the culm. Although such an approach has been applied to understanding regions within the genome associated with cellulose content in *Miscanthus* [22] and *Populus* [23], we are not aware of any other GWAS for this trait on other members of the Poaceae, an economically important family of plants that includes grasses and cereals. We quantified this trait in several sets of spring barley germplasm, chosen to reflect both 2-rowed and 6-rowed breeding germplasm, as well as malting and feed type barley, currently the two main uses of this crop. The germplasm samples used in our analysis are part of the USDA-funded barley CAP project and therefore represent accessions that are currently or have been included in contemporary breeding programs. We identified significant associations in regions containing glycosyl hydrolases, and others that overlap with the map positions of two other fragile stem loci, *fs1* and *fs3*. Several of the regions contained genes known to be highly co-expressed with *HvCesA* genes involved in both primary and secondary cell wall development, and we show here that they are co-expressed across an extended range of barley tissues, providing independent support for their role in determining final culm cellulose concentration.

## Material and Methods

### Germplasm

Germplasm from six US spring breeding programs, each represented by 96 accessions (S1 Table) was grown and phenotyped for cellulose content. The germplasm has been described previously by Hamblin et al. [24] and Zhou et al [25]. Here, a combination of both 2- and six- rowed genotypes from the Montana (MT) and North Dakota (N2), Minnesota (MN), North Dakota (N6) Washington (WA) and Utah (UT) programs were included. A list of the genotypes used is given in S3 Table. All lines were genotyped with barley oligonucleotide pool assays (BOPA) 1 and 2 yielding data for 3,072 SNP loci in total per individual [24, 26]. The BOPA assays were designed using Expressed Sequence Tags (ESTs), therefore all of these SNP loci reside in protein coding regions of the genome. Genotypic data can be accessed at http://www.hordeumtoolbox.org/.

### Phenotyping

The barley accessions were grown according to standard agronomic practices at the Minnesota Agricultural Experiment Station on the St. Paul campus of the University of Minnesota in 2008. Plots consisted of 30 cm long rows sown with a four-row planter with the centre two rows being the test entries and the two outside rows the border plots. Two grams of seed was used for each plot with 30 cm spacing between entries within a row and between rows. The experiment was conducted using a completely randomized design. When plants reached maturity, (hard dough stage, and leaf tissue all senescent), all lines were harvested within the same 3 day interval at the end of the season and tissue was sampled from one internode (the first below the peduncle). Internode tissue from at least three culms was bulked and ground per line. Crystalline cellulose was determined by the acetic acid/nitric acid method [27], with modifications as described in [28] and is presented in Tables 1 and 2.

**Table 1:** Cellulose quantities by breeding program and subpopulation in barley culms.

Abbreviation	Breeding program	sample size	mean cellulose concentration	range of cellulose concentration
WA	Washington	94	0.394	0.222–0.464
MT	Montana	96	0.433	0.340–0.497
N2	North Dakota	84	0.456	0.264–0.648
UT	Utah	96	0.409	0.292–0.498
N6	North Dakota	94	0.361	0.035–0.477
MN	Minnesota	96	0.402	0.179–0.459
	All	560	0.408	0.035–0.648

Sample size, mean cellulose concentration, and range of cellulose concentration in mg cellulose / mg dry weight.

Populations are based on Barley CAP breeding programs.

**Table 2:** Cellulose quantities by subpopulation defined by STRUCTURE in barley culms.

Subpopulation	sample size	mean cellulose concentration	range of cellulose concentration
1	18	0.418	0.364–0.468
2	84	0.356	0.035–0.427
3	87	0.401	0.264–0.648
4	98	0.401	0.179–0.459
5	183	0.414	0.222–0.597
6	58	0.405	0.292–0.498
All	528	0.399	0.035–0.648

Sample size, mean cellulose concentration, and range of cellulose concentration in mg cellulose / mg dry weight.

Populations are based on subpopulations lines were assigned to after STRUCTURE analysis.

### Genetic analysis

GenALEx 6.5 [29] was used to construct a genetic distance matrix and carry out a principal coordinates analysis (PCoA) to identify subgroups within the data set based on differences in allele frequency. We then visualised the first 3 principal coordinates using CurlyWhirly (http://bioinf.hutton.ac.uk/curlywhirly). The Bayesian clustering method STRUCTURE [30] provides Fst values by population, allowing quantification of differentiation between subgroups. Because our PCoA analysis grouped the data into between 5 and 9 subgroups, and Evanno et al., [31] suggest testing a range of values starting at 1 or 2, up to and including the true number of populations plus 3, we used 2 to 12 as our priors (*K* values) for STRUCTURE [30]. The admixture model was selected with a burn in length of 10 x 10^3^ and Markov Chain Monte Carlo (MCMC) of 10 x 10^3^ using 20 reps per value of *K*. The results file was uploaded into Structure Harvester v0.6 [32] to estimate the most likely number of populations within the dataset (Δ*K*) [31]. This corresponds to the K value with the highest Δ*K*. Linkage disequilibrium (LD) was calculated using Haploview [33] for both the entire dataset, and for the four subpopulations with more than 88 lines.

In total 2,842 markers that had less than 10% missing data and minimum allele frequency (MAF) >5% were included in the GWAS analysis in Genstat v.14 (VSN International). A null and kinship analysis, using a kinship matrix also generated in GenStat was performed. This analysis was carried out using the subpopulations identified as described in the previous section separately. A stringent false discovery rate (FDR) < 5% was calculated using the qvalue package [34] in R version 3.1.1 (R core team 2014) to provide adjusted p values. This set the significance threshold to (-log10 p ≥ 3.0) for the adjusted p≤0.05, and (-log10 p ≥ 5.69) for the adjusted p ≤0.001. Significant SNPs (−log10 p ≥ 3.0) positioned within 5 cM were considered to be linked to the same QTL, with the most significant SNP chosen as representing the QTL. Package stats in R Statistical Computing Environment (R Development Core Team 2014) was used to calculate the measure of association between the marker and the phenotype, the cellulose content. As the data was not expected to follow bivariate normal distribution, the squared rank-based correlation coefficient was calculated based on Kendall’s tau statistic.

Nomenclature for identified QTLs broadly follows that described in [35] and OWB-DGGT (http://wheat.pw.usda.gov/ggpages/maps/OWB/), using Cel as an abbreviation for cellulose content.

Regions that contain significant associations with cellulose concentration were anchored using the barley sequence assembly and map positions determined for all markers that had a significant association with the trait using three genetic maps, a 9K i-Select map [36], the Barley Genome Zipper [37], the POPSEQ map [38] and one physical map, i.e. the barley genome assembly [39]. However in Table 3 we just provide the name of the marker with the highest LOD score for each region that showed a significant association with cellulose content. These genetic and physical maps were used in combination with http://floresta.eead.csic.es/barleymap/ to provide annotations for genes within the intervals identified by the association analysis. When querying http://floresta.eead.csic.es/barleymap/, we extended the interval by 2.5 cM in both directions from markers that flanked our QTL (i.e. a total of 5cM) to take account of map order uncertainty and LD.

**Table 3:** Regions of the barley genome identified by GWAS as having significant associations with cellulose content.

							Pop 3			Pop 5							
							(2-row)			(2-row)							3rd
**Ch**	**Peak**	Marker	i-select cM	POPSEQ	IBSC	Zipper MxBk	-log 10 (pvalue)		R2	-log 10 (pvalue)		R2	*Candidate annotation*	Barley Gene id (MLOC)	cM (IBSC)	Morex Contig	internode FPKM
**1H**	**QCel1H1**	11_10516	65.5	59.1	61	60.8	0.8		0	3.6	*	0.04	?	n/a	n/a	n/a	n/a
	**QCel1H2**	11_11037	84.7	n/a	n/a	n/a	0		0.01	6.1	***	0.1	*HvGT1*	MLOC_66574	72	contig_51769	611.2
**5H**	**QCel5H1**	11_20097	100.3	80.3	80.3	n/a	1		0.04	5.7	***	0.08	*HvGH1*	MLOC_66740	83.5	contig_52038	20.7
													*HvChitinase*	AK249826	80.4	n/a	n/a
	**QCel5H2**	11_20134	106.2	n/a	92.4	n/a	0.1		0	3.9	*	0.05	*HvGH1*	AK252806	88.2	contig_230478	5.6
													*HvCel1*	AK373680	97.3	contig_137207	0.1
		11_10094	122.4	95.5	n/a	n/a	0		0.01	3.8	*	0.07	*HvGT2*	MLOC_1996	97.7	contig_117772	20.9
	**QCel5H3**	11_20487	134.6	n/a	n/a	n/a	2.3		0.03	4.1	*	0.06	*EXO67725 (GH3)*	MLOC_67725	122.4	contig_53512	3
													*EXO75585 (GH3)*	MLOC_75585	122.4	contig_68278	0.2
													*GH3*	MLOC_56792	126.1	contig_41156	0
													*HvCobra1 (GH1)*	MLOC_55526	125.8	contig_40045	482.2
	**QCel5H4**	12_30502	0	169.4	n/a	n/a	0		0	5.9	***	0.08	*Sugar transferase*	MLOC_11345	166.67	contig_1560890	71.5
**6H**	**QCel6H1**	11_10427	34.4	38	n/a	37.4	0.1		0	4.2	*	0.06	*HvCesA9*	AK367031	37.4	contig_39637	777.1
	**QCel6H2**	11_10239	112.3	105.1	104.8	n/a	0		0	3.6	*	0.05	?	n/a	n/a	n/a	n/a
**7H**	**QCel7H1**	11_10069	83.4	75.2	n/a	74.4	4.7	*	0.04	0		0	*HvCslF6*	MLOC_57200	72.5	contig_41513	244.1
													*HvNAM*	MLOC_19933	73.2	contig_1587220	7.3

This table includes just pop 3 and 5 as these were the two subpopulatons in which significant association with culm cellulose content were identified. Significant (−log10 p ≥ 3) marker-trait associations which pass the False Discovery Rate (FDR) adjusted p value threshold of ≤ 0.05 are indicated by *,

≤ 0.01 are indicated by **,

and ≤ 0.001 are indicated by ***

R-squared values for each marker are also included. The marker name and location for the SNP with the highest lod score is provided for each QTL. Germplasm included in the analysis was a subset of 2-rowed and 6- rowed spring accessions from the Barley CAP project. Subpopulations, referred to here as “Pop”, are those determined by STRUCTURE analysis based on degree of shared genetic ancestry. Barley Gene ID (MLOC), Morex Contig, 3^rd^ internode FPKM—fragments per kilobase of exon per million fragments mapped from [39] are provided for each candidate gene identified under each peak where information is available. Four different versions of the barley map were used to provide information for each locus: 9K i-Select [35], Barley Genome Zipper [37], POPSEQ [38] and the IBSC [39]. Question marks indicate where no obvious candidate was identified for particular regions based on the available annotations.

For those genes that we considered to be candidates for influencing cellulose content in the culm we referred to the barley genome assembly [39] to extract information on gene expression in the third internode of the culm, a similar tissue to that assayed in the current experiment.

For those genes that we suspected to be the most likely candidates for influencing cellulose content we compared expression patterns across a range of tissues using data from the IBSC [39], and our unpublished data. The developmental stages and tissues are embryo (germinating), root (10cm seedlings), shoot (10cm seedlings), inflorescence (0.5cm), inflorescence (1–1.5cm), tillers (3rd internode), grain (5 days post anthesis (DPA)), grain (15 DPA), etiolated (10 day seedlings), lemma (42 days after planting (DAP)), lodicule (42 DAP), palea (42 DAP), epidermis (28 DAP), rachis (35 DAP), root (4 week seedling), and senescing leaf (2 months). The gene expression data are available as normalised transcript abundance in fragments per kilobase of exon per million fragments mapped (FPKM). Details regarding how the reads were mapped, total number of reads per stage and how FPKM was calculated can be found in IBSC 2012 [39]. For groups of genes we identified as being coexpressed we retrieved the rice orthologs and expression levels in 16 tissues from http://rice.plantbiology.msu.edu/. The genetic position of these associations was compared to that of introgressions containing mutant alleles of fragile stem (*fs*) loci, namely *fs1* on chromosome 5H, *fs2* on 1H, and *fS3* on 7H, [40] as these have been shown to have decreased cellulose content in the culm.

## Results

### Population structure and linkage disequilibrium

**Fig 1:**
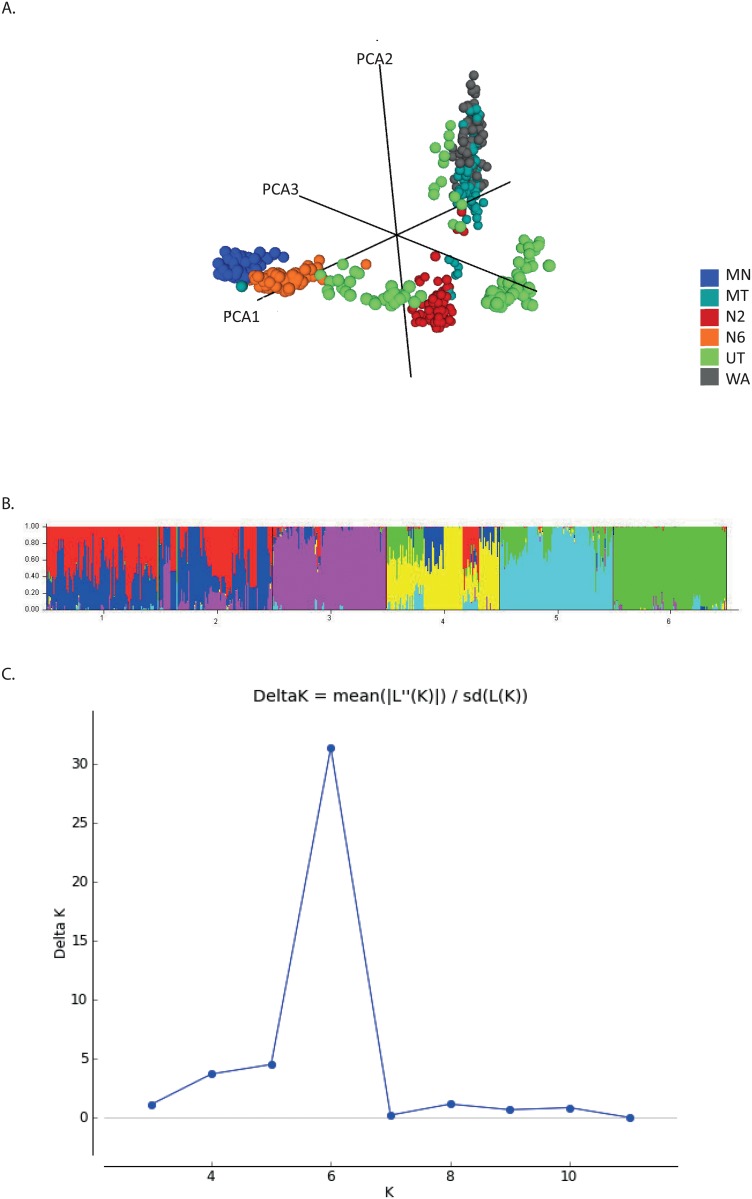
Genotypic data and population structure analysis. (A) Principal coordinates analysis of all lines phenotyped, colour coded by breeding program. (B) STRUCTURE bar plot for K = 6 based on bOPA 1&2 genotyping data for spring barley lines ordered by breeding programs, but colour coded by K value. Please note colours in A. are independent to those in B. Breeding program 1 = Washington (WA), 2 = Montana (MT), 3 = 2- row North Dakota (N2), 4 = Utah (UT), 5 = 6-row North Dakota (N6), and 6 = Minnesota (MN). Colours represent subpopulation defined by shared genetic ancestry. Q value represents proportion of ancestry to a given subpopulation. (C) Output from Structure Harvester showing *K* as calculated based on Δ*K* method, in this case *K* = 6. *L(K)* represents the likelihood distribution of *K*, and *L”(K)* represents the second order rate of change from *L(K)*.

The first axis of the PCoA explained 58% of variation within this dataset, clearly separating lines based on row type (Fig 1). Axis 2 and 3 explained a further 15.6% and 11.5% respectively. The PCoA identified between 5 and 9 putative subpopulations. Structure analysis showed that there was clearly admixture in some subpopulations, and Δ*K* indicated that the most likely number of subpopulations within the dataset was 6 (Fig 1, S1 Fig). Setting the shared ancestry threshold, Q, to 0.6 allocated individual lines into the populations summarised in S2 Table, with more detail provided in S3 Table. Lines that did not meet these criteria were excluded from further analysis. Intra chromosomal LD analysis revealed that the average extent of LD across all chromosomes varied between subpopulations when they were analysed separately (pop2 = 21.2cM, pop 3 = 10.8cM, pop 4 = 7.5cM, and pop5 = 13.2 cM). The average extent of LD was much smaller when all lines were analysed together (5.5cM).

### Cellulose concentration

**Fig 2:**
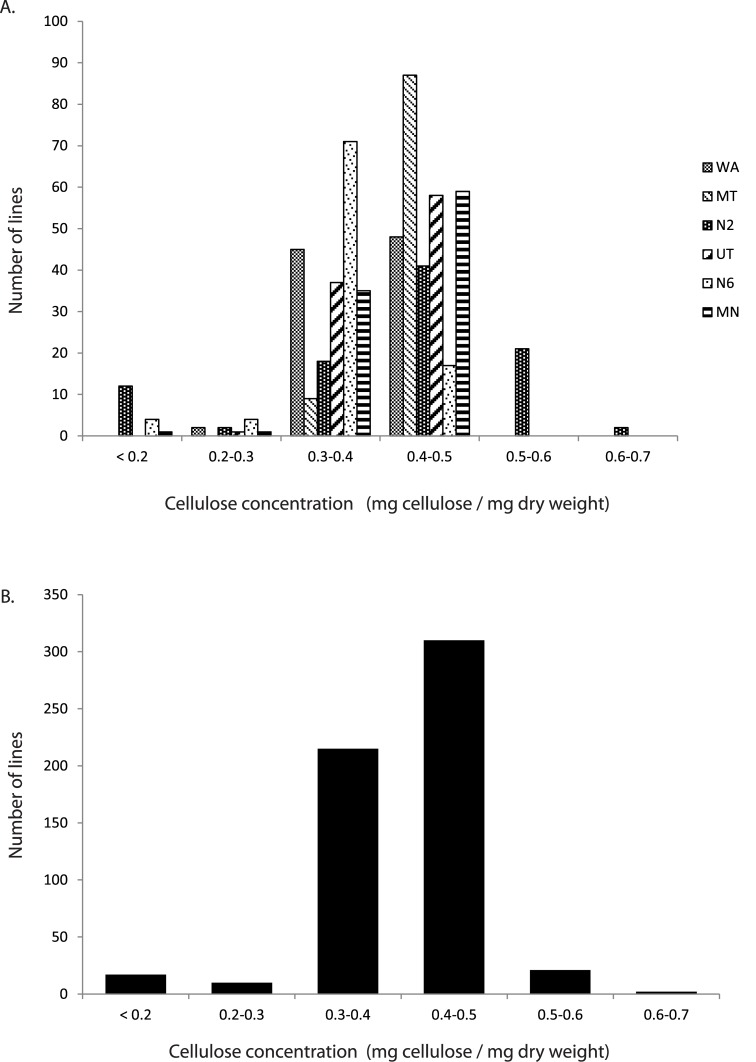
Phenotypic data used to carry out a genome wide association study (GWAS). (A) Mean culm cellulose content for 2-row and 6 row spring barley accessions by breeding program. (B) Mean culm cellulose content for all lines to illustrate the distribution of this trait in the barley CAP programs included in this analysis.

For all subpopulations most lines (between 61.5–100%) had between 0.3 and 0.5 mg cellulose /mg of dry weight. The average concentration of cellulose differed significantly among breeding programs [F (4) = 3.58, p = 0.007], ranging from 0.39 mg cellulose /mg of dry weight (MN) to 0.43 mg cellulose /mg of dry weight (MT) (Fig 2). Sample size, mean cellulose concentration, range of (cellulose concentration) values, and heritability (of cellulose concentration), within each breeding program and subpopulation as well as across all lines can be found in Table 1.

### GWAS

**Fig 3:**
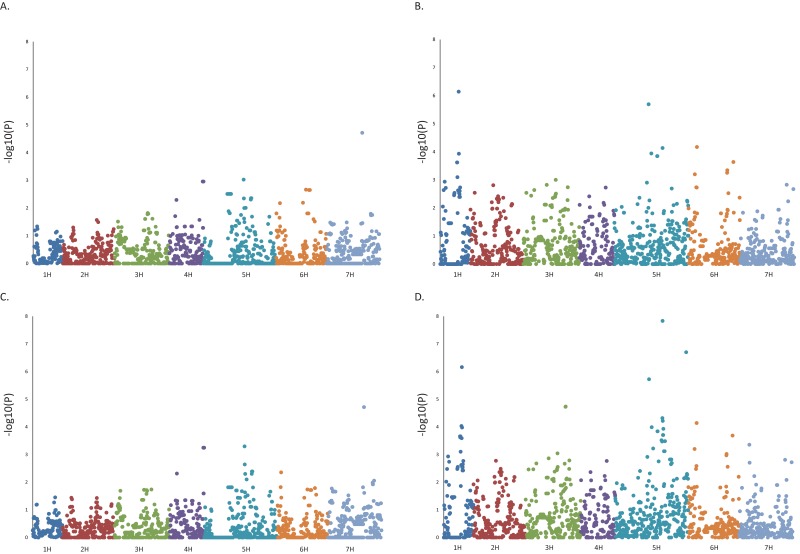
Manhattan plots of culm cellulose content (mg cellulose / mg dry weight) genome wide association scans (GWAS) using the Kinship relationship model. The-log10 (p-values) from a genome-wide scan are plotted against the position on each of the 7 barley chromosomes. Manhattan plots are displayed for those populations which had associations that passed the significance threshold set by FDR p > 0.05 (-log10P >3.0), i.e. populations 3 and 5. Manhattan plots of the null models are provided for comparison. (A) Population 3 Kinship model. (B) Population 5 Kinship model. (C) Population 3 Null model. (D) Population 5 Null model.

GWAS on each of the four largest subpopulations individually (subpopulations 2, 3, 4 and 5), detected nine regions containing significant associations (using a FDR corrected adjusted p value of −log10 p ≥3.0 with adjusted p≤0.05) with cellulose concentration (Table 3, S4 Table, Fig 3). Significant associations were detected in the two two-rowed subpopulations (subpopulations 3 and 5) but not in the two six-rowed subpopulations (subpopulations 2 and 4). The largest subpopulation, subpopulation 5, was the germplasm in which we detected most associations, and several had adjusted p values of p≤0.001 (−log10 p ≥5.69) (Table 3,S4 Table). A further significant association was identified in subpopulation 3. GWAS was not carried out on populations one and six due to the small number of individuals in each. When considering the PCoA (Fig 1) it is quite clear that the two rowed subpopulations, subpopulations 3 (dominated by N2), and 5 (mostly consisting of WA and MT) are almost as distinct from each other as they are from the six rowed subpopulations 2 and 4.

We focused our attention on these 9 loci, in particular those regions for which annotations of closely associated genes implied a possible functional link to cellulose content. Regional gene content and gene annotations were derived from the barley, *Brachypodium*, and rice genome assemblies (Table 3) using the resources described in the material and methods. This allowed us to predict putative candidate genes for 7 of the 9 associations. The candidates fell within three groups dictated by function; primary cell wall cellulose synthesis, secondary cell wall cellulose synthesis, and glycoside hydrolases. We then referred to deep RNA-seq expression data across multiple tissues and developmental stages to explore patterns of expression for each of the candidate genes [39]. Although cellulose content in the current study was assayed using the first internode, a dataset from the 3^rd^ internode of the culm was included in the IBSC study. We used these data to help prioritise an individual candidate gene’s likely contribution to cellulose content. We did not strictly interpret lack of expression as ruling a gene out of having a role in cellulose content; however, we did assume that those expressed in the target tissue are more likely to be important for the trait.

### Glycosyl transferases (GT) and associated genes

Several regions identified in our analysis included members of GT gene families [14] (QCel1H2, QCel5H2, QCel6H1, and QCel7H1), such as the cellulose synthase (*HvCesA*) genes of the GT2 family of glycosyl transferases [41, 42] that have been shown to influence cellulose content. Supportive of the GWAS approach was the identification of a region containing *HvCesA9* (QCel6H1), a member of the GT2 family that shows very high levels of transcript abundance in the 3^rd^ internode (777.1 FPKM) and has high sequence similarity to *HvCesA6*, which itself is associated with primary cell wall cellulose synthesis [9]. Another member of the GT2 family, a gene with a known function in (1,3;1,4)-β-glucan synthesis during primary cell wall development, *CELLULOSE SYTHASE LIKE F6* (*HvCslF6*) [43], is located within the region under QCel7H1 on chromosome 7H. This gene is represented by MLOC_ 572000 and shows high levels of transcript abundance in the culm tissue (244.1 FPKM). Further support for the validity of our approach was provided by associations that collocated with two of the three fragile stem loci known in barley: *fs1* on chromosome 5H (QCel5H1) and *fs3* on chromosome 7H (QCel7H1) [40]. Burton et al., [13] demonstrated that the *fs2* brittle stem mutant on chromosome 1H was attributed to the insertion of a retroelement in the first intron of the *CesA4* gene. Another gene annotation within the introgression on chromosome 7H containing *fs1* is MLOC_19933, which is known to be a transcription factor master switch of secondary wall development. This gene is an ortholog of *AtVND7* which activates transcription of several MYB transcript factors, including MYB46, that regulate secondary cell wall development in *Arabidopsis* [44].

### Glycoside hydrolases

Genes encoding glycosyl hydrolase enzymes (GH) were found in three of the nine regions associated with cellulose levels (QCel5H1, QCel5H2, and QCel5H3), including members of families GH1, GH3, GH9 and GH19 [14]. These glycosyl hydrolase families have been previously shown to hydrolyse the glycosidic linkages between the β-linked glucosyl residues in cellulose or cellodextrins, with the exception of the GH19 family, which includes chitinases and lysozymes [14]. Some members of the GH1 family, which include β-glucosidases and the Cobra-like enzymes, have been discussed above. One of the candidate gene annotations for QCel5H2 was *HvCel2*, which is a member of hydrolase family GH9 and is closely related to *HvCel1*, also known as *KOR* [15]. This GH family includes endo-β-glucanases, which have been implicated in cellulose synthesis [2], and cellobiohydrolases, which are important in the complete depolymerisation of cellulose to glucose [14]. However, it must be noted that the GH9 family also includes endo-xylanases and xyloglucanases [14].

### Co-expression across various tissues and developmental stages

**Fig 4:**
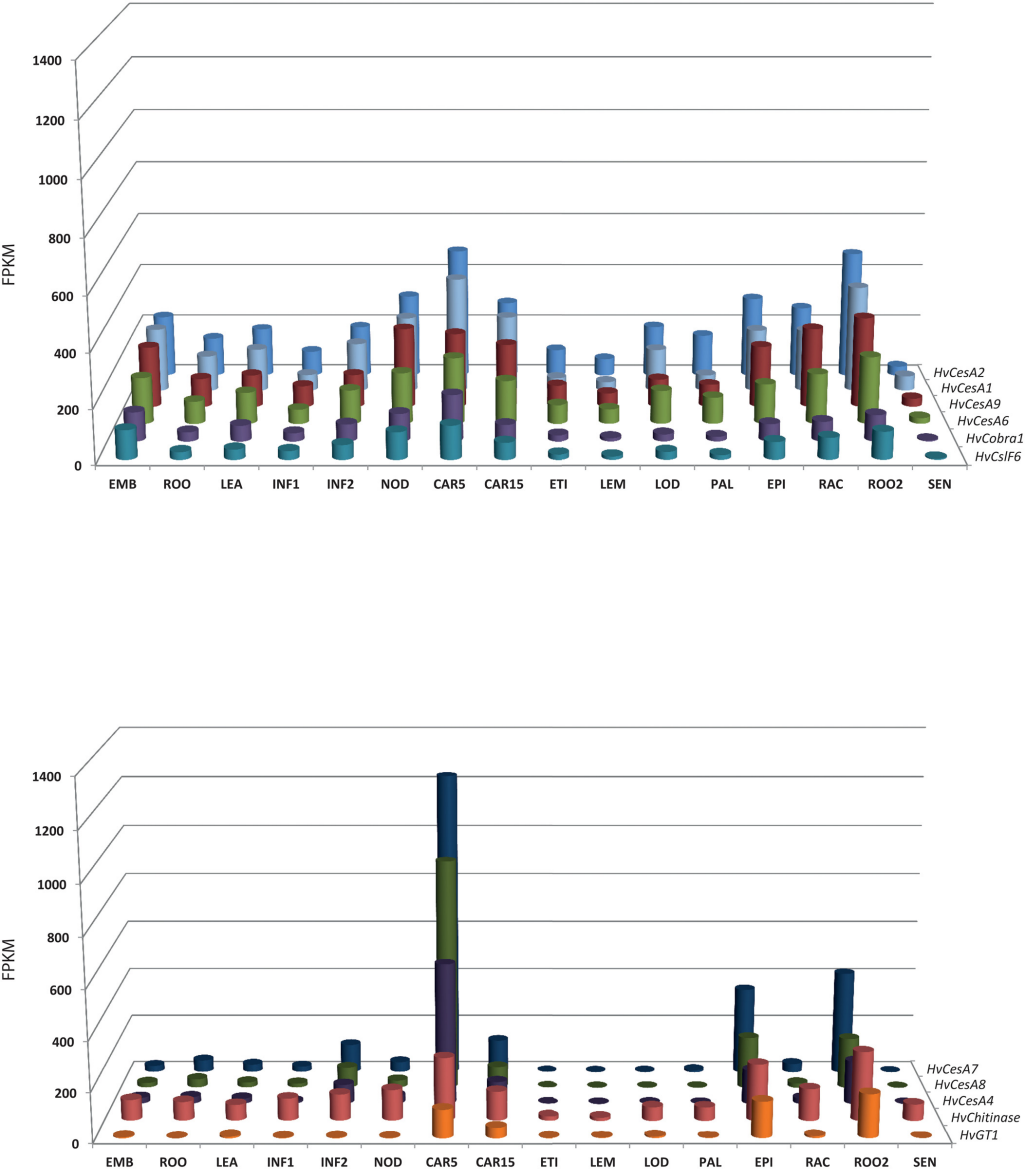
Co-expression of two groups of genes (*HvCesA9*, *HvCobra1*, *HvCslF6*, and *HvChitinase*, *HvGT1*) identified by GWAS as putatively linked to culm cellulose content with *HvCesA* genes known to be involved in primary and secondary cell wall development. Transcript abundance across a range of tissues shown in fragment per kilobase of exon per million fragments mapped (FPKM) for group 1, primary cell wall including *HvCesA1*, *HvCesA2*, and *HvCesA6* (A) for reference, and group 2, secondary cell wall including *HvCesA4*, *HvCesA7* and *HvCesA8* for reference (B). Abbreviations for tissues/ developmental stages as follows; EMB = Embryo tissues (germinating), ROO = Root (10cm seedlings), LEA = Shoot (10cm seedlings), INF1 = Inflorescence (0.5cm), INF2 = Inflorescence (1–1.5cm), NOD = Tillers (3rd internode), CAR5 = Grain (5 Days Post Anthesis—DPA), CAR15 = Grain (15 DPA), ETI = Etiolated (10 day seedlings), LEM = Lemma (6 weeks Days After Planting—DAP), LOD = Lodicule (42 DAP), PAL = Palea (42 DAP), EPI = Epidermis (28 DAP), RAC = Rachis (35 DAP), ROO2 = Root (28 DAP seedling), SEN = Senescing leaf (63 DAP).

We were interested to see if any of the candidate genes from the GWAS were co-expressed, as this could provide further evidence for their being responsible for the association peaks identified. This is particularly relevant for understanding variation in cellulose content, because the synthesis of cellulose is likely to involve a number of co-factors and interacting proteins. The co-expression analysis allowed us to prioritise genes on our list of potential candidates in Table 3. We extended the number of tissues and developmental stages for which we compared candidate gene expression to sixteen (our unpublished data). When we considered the data for the 14 candidate genes from the nine regions highlighted in Table 3, we observed that five could be classified into two groups of co-expressed genes, groups 1 and 2, which are strongly co-expressed based on pairwise regression analysis between each gene across these 16 tissues with members of the *CesA* gene family (Fig 4). The output from the regression analysis can be found in S5 Table. Group 1 genes comprised *HvCobra1*, *HvCslF6*, and *HvCesA9* (a close ortholog /paralog of *HvCesA6*), and formed a co-expression complex across the 16 different tissues with *HvCesA1*, *HvCesA2*, and *HvCesA6*, genes previously implicated in primary cell wall cellulose biosynthesis [9]. Group 2 genes, *HvChitinase* and *HvUDP-glucosyltransferase* (*HvGT)*, were co-expressed with genes important for secondary cell wall development, namely *HvCesA4*, *HvCesA7*, and *HvCesA8*. Where possible we compared the coexpression of the rice orthologs of these genes, however there was no ortholog of *HvCslF9* or *HvChitinase1*. Across both species we observed that genes in group 1 showed a higher and more significant degree of coexpression than those in group 2, with the expression of the candidate genes in barley showing a higher correlation to the known primary and secondary cell wall genes than their rice orthologs (S5 Table).

## Discussion

The barley CAP program, from which the plant material used in the current study was sourced, is based upon a well-studied set of accessions. Several papers describe the population structure within this germplasm [24,25,45], and more recently a GWAS for one quality trait, grain protein content, and five agronomic traits, plant height, heading date, percent kernel plump, grain test weight, and yield, has been published [45]. While we used only a subset of the germplasm from the CAP project in our analysis, our population structure analysis broadly agrees with the groups identified by others [24,25,45]. From the germplasm we sampled here, the program STRUCTURE resolved six sub-populations and due to sample size constraints we used only four of these separately in our analyses.

Using GWAS, we identified associations between cellulose content and nine regions of the barley genome in three subpopulations of 2-rowed barleys. Analysis of subpopulations 2 and 4, which are comprised of six-rowed barley lines, failed to identify any significant associations with cellulose content using FDR correction. This was likely due to limitations of our GWAS in terms of numbers of lines and numbers of SNPs used. This also indicates a relatively narrow gene pool, with the North Dakota 6 rowed program (N6) which constitutes subpopulation 2 having the narrowest genetic diversity [25]. Consequently greater population size, and, or marker density would likely provide higher confidence in our genetic results. However, despite the relatively low genetic resolution, by using the barley genome assembly [39] we were able to anchor the regions in which associations were detected, and screen gene annotations within them for putative candidate genes that could possibly be related to cellulose content. This provided us with a list of genes, and we subsequently used a gene expression atlas to further investigate their candidature based on tissue specific expression and patterns of co-expression with members of the *CesA* gene family and other genes known to be involved in cellulose synthesis.

While several GWAS studies concerning cellulose content have been carried out to date [23,46–48], none to our knowledge have specifically focused on barley. However, it has been the focus of QTL mapping studies taking a biparental mapping population approach [49–54]. These studies allowed us to compare the associations identified in our current work with the work of previous authors, and gauge how many of our associations are supported by the literature, and how many appeared novel/specific to the germplasm we have surveyed. Previous studies did identify QTLs collocating with either members of gene families highlighted in our current analysis, and/or genes that we observed were co-expressed with our candidate genes. Two QTL studies in *Eucalyptus nitens* [48,49] consisting of 420 and 296 individuals, respectively, identified a QTL in a region of the genome which includes *EniCOBL4*, an ortholog of *AtCOBL4*, which is in the same gene family as the two *COBRA* genes identified in our analysis. In poplar, the ortholog of one of the *CesA* genes that is implicated in secondary cell wall synthesis and co-expressed with genes identified in our analysis, *PtiCesA7-B*, was also found to be associated with cellulose content in a GWAS [23]. Collocation of a QTL for cellulose content and hemicellulose content in sorghum was reported in two bi-parental mapping populations [53,54]. Moreover, a recent GWAS for (1,3:1,4)-β-glucan content in the CAP populations [55] identified a QTL for this trait which maps to the same cM position as the most significant marker defining QCel7H1 reported in the current study.

Leveraging the barley genome assembly and published (and unpublished) RNA-seq expression data [39], we were able to highlight genes potentially contributing to variation in cellulose content based on annotation and subsequently by comparing their expression patterns to the *CesA* genes that are central to cellulose synthesis. This identified a group of co-expressed genes linked to primary cell wall cellulose synthesis and a second group involved in secondary cell wall development. For the primary cell wall group, there is also evidence from the literature that the genes highlighted by our analysis are co-expressed, and potentially involved in a regulatory complex [9]. *HvCobra1*, a likely ortholog of *ZmBk2L3*, has been shown in maize to be co-expressed with primary cell wall *CesA* genes [56]. The *HvCslF6* gene is down-regulated by a small interfering RNA antisense transcript of *HvCesA6*, illustrating the link between several of these co-expressed genes [57]. The current work provides independent evidence of the co-expression of these genes, and their possible involvement in enzyme-protein complexes.

The secondary cell wall association group includes *HvCesA4*, *HvCesA7*, *HvCesA8*, *HvChitinase*, and *HvGT1* (MLOC_66574) (Table 3). The *Arabidopsis* homolog of *HvChitinase*, *AtPom1*, has previously been shown to be co-expressed with genes involved in primary cell wall development in seedlings and mature roots, rosette leaves, various floral tissues, and siliques, and has been described as having a role in cellulose microfibril assembly [58]. However, while we observed similar patterns of expression to the primary cell wall *CesA* genes in equivalent tissues, a survey of the 16 tissues available from the barley genome assembly [39] we revealed a strong trend of co-expression with the secondary cell wall *CesA* genes. This does not disprove the hypothesis of Sanchez-Rodriguez et al., [58], that chitinases such as *AtPom1* bind to cellulose microfibrils during assembly to regulate this process, but instead suggests a role in both processes, depending on the tissue. It must be noted that plants and their cell walls do not contain chitin and that the chitinases have long been identified as pathogenesis-related proteins that are involved in defence against fungal pathogens [59]. Finally, a putative role for the *HvGT1*, a UDP-glucosyltransferase, in secondary cell wall synthesis has yet to be proposed, however the enzyme is has been annotated as a 3-O-glucosyltransferase and a UDP-glucosyl transferase [14] and may be involved in the provision of the UDP-glucose donor substrate for cellulose biosynthesis.

Two regions associated with cellulose content, QCel6H1 and QCel7H1, include members of glycosyl transferase family 2, namely *HvCesA9* on chromosome 6H and *HvCslF6* on chromosome 7H. In addition, QCel5H3 on chromosome 5H contained genes annotated as *COBRA—LIKE* (*COBL*). There is evidence from mutant studies that the *COBRA* gene family influences cellulose content based on a reduction in cellulose content in *cobl* mutants. Schindelman et al.[60], described *COB* mutants as having reduced cellulose content in the elongation zone of the root where the orientation of cell expansion increased in a radial direction compared to the wild type (that exhibited longitudinal expansion in this zone). Based on work using the *cob-1* Arabidopsis mutant, *COB* was subsequently implicated in the deposition of cellulose microfibrils in the primary cell wall of developing roots [61]. MLOC_55526, the likely ortholog of *AtCOB* [57], falls within the interval identified on chromosome 5H, which is significantly associated with cellulose content. This gene is also expressed highly in the 3^rd^ internode of the culm (482.2 FPKM) and is co-expressed with three members of the CesA family, *ZmCesA1*, *ZmCesA2*, and *ZmCes6*. MLOC_11345.4 is the best blastn hit for *ZmBk2L3*, a maize gene previously shown to be co-expressed with the *CesA* genes. *Bk2* is another gene that encodes a member of the *COBRA LIKE* family, a likely ortholog of *AtCobL4*, and shows high levels of correlated co-expression with *AtCesA10*, *AtCesA11*, and *AtCesA12*, genes involved in secondary cell wall development. Plants with the mutant allele of *Bk2* show a reduction in cellulose content in the culm and this is thought to be due to a fault in the cellulose synthase complex. Ultimately, this not only reduces the cellulose content but leads to a reduction in mechanical strength of the culm [62]. The mechanism by which a *COBRA* co-expressed with the secondary cell wall associated *CesA* genes influences cellulose crystallization has recently been described [63]. The NH_2_- terminal region of the protein encoded by *Brittle Culm1* (*BC1*), a *COBRA-LIKE* gene, contains a carbohydrate binding module that interacts with crystalline cellulose and determines the size of microfibril crystallite size, regulating cellulose assembly [63]. To our knowledge the equivalent mechanism for the *COBRA-LIKE* genes associated with primary cell wall *CesA* genes is yet to be described.

We identified additional genes in regions associated with cellulose content that are known to have an impact on secondary cell wall development in barley. QCel5H1 in population 5 on chromosome 5H co-locates with the introgression of *fs1* [40]. Similarly, a region identified as being associated with cellulose content on chromosome 7H, QCel7H1 in population 3, overlaps with the introgression known to contain *fs3* [40]. Both *fs1* and *fs3* mutant accessions have reduced mechanical strength in their culms; a phenotype commonly linked to mutations in genes involved in secondary cell wall synthesis. Burton et al. [13] identified GH17 genes as being differentially transcribed in the maturation zone of internodes of stems from a wild type barley line (Kobinkatagi (J066)) and M382, a near isogenic *fs3* mutant line. The same authors [13] also showed that the fragile stem phenotype of *fs2*, not identified in the current study, was due to a retroelement in the *HvCesA4* gene, thereby illustrating the link between the fragile stem loci and key genes known to determine cellulose content.

Three of these nine regions contain members of the glycoside hydrolase families GH1, GH3, GH9, and GH19, which have previously been implicated in cellulose synthesis and degradation [64]. Several glycoside hydrolase (GH) families are capable of hydrolysing (1,4)-β-glycosidic linkages in cellulose and other polysaccharides, together with oligosaccharide products generated by endo-hydrolase activity [14]. Therefore, (1,4)-β-glucan endohydrolases (cellulases) of the GH9 families can hydrolyse cellulose chains, although their activity on crystalline cellulose is generally low. Certain β-glucosidases and β-glucan exohydrolases of the GH1 and GH3 families hydrolyse oligosaccharides derived from cellulose by endo-hydrolase action. An association identified on chromosome 6H contained a GH9 gene, which includes the endo-(1,4)-β-glucanases (cellulases)[64]. By constitutively expressing a poplar cellulase gene (*PaPopCel1*) in *Arabidopsis thaliana*, Park et al., [65] concluded that this group of enzymes trims noncrystalline regions of a cellulose molecule, releasing the trapped xyloglucan from the cellulose microfibrils. Cosgrove [2] also highlighted the potential role of the cellulase gene *KOR* in the maintenance of normal development of cellulose microtubules in terms of proper crystallisation into microfibrils. QCel5H1 on chromosome 5H contained an ortholog of *AtCTL1*, which is a GH19 (*HvChitinase*) and has been implicated in cellulose synthesis and assembly [59, 66] in both the primary and secondary cell walls [67].

We carried out our GWAS using subpopulations identified by rigorous population genetics analysis as described in [68]. We did also consider whether the Q +K method in Tassel version 5.2.6 [69]would be suitable for this germplasm set using a trait, row type, which is both genetically well characterised and simple compared to cellulose content. Row type is represented by equal numbers of two rowed and six rowed accessions in our germplasm, and the key genes influencing this trait are either cloned i.e. *HvVrs1* on 2H [70], *HvInt*.*c* on 4H [71], *HvVrs4* on 3H [72], or have been delimited to a small interval i.e. *HvVrs2* on 5H and *HvVrs3* on 1H [40]. We know from Ramsay et al., [71] that taking a population of two and six rowed lines can detect row type genes when a kinship matrix is applied. However even if we used a relaxed adjusted p- value after FDR of p<0.05 no significant associations were identified (S2 Fig, S6 Table). The strongest association with row type was identified using marker 12_30880 on 7H, with a—log10 (p) = 3.42, and (non-significant) adjusted p value = 0.53. We also observed very different patterns of LD between subpopulations and with different regions showed LD depending on subpopulation. A much faster rate of LD decay was observed when the dataset was analysed as whole compared to when subpopulations were analysed separately, concurrent with Mohammadi et al. [73]. Taken together with the clear divisions within this germplasm evident from population structure analysis we decided that analysing all lines together might “dilute” any potential population specific marker trait associations.

## Conclusion

In summary, the GWAS analysis revealed a number of candidate genes that are likely to influence cellulose synthesis in barley. Participation of the products of these genes in cellulose biosynthesis has, in many cases, been validated in previous QTL analyses of biparental mapping populations and through biochemical, co-expression and other approaches that link these genes to the cellulose synthesis process. Of particular importance is the possibility that one of the products of these candidate genes interacts directly with the CESA enzyme to form an active complex. Despite the best attempts of many groups around the world over several decades, expression of *CesA* genes in cell-free systems has rarely produced credible levels of cellulose synthesis *in vitro*. A possible explanation for this has been presented by Morgan et al. [74] who showed that the cellulose synthase enzyme (designated BcsA) in the bacterium *Rhodobacter* was inactive in the absence of a second protein (BcsB) and solved the three-dimensional structure of the two co-crystallised proteins of the active complex. If higher plant CESA enzymes also require a second partner to form an active cellulose synthase complex, one of the candidate genes identified in the present GWAS analysis might encode the ‘missing’ partner protein. This possibility can now be explored through further validation of the role of these candidate genes using transgenesis and directed mutagenesis approaches, co-expression in heterologous expression systems and through *in vitro* assays.

## Supporting Information

S1 Fig Bar plot outputs from STRUCTURE for K values ranging from 2 (A) and 7 (F). Breeding program 1 = Washington (WA), 2 = Montana (MT), 3 = 2- row North Dakota (N2), 4 = Utah (UT), 5 = 6-row North Dakota (N6), and 6 = Minnesota (MN). Q value represents proportion of ancestry to a given subpopulation.
(EPS)

S2 Fig Manhattan plot of GWAS of row type using Q+K matrix with all lines. (EPS)

S1 Table Details of germplasm used including breeding program, row type and primary use. * = included in this study.
(DOCX)

S2 Table Assigning populations of the germplasm from six barley CAP breeding programs based on population genetics analysis. The probability that an individual belongs to a population, Q = 0.6, and the number of subpopulations within the dataset, Δ*K* = 6. Numbers in brackets representing the number of lines from each breeding program assigned into subpopulation.
(DOCX)

S3 Table A full list of lines assigned to each subpopulation using the criteria described for Supporting information 2. (DOCX)

S4 Table Raw and FDR adjusted P values for populations with significant associations (Populations 3 and 5). Marker positions are as described in Comadran et al [36].
(DOCX)

S5 Table Regression analysis between candidate genes identified by GWAS and the primary and secondary cell wall *Cellulose Synthase A* (*CesA*) genes. Regression analysis of the rice orthologs of these genes is included for comparison.
(DOCX)

S6 Table Raw and FDR adjusted P values for row type GWAS using Q +K matrix method. Marker positions are as described in Comadran et al [36].
(DOCX)
