# Supplementary material for: A Genome-Wide Association Study for Culm Cellulose Content in Barley Reveals Candidate Genes Co-Expressed with Members of the CELLULOSE SYNTHASE A Gene Family
Source: PLoS One. 2015 Jul 8;10(7):e0130890. doi: 10.1371/journal.pone.0130890 (PMC4496100; doi:10.1371/journal.pone.0130890)
Supplement: S1 Table — * = included in this study. (DOCX) [file pone.0130890.s003.docx]

| Breeding Program | No. of lines* | Row type | Growth habit | Main use |
| --- | --- | --- | --- | --- |
| Minnesota (MN) | 96 | Six | Spring | Malting |
| Montana (MT) | 96 | Two | Spring | Malting/ feed |
| North Dakota (N2) | 96 | Two | Spring | Malting |
| North Dakota (N6) | 96 | Six | Spring | Malting |
| Utah (UT) | 96 | Six | Spring | Feed |
| Washington (WA) | 94 | Two* | Spring | Malting/ feed |
