# Supplementary material for: A Genome-Wide Association Study for Culm Cellulose Content in Barley Reveals Candidate Genes Co-Expressed with Members of the CELLULOSE SYNTHASE A Gene Family
Source: PLoS One. 2015 Jul 8;10(7):e0130890. doi: 10.1371/journal.pone.0130890 (PMC4496100; doi:10.1371/journal.pone.0130890)
Supplement: S2 Table — The probability that an individual belongs to a population, Q = 0.6, and the number of subpopulations within the dataset, ΔK = 6. Numbers in brackets representing the number of lines from each breeding program assigned into subpopulation. (DOCX) [file pone.0130890.s004.docx]

| Subpopulation | Breeding program | Number of lines |
| --- | --- | --- |
| 1 | UT (18) | 18 |
| 2 | N6 (88) | 88 |
| 3 | N2 (91) MT(7) | 98 |
| 4 | MN (96) N6 (1) MT (1) | 98 |
| 5 | WA (94) MT (87) UT (1) N2 (1) | 183 |
| 6 | UT (58) | 58 |
| Not assigned | UT (19) N6 (7) N2 (4) MT (1) | 31 |
