# Supplementary material for: A Genome-Wide Association Study for Culm Cellulose Content in Barley Reveals Candidate Genes Co-Expressed with Members of the CELLULOSE SYNTHASE A Gene Family
Source: PLoS One. 2015 Jul 8;10(7):e0130890. doi: 10.1371/journal.pone.0130890 (PMC4496100; doi:10.1371/journal.pone.0130890)
Supplement: S3 Table — (DOCX) [file pone.0130890.s005.docx]

| Subpopulation | Breeding program | Line name |
| --- | --- | --- |
| 1 | UT | 2153-107 |
| 1 | UT | 2153-23 |
| 1 | UT | 2153-68 |
| 1 | UT | 2153-40 |
| 1 | UT | 2153-52 |
| 1 | UT | 2153-56 |
| 1 | UT | 2156-73 |
| 1 | UT | 2153-41 |
| 1 | UT | 2153-70 |
| 1 | UT | 2153-30 |
| 1 | UT | 2157-26 |
| 1 | UT | 2153-61 |
| 1 | UT | 2153-67 |
| 1 | UT | 2156-46 |
| 1 | UT | 2156-8 |
| 1 | UT | 2156-59 |
| 1 | UT | 2156-12 |
| 1 | UT | 2157-69 |
| 2 | N6 | ND25999 |
| 2 | N6 | ND25826 |
| 2 | N6 | ND25832 |
| 2 | N6 | ND25843 |
| 2 | N6 | ND26010 |
| 2 | N6 | ND26062 |
| 2 | N6 | ND26090 |
| 2 | N6 | ND26092 |
| 2 | N6 | ND26100 |
| 2 | N6 | ND26105 |
| 2 | N6 | ND26111 |
| 2 | N6 | ND26284 |
| 2 | N6 | ND25825 |
| 2 | N6 | ND25835 |
| 2 | N6 | ND25839 |
| 2 | N6 | ND25840 |
| 2 | N6 | ND25982 |
| 2 | N6 | ND25989 |
| 2 | N6 | ND25998 |
| 2 | N6 | ND26009 |
| 2 | N6 | ND26018 |
| 2 | N6 | ND26058 |
| 2 | N6 | ND26085 |
| 2 | N6 | ND25824 |
| 2 | N6 | ND26055 |
| 2 | N6 | ND26181 |
| 2 | N6 | ND26086 |
| 2 | N6 | ND26104 |
| 2 | N6 | ND26017 |
| 2 | N6 | ND26032 |
| 2 | N6 | ND25988 |
| 2 | N6 | ND25986 |
| 2 | N6 | ND25996 |
| 2 | N6 | ND20448 |
| 2 | N6 | ND26037 |
| 2 | N6 | ND26030 |
| 2 | N6 | ND26303 |
| 2 | N6 | ND25908 |
| 2 | N6 | ND26139 |
| 2 | N6 | ND25979 |
| 2 | N6 | ND26229 |
| 2 | N6 | ND25917 |
| 2 | N6 | ND26033 |
| 2 | N6 | ND25882 |
| 2 | N6 | ND25949 |
| 2 | N6 | ND26015 |
| 2 | N6 | ND26036 |
| 2 | N6 | ND26180 |
| 2 | N6 | ND26300 |
| 2 | N6 | ND26034 |
| 2 | N6 | ND24906 |
| 2 | N6 | ND26239 |
| 2 | N6 | ND26205 |
| 2 | N6 | ND25786 |
| 2 | N6 | ND25030 |
| 2 | N6 | ND26292 |
| 2 | N6 | ND26048 |
| 2 | N6 | ND25911 |
| 2 | N6 | ND24843 |
| 2 | N6 | ND26178 |
| 2 | N6 | ND25782 |
| 2 | N6 | ND24890 |
| 2 | N6 | ND25975 |
| 2 | N6 | ND25976 |
| 2 | N6 | ND25977 |
| 2 | N6 | ND25805 |
| 2 | N6 | ND25205 |
| 2 | N6 | ND25220 |
| 2 | N6 | ND25969 |
| 2 | N6 | ND25152 |
| 2 | N6 | ND25151 |
| 2 | N6 | ND26208 |
| 2 | N6 | ND25768 |
| 2 | N6 | ND25161 |
| 2 | N6 | ND25172 |
| 2 | N6 | ND26249 |
| 2 | N6 | ND26289 |
| 2 | N6 | ND26288 |
| 2 | N6 | ND25208 |
| 2 | N6 | ND25153 |
| 2 | N6 | ND25967 |
| 2 | N6 | ND25966 |
| 2 | N6 | ND25160 |
| 2 | N6 | ND25165 |
| 2 | N6 | ND25148 |
| 2 | N6 | ND25970 |
| 2 | N6 | ND26251 |
| 2 | N6 | ND25163 |
| 3 | MT | MT08053 |
| 3 | MT | MT08056 |
| 3 | MT | MT08061 |
| 3 | MT | MT08047 |
| 3 | MT | MT08044 |
| 3 | MT | MT08055 |
| 3 | MT | MT08152 |
| 3 | N2 | ND27345 |
| 3 | N2 | ND27373 |
| 3 | N2 | ND27375 |
| 3 | N2 | ND27378 |
| 3 | N2 | ND27381 |
| 3 | N2 | ND27395 |
| 3 | N2 | ND27397 |
| 3 | N2 | ND27402 |
| 3 | N2 | ND27422 |
| 3 | N2 | ND27388 |
| 3 | N2 | ND27405 |
| 3 | N2 | ND27336 |
| 3 | N2 | ND27341 |
| 3 | N2 | ND27346 |
| 3 | N2 | ND27350 |
| 3 | N2 | ND27351 |
| 3 | N2 | ND27352 |
| 3 | N2 | ND27361 |
| 3 | N2 | ND27374 |
| 3 | N2 | ND27376 |
| 3 | N2 | ND27377 |
| 3 | N2 | ND27379 |
| 3 | N2 | ND27380 |
| 3 | N2 | ND27387 |
| 3 | N2 | ND27389 |
| 3 | N2 | ND27390 |
| 3 | N2 | ND27392 |
| 3 | N2 | ND27393 |
| 3 | N2 | ND27396 |
| 3 | N2 | ND27398 |
| 3 | N2 | ND27399 |
| 3 | N2 | ND27400 |
| 3 | N2 | ND27401 |
| 3 | N2 | ND27403 |
| 3 | N2 | ND27404 |
| 3 | N2 | ND27406 |
| 3 | N2 | ND27407 |
| 3 | N2 | ND27409 |
| 3 | N2 | ND27412 |
| 3 | N2 | ND27372 |
| 3 | N2 | ND27337 |
| 3 | N2 | ND27344 |
| 3 | N2 | ND27408 |
| 3 | N2 | ND27415 |
| 3 | N2 | ND27417 |
| 3 | N2 | ND27394 |
| 3 | N2 | ND27368 |
| 3 | N2 | ND27413 |
| 3 | N2 | ND27421 |
| 3 | N2 | ND27383 |
| 3 | N2 | ND27391 |
| 3 | N2 | ND27410 |
| 3 | N2 | ND27411 |
| 3 | N2 | ND27416 |
| 3 | N2 | ND27382 |
| 3 | N2 | ND27414 |
| 3 | N2 | ND27353 |
| 3 | N2 | ND27342 |
| 3 | N2 | ND27339 |
| 3 | N2 | ND27371 |
| 3 | N2 | ND27348 |
| 3 | N2 | ND27420 |
| 3 | N2 | ND27347 |
| 3 | N2 | ND27338 |
| 3 | N2 | ND27385 |
| 3 | N2 | ND27357 |
| 3 | N2 | ND27384 |
| 3 | N2 | ND27386 |
| 3 | N2 | ND27362 |
| 3 | N2 | ND27354 |
| 3 | N2 | ND27358 |
| 3 | N2 | ND27419 |
| 3 | N2 | ND27359 |
| 3 | N2 | ND27360 |
| 3 | N2 | ND27343 |
| 3 | N2 | ND27335 |
| 3 | N2 | ND27332 |
| 3 | N2 | ND27340 |
| 3 | N2 | ND27355 |
| 3 | N2 | ND27356 |
| 3 | N2 | ND27331 |
| 3 | N2 | ND27333 |
| 3 | N2 | ND27349 |
| 3 | N2 | ND27370 |
| 3 | N2 | ND27334 |
| 3 | N2 | ND27369 |
| 3 | N2 | ND27418 |
| 3 | N2 | ND27328 |
| 3 | N2 | ND27330 |
| 3 | N2 | ND27329 |
| 3 | N2 | ND27364 |
| 4 | MN | FEG180-19 |
| 4 | MN | FEG183-24 |
| 4 | MN | FEG185-31 |
| 4 | MN | FEG189-12 |
| 4 | MN | MN05-07 |
| 4 | MN | FEG179-17 |
| 4 | MN | FEG181-64 |
| 4 | MN | FEG182-15 |
| 4 | MN | FEG189-01 |
| 4 | MN | MN05-21 |
| 4 | MN | FEG179-28 |
| 4 | MN | FEG179-66 |
| 4 | MN | FEG181-09 |
| 4 | MN | FEG181-40 |
| 4 | MN | FEG181-62 |
| 4 | MN | FEG182-37 |
| 4 | MN | FEG183-25 |
| 4 | MN | FEG183-28 |
| 4 | MN | FEG183-51 |
| 4 | MN | FEG185-15 |
| 4 | MN | FEG185-22 |
| 4 | MN | FEG185-32 |
| 4 | MN | FEG185-37 |
| 4 | MN | FEG185-64 |
| 4 | MN | FEG188-02 |
| 4 | MN | FEG188-39 |
| 4 | MN | FEG188-53 |
| 4 | MN | FEG189-05 |
| 4 | MN | FEG189-15 |
| 4 | MN | FEG189-27 |
| 4 | MN | FEG189-67 |
| 4 | MN | FEG190-01 |
| 4 | MN | FEG190-65 |
| 4 | MN | FEG190-67 |
| 4 | MN | MN05-09 |
| 4 | MN | MN05-11 |
| 4 | MN | MN05-12 |
| 4 | MN | MN05-13 |
| 4 | MN | MN05-24 |
| 4 | MN | FEG181-17 |
| 4 | MN | FEG183-53 |
| 4 | MN | FEG181-42 |
| 4 | MN | FEG183-52 |
| 4 | MN | FEG190-08 |
| 4 | MN | FEG181-66 |
| 4 | MN | FEG182-57 |
| 4 | MN | FEG179-18 |
| 4 | MN | FEG178-65 |
| 4 | MN | FEG179-10 |
| 4 | MN | FEG185-46 |
| 4 | MN | FEG185-10 |
| 4 | MN | FEG184-43 |
| 4 | MN | FEG184-50 |
| 4 | MN | FEG182-60 |
| 4 | MN | FEG182-61 |
| 4 | MN | MN05-08 |
| 4 | MN | MN05-04 |
| 4 | MN | MN05-03 |
| 4 | MN | FEG178-05 |
| 4 | MN | MN05-01 |
| 4 | MN | FEG182-31 |
| 4 | MN | MN05-05 |
| 4 | MN | FEG178-16 |
| 4 | MN | FEG192-04 |
| 4 | MN | MN05-16 |
| 4 | MN | FEG184-23 |
| 4 | MN | FEG184-34 |
| 4 | MN | FEG184-35 |
| 4 | MN | MN05-17 |
| 4 | MN | MN05-20 |
| 4 | MN | FEG178-11 |
| 4 | MN | FEG187-41 |
| 4 | MN | FEG178-18 |
| 4 | MN | MN05-15 |
| 4 | MN | FEG178-64 |
| 4 | MN | MN05-19 |
| 4 | MN | FEG178-02 |
| 4 | MN | FEG187-11 |
| 4 | MN | MN05-25 |
| 4 | MN | MN05-31 |
| 4 | MN | FEG178-45 |
| 4 | MN | FEG192-69 |
| 4 | MN | FEG184-11 |
| 4 | MN | FEG192-49 |
| 4 | MN | FEG192-15 |
| 4 | MN | MN05-29 |
| 4 | MN | FEG187-02 |
| 4 | MN | MN05-39 |
| 4 | MN | FEG192-30 |
| 4 | MN | FEG192-42 |
| 4 | MN | FEG192-56 |
| 4 | MN | FEG184-54 |
| 4 | MN | FEG184-24 |
| 4 | MN | FEG184-60 |
| 4 | MN | FEG192-14 |
| 4 | MN | MN05-35 |
| 4 | MT | MT08104 |
| 4 | N6 | ND26212 |
| 5 | MT | MT08165 |
| 5 | MT | MT08284 |
| 5 | MT | MT08285 |
| 5 | MT | MT08248 |
| 5 | MT | MT08249 |
| 5 | MT | MT08279 |
| 5 | MT | MT08281 |
| 5 | MT | MT08282 |
| 5 | MT | MT08283 |
| 5 | MT | MT08273 |
| 5 | MT | MT08277 |
| 5 | MT | MT08085 |
| 5 | MT | MT08083 |
| 5 | MT | MT08087 |
| 5 | MT | MT08166 |
| 5 | MT | MT08084 |
| 5 | MT | MT08077 |
| 5 | MT | MT08153 |
| 5 | MT | MT08236 |
| 5 | MT | MT08232 |
| 5 | MT | MT08270 |
| 5 | MT | MT08211 |
| 5 | MT | MT08239 |
| 5 | MT | MT08231 |
| 5 | MT | MT08238 |
| 5 | MT | MT08154 |
| 5 | MT | MT08164 |
| 5 | MT | MT08155 |
| 5 | MT | MT08173 |
| 5 | MT | MT08189 |
| 5 | MT | MT08011 |
| 5 | MT | MT08206 |
| 5 | MT | MT08203 |
| 5 | MT | MT08220 |
| 5 | MT | MT08217 |
| 5 | MT | MT08221 |
| 5 | MT | MT08198 |
| 5 | MT | MT08199 |
| 5 | MT | MT08197 |
| 5 | MT | MT08106 |
| 5 | MT | MT08230 |
| 5 | MT | MT08170 |
| 5 | MT | MT08200 |
| 5 | MT | MT08208 |
| 5 | MT | MT08005 |
| 5 | MT | MT08107 |
| 5 | MT | MT08142 |
| 5 | MT | MT08257 |
| 5 | MT | MT08190 |
| 5 | MT | MT08119 |
| 5 | MT | MT08112 |
| 5 | MT | MT08184 |
| 5 | MT | MT08156 |
| 5 | MT | MT08243 |
| 5 | MT | MT08192 |
| 5 | MT | MT08179 |
| 5 | MT | MT08160 |
| 5 | MT | MT08177 |
| 5 | MT | MT08006 |
| 5 | MT | MT08010 |
| 5 | MT | MT08272 |
| 5 | MT | MT08193 |
| 5 | MT | MT08351 |
| 5 | MT | MT08262 |
| 5 | MT | MT08254 |
| 5 | MT | MT08113 |
| 5 | MT | MT08370 |
| 5 | MT | MT08255 |
| 5 | MT | MT08120 |
| 5 | MT | MT08129 |
| 5 | MT | MT08258 |
| 5 | MT | MT08182 |
| 5 | MT | MT08128 |
| 5 | MT | MT08300 |
| 5 | MT | MT08290 |
| 5 | MT | MT08292 |
| 5 | MT | MT08291 |
| 5 | MT | MT08289 |
| 5 | MT | MT08372 |
| 5 | MT | MT08327 |
| 5 | MT | MT08353 |
| 5 | MT | MT08328 |
| 5 | MT | MT08161 |
| 5 | MT | MT08260 |
| 5 | MT | MT08275 |
| 5 | MT | MT08305 |
| 5 | MT | MT08256 |
| 5 | N2 | ND27365 |
| 5 | UT | 2156-45 |
| 5 | WA | 06WA-412.29 |
| 5 | WA | 06WA-412.3 |
| 5 | WA | 06WA-448.5 |
| 5 | WA | 06WA-473.17 |
| 5 | WA | 06WA-473.3 |
| 5 | WA | 06WA-473.9 |
| 5 | WA | X02007-DH1 |
| 5 | WA | X02007-DH12 |
| 5 | WA | X02037-DH5 |
| 5 | WA | X02007-DH6 |
| 5 | WA | 06WA-427.6 |
| 5 | WA | 06WA-409.8 |
| 5 | WA | 06WA-412.12 |
| 5 | WA | 06WA-412.14 |
| 5 | WA | 06WA-412.16 |
| 5 | WA | 06WA-412.4 |
| 5 | WA | 06WA-412.5 |
| 5 | WA | 06WA-425.3 |
| 5 | WA | 06WA-427.15 |
| 5 | WA | 06WA-427.31 |
| 5 | WA | 06WA-427.50 |
| 5 | WA | 06WA-431.16 |
| 5 | WA | 06WA-431.25 |
| 5 | WA | 06WA-453.1 |
| 5 | WA | 06WA-455.5 |
| 5 | WA | 06WA-458.10 |
| 5 | WA | 06WA-458.8 |
| 5 | WA | X02035-DH2 |
| 5 | WA | X04055-T3 |
| 5 | WA | X04061-T71 |
| 5 | WA | 06WA-423.30 |
| 5 | WA | 06WA-473.11 |
| 5 | WA | 06WA-423.21 |
| 5 | WA | 06WA-423.25 |
| 5 | WA | X04055-T56 |
| 5 | WA | 06WA-432.7 |
| 5 | WA | 06WA-466.16 |
| 5 | WA | 06WA-406.9 |
| 5 | WA | 03WA-204.22 |
| 5 | WA | 06WA-420.10 |
| 5 | WA | 06WA-426.18 |
| 5 | WA | 06WA-421.17 |
| 5 | WA | 06WA-475.14 |
| 5 | WA | 06WA-469.14 |
| 5 | WA | 06WA-416.21 |
| 5 | WA | X02037-DH1 |
| 5 | WA | 06WA-428.11 |
| 5 | WA | 03WA-204.4W |
| 5 | WA | X04012-T172 |
| 5 | WA | 06WA-409.31 |
| 5 | WA | 06WA-472.13 |
| 5 | WA | 03WA-105.4H |
| 5 | WA | 06WA-415.30 |
| 5 | WA | 06WA-466.6 |
| 5 | WA | 06WA-420.19 |
| 5 | WA | 06WA-406.21 |
| 5 | WA | X04041-T194 |
| 5 | WA | X04041-T15 |
| 5 | WA | X04012-T46 |
| 5 | WA | 06WA-408.14 |
| 5 | WA | 06WA-432.23 |
| 5 | WA | 06WA-458.20 |
| 5 | WA | 06WA-456.9 |
| 5 | WA | X04041-T7 |
| 5 | WA | 03WA-204.19 |
| 5 | WA | X04041-T81 |
| 5 | WA | X04012-T80 |
| 5 | WA | 06WA-419.6 |
| 5 | WA | 06WA-414.7 |
| 5 | WA | 03WA-168.14 |
| 5 | WA | X04041-T113 |
| 5 | WA | X04012-T149 |
| 5 | WA | 06WA-414.23 |
| 5 | WA | 06WA-406.3 |
| 5 | WA | 06WA-475.15 |
| 5 | WA | X04041-T32 |
| 5 | WA | 06WA-470.7 |
| 5 | WA | 03WA-204.9W |
| 5 | WA | 06WA-428.19 |
| 5 | WA | 03WA-203.9H |
| 5 | WA | 06WA-461.1 |
| 5 | WA | 06WA-406.11 |
| 5 | WA | X04012-T51 |
| 5 | WA | 06WA-406.6 |
| 5 | WA | 06WA-406.18 |
| 5 | WA | 06WA-414.10 |
| 5 | WA | 03WA-203.1H |
| 5 | WA | 06WA-422.19 |
| 5 | WA | 06WA-461.23 |
| 5 | WA | 06WA-429.10 |
| 5 | WA | 06WA-444.5 |
| 5 | WA | 06WA-429.16 |
| 5 | WA | 03WA-203.18 |
| 5 | WA | 06WA-414.3 |
| 6 | UT | 2159-117 |
| 6 | UT | 2155-140 |
| 6 | UT | 2155-101 |
| 6 | UT | 2155-16 |
| 6 | UT | 2155-46 |
| 6 | UT | 2155-48 |
| 6 | UT | 2155-54 |
| 6 | UT | 2155-108 |
| 6 | UT | 2155-138 |
| 6 | UT | 2155-139 |
| 6 | UT | 2155-43 |
| 6 | UT | 2155-62 |
| 6 | UT | 2155-73 |
| 6 | UT | 2155-75 |
| 6 | UT | 2159-62 |
| 6 | UT | 2159-72 |
| 6 | UT | 2155-144 |
| 6 | UT | 2155-35 |
| 6 | UT | 2155-66 |
| 6 | UT | 2153-27 |
| 6 | UT | 2159-44 |
| 6 | UT | 2153-11 |
| 6 | UT | 2159-113 |
| 6 | UT | 2159-135 |
| 6 | UT | 2159-99 |
| 6 | UT | 2154-101 |
| 6 | UT | 2159-125 |
| 6 | UT | 2159-103 |
| 6 | UT | 2159-27 |
| 6 | UT | 2154-55 |
| 6 | UT | 2159-47 |
| 6 | UT | 2154-92 |
| 6 | UT | 2159-94 |
| 6 | UT | 2159-132 |
| 6 | UT | 2154-56 |
| 6 | UT | 2159-123 |
| 6 | UT | 2153-4 |
| 6 | UT | 2159-140 |
| 6 | UT | 2154-97 |
| 6 | UT | 2154-9 |
| 6 | UT | 2159-32 |
| 6 | UT | 2154-40 |
| 6 | UT | 2159-133 |
| 6 | UT | 2159-90 |
| 6 | UT | 2152-3 |
| 6 | UT | 2152-68 |
| 6 | UT | 2154-86 |
| 6 | UT | 2154-82 |
| 6 | UT | 2154-26 |
| 6 | UT | 2154-58 |
| 6 | UT | 2154-102 |
| 6 | UT | 2152-113 |
| 6 | UT | 2152-50 |
| 6 | UT | 2152-99 |
| 6 | UT | 2154-88 |
| 6 | UT | 2152-87 |
| 6 | UT | 2152-29 |
| 6 | UT | 2152-32 |
|  |  |  |
