# Supplementary material for: A Genome-Wide Association Study for Culm Cellulose Content in Barley Reveals Candidate Genes Co-Expressed with Members of the CELLULOSE SYNTHASE A Gene Family
Source: PLoS One. 2015 Jul 8;10(7):e0130890. doi: 10.1371/journal.pone.0130890 (PMC4496100; doi:10.1371/journal.pone.0130890)
Supplement: S4 Table — Marker positions are as described in Comadran et al [36]. (DOCX) [file pone.0130890.s006.docx]

| Population | Marker | Chr | cM | Kinship -log 10 (p) | p-value | q-value |
| --- | --- | --- | --- | --- | --- | --- |
| 3 | 11_20149 | 1H | 0 | 0.269 | 0.53827 | 0.754654 |
| 3 | 11_20373 | 1H | 0 | 0.221 | 0.601174 | 0.769383 |
| 3 | 12_30969 | 1H | 0 | 0.161 | 0.69024 | 0.795231 |
| 3 | 11_11223 | 1H | 0.8 | 0.61 | 0.785236 | 0.836619 |
| 3 | 11_21354 | 1H | 0.8 | 0.105 | 0.245471 | 0.641355 |
| 3 | 11_21067 | 1H | 1.5 | 0.251 | 0.561048 | 0.759523 |
| 3 | 11_10419 | 1H | 3.8 | 1.192 | 0.542001 | 0.754654 |
| 3 | 12_10410 | 1H | 3.8 | 0.266 | 0.064269 | 0.582372 |
| 3 | 12_31144 | 1H | 3.8 | 0.109 | 0.778037 | 0.832768 |
| 3 | 12_10636 | 1H | 4.5 | 0.033 | 0.92683 | 0.869833 |
| 3 | 12_11011 | 1H | 6 | 0.033 | 0.92683 | 0.869833 |
| 3 | 12_30933 | 1H | 6 | 0.033 | 0.92683 | 0.869833 |
| 3 | 11_21174 | 1H | 8.3 | 0.034 | 0.924698 | 0.869833 |
| 3 | 11_21226 | 1H | 8.8 | 0.562 | 0.274157 | 0.641355 |
| 3 | 12_30817 | 1H | 10.7 | 1.025 | 0.993116 | 0.875778 |
| 3 | 12_31149 | 1H | 10.7 | 0.003 | 0.094406 | 0.582372 |
| 3 | 12_30918 | 1H | 11.4 | 0.732 | 0.185353 | 0.606353 |
| 3 | 12_30951 | 1H | 11.4 | 0.625 | 0.237137 | 0.641355 |
| 3 | 12_30588 | 1H | 13.1 | 1.335 | 0.046238 | 0.582372 |
| 3 | 11_10332 | 1H | 15.4 | 0.697 | 0.200909 | 0.630348 |
| 3 | 12_30948 | 1H | 17.3 | 1.245 | 0.056885 | 0.582372 |
| 3 | 11_10030 | 1H | 18.1 | 0.583 | 0.261216 | 0.641355 |
| 3 | 11_20712 | 1H | 20.8 | 0.403 | 0.395367 | 0.684276 |
| 3 | 11_10873 | 1H | 20.9 | 0.462 | 0.345144 | 0.648583 |
| 3 | 11_10186 | 1H | 23.9 | 0.327 | 0.470977 | 0.717793 |
| 3 | 11_10275 | 1H | 42.5 | 0.028 | 1 | 0.876472 |
| 3 | 11_10597 | 1H | 42.5 | 0.001 | 0.9977 | 0.875778 |
| 3 | 11_20514 | 1H | 42.5 | 0 | 0.937562 | 0.871665 |
| 3 | 11_10259 | 1H | 47.5 | 0.14 | 0.724436 | 0.808171 |
| 3 | 11_10526 | 1H | 47.5 | 0.085 | 0.822243 | 0.861053 |
| 3 | 11_10438 | 1H | 50 | 0.03 | 0.933254 | 0.869833 |
| 3 | 11_20660 | 1H | 50 | 0.03 | 0.933254 | 0.869833 |
| 3 | 11_20427 | 1H | 50.6 | 0.03 | 0.933254 | 0.869833 |
| 3 | 12_31208 | 1H | 50.6 | 0.03 | 0.933254 | 0.869833 |
| 3 | 12_31381 | 1H | 50.6 | 0.03 | 0.933254 | 0.869833 |
| 3 | 11_11484 | 1H | 51.2 | 0.121 | 0.756833 | 0.824512 |
| 3 | 11_10833 | 1H | 52.5 | 0.012 | 0.756833 | 0.824512 |
| 3 | 11_20810 | 1H | 52.5 | 0.121 | 0.972747 | 0.875024 |
| 3 | 11_20912 | 1H | 52.5 | 0.191 | 0.972747 | 0.875024 |
| 3 | 11_21000 | 1H | 52.5 | 0.012 | 0.772681 | 0.83201 |
| 3 | 11_21312 | 1H | 52.5 | 0.121 | 0.644169 | 0.773384 |
| 3 | 12_11169 | 1H | 52.5 | 0.121 | 0.972747 | 0.875024 |
| 3 | 12_30592 | 1H | 52.5 | 0.112 | 0.756833 | 0.824512 |
| 3 | 12_31134 | 1H | 52.5 | 0.012 | 0.644169 | 0.773384 |
| 3 | 12_31272 | 1H | 52.5 | 0.191 | 0.756833 | 0.824512 |
| 3 | 11_11359 | 1H | 54.7 | 0.078 | 0.972747 | 0.875024 |
| 3 | 12_30672 | 1H | 54.7 | 0.012 | 0.835603 | 0.861053 |
| 3 | 12_30786 | 1H | 54.7 | 0.078 | 0.835603 | 0.861053 |
| 3 | 11_10075 | 1H | 55.5 | 0.191 | 0.644169 | 0.773384 |
| 3 | 11_10520 | 1H | 55.5 | 0.066 | 0.972747 | 0.875024 |
| 3 | 11_10933 | 1H | 55.5 | 0.012 | 0.92683 | 0.869833 |
| 3 | 11_20798 | 1H | 55.5 | 0.012 | 0.931108 | 0.869833 |
| 3 | 11_21361 | 1H | 55.5 | 0.033 | 0.859014 | 0.865709 |
| 3 | 12_30110 | 1H | 55.5 | 0.066 | 0.972747 | 0.875024 |
| 3 | 12_30406 | 1H | 55.5 | 0.012 | 0.972747 | 0.875024 |
| 3 | 12_30478 | 1H | 55.5 | 0.031 | 0.859014 | 0.865709 |
| 3 | 12_30499 | 1H | 55.5 | 0.066 | 0.859014 | 0.865709 |
| 3 | 12_30694 | 1H | 55.5 | 0.078 | 0.972747 | 0.875024 |
| 3 | 12_30750 | 1H | 55.5 | 0.012 | 0.835603 | 0.861053 |
| 3 | 12_30710 | 1H | 56.2 | 0.012 | 0.972747 | 0.875024 |
| 3 | 11_10324 | 1H | 57 | 0.037 | 0.918333 | 0.869833 |
| 3 | 12_10198 | 1H | 57.8 | 0.059 | 0.872971 | 0.866507 |
| 3 | 11_10552 | 1H | 59.7 | 0.046 | 0.899498 | 0.869833 |
| 3 | 11_20997 | 1H | 60.2 | 0.061 | 0.86896 | 0.866507 |
| 3 | 11_20095 | 1H | 60.8 | 0.036 | 0.92045 | 0.869833 |
| 3 | 11_10617 | 1H | 61.5 | 0.052 | 0.887156 | 0.866507 |
| 3 | 11_10798 | 1H | 61.5 | 0.052 | 0.887156 | 0.866507 |
| 3 | 11_11049 | 1H | 61.5 | 0.052 | 0.887156 | 0.866507 |
| 3 | 12_30304 | 1H | 61.5 | 0.052 | 0.887156 | 0.866507 |
| 3 | 12_31179 | 1H | 63.5 | 0.036 | 0.92045 | 0.869833 |
| 3 | 12_31401 | 1H | 63.5 | 0.036 | 0.92045 | 0.869833 |
| 3 | 11_20432 | 1H | 64.3 | 0.194 | 0.639735 | 0.773384 |
| 3 | 11_20642 | 1H | 64.9 | 0.194 | 0.639735 | 0.773384 |
| 3 | 11_21431 | 1H | 64.9 | 0.194 | 0.639735 | 0.773384 |
| 3 | 11_10516 | 1H | 65.5 | 0.799 | 0.158855 | 0.59212 |
| 3 | 11_10043 | 1H | 66 | 0.799 | 0.158855 | 0.59212 |
| 3 | 12_10166 | 1H | 69.5 | 0.501 | 0.315501 | 0.648493 |
| 3 | 12_11267 | 1H | 72.4 | 0.728 | 0.187068 | 0.608234 |
| 3 | 11_10006 | 1H | 73.9 | 0.792 | 0.161436 | 0.593148 |
| 3 | 11_21126 | 1H | 73.9 | 0.792 | 0.161436 | 0.593148 |
| 3 | 11_10466 | 1H | 80.3 | 0.746 | 0.179473 | 0.601743 |
| 3 | 12_30072 | 1H | 83.3 | 0.741 | 0.181552 | 0.601743 |
| 3 | 11_11037 | 1H | 84.7 | 0.027 | 0.939723 | 0.871665 |
| 3 | 12_11463 | 1H | 85.4 | 0.741 | 0.181552 | 0.601743 |
| 3 | 11_10434 | 1H | 86.2 | 0.746 | 0.179473 | 0.601743 |
| 3 | 12_30204 | 1H | 86.2 | 0.073 | 0.845279 | 0.861565 |
| 3 | 12_11144 | 1H | 87.6 | 0.32 | 0.47863 | 0.725647 |
| 3 | 11_10471 | 1H | 88.2 | 0.269 | 0.53827 | 0.754654 |
| 3 | 11_10830 | 1H | 88.2 | 0.25 | 0.695024 | 0.796791 |
| 3 | 11_20434 | 1H | 88.2 | 0.158 | 0.53827 | 0.754654 |
| 3 | 11_21192 | 1H | 88.2 | 0.269 | 0.562341 | 0.759523 |
| 3 | 11_11189 | 1H | 91 | 0.675 | 0.413048 | 0.700267 |
| 3 | 11_20792 | 1H | 91 | 0.384 | 0.211349 | 0.641355 |
| 3 | 11_21446 | 1H | 92 | 0.583 | 0.261216 | 0.641355 |
| 3 | 12_10535 | 1H | 92 | 0.43 | 0.371535 | 0.661769 |
| 3 | 11_20475 | 1H | 92.8 | 0.755 | 0.175792 | 0.601743 |
| 3 | 11_10433 | 1H | 94 | 0.755 | 0.175792 | 0.601743 |
| 3 | 12_31163 | 1H | 94 | 0.269 | 0.53827 | 0.754654 |
| 3 | 11_21373 | 1H | 95.4 | 0.364 | 0.432514 | 0.70745 |
| 3 | 11_10396 | 1H | 96.9 | 0.74 | 0.18197 | 0.601743 |
| 3 | 11_20769 | 1H | 96.9 | 0.583 | 0.261216 | 0.641355 |
| 3 | 11_11277 | 1H | 97.7 | 0.74 | 0.699842 | 0.79759 |
| 3 | 12_31319 | 1H | 97.7 | 0.155 | 0.18197 | 0.601743 |
| 3 | 11_20780 | 1H | 105.1 | 0.488 | 0.325087 | 0.648583 |
| 3 | 11_20921 | 1H | 105.8 | 1.112 | 0.077268 | 0.582372 |
| 3 | 11_20625 | 1H | 106.6 | 0.699 | 0.199986 | 0.629401 |
| 3 | 11_20220 | 1H | 107.5 | 0.361 | 0.435512 | 0.708363 |
| 3 | 11_20844 | 1H | 108.3 | 0.361 | 0.435512 | 0.708363 |
| 3 | 12_30532 | 1H | 109.8 | 0.278 | 0.52723 | 0.753599 |
| 3 | 12_31526 | 1H | 112.9 | 1.15 | 0.070795 | 0.582372 |
| 3 | 11_21392 | 1H | 114.8 | 0.702 | 0.19861 | 0.628212 |
| 3 | 11_10729 | 1H | 116.3 | 0.005 | 0.988553 | 0.875778 |
| 3 | 11_10338 | 1H | 117.8 | 0.849 | 0.141579 | 0.59212 |
| 3 | 11_10854 | 1H | 117.8 | 0.482 | 0.32961 | 0.648583 |
| 3 | 11_20959 | 1H | 117.8 | 0.455 | 0.350752 | 0.650698 |
| 3 | 11_10911 | 1H | 120.5 | 0.574 | 0.266686 | 0.641355 |
| 3 | 11_20908 | 1H | 121.1 | 0.574 | 0.909913 | 0.869833 |
| 3 | 11_21038 | 1H | 121.1 | 0.041 | 0.159588 | 0.59212 |
| 3 | 12_21172 | 1H | 121.1 | 0.797 | 0.266686 | 0.641355 |
| 3 | 11_10586 | 1H | 121.8 | 0.13 | 0.74131 | 0.817571 |
| 3 | 11_10722 | 1H | 125.3 | 0.043 | 0.905733 | 0.869833 |
| 3 | 11_21140 | 1H | 126 | 0.104 | 0.787046 | 0.837779 |
| 3 | 12_10207 | 1H | 126.5 | 0.629 | 0.357273 | 0.654711 |
| 3 | 12_30403 | 1H | 126.5 | 0.447 | 0.234963 | 0.641355 |
| 3 | 12_31377 | 1H | 126.5 | 0.629 | 0.234963 | 0.641355 |
| 3 | 11_10644 | 1H | 127.1 | 0.069 | 0.8531 | 0.863755 |
| 3 | 12_31387 | 1H | 127.4 | 0.797 | 0.159588 | 0.59212 |
| 3 | 12_10693 | 1H | 128.1 | 0.972 | 0.10666 | 0.59212 |
| 3 | 11_20383 | 1H | 131.2 | 0.446 | 0.358096 | 0.654711 |
| 3 | 11_10041 | 1H | 135.6 | 0.513 | 0.685488 | 0.793694 |
| 3 | 11_11105 | 1H | 135.6 | 0.54 | 0.578096 | 0.759523 |
| 3 | 11_11509 | 1H | 135.6 | 0.164 | 0.288403 | 0.641355 |
| 3 | 11_20603 | 1H | 135.6 | 0.238 | 0.288403 | 0.641355 |
| 3 | 12_11496 | 1H | 135.6 | 0.54 | 0.306902 | 0.641355 |
| 3 | 11_20594 | 1H | 136.3 | 0.213 | 0.61235 | 0.769383 |
| 3 | 11_20840 | 1H | 137.8 | 0.168 | 0.679204 | 0.79115 |
| 3 | 12_30231 | 1H | 138.3 | 0.177 | 0.665273 | 0.783785 |
| 3 | 11_20772 | 1H | 139.8 | 0.81 | 0.154882 | 0.59212 |
| 3 | 12_31081 | 1H | 140.5 | 0.069 | 0.8531 | 0.863755 |
| 3 | 11_10352 | 2H | 0 | 0.531 | 0.294442 | 0.641355 |
| 3 | 11_10977 | 2H | 0 | 0.565 | 0.27227 | 0.641355 |
| 3 | 11_11346 | 2H | 0 | 0.531 | 0.696627 | 0.79759 |
| 3 | 11_20099 | 2H | 0 | 0.157 | 0.210378 | 0.641355 |
| 3 | 11_20498 | 2H | 0 | 0.429 | 0.512861 | 0.749181 |
| 3 | 11_20609 | 2H | 0 | 0.29 | 0.372392 | 0.662279 |
| 3 | 11_21184 | 2H | 0 | 0.677 | 0.294442 | 0.641355 |
| 3 | 11_10326 | 2H | 6.5 | 0.383 | 0.414 | 0.700858 |
| 3 | 11_11059 | 2H | 7.1 | 0.044 | 0.90365 | 0.869833 |
| 3 | 11_21377 | 2H | 8.6 | 0.217 | 0.606736 | 0.769383 |
| 3 | 11_20563 | 2H | 9.3 | 0.274 | 0.532108 | 0.753599 |
| 3 | 11_21416 | 2H | 10.1 | 0.146 | 0.688652 | 0.79419 |
| 3 | 12_11119 | 2H | 10.1 | 0.175 | 0.737904 | 0.817571 |
| 3 | 12_30155 | 2H | 10.1 | 0.171 | 0.714496 | 0.80385 |
| 3 | 12_30402 | 2H | 10.1 | 0.132 | 0.674528 | 0.788075 |
| 3 | 12_31497 | 2H | 10.1 | 0.162 | 0.668344 | 0.784005 |
| 3 | 12_30631 | 2H | 15.2 | 0.255 | 0.555904 | 0.759523 |
| 3 | 11_20107 | 2H | 17.9 | 0.114 | 0.76913 | 0.83201 |
| 3 | 11_10943 | 2H | 18.3 | 0.371 | 0.425598 | 0.704312 |
| 3 | 11_11040 | 2H | 19.3 | 0.01 | 0.977237 | 0.875778 |
| 3 | 12_31284 | 2H | 19.5 | 0.073 | 0.845279 | 0.861565 |
| 3 | 11_10180 | 2H | 21.6 | 0.582 | 0.261818 | 0.641355 |
| 3 | 12_10777 | 2H | 21.6 | 0.084 | 0.824138 | 0.861053 |
| 3 | 12_30871 | 2H | 26.6 | 0.092 | 0.809096 | 0.853428 |
| 3 | 12_30872 | 2H | 26.6 | 0.092 | 0.809096 | 0.853428 |
| 3 | 11_21015 | 2H | 27.3 | 0.064 | 0.862979 | 0.866507 |
| 3 | 11_21261 | 2H | 28.4 | 0.137 | 0.827942 | 0.861053 |
| 3 | 11_21265 | 2H | 28.4 | 0.082 | 0.729458 | 0.811435 |
| 3 | 11_21366 | 2H | 28.4 | 0.082 | 0.827942 | 0.861053 |
| 3 | 11_10787 | 2H | 31 | 0.954 | 0.111173 | 0.59212 |
| 3 | 11_10987 | 2H | 31 | 0.92 | 0.120226 | 0.59212 |
| 3 | 11_20864 | 2H | 31.7 | 0.954 | 0.111173 | 0.59212 |
| 3 | 11_21304 | 2H | 33.7 | 0.556 | 0.277971 | 0.641355 |
| 3 | 11_10525 | 2H | 38 | 1.003 | 0.099312 | 0.582372 |
| 3 | 11_11073 | 2H | 38.5 | 0.372 | 0.42462 | 0.704312 |
| 3 | 11_10178 | 2H | 39.1 | 1.066 | 0.085901 | 0.582372 |
| 3 | 11_10399 | 2H | 39.1 | 1.194 | 0.150314 | 0.59212 |
| 3 | 11_10919 | 2H | 39.1 | 0.823 | 0.05035 | 0.582372 |
| 3 | 12_10296 | 2H | 39.1 | 1.066 | 0.383707 | 0.672645 |
| 3 | 12_20326 | 2H | 39.1 | 1.298 | 0.063973 | 0.582372 |
| 3 | 12_30420 | 2H | 39.1 | 0.416 | 0.085901 | 0.582372 |
| 3 | 12_30657 | 2H | 39.1 | 0.416 | 0.383707 | 0.672645 |
| 3 | 11_10837 | 2H | 40.5 | 0.411 | 0.38815 | 0.67683 |
| 3 | 11_21153 | 2H | 40.9 | 0.514 | 0.306196 | 0.641355 |
| 3 | 12_30363 | 2H | 45.5 | 0.845 | 0.142889 | 0.59212 |
| 3 | 11_10498 | 2H | 49.1 | 0.537 | 0.363078 | 0.654711 |
| 3 | 11_11505 | 2H | 49.1 | 0.44 | 0.290402 | 0.641355 |
| 3 | 11_21005 | 2H | 50.5 | 0.074 | 0.843335 | 0.861565 |
| 3 | 11_10234 | 2H | 51.8 | 0.469 | 0.843335 | 0.861565 |
| 3 | 11_10297 | 2H | 51.8 | 0.44 | 0.363078 | 0.654711 |
| 3 | 11_20674 | 2H | 51.8 | 0.074 | 0.339625 | 0.648583 |
| 3 | 12_30604 | 2H | 51.8 | 0.074 | 0.843335 | 0.861565 |
| 3 | 11_10422 | 2H | 52.5 | 0.329 | 0.468813 | 0.717083 |
| 3 | 11_20929 | 2H | 52.5 | 0.329 | 0.468813 | 0.717083 |
| 3 | 11_10733 | 2H | 55 | 0.315 | 0.484172 | 0.727402 |
| 3 | 12_20234 | 2H | 55 | 0.237 | 0.579429 | 0.759523 |
| 3 | 11_20748 | 2H | 56.3 | 0.334 | 0.463447 | 0.717083 |
| 3 | 11_20891 | 2H | 56.3 | 0.334 | 0.564937 | 0.759523 |
| 3 | 12_30251 | 2H | 56.3 | 0.248 | 0.463447 | 0.717083 |
| 3 | 11_10997 | 2H | 57.5 | 0.334 | 0.463447 | 0.717083 |
| 3 | 11_11133 | 2H | 58.2 | 0.056 | 0.812831 | 0.855812 |
| 3 | 11_20500 | 2H | 58.2 | 0.248 | 0.924698 | 0.869833 |
| 3 | 12_10485 | 2H | 58.2 | 0.266 | 0.879023 | 0.866507 |
| 3 | 12_30634 | 2H | 58.2 | 0.09 | 0.542001 | 0.754654 |
| 3 | 12_31288 | 2H | 58.2 | 0.034 | 0.564937 | 0.759523 |
| 3 | 11_20476 | 2H | 58.9 | 0.34 | 0.457088 | 0.717083 |
| 3 | 11_20690 | 2H | 62.8 | 0.079 | 0.833681 | 0.861053 |
| 3 | 12_11278 | 2H | 63.5 | 0.071 | 0.849181 | 0.862041 |
| 3 | 12_30108 | 2H | 63.5 | 0.071 | 0.912011 | 0.869833 |
| 3 | 12_30724 | 2H | 63.5 | 0.04 | 0.849181 | 0.862041 |
| 3 | 12_31252 | 2H | 64.2 | 0.072 | 0.847227 | 0.861565 |
| 3 | 11_21094 | 2H | 65.7 | 0.056 | 0.879023 | 0.866507 |
| 3 | 11_21166 | 2H | 66.8 | 0.113 | 0.770904 | 0.83201 |
| 3 | 11_21110 | 2H | 67.5 | 0.113 | 0.770904 | 0.83201 |
| 3 | 11_21144 | 2H | 69.2 | 0.039 | 0.914113 | 0.869833 |
| 3 | 11_21251 | 2H | 71.1 | 0.369 | 0.695024 | 0.796791 |
| 3 | 12_10719 | 2H | 71.1 | 0.158 | 0.346737 | 0.648879 |
| 3 | 12_31021 | 2H | 71.1 | 0.46 | 0.427563 | 0.704312 |
| 3 | 11_21205 | 2H | 71.6 | 0.362 | 0.43451 | 0.708363 |
| 3 | 11_20667 | 2H | 72.3 | 0.153 | 0.703072 | 0.79892 |
| 3 | 11_20528 | 2H | 73 | 0.153 | 0.703072 | 0.79892 |
| 3 | 12_31383 | 2H | 73.8 | 0.284 | 0.519996 | 0.753599 |
| 3 | 11_20699 | 2H | 78 | 0.148 | 0.711214 | 0.803454 |
| 3 | 11_10619 | 2H | 82.8 | 0.244 | 0.570164 | 0.759523 |
| 3 | 11_10786 | 2H | 82.8 | 0.244 | 0.480839 | 0.726152 |
| 3 | 11_10823 | 2H | 82.8 | 0.244 | 0.570164 | 0.759523 |
| 3 | 11_11214 | 2H | 82.8 | 0.318 | 0.570164 | 0.759523 |
| 3 | 11_21242 | 2H | 82.8 | 0.318 | 0.480839 | 0.726152 |
| 3 | 11_20781 | 2H | 83.8 | 0.578 | 0.264241 | 0.641355 |
| 3 | 12_31205 | 2H | 86.6 | 0.055 | 0.881049 | 0.866507 |
| 3 | 11_11533 | 2H | 87.3 | 0.038 | 0.916221 | 0.869833 |
| 3 | 11_10475 | 2H | 88.7 | 0.343 | 0.616595 | 0.769383 |
| 3 | 11_21037 | 2H | 88.7 | 0.21 | 0.453942 | 0.717083 |
| 3 | 11_21136 | 2H | 88.7 | 0.12 | 0.758578 | 0.824865 |
| 3 | 11_21245 | 2H | 89.3 | 0.281 | 0.5236 | 0.753599 |
| 3 | 12_31424 | 2H | 89.3 | 0.21 | 0.616595 | 0.769383 |
| 3 | 11_21351 | 2H | 90.1 | 0.21 | 0.616595 | 0.769383 |
| 3 | 12_10969 | 2H | 90.1 | 0.21 | 0.616595 | 0.769383 |
| 3 | 11_10214 | 2H | 93.5 | 0.217 | 0.606736 | 0.769383 |
| 3 | 11_20080 | 2H | 95.6 | 0.09 | 0.812831 | 0.855812 |
| 3 | 11_21007 | 2H | 96.2 | 0.083 | 0.826038 | 0.861053 |
| 3 | 11_10138 | 2H | 96.8 | 0.163 | 0.642688 | 0.773384 |
| 3 | 11_11307 | 2H | 96.8 | 0.422 | 0.378443 | 0.668942 |
| 3 | 11_21175 | 2H | 96.8 | 0.192 | 0.244906 | 0.641355 |
| 3 | 12_10649 | 2H | 96.8 | 0.259 | 0.687068 | 0.79419 |
| 3 | 12_30216 | 2H | 96.8 | 0.611 | 0.550808 | 0.758798 |
| 3 | 11_20086 | 2H | 98.6 | 0.928 | 0.118032 | 0.59212 |
| 3 | 11_10398 | 2H | 100.4 | 0.482 | 0.32961 | 0.648583 |
| 3 | 11_10900 | 2H | 101.8 | 0.601 | 0.250611 | 0.641355 |
| 3 | 12_30480 | 2H | 102.5 | 0.29 | 0.512861 | 0.749181 |
| 3 | 11_21340 | 2H | 103.7 | 0.072 | 0.847227 | 0.861565 |
| 3 | 11_10630 | 2H | 105.8 | 0.072 | 0.847227 | 0.861565 |
| 3 | 11_11323 | 2H | 106.5 | 0.285 | 0.509331 | 0.747786 |
| 3 | 12_30049 | 2H | 106.5 | 0.316 | 0.5188 | 0.75312 |
| 3 | 12_30555 | 2H | 106.5 | 0.293 | 0.483059 | 0.727402 |
| 3 | 11_11480 | 2H | 108.6 | 0.276 | 0.529663 | 0.753599 |
| 3 | 11_20064 | 2H | 112.9 | 0.035 | 0.922571 | 0.869833 |
| 3 | 11_10128 | 2H | 113.5 | 0.531 | 0.161065 | 0.593148 |
| 3 | 11_10731 | 2H | 113.5 | 1.567 | 0.601174 | 0.769383 |
| 3 | 11_10988 | 2H | 113.5 | 0.745 | 0.294442 | 0.641355 |
| 3 | 11_10989 | 2H | 113.5 | 0.221 | 0.294442 | 0.641355 |
| 3 | 11_10990 | 2H | 113.5 | 0.531 | 0.179887 | 0.601743 |
| 3 | 11_11043 | 2H | 113.5 | 0.896 | 0.601174 | 0.769383 |
| 3 | 11_11118 | 2H | 113.5 | 0.793 | 0.027102 | 0.51597 |
| 3 | 11_21238 | 2H | 113.5 | 0.221 | 0.127057 | 0.59212 |
| 3 | 12_31402 | 2H | 113.5 | 0.037 | 0.918333 | 0.869833 |
| 3 | 11_10429 | 2H | 115.1 | 1.113 | 0.07709 | 0.582372 |
| 3 | 11_11236 | 2H | 115.8 | 1.044 | 0.090365 | 0.582372 |
| 3 | 11_10538 | 2H | 116.5 | 1.044 | 0.194536 | 0.620385 |
| 3 | 11_10707 | 2H | 116.5 | 1.044 | 0.090365 | 0.582372 |
| 3 | 11_20182 | 2H | 116.5 | 0.711 | 0.032211 | 0.558315 |
| 3 | 12_30459 | 2H | 116.5 | 1.492 | 0.090365 | 0.582372 |
| 3 | 11_10404 | 2H | 117.2 | 0.623 | 0.238232 | 0.641355 |
| 3 | 11_10916 | 2H | 117.9 | 0.932 | 0.11695 | 0.59212 |
| 3 | 12_30598 | 2H | 119.3 | 0.607 | 0.247172 | 0.641355 |
| 3 | 12_31264 | 2H | 119.3 | 0.607 | 0.247172 | 0.641355 |
| 3 | 11_21220 | 2H | 120 | 0.914 | 0.121899 | 0.59212 |
| 3 | 11_20511 | 2H | 120.8 | 0.856 | 0.139316 | 0.59212 |
| 3 | 11_21315 | 2H | 121.5 | 0.128 | 0.744732 | 0.819012 |
| 3 | 12_30152 | 2H | 122.2 | 0.359 | 0.524808 | 0.753599 |
| 3 | 12_30636 | 2H | 122.2 | 0.28 | 0.437522 | 0.708626 |
| 3 | 11_10446 | 2H | 125.5 | 0.488 | 0.325087 | 0.648583 |
| 3 | 11_21370 | 2H | 125.5 | 0.488 | 0.325087 | 0.648583 |
| 3 | 12_31100 | 2H | 125.5 | 0.488 | 0.325087 | 0.648583 |
| 3 | 11_20480 | 2H | 126 | 0.907 | 0.243781 | 0.641355 |
| 3 | 11_21440 | 2H | 126 | 0.613 | 0.12388 | 0.59212 |
| 3 | 11_21406 | 2H | 126.4 | 0.488 | 0.325087 | 0.648583 |
| 3 | 11_11486 | 2H | 127.1 | 0.488 | 0.549541 | 0.758798 |
| 3 | 11_21459 | 2H | 127.1 | 0.26 | 0.325087 | 0.648583 |
| 3 | 11_10109 | 2H | 127.6 | 0.729 | 0.186638 | 0.608234 |
| 3 | 12_20183 | 2H | 127.6 | 0.045 | 0.901571 | 0.869833 |
| 3 | 11_21125 | 2H | 129.3 | 0.304 | 0.496592 | 0.736533 |
| 3 | 12_10164 | 2H | 130 | 0.436 | 0.366438 | 0.655706 |
| 3 | 12_30942 | 2H | 130 | 0.436 | 0.366438 | 0.655706 |
| 3 | 12_31268 | 2H | 130 | 0.304 | 0.496592 | 0.736533 |
| 3 | 11_10376 | 2H | 131.8 | 0.285 | 0.647143 | 0.773384 |
| 3 | 11_20895 | 2H | 131.8 | 0.189 | 0.5188 | 0.75312 |
| 3 | 12_10579 | 2H | 132.5 | 0.306 | 0.494311 | 0.736533 |
| 3 | 11_20715 | 2H | 133.9 | 0.155 | 0.699842 | 0.79759 |
| 3 | 12_30106 | 2H | 133.9 | 0.035 | 0.922571 | 0.869833 |
| 3 | 12_30396 | 2H | 133.9 | 0.011 | 0.97499 | 0.875024 |
| 3 | 11_20590 | 2H | 137.5 | 0.491 | 0.355631 | 0.654711 |
| 3 | 12_30341 | 2H | 137.5 | 0.449 | 0.322849 | 0.648583 |
| 3 | 12_31461 | 2H | 138.9 | 0.442 | 0.36141 | 0.654711 |
| 3 | 11_10551 | 2H | 139.7 | 0.75 | 0.451856 | 0.717083 |
| 3 | 11_10625 | 2H | 139.7 | 0.672 | 0.322849 | 0.648583 |
| 3 | 11_10826 | 2H | 139.7 | 0.491 | 0.177828 | 0.601743 |
| 3 | 11_11262 | 2H | 139.7 | 0.345 | 0.098855 | 0.582372 |
| 3 | 11_20494 | 2H | 139.7 | 0.75 | 0.177828 | 0.601743 |
| 3 | 12_30352 | 2H | 139.7 | 1.005 | 0.177828 | 0.601743 |
| 3 | 12_30914 | 2H | 139.7 | 0.75 | 0.212814 | 0.641355 |
| 3 | 12_31209 | 2H | 139.7 | 0.75 | 0.177828 | 0.601743 |
| 3 | 11_10566 | 2H | 140.3 | 0.75 | 0.177828 | 0.601743 |
| 3 | 11_11023 | 2H | 141.3 | 0.499 | 0.316957 | 0.648583 |
| 3 | 12_10447 | 2H | 141.3 | 0.442 | 0.36141 | 0.654711 |
| 3 | 11_11380 | 2H | 145 | 0.613 | 0.243781 | 0.641355 |
| 3 | 11_20994 | 2H | 147.1 | 0.56 | 0.275423 | 0.641355 |
| 3 | 11_21346 | 2H | 147.1 | 0.015 | 0.966051 | 0.875024 |
| 3 | 11_20293 | 2H | 147.9 | 0.015 | 0.966051 | 0.875024 |
| 3 | 11_21299 | 2H | 149.4 | 0.477 | 0.333426 | 0.648583 |
| 3 | 11_20943 | 2H | 149.6 | 0.803 | 0.157398 | 0.59212 |
| 3 | 11_10791 | 2H | 150.7 | 0.297 | 0.226464 | 0.641355 |
| 3 | 11_21436 | 2H | 150.7 | 1.016 | 0.504661 | 0.744521 |
| 3 | 12_30823 | 2H | 150.7 | 0.645 | 0.096383 | 0.582372 |
| 3 | 11_10072 | 2H | 151.4 | 0.195 | 0.638264 | 0.773384 |
| 3 | 12_31527 | 2H | 151.4 | 0.202 | 0.628058 | 0.773384 |
| 3 | 11_21099 | 2H | 155.3 | 1.146 | 0.07145 | 0.582372 |
| 3 | 11_21453 | 2H | 155.3 | 1.146 | 0.07145 | 0.582372 |
| 3 | 11_20561 | 2H | 156.7 | 1.239 | 0.057677 | 0.582372 |
| 3 | 12_30102 | 2H | 160.3 | 0.188 | 0.648634 | 0.773384 |
| 3 | 11_10044 | 3H | 0 | 0.367 | 0.429536 | 0.705561 |
| 3 | 11_11411 | 3H | 0 | 0.215 | 0.609537 | 0.769383 |
| 3 | 11_20952 | 3H | 0 | 0.05 | 0.891251 | 0.86886 |
| 3 | 11_20159 | 3H | 2.9 | 0.976 | 0.105682 | 0.59212 |
| 3 | 11_11453 | 3H | 6 | 0.525 | 0.298538 | 0.641355 |
| 3 | 11_20252 | 3H | 6 | 0.459 | 0.347536 | 0.648879 |
| 3 | 12_31409 | 3H | 6.7 | 0.459 | 0.347536 | 0.648879 |
| 3 | 11_21398 | 3H | 8.2 | 0.307 | 0.493174 | 0.736164 |
| 3 | 11_21027 | 3H | 8.9 | 0.046 | 0.899498 | 0.869833 |
| 3 | 11_20976 | 3H | 9.6 | 1.512 | 0.171791 | 0.601743 |
| 3 | 12_30818 | 3H | 9.6 | 0.765 | 0.030761 | 0.549592 |
| 3 | 11_10112 | 3H | 10.8 | 1.014 | 0.096828 | 0.582372 |
| 3 | 11_10886 | 3H | 10.8 | 1.307 | 0.049317 | 0.582372 |
| 3 | 11_20595 | 3H | 12.5 | 1.043 | 0.090573 | 0.582372 |
| 3 | 12_30915 | 3H | 13.2 | 0.727 | 0.1875 | 0.608234 |
| 3 | 12_30113 | 3H | 15.6 | 0.882 | 0.13122 | 0.59212 |
| 3 | 11_20742 | 3H | 19.1 | 0.693 | 0.202768 | 0.633011 |
| 3 | 11_20982 | 3H | 22.7 | 0.938 | 0.115345 | 0.59212 |
| 3 | 12_30192 | 3H | 23.4 | 1.013 | 0.097051 | 0.582372 |
| 3 | 11_20552 | 3H | 24.2 | 1.013 | 0.097051 | 0.582372 |
| 3 | 11_20794 | 3H | 26.9 | 0.852 | 0.140605 | 0.59212 |
| 3 | 11_20455 | 3H | 28.4 | 0.852 | 0.140605 | 0.59212 |
| 3 | 12_30284 | 3H | 28.4 | 0.931 | 0.11722 | 0.59212 |
| 3 | 11_10026 | 3H | 32.8 | 1.154 | 0.070146 | 0.582372 |
| 3 | 11_20607 | 3H | 32.8 | 1.309 | 0.194985 | 0.620385 |
| 3 | 12_11414 | 3H | 32.8 | 0.825 | 0.149624 | 0.59212 |
| 3 | 12_30571 | 3H | 32.8 | 0.71 | 0.049091 | 0.582372 |
| 3 | 11_10672 | 3H | 37.2 | 0.883 | 0.130918 | 0.59212 |
| 3 | 12_30925 | 3H | 37.2 | 0.883 | 0.130918 | 0.59212 |
| 3 | 12_10968 | 3H | 38.7 | 0.814 | 0.153462 | 0.59212 |
| 3 | 11_10081 | 3H | 39.5 | 0.883 | 0.130918 | 0.59212 |
| 3 | 11_10710 | 3H | 39.5 | 0.883 | 0.130918 | 0.59212 |
| 3 | 11_10825 | 3H | 39.5 | 0.883 | 0.130918 | 0.59212 |
| 3 | 11_20410 | 3H | 39.5 | 0.883 | 0.130918 | 0.59212 |
| 3 | 12_30953 | 3H | 41 | 0.883 | 0.130918 | 0.59212 |
| 3 | 11_21533 | 3H | 43.2 | 0.585 | 0.260016 | 0.641355 |
| 3 | 11_11002 | 3H | 44 | 0.604 | 0.248886 | 0.641355 |
| 3 | 12_30913 | 3H | 44.8 | 0.789 | 0.162555 | 0.593148 |
| 3 | 12_31475 | 3H | 45.5 | 0.853 | 0.140281 | 0.59212 |
| 3 | 11_10601 | 3H | 46.3 | 0.931 | 0.11722 | 0.59212 |
| 3 | 12_30064 | 3H | 46.3 | 0.931 | 0.11722 | 0.59212 |
| 3 | 12_30609 | 3H | 48.6 | 0.931 | 0.11722 | 0.59212 |
| 3 | 12_31122 | 3H | 48.6 | 0.931 | 0.11722 | 0.59212 |
| 3 | 11_21197 | 3H | 51.7 | 0.902 | 0.125314 | 0.59212 |
| 3 | 11_11086 | 3H | 53.3 | 0.512 | 0.30761 | 0.641355 |
| 3 | 12_30618 | 3H | 53.3 | 0.512 | 0.30761 | 0.641355 |
| 3 | 11_10137 | 3H | 54.4 | 0.512 | 0.30761 | 0.641355 |
| 3 | 11_10328 | 3H | 54.4 | 0.512 | 0.413048 | 0.700267 |
| 3 | 11_11099 | 3H | 54.4 | 0.384 | 0.30761 | 0.641355 |
| 3 | 11_11501 | 3H | 54.4 | 0.512 | 0.30761 | 0.641355 |
| 3 | 11_20970 | 3H | 54.4 | 0.512 | 0.30761 | 0.641355 |
| 3 | 12_30318 | 3H | 54.4 | 0.512 | 0.30761 | 0.641355 |
| 3 | 12_31012 | 3H | 55.6 | 0.512 | 0.30761 | 0.641355 |
| 3 | 12_31502 | 3H | 55.6 | 0.512 | 0.30761 | 0.641355 |
| 3 | 11_10456 | 3H | 56.4 | 0.512 | 0.314775 | 0.648493 |
| 3 | 11_10620 | 3H | 56.4 | 0.502 | 0.30761 | 0.641355 |
| 3 | 11_10925 | 3H | 56.4 | 0.512 | 0.30761 | 0.641355 |
| 3 | 11_10926 | 3H | 56.4 | 0.512 | 0.423643 | 0.704312 |
| 3 | 11_10966 | 3H | 56.4 | 0.512 | 0.30761 | 0.641355 |
| 3 | 11_11124 | 3H | 56.4 | 0.548 | 0.314775 | 0.648493 |
| 3 | 11_11125 | 3H | 56.4 | 0.373 | 0.30761 | 0.641355 |
| 3 | 11_11283 | 3H | 56.4 | 0.512 | 0.30761 | 0.641355 |
| 3 | 11_11337 | 3H | 56.4 | 0.512 | 0.413048 | 0.700267 |
| 3 | 11_11530 | 3H | 56.4 | 0.502 | 0.30761 | 0.641355 |
| 3 | 11_20002 | 3H | 56.4 | 0.512 | 0.34435 | 0.648583 |
| 3 | 11_20288 | 3H | 56.4 | 0.512 | 0.30761 | 0.641355 |
| 3 | 11_20333 | 3H | 56.4 | 0.512 | 0.30761 | 0.641355 |
| 3 | 11_20486 | 3H | 56.4 | 0.384 | 0.30761 | 0.641355 |
| 3 | 11_20583 | 3H | 56.4 | 0.512 | 0.30761 | 0.641355 |
| 3 | 11_20801 | 3H | 56.4 | 0.463 | 0.30761 | 0.641355 |
| 3 | 11_20856 | 3H | 56.4 | 0.512 | 0.283139 | 0.641355 |
| 3 | 11_21147 | 3H | 56.4 | 0.512 | 0.30761 | 0.641355 |
| 3 | 11_21435 | 3H | 56.4 | 0.512 | 0.30761 | 0.641355 |
| 3 | 11_21472 | 3H | 56.4 | 0.384 | 0.413048 | 0.700267 |
| 3 | 12_31214 | 3H | 56.4 | 0.512 | 0.30761 | 0.641355 |
| 3 | 12_31281 | 3H | 56.4 | 0.331 | 0.466659 | 0.717083 |
| 3 | 12_31368 | 3H | 56.4 | 0.331 | 0.466659 | 0.717083 |
| 3 | 11_10225 | 3H | 58 | 0.331 | 0.466659 | 0.717083 |
| 3 | 11_11401 | 3H | 58 | 0.331 | 0.466659 | 0.717083 |
| 3 | 11_11016 | 3H | 58.6 | 0.331 | 0.530884 | 0.753599 |
| 3 | 11_20276 | 3H | 58.6 | 0.275 | 0.466659 | 0.717083 |
| 3 | 12_31011 | 3H | 58.6 | 0.331 | 0.466659 | 0.717083 |
| 3 | 12_31393 | 3H | 58.6 | 0.331 | 0.466659 | 0.717083 |
| 3 | 11_10653 | 3H | 59.9 | 0.629 | 0.466659 | 0.717083 |
| 3 | 11_21511 | 3H | 59.9 | 0.331 | 0.234963 | 0.641355 |
| 3 | 11_10728 | 3H | 63 | 0.492 | 0.322107 | 0.648583 |
| 3 | 11_10281 | 3H | 64.2 | 0.492 | 0.322107 | 0.648583 |
| 3 | 11_11191 | 3H | 64.2 | 0.146 | 0.714496 | 0.80385 |
| 3 | 11_21502 | 3H | 65.5 | 0.176 | 0.666807 | 0.783785 |
| 3 | 11_20931 | 3H | 68.3 | 0.053 | 0.885116 | 0.866507 |
| 3 | 12_30616 | 3H | 68.3 | 0.053 | 0.933254 | 0.869833 |
| 3 | 12_30788 | 3H | 68.3 | 0.03 | 0.885116 | 0.866507 |
| 3 | 12_31242 | 3H | 69.6 | 0.113 | 0.770904 | 0.83201 |
| 3 | 11_11241 | 3H | 70.7 | 0.241 | 0.651628 | 0.774568 |
| 3 | 11_20273 | 3H | 70.7 | 0.186 | 0.574117 | 0.759523 |
| 3 | 11_20877 | 3H | 70.7 | 0.241 | 0.574117 | 0.759523 |
| 3 | 12_31323 | 3H | 70.7 | 0.241 | 0.574117 | 0.759523 |
| 3 | 12_30754 | 3H | 71.5 | 0.186 | 0.651628 | 0.774568 |
| 3 | 12_31529 | 3H | 72.3 | 0.514 | 0.306196 | 0.641355 |
| 3 | 12_31356 | 3H | 73.5 | 0.548 | 0.283139 | 0.641355 |
| 3 | 11_10276 | 3H | 74.8 | 0.514 | 0.306196 | 0.641355 |
| 3 | 12_30399 | 3H | 74.8 | 0.514 | 0.306196 | 0.641355 |
| 3 | 11_20566 | 3H | 75.5 | 0.214 | 0.610942 | 0.769383 |
| 3 | 11_10683 | 3H | 76.2 | 1.195 | 0.063826 | 0.582372 |
| 3 | 11_20695 | 3H | 76.2 | 1.195 | 0.063826 | 0.582372 |
| 3 | 11_20778 | 3H | 76.2 | 1.195 | 0.063826 | 0.582372 |
| 3 | 12_10609 | 3H | 76.2 | 1.195 | 0.063826 | 0.582372 |
| 3 | 12_30743 | 3H | 76.2 | 1.195 | 0.063826 | 0.582372 |
| 3 | 12_31346 | 3H | 77 | 0.675 | 0.211349 | 0.641355 |
| 3 | 11_10047 | 3H | 78.5 | 1.607 | 0.276694 | 0.641355 |
| 3 | 11_20362 | 3H | 78.5 | 0.558 | 0.024717 | 0.503592 |
| 3 | 11_20597 | 3H | 78.5 | 1.607 | 0.024717 | 0.503592 |
| 3 | 11_20115 | 3H | 80.9 | 0.822 | 0.86896 | 0.866507 |
| 3 | 12_30170 | 3H | 80.9 | 0.061 | 0.150661 | 0.59212 |
| 3 | 11_20093 | 3H | 81.7 | 0.822 | 0.150661 | 0.59212 |
| 3 | 11_21358 | 3H | 81.7 | 0.822 | 0.150661 | 0.59212 |
| 3 | 12_30677 | 3H | 81.7 | 0.822 | 0.150661 | 0.59212 |
| 3 | 12_31262 | 3H | 81.7 | 0.154 | 0.701455 | 0.798645 |
| 3 | 12_30278 | 3H | 83.2 | 0.46 | 0.346737 | 0.648879 |
| 3 | 12_11517 | 3H | 83.7 | 0.924 | 0.119124 | 0.59212 |
| 3 | 11_20063 | 3H | 86 | 1.778 | 0.190985 | 0.6161 |
| 3 | 12_11138 | 3H | 86 | 0.719 | 0.016672 | 0.43027 |
| 3 | 11_10444 | 3H | 87.2 | 1.82 | 0.015136 | 0.42511 |
| 3 | 11_10628 | 3H | 87.2 | 1.793 | 0.139637 | 0.59212 |
| 3 | 12_31299 | 3H | 87.2 | 0.855 | 0.016106 | 0.42511 |
| 3 | 11_20136 | 3H | 88.8 | 1.248 | 0.056494 | 0.582372 |
| 3 | 12_10134 | 3H | 89.3 | 0.312 | 0.6223 | 0.771284 |
| 3 | 12_30663 | 3H | 89.3 | 0.206 | 0.078524 | 0.582372 |
| 3 | 12_31018 | 3H | 89.3 | 1.105 | 0.487529 | 0.730554 |
| 3 | 11_20659 | 3H | 91.2 | 1.105 | 0.078524 | 0.582372 |
| 3 | 11_10253 | 3H | 91.9 | 0.462 | 0.345144 | 0.648583 |
| 3 | 12_10583 | 3H | 91.9 | 0.129 | 0.743019 | 0.818679 |
| 3 | 11_10747 | 3H | 93.4 | 1.12 | 0.075858 | 0.582372 |
| 3 | 11_11021 | 3H | 93.4 | 0.462 | 0.345144 | 0.648583 |
| 3 | 12_30250 | 3H | 96.2 | 0.178 | 0.663743 | 0.783356 |
| 3 | 12_30090 | 3H | 97.7 | 0.131 | 0.739605 | 0.817571 |
| 3 | 11_20130 | 3H | 98.5 | 0.236 | 0.580764 | 0.759523 |
| 3 | 11_20628 | 3H | 98.5 | 0.236 | 0.729458 | 0.811435 |
| 3 | 11_20999 | 3H | 98.5 | 0.137 | 0.580764 | 0.759523 |
| 3 | 11_21438 | 3H | 98.5 | 0.236 | 0.580764 | 0.759523 |
| 3 | 11_20626 | 3H | 99.1 | 0.033 | 0.92683 | 0.869833 |
| 3 | 11_10515 | 3H | 99.9 | 1.612 | 0.024434 | 0.503592 |
| 3 | 11_10184 | 3H | 100.7 | 0.374 | 0.422669 | 0.704312 |
| 3 | 11_21083 | 3H | 101.4 | 0.287 | 0.516416 | 0.75312 |
| 3 | 11_21381 | 3H | 102.2 | 0.216 | 0.608135 | 0.769383 |
| 3 | 11_21517 | 3H | 103 | 0.032 | 0.928966 | 0.869833 |
| 3 | 11_21495 | 3H | 104.5 | 0.456 | 0.349945 | 0.650698 |
| 3 | 12_10344 | 3H | 104.5 | 0.242 | 0.572796 | 0.759523 |
| 3 | 12_10662 | 3H | 104.5 | 0.242 | 0.94189 | 0.8729 |
| 3 | 12_30342 | 3H | 104.5 | 0.026 | 0.572796 | 0.759523 |
| 3 | 11_21493 | 3H | 105.3 | 0.216 | 0.608135 | 0.769383 |
| 3 | 12_30119 | 3H | 106.8 | 0.216 | 0.608135 | 0.769383 |
| 3 | 11_20009 | 3H | 107.6 | 0.173 | 0.671429 | 0.786829 |
| 3 | 11_20023 | 3H | 111.4 | 0.759 | 0.174181 | 0.601743 |
| 3 | 11_21161 | 3H | 111.4 | 0.759 | 0.174181 | 0.601743 |
| 3 | 11_21212 | 3H | 111.4 | 1.325 | 0.174181 | 0.601743 |
| 3 | 12_30423 | 3H | 111.4 | 0.759 | 0.047315 | 0.582372 |
| 3 | 11_10312 | 3H | 114 | 0.254 | 0.594292 | 0.766852 |
| 3 | 11_10753 | 3H | 114 | 0.226 | 0.077804 | 0.582372 |
| 3 | 11_11503 | 3H | 114 | 1.065 | 0.557186 | 0.759523 |
| 3 | 11_20168 | 3H | 114 | 1.109 | 0.077804 | 0.582372 |
| 3 | 11_20523 | 3H | 114 | 0.349 | 0.447713 | 0.717083 |
| 3 | 11_21277 | 3H | 114 | 0.254 | 0.086099 | 0.582372 |
| 3 | 12_10100 | 3H | 114 | 1.109 | 0.077804 | 0.582372 |
| 3 | 12_30276 | 3H | 114 | 1.109 | 0.557186 | 0.759523 |
| 3 | 12_30375 | 3H | 114.8 | 0.14 | 0.724436 | 0.808171 |
| 3 | 12_31329 | 3H | 115.5 | 0.254 | 0.557186 | 0.759523 |
| 3 | 12_30927 | 3H | 117.1 | 0.309 | 0.490908 | 0.73467 |
| 3 | 12_31220 | 3H | 120.6 | 0.087 | 0.818465 | 0.860185 |
| 3 | 11_10918 | 3H | 123.7 | 0.66 | 0.218776 | 0.641355 |
| 3 | 11_21405 | 3H | 123.7 | 0.66 | 0.218776 | 0.641355 |
| 3 | 12_30274 | 3H | 124.8 | 0.539 | 0.289068 | 0.641355 |
| 3 | 11_10754 | 3H | 126.3 | 0.52 | 0.429536 | 0.705561 |
| 3 | 11_10821 | 3H | 126.3 | 0.367 | 0.301995 | 0.641355 |
| 3 | 11_11172 | 3H | 126.3 | 0.342 | 0.454988 | 0.717083 |
| 3 | 12_31525 | 3H | 126.3 | 0.342 | 0.454988 | 0.717083 |
| 3 | 11_10280 | 3H | 130.2 | 0.267 | 0.540754 | 0.754654 |
| 3 | 11_20343 | 3H | 130.2 | 0.267 | 0.540754 | 0.754654 |
| 3 | 11_11141 | 3H | 130.8 | 0.267 | 0.540754 | 0.754654 |
| 3 | 12_10505 | 3H | 130.8 | 0.267 | 0.540754 | 0.754654 |
| 3 | 11_21266 | 3H | 148.9 | 0.049 | 0.893306 | 0.86886 |
| 3 | 11_21272 | 3H | 150.4 | 0.05 | 0.891251 | 0.86886 |
| 3 | 11_10646 | 3H | 162.2 | 0.504 | 0.419759 | 0.704312 |
| 3 | 11_10702 | 3H | 162.2 | 0.38 | 0.416869 | 0.703664 |
| 3 | 12_30271 | 3H | 162.2 | 0.377 | 0.313329 | 0.648493 |
| 3 | 12_30767 | 3H | 162.2 | 0.377 | 0.419759 | 0.704312 |
| 3 | 11_20605 | 3H | 166.2 | 0.539 | 0.289068 | 0.641355 |
| 3 | 11_10681 | 3H | 167.8 | 0.215 | 0.609537 | 0.769383 |
| 3 | 11_11410 | 3H | 167.8 | 0.215 | 0.97949 | 0.875778 |
| 3 | 12_31388 | 3H | 167.8 | 0.009 | 0.609537 | 0.769383 |
| 3 | 11_10694 | 3H | 168.4 | 0.424 | 0.83946 | 0.861565 |
| 3 | 11_20057 | 3H | 168.4 | 0.192 | 0.642688 | 0.773384 |
| 3 | 11_21267 | 3H | 168.4 | 0.106 | 0.78343 | 0.835461 |
| 3 | 12_10014 | 3H | 168.4 | 0.076 | 0.376704 | 0.667902 |
| 3 | 12_30736 | 3H | 168.4 | 0.073 | 0.845279 | 0.861565 |
| 3 | 11_11516 | 3H | 169.3 | 0.65 | 0.223872 | 0.641355 |
| 3 | 12_30055 | 3H | 172.4 | 0.364 | 0.432514 | 0.70745 |
| 3 | 11_10343 | 3H | 173.2 | 1.012 | 0.097275 | 0.582372 |
| 3 | 12_30135 | 3H | 173.2 | 0.396 | 0.401791 | 0.6923 |
| 3 | 11_10028 | 4H | 0 | 0.991 | 0.427563 | 0.704312 |
| 3 | 11_10247 | 4H | 0 | 0.533 | 0.966051 | 0.875024 |
| 3 | 11_10379 | 4H | 0 | 0.015 | 0.157036 | 0.59212 |
| 3 | 11_10509 | 4H | 0 | 0.577 | 0.26485 | 0.641355 |
| 3 | 11_10751 | 4H | 0 | 0.804 | 0.174181 | 0.601743 |
| 3 | 11_20668 | 4H | 0 | 0.369 | 0.102094 | 0.582372 |
| 3 | 11_20740 | 4H | 0 | 0.759 | 0.293089 | 0.641355 |
| 3 | 11_10409 | 4H | 3.7 | 0.462 | 0.345144 | 0.648583 |
| 3 | 11_21228 | 4H | 3.7 | 0.462 | 0.345144 | 0.648583 |
| 3 | 11_11345 | 4H | 5.5 | 0.476 | 0.334195 | 0.648583 |
| 3 | 12_30540 | 4H | 15.8 | 1.065 | 0.086099 | 0.582372 |
| 3 | 11_10223 | 4H | 20.1 | 0.162 | 0.688652 | 0.79419 |
| 3 | 11_10221 | 4H | 21.6 | 0.038 | 0.916221 | 0.869833 |
| 3 | 12_10395 | 4H | 24.6 | 0.275 | 0.530884 | 0.753599 |
| 3 | 11_20302 | 4H | 26.2 | 0.155 | 0.699842 | 0.79759 |
| 3 | 11_10031 | 4H | 28.4 | 1.708 | 0.019588 | 0.454971 |
| 3 | 12_31164 | 4H | 32.5 | 0.166 | 0.682339 | 0.792659 |
| 3 | 11_21122 | 4H | 33.4 | 0.679 | 0.209411 | 0.641355 |
| 3 | 11_21397 | 4H | 33.4 | 0.515 | 0.305492 | 0.641355 |
| 3 | 11_21389 | 4H | 36.4 | 2.293 | 0.635331 | 0.773384 |
| 3 | 12_10860 | 4H | 36.4 | 0.197 | 0.005093 | 0.257174 |
| 3 | 11_20012 | 4H | 39.8 | 1.307 | 0.049317 | 0.582372 |
| 3 | 11_20114 | 4H | 40.4 | 1.314 | 0.048529 | 0.582372 |
| 3 | 11_20180 | 4H | 40.4 | 0.994 | 0.101391 | 0.582372 |
| 3 | 12_10063 | 4H | 40.4 | 0.877 | 0.545758 | 0.756328 |
| 3 | 12_10371 | 4H | 40.4 | 0.263 | 0.132739 | 0.59212 |
| 3 | 12_20240 | 4H | 40.4 | 0.877 | 0.132739 | 0.59212 |
| 3 | 11_11180 | 4H | 41 | 1.002 | 0.099541 | 0.582372 |
| 3 | 11_10048 | 4H | 42.5 | 0.696 | 0.201372 | 0.630348 |
| 3 | 11_10668 | 4H | 44.9 | 0.222 | 0.599791 | 0.769383 |
| 3 | 11_11405 | 4H | 47.6 | 0.804 | 0.157036 | 0.59212 |
| 3 | 11_10093 | 4H | 48.5 | 0.857 | 0.774462 | 0.83201 |
| 3 | 11_10577 | 4H | 48.5 | 0.111 | 0.09528 | 0.582372 |
| 3 | 11_10756 | 4H | 48.5 | 1.021 | 0.09528 | 0.582372 |
| 3 | 11_10942 | 4H | 48.5 | 0.857 | 0.845279 | 0.861565 |
| 3 | 11_20269 | 4H | 48.5 | 1.021 | 0.309742 | 0.643491 |
| 3 | 11_20782 | 4H | 48.5 | 0.517 | 0.250035 | 0.641355 |
| 3 | 11_20853 | 4H | 48.5 | 0.928 | 0.304089 | 0.641355 |
| 3 | 11_21071 | 4H | 48.5 | 0.073 | 0.118032 | 0.59212 |
| 3 | 11_21073 | 4H | 48.5 | 1.021 | 0.09528 | 0.582372 |
| 3 | 12_30331 | 4H | 48.5 | 0.509 | 0.138995 | 0.59212 |
| 3 | 12_31382 | 4H | 48.5 | 0.602 | 0.138995 | 0.59212 |
| 3 | 12_30777 | 4H | 49.5 | 1.026 | 0.094189 | 0.582372 |
| 3 | 11_20289 | 4H | 50.4 | 0.286 | 0.517607 | 0.75312 |
| 3 | 12_30450 | 4H | 50.4 | 0.286 | 0.157036 | 0.59212 |
| 3 | 12_30684 | 4H | 50.4 | 0.804 | 0.517607 | 0.75312 |
| 3 | 12_30866 | 4H | 50.4 | 0.804 | 0.157036 | 0.59212 |
| 3 | 11_10411 | 4H | 51.3 | 0.804 | 0.157036 | 0.59212 |
| 3 | 11_10480 | 4H | 51.3 | 0.804 | 0.157036 | 0.59212 |
| 3 | 11_11042 | 4H | 51.3 | 0.62 | 0.239883 | 0.641355 |
| 3 | 11_10946 | 4H | 52.8 | 0.663 | 0.21727 | 0.641355 |
| 3 | 11_11114 | 4H | 54.2 | 0.578 | 0.264241 | 0.641355 |
| 3 | 11_11244 | 4H | 55 | 0.635 | 0.23174 | 0.641355 |
| 3 | 11_10046 | 4H | 55.6 | 1.333 | 0.046452 | 0.582372 |
| 3 | 11_10527 | 4H | 55.6 | 0.392 | 0.530884 | 0.753599 |
| 3 | 11_10568 | 4H | 55.6 | 0.275 | 0.046452 | 0.582372 |
| 3 | 11_20020 | 4H | 55.6 | 1.333 | 0.08995 | 0.582372 |
| 3 | 11_20412 | 4H | 55.6 | 0.529 | 0.530884 | 0.753599 |
| 3 | 11_21481 | 4H | 55.6 | 0.275 | 0.405509 | 0.69356 |
| 3 | 12_10426 | 4H | 55.6 | 0.362 | 0.43451 | 0.708363 |
| 3 | 12_30839 | 4H | 55.6 | 0.392 | 0.405509 | 0.69356 |
| 3 | 12_30995 | 4H | 55.6 | 1.046 | 0.295801 | 0.641355 |
| 3 | 12_31462 | 4H | 55.6 | 0.275 | 0.530884 | 0.753599 |
| 3 | 11_20361 | 4H | 59.4 | 0.346 | 0.450817 | 0.717083 |
| 3 | 11_21191 | 4H | 61 | 0.577 | 0.26485 | 0.641355 |
| 3 | 12_30237 | 4H | 61 | 0.577 | 0.26485 | 0.641355 |
| 3 | 11_21087 | 4H | 62.1 | 0.039 | 0.914113 | 0.869833 |
| 3 | 11_20453 | 4H | 62.8 | 0.039 | 0.914113 | 0.869833 |
| 3 | 11_21296 | 4H | 62.8 | 0.039 | 0.914113 | 0.869833 |
| 3 | 11_20820 | 4H | 63.6 | 0.241 | 0.574117 | 0.759523 |
| 3 | 11_20723 | 4H | 64.3 | 0.241 | 0.574117 | 0.759523 |
| 3 | 11_10639 | 4H | 65 | 0.934 | 0.116413 | 0.59212 |
| 3 | 11_11431 | 4H | 65 | 0.934 | 0.116413 | 0.59212 |
| 3 | 11_20062 | 4H | 65 | 0.934 | 0.574117 | 0.759523 |
| 3 | 11_20924 | 4H | 65 | 0.241 | 0.116413 | 0.59212 |
| 3 | 11_10010 | 4H | 66 | 0.468 | 0.340408 | 0.648583 |
| 3 | 12_30755 | 4H | 66 | 0.426 | 0.340408 | 0.648583 |
| 3 | 12_30904 | 4H | 66 | 0.468 | 0.340408 | 0.648583 |
| 3 | 12_30905 | 4H | 66 | 0.771 | 0.374973 | 0.66585 |
| 3 | 12_30906 | 4H | 66 | 0.771 | 0.340408 | 0.648583 |
| 3 | 12_31385 | 4H | 66 | 0.468 | 0.169434 | 0.601743 |
| 3 | 12_31493 | 4H | 66 | 0.468 | 0.169434 | 0.601743 |
| 3 | 11_10606 | 4H | 67.5 | 0.934 | 0.116413 | 0.59212 |
| 3 | 11_20072 | 4H | 67.5 | 0.08 | 0.831764 | 0.861053 |
| 3 | 11_10627 | 4H | 68.2 | 1.339 | 0.045814 | 0.582372 |
| 3 | 11_20580 | 4H | 68.2 | 1.013 | 0.097051 | 0.582372 |
| 3 | 12_31536 | 4H | 68.2 | 1.339 | 0.045814 | 0.582372 |
| 3 | 11_11513 | 4H | 69.5 | 0.267 | 0.540754 | 0.754654 |
| 3 | 11_10467 | 4H | 72.1 | 0.082 | 0.827942 | 0.861053 |
| 3 | 12_31362 | 4H | 73.6 | 0.904 | 0.124738 | 0.59212 |
| 3 | 11_10309 | 4H | 76 | 0.223 | 0.944061 | 0.8729 |
| 3 | 12_20143 | 4H | 76 | 0.025 | 0.598412 | 0.769383 |
| 3 | 11_21332 | 4H | 77.3 | 0.027 | 0.939723 | 0.871665 |
| 3 | 11_10523 | 4H | 78.8 | 0.002 | 0.995405 | 0.875778 |
| 3 | 12_31148 | 4H | 78.8 | 0.002 | 0.995405 | 0.875778 |
| 3 | 11_11500 | 4H | 79.6 | 0.159 | 0.693426 | 0.796791 |
| 3 | 11_10724 | 4H | 82.4 | 0.291 | 0.511682 | 0.749181 |
| 3 | 12_31246 | 4H | 83.5 | 0.291 | 0.511682 | 0.749181 |
| 3 | 11_10723 | 4H | 84.3 | 0.319 | 0.479733 | 0.726152 |
| 3 | 12_30390 | 4H | 84.3 | 0.193 | 0.64121 | 0.773384 |
| 3 | 11_11398 | 4H | 87.5 | 0.739 | 0.18239 | 0.601743 |
| 3 | 11_20358 | 4H | 88.2 | 0.151 | 0.706318 | 0.80104 |
| 3 | 11_10588 | 4H | 89.4 | 0.4 | 0.398107 | 0.687994 |
| 3 | 11_20384 | 4H | 91.8 | 0.127 | 0.746449 | 0.820123 |
| 3 | 12_30554 | 4H | 96.6 | 0.877 | 0.132739 | 0.59212 |
| 3 | 11_20762 | 4H | 98.5 | 0.565 | 0.27227 | 0.641355 |
| 3 | 12_10666 | 4H | 98.5 | 0.343 | 0.453942 | 0.717083 |
| 3 | 11_20119 | 4H | 99.3 | 0.133 | 0.736207 | 0.816596 |
| 3 | 11_10614 | 4H | 100.7 | 0.438 | 0.364754 | 0.654711 |
| 3 | 11_11470 | 4H | 100.7 | 0.195 | 0.97499 | 0.875024 |
| 3 | 12_30987 | 4H | 100.7 | 0.011 | 0.638264 | 0.773384 |
| 3 | 12_30988 | 4H | 100.7 | 0.195 | 0.638264 | 0.773384 |
| 3 | 11_20454 | 4H | 101.6 | 0.248 | 0.564937 | 0.759523 |
| 3 | 11_20515 | 4H | 101.6 | 0.238 | 0.578096 | 0.759523 |
| 3 | 11_10510 | 4H | 102.4 | 1.001 | 0.09977 | 0.582372 |
| 3 | 11_10334 | 4H | 103.1 | 0.824 | 0.162181 | 0.593148 |
| 3 | 11_21111 | 4H | 103.1 | 0.79 | 0.149969 | 0.59212 |
| 3 | 12_30990 | 4H | 103.1 | 0.55 | 0.629506 | 0.773384 |
| 3 | 12_31139 | 4H | 103.1 | 0.201 | 0.281838 | 0.641355 |
| 3 | 11_20974 | 4H | 106 | 1.571 | 0.026853 | 0.51597 |
| 3 | 12_30385 | 4H | 107.9 | 0.093 | 0.807235 | 0.853428 |
| 3 | 12_31138 | 4H | 111.1 | 0.486 | 0.326588 | 0.648583 |
| 3 | 11_21130 | 4H | 116.8 | 0.02 | 0.954993 | 0.875024 |
| 3 | 11_10610 | 4H | 119.1 | 0.522 | 0.300608 | 0.641355 |
| 3 | 11_20272 | 4H | 119.1 | 0.522 | 0.526017 | 0.753599 |
| 3 | 12_30476 | 4H | 119.1 | 0.279 | 0.300608 | 0.641355 |
| 3 | 12_30873 | 4H | 119.1 | 0.245 | 0.568853 | 0.759523 |
| 3 | 11_10387 | 4H | 119.8 | 0.664 | 0.21677 | 0.641355 |
| 3 | 12_30006 | 4H | 119.8 | 0.209 | 0.618016 | 0.769383 |
| 3 | 12_30239 | 4H | 119.8 | 0.079 | 0.833681 | 0.861053 |
| 3 | 12_31422 | 4H | 120.6 | 0.664 | 0.21677 | 0.641355 |
| 3 | 11_11186 | 4H | 121.8 | 0.36 | 0.436516 | 0.708626 |
| 3 | 11_11019 | 4H | 123.3 | 2.957 | 0.001104 | 0.211595 |
| 3 | 11_20013 | 4H | 123.3 | 2.957 | 0.052 | 0.582372 |
| 3 | 11_20089 | 4H | 123.3 | 2.957 | 0.052 | 0.582372 |
| 3 | 12_30824 | 4H | 123.3 | 1.284 | 0.001104 | 0.211595 |
| 3 | 12_30825 | 4H | 123.3 | 1.284 | 0.001104 | 0.211595 |
| 3 | 11_10405 | 5H | 0 | 0.072 | 0.933254 | 0.869833 |
| 3 | 11_11361 | 5H | 0 | 0.139 | 0.335738 | 0.648583 |
| 3 | 11_11448 | 5H | 0 | 0.19 | 0.726106 | 0.809257 |
| 3 | 11_20386 | 5H | 0 | 0.474 | 0.168267 | 0.601743 |
| 3 | 11_20644 | 5H | 0 | 0.774 | 0.645654 | 0.773384 |
| 3 | 11_21244 | 5H | 0 | 0.03 | 0.125026 | 0.59212 |
| 3 | 11_21514 | 5H | 0 | 0.903 | 0.847227 | 0.861565 |
| 3 | 11_20226 | 5H | 2.1 | 0.228 | 0.591562 | 0.765029 |
| 3 | 11_20553 | 5H | 2.8 | 0.32 | 0.47863 | 0.725647 |
| 3 | 12_30543 | 5H | 2.8 | 0.024 | 0.946237 | 0.873452 |
| 3 | 12_31023 | 5H | 5 | 0.396 | 0.401791 | 0.6923 |
| 3 | 12_30591 | 5H | 5.7 | 0.234 | 0.98628 | 0.875778 |
| 3 | 12_30979 | 5H | 5.7 | 0.006 | 0.583445 | 0.761314 |
| 3 | 11_20206 | 5H | 6.4 | 0.414 | 0.385478 | 0.673182 |
| 3 | 11_11381 | 5H | 7.5 | 0.407 | 0.391742 | 0.681048 |
| 3 | 11_20533 | 5H | 17.4 | 0.101 | 0.792501 | 0.841272 |
| 3 | 12_31094 | 5H | 18.1 | 0.101 | 0.792501 | 0.841272 |
| 3 | 11_20010 | 5H | 18.7 | 0.297 | 0.504661 | 0.744521 |
| 3 | 11_10695 | 5H | 25.2 | 0.24 | 0.57544 | 0.759523 |
| 3 | 11_20873 | 5H | 26.3 | 0.642 | 0.58479 | 0.762213 |
| 3 | 11_21065 | 5H | 26.3 | 0.233 | 0.228034 | 0.641355 |
| 3 | 12_30167 | 5H | 26.3 | 0.642 | 0.228034 | 0.641355 |
| 3 | 11_10974 | 5H | 27 | 0.236 | 0.606736 | 0.769383 |
| 3 | 11_21426 | 5H | 27 | 0.217 | 0.580764 | 0.759523 |
| 3 | 11_21324 | 5H | 31 | 0.717 | 0.191867 | 0.61723 |
| 3 | 12_10530 | 5H | 33.1 | 0.188 | 0.648634 | 0.773384 |
| 3 | 11_10688 | 5H | 34.2 | 0.799 | 0.158855 | 0.59212 |
| 3 | 12_10499 | 5H | 34.2 | 0.204 | 0.625173 | 0.772371 |
| 3 | 11_10580 | 5H | 35.7 | 0.19 | 0.645654 | 0.773384 |
| 3 | 11_20501 | 5H | 59.4 | 2.509 | 0.003097 | 0.211595 |
| 3 | 12_30111 | 5H | 59.4 | 2.509 | 0.125026 | 0.59212 |
| 3 | 12_30700 | 5H | 59.4 | 1.884 | 0.013062 | 0.42511 |
| 3 | 12_31340 | 5H | 59.4 | 0.903 | 0.003097 | 0.211595 |
| 3 | 11_11159 | 5H | 60.7 | 2.509 | 0.003097 | 0.211595 |
| 3 | 11_11221 | 5H | 60.7 | 2.509 | 0.015417 | 0.42511 |
| 3 | 11_21200 | 5H | 60.7 | 2.509 | 0.015417 | 0.42511 |
| 3 | 11_21508 | 5H | 60.7 | 2.509 | 0.534564 | 0.754654 |
| 3 | 12_11385 | 5H | 60.7 | 1.812 | 0.003097 | 0.211595 |
| 3 | 12_30011 | 5H | 60.7 | 1.812 | 0.003097 | 0.211595 |
| 3 | 12_31033 | 5H | 60.7 | 0.272 | 0.003097 | 0.211595 |
| 3 | 12_31280 | 5H | 60.7 | 2.509 | 0.003097 | 0.211595 |
| 3 | 11_20265 | 5H | 62.1 | 1.133 | 0.073621 | 0.582372 |
| 3 | 12_30745 | 5H | 64 | 0.503 | 0.314051 | 0.648493 |
| 3 | 11_20713 | 5H | 65.5 | 1.328 | 0.046989 | 0.582372 |
| 3 | 11_21275 | 5H | 67.5 | 1.239 | 0.057677 | 0.582372 |
| 3 | 11_21121 | 5H | 68.3 | 0.472 | 0.337287 | 0.648583 |
| 3 | 11_21239 | 5H | 69.3 | 0.073 | 0.845279 | 0.861565 |
| 3 | 11_10641 | 5H | 69.9 | 0.452 | 0.74131 | 0.817571 |
| 3 | 12_30080 | 5H | 69.9 | 0.13 | 0.353183 | 0.654164 |
| 3 | 11_11249 | 5H | 70.5 | 0.061 | 0.86896 | 0.866507 |
| 3 | 11_20392 | 5H | 70.5 | 0.061 | 0.86896 | 0.866507 |
| 3 | 12_30007 | 5H | 70.5 | 0.061 | 0.86896 | 0.866507 |
| 3 | 12_20350 | 5H | 72.3 | 0.473 | 0.336512 | 0.648583 |
| 3 | 11_20367 | 5H | 75.4 | 0.334 | 0.5236 | 0.753599 |
| 3 | 11_21001 | 5H | 75.4 | 0.281 | 0.463447 | 0.717083 |
| 3 | 11_20236 | 5H | 80.6 | 0.209 | 0.809096 | 0.853428 |
| 3 | 11_21133 | 5H | 80.6 | 0.092 | 0.618016 | 0.769383 |
| 3 | 12_10634 | 5H | 80.6 | 0.209 | 0.618016 | 0.769383 |
| 3 | 11_20246 | 5H | 85.2 | 0.209 | 0.618016 | 0.769383 |
| 3 | 11_11355 | 5H | 86.6 | 1.355 | 0.044157 | 0.582372 |
| 3 | 11_20645 | 5H | 87.3 | 0.855 | 0.139637 | 0.59212 |
| 3 | 12_30314 | 5H | 87.3 | 0.423 | 0.377572 | 0.668421 |
| 3 | 12_31427 | 5H | 90.8 | 1.355 | 0.044157 | 0.582372 |
| 3 | 11_11290 | 5H | 94.4 | 1.06 | 0.833681 | 0.861053 |
| 3 | 11_21150 | 5H | 94.4 | 0.079 | 0.087096 | 0.582372 |
| 3 | 11_20526 | 5H | 99.6 | 0.438 | 0.364754 | 0.654711 |
| 3 | 11_10518 | 5H | 100.3 | 0.545 | 0.54325 | 0.754654 |
| 3 | 11_11473 | 5H | 100.3 | 0.265 | 0.028314 | 0.521931 |
| 3 | 11_20097 | 5H | 100.3 | 1.032 | 0.092897 | 0.582372 |
| 3 | 11_20449 | 5H | 100.3 | 1.548 | 0.028314 | 0.521931 |
| 3 | 12_30533 | 5H | 100.3 | 1.548 | 0.242103 | 0.641355 |
| 3 | 12_30834 | 5H | 100.3 | 0.616 | 0.285102 | 0.641355 |
| 3 | 11_20850 | 5H | 102.1 | 0.047 | 0.897429 | 0.869833 |
| 3 | 11_10622 | 5H | 103 | 0.245 | 0.568853 | 0.759523 |
| 3 | 11_21421 | 5H | 103.7 | 0.44 | 0.363078 | 0.654711 |
| 3 | 11_10414 | 5H | 103.9 | 0.581 | 0.765597 | 0.829391 |
| 3 | 11_20327 | 5H | 103.9 | 0.116 | 0.262422 | 0.641355 |
| 3 | 11_11350 | 5H | 104.5 | 1.453 | 0.035237 | 0.573415 |
| 3 | 12_30098 | 5H | 105.2 | 0.208 | 0.619441 | 0.769383 |
| 3 | 11_20018 | 5H | 106.1 | 0.208 | 0.619441 | 0.769383 |
| 3 | 11_20134 | 5H | 106.2 | 0.06 | 0.870964 | 0.866507 |
| 3 | 11_10024 | 5H | 107.6 | 0.006 | 0.98628 | 0.875778 |
| 3 | 11_20549 | 5H | 108 | 0.011 | 0.97499 | 0.875024 |
| 3 | 11_10834 | 5H | 108.2 | 0.763 | 0.206538 | 0.641355 |
| 3 | 11_20320 | 5H | 108.2 | 0.636 | 0.335738 | 0.648583 |
| 3 | 11_21321 | 5H | 108.2 | 0.685 | 0.017783 | 0.430287 |
| 3 | 12_10844 | 5H | 108.2 | 0.763 | 0.57544 | 0.759523 |
| 3 | 12_30846 | 5H | 108.2 | 0.474 | 0.172584 | 0.601743 |
| 3 | 12_30847 | 5H | 108.2 | 1.75 | 0.172584 | 0.601743 |
| 3 | 12_30852 | 5H | 108.2 | 0.763 | 0.231207 | 0.641355 |
| 3 | 12_30854 | 5H | 108.2 | 0.24 | 0.172584 | 0.601743 |
| 3 | 12_31417 | 5H | 108.2 | 0.235 | 0.582103 | 0.760418 |
| 3 | 11_20795 | 5H | 108.6 | 0.06 | 0.870964 | 0.866507 |
| 3 | 11_21168 | 5H | 109.6 | 0.263 | 0.545758 | 0.756328 |
| 3 | 11_20805 | 5H | 110.3 | 2.347 | 0.08995 | 0.582372 |
| 3 | 11_21061 | 5H | 110.3 | 1.622 | 0.172584 | 0.601743 |
| 3 | 12_10507 | 5H | 110.3 | 1.046 | 0.000942 | 0.211595 |
| 3 | 12_30705 | 5H | 110.3 | 0.763 | 0.004498 | 0.248734 |
| 3 | 12_31236 | 5H | 110.3 | 3.026 | 0.023878 | 0.503592 |
| 3 | 11_11273 | 5H | 111.7 | 1.999 | 0.010023 | 0.415715 |
| 3 | 11_10477 | 5H | 113.1 | 1.492 | 0.032211 | 0.558315 |
| 3 | 12_30456 | 5H | 113.1 | 0.025 | 0.944061 | 0.8729 |
| 3 | 11_11341 | 5H | 113.8 | 0.032 | 0.928966 | 0.869833 |
| 3 | 12_30619 | 5H | 113.8 | 0.02 | 0.954993 | 0.875024 |
| 3 | 12_11245 | 5H | 118.8 | 0.61 | 0.245471 | 0.641355 |
| 3 | 11_20629 | 5H | 122.4 | 2.007 | 0.00984 | 0.415715 |
| 3 | 12_11298 | 5H | 123.3 | 0.588 | 0.258226 | 0.641355 |
| 3 | 11_20127 | 5H | 123.5 | 0.803 | 0.157398 | 0.59212 |
| 3 | 12_31278 | 5H | 124.2 | 0.609 | 0.246037 | 0.641355 |
| 3 | 11_11507 | 5H | 125.8 | 0.476 | 0.334195 | 0.648583 |
| 3 | 12_20045 | 5H | 127.2 | 0.176 | 0.666807 | 0.783785 |
| 3 | 11_11456 | 5H | 128 | 1.28 | 0.052481 | 0.582372 |
| 3 | 11_10360 | 5H | 129.4 | 0.432 | 0.948419 | 0.873452 |
| 3 | 11_20003 | 5H | 129.4 | 0.258 | 0.552077 | 0.759523 |
| 3 | 11_20653 | 5H | 129.4 | 0.211 | 0.615177 | 0.769383 |
| 3 | 11_21203 | 5H | 129.4 | 0.218 | 0.63387 | 0.773384 |
| 3 | 11_21325 | 5H | 129.4 | 0.198 | 0.605341 | 0.769383 |
| 3 | 12_30169 | 5H | 129.4 | 0.054 | 0.88308 | 0.866507 |
| 3 | 12_30611 | 5H | 129.4 | 0.023 | 0.369828 | 0.660755 |
| 3 | 11_20300 | 5H | 130.1 | 0.972 | 0.10666 | 0.59212 |
| 3 | 11_11375 | 5H | 130.8 | 0.416 | 0.383707 | 0.672645 |
| 3 | 12_30067 | 5H | 131.6 | 0.364 | 0.432514 | 0.70745 |
| 3 | 11_11090 | 5H | 132.6 | 1.191 | 0.064417 | 0.582372 |
| 3 | 11_20259 | 5H | 132.6 | 1.191 | 0.064417 | 0.582372 |
| 3 | 11_21177 | 5H | 132.6 | 1.191 | 0.064417 | 0.582372 |
| 3 | 11_21247 | 5H | 132.6 | 1.191 | 0.064417 | 0.582372 |
| 3 | 11_20487 | 5H | 134.6 | 2.306 | 0.004943 | 0.257174 |
| 3 | 11_10783 | 5H | 135.7 | 2.355 | 0.004416 | 0.248734 |
| 3 | 12_30883 | 5H | 135.7 | 0.265 | 0.54325 | 0.754654 |
| 3 | 12_30668 | 5H | 136.4 | 2.355 | 0.004416 | 0.248734 |
| 3 | 12_30869 | 5H | 136.4 | 2.355 | 0.004416 | 0.248734 |
| 3 | 11_10095 | 5H | 137.2 | 0.031 | 0.931108 | 0.869833 |
| 3 | 11_10855 | 5H | 137.2 | 0.276 | 0.55847 | 0.759523 |
| 3 | 11_11080 | 5H | 137.2 | 0.031 | 0.020464 | 0.45809 |
| 3 | 11_20884 | 5H | 137.2 | 1.689 | 0.529663 | 0.753599 |
| 3 | 11_21241 | 5H | 137.2 | 1.34 | 0.931108 | 0.869833 |
| 3 | 12_30930 | 5H | 137.2 | 0.253 | 0.045709 | 0.582372 |
| 3 | 12_31237 | 5H | 137.2 | 0.031 | 0.931108 | 0.869833 |
| 3 | 12_30635 | 5H | 140.8 | 0.124 | 0.751623 | 0.824248 |
| 3 | 11_10755 | 5H | 142.2 | 0.045 | 0.484172 | 0.727402 |
| 3 | 11_10845 | 5H | 142.2 | 0.315 | 0.301995 | 0.641355 |
| 3 | 11_11071 | 5H | 142.2 | 0.098 | 0.901571 | 0.869833 |
| 3 | 11_11532 | 5H | 142.2 | 0.098 | 0.797995 | 0.845558 |
| 3 | 11_21289 | 5H | 142.2 | 0.52 | 0.301995 | 0.641355 |
| 3 | 12_31366 | 5H | 142.2 | 0.52 | 0.797995 | 0.845558 |
| 3 | 11_10819 | 5H | 143.9 | 1.13 | 0.074131 | 0.582372 |
| 3 | 11_20375 | 5H | 143.9 | 1.13 | 0.074131 | 0.582372 |
| 3 | 12_30556 | 5H | 143.9 | 0.665 | 0.216272 | 0.641355 |
| 3 | 11_10292 | 5H | 144.6 | 1.13 | 0.074131 | 0.582372 |
| 3 | 12_31217 | 5H | 144.6 | 1.045 | 0.090157 | 0.582372 |
| 3 | 11_11092 | 5H | 145.3 | 1.041 | 0.090991 | 0.582372 |
| 3 | 11_10104 | 5H | 146 | 0.854 | 0.139959 | 0.59212 |
| 3 | 11_20676 | 5H | 146 | 0.854 | 0.139959 | 0.59212 |
| 3 | 11_20731 | 5H | 146 | 0.854 | 0.139959 | 0.59212 |
| 3 | 11_21077 | 5H | 146 | 0.048 | 0.895365 | 0.869833 |
| 3 | 11_20791 | 5H | 149.6 | 0.629 | 0.234963 | 0.641355 |
| 3 | 11_21297 | 5H | 149.6 | 0.629 | 0.234963 | 0.641355 |
| 3 | 11_20388 | 5H | 150.3 | 0.078 | 0.835603 | 0.861053 |
| 3 | 11_10363 | 5H | 151.4 | 0.357 | 0.439542 | 0.708626 |
| 3 | 11_20100 | 5H | 151.4 | 0.077 | 0.860994 | 0.865709 |
| 3 | 11_21360 | 5H | 151.4 | 0.065 | 0.439542 | 0.708626 |
| 3 | 12_30062 | 5H | 151.4 | 0.357 | 0.439542 | 0.708626 |
| 3 | 12_30183 | 5H | 151.4 | 0.357 | 0.993116 | 0.875778 |
| 3 | 12_31050 | 5H | 151.4 | 0.003 | 0.837529 | 0.861565 |
| 3 | 12_31165 | 5H | 151.4 | 0.357 | 0.439542 | 0.708626 |
| 3 | 12_30795 | 5H | 152.8 | 0.013 | 0.9977 | 0.875778 |
| 3 | 12_31221 | 5H | 152.8 | 0.001 | 0.97051 | 0.875024 |
| 3 | 11_10217 | 5H | 153.5 | 0.571 | 0.268534 | 0.641355 |
| 3 | 11_10589 | 5H | 153.5 | 0.571 | 0.268534 | 0.641355 |
| 3 | 11_20104 | 5H | 153.5 | 0.571 | 0.268534 | 0.641355 |
| 3 | 11_21355 | 5H | 153.5 | 0.571 | 0.498885 | 0.738048 |
| 3 | 12_10016 | 5H | 153.5 | 0.302 | 0.268534 | 0.641355 |
| 3 | 11_11490 | 5H | 153.6 | 0.801 | 0.158125 | 0.59212 |
| 3 | 11_11497 | 5H | 155.1 | 0.017 | 0.961612 | 0.875024 |
| 3 | 11_10901 | 5H | 158.4 | 0.041 | 0.909913 | 0.869833 |
| 3 | 11_10820 | 5H | 159.1 | 0.602 | 0.937562 | 0.871665 |
| 3 | 11_11185 | 5H | 159.1 | 0.028 | 0.250035 | 0.641355 |
| 3 | 11_21041 | 5H | 159.1 | 0.023 | 0.948419 | 0.873452 |
| 3 | 11_20545 | 5H | 159.8 | 0.473 | 0.336512 | 0.648583 |
| 3 | 12_30162 | 5H | 161.6 | 0.238 | 0.578096 | 0.759523 |
| 3 | 12_31375 | 5H | 161.6 | 0.237 | 0.579429 | 0.759523 |
| 3 | 11_20546 | 5H | 172.4 | 0.19 | 0.657658 | 0.779342 |
| 3 | 11_20686 | 5H | 172.4 | 0.182 | 0.645654 | 0.773384 |
| 3 | 11_10869 | 5H | 173.1 | 1.423 | 0.037757 | 0.573415 |
| 3 | 11_21141 | 5H | 177.1 | 1.688 | 0.020512 | 0.45809 |
| 3 | 12_11010 | 5H | 178.4 | 0.797 | 0.159588 | 0.59212 |
| 3 | 12_11450 | 5H | 178.4 | 0.8 | 0.158489 | 0.59212 |
| 3 | 11_10254 | 5H | 179.1 | 0.8 | 0.158489 | 0.59212 |
| 3 | 11_21138 | 5H | 179.6 | 1.098 | 0.079799 | 0.582372 |
| 3 | 12_30656 | 5H | 179.6 | 1.098 | 0.079799 | 0.582372 |
| 3 | 11_10736 | 5H | 180.7 | 0.004 | 0.990832 | 0.875778 |
| 3 | 11_10236 | 5H | 181.4 | 0.35 | 0.993116 | 0.875778 |
| 3 | 11_20189 | 5H | 181.4 | 0.003 | 0.446684 | 0.716499 |
| 3 | 11_20897 | 5H | 182.9 | 0.468 | 0.340408 | 0.648583 |
| 3 | 12_30577 | 5H | 182.9 | 0.408 | 0.809096 | 0.853428 |
| 3 | 12_30769 | 5H | 182.9 | 0.092 | 0.390841 | 0.6805 |
| 3 | 11_21155 | 5H | 187.4 | 0.123 | 0.753356 | 0.824512 |
| 3 | 11_10310 | 5H | 188 | 0.595 | 0.254097 | 0.641355 |
| 3 | 11_20786 | 5H | 189.6 | 0.468 | 0.625173 | 0.772371 |
| 3 | 11_21052 | 5H | 189.6 | 0.204 | 0.185353 | 0.606353 |
| 3 | 12_31292 | 5H | 189.6 | 0.732 | 0.340408 | 0.648583 |
| 3 | 12_31481 | 5H | 192 | 0.322 | 0.476431 | 0.725153 |
| 3 | 11_20402 | 5H | 195.4 | 0.216 | 0.608135 | 0.769383 |
| 3 | 12_10322 | 5H | 196.1 | 0.111 | 0.774462 | 0.83201 |
| 3 | 12_31123 | 5H | 196.8 | 0.111 | 0.774462 | 0.83201 |
| 3 | 11_10496 | 6H | 0 | 0.108 | 0.628058 | 0.773384 |
| 3 | 11_11329 | 6H | 0 | 0.202 | 0.015631 | 0.42511 |
| 3 | 11_11406 | 6H | 0 | 1.806 | 0.064565 | 0.582372 |
| 3 | 11_20232 | 6H | 0 | 0.944 | 0.113763 | 0.59212 |
| 3 | 11_20292 | 6H | 0 | 0 | 0.77983 | 0.833152 |
| 3 | 11_20336 | 6H | 0 | 1.143 | 1 | 0.876472 |
| 3 | 12_30319 | 6H | 0 | 1.19 | 0.071945 | 0.582372 |
| 3 | 11_20493 | 6H | 1.3 | 0.988 | 0.102802 | 0.582372 |
| 3 | 11_20881 | 6H | 1.3 | 0.988 | 0.102802 | 0.582372 |
| 3 | 11_20886 | 6H | 1.3 | 0.988 | 0.102802 | 0.582372 |
| 3 | 11_10669 | 6H | 2.3 | 0.557 | 0.277332 | 0.641355 |
| 3 | 11_10120 | 6H | 3.1 | 0.55 | 0.281838 | 0.641355 |
| 3 | 11_20882 | 6H | 3.2 | 1.195 | 0.063826 | 0.582372 |
| 3 | 12_30651 | 6H | 4.4 | 1.652 | 0.022284 | 0.48829 |
| 3 | 11_20294 | 6H | 5.4 | 0.371 | 0.425598 | 0.704312 |
| 3 | 11_21204 | 6H | 6.1 | 2.175 | 0.006683 | 0.298525 |
| 3 | 11_20262 | 6H | 8.1 | 1.337 | 0.046026 | 0.582372 |
| 3 | 11_21032 | 6H | 9.1 | 0.067 | 0.857038 | 0.865709 |
| 3 | 11_11479 | 6H | 12.5 | 0.118 | 0.762079 | 0.827123 |
| 3 | 11_20415 | 6H | 13.2 | 0.131 | 0.739605 | 0.817571 |
| 3 | 12_10554 | 6H | 17 | 0.128 | 0.981748 | 0.875778 |
| 3 | 12_30842 | 6H | 17 | 0.008 | 0.744732 | 0.819012 |
| 3 | 11_10064 | 6H | 21.7 | 0.494 | 0.320627 | 0.648583 |
| 3 | 11_10023 | 6H | 22.4 | 0.473 | 0.668344 | 0.784005 |
| 3 | 11_21246 | 6H | 22.4 | 0.175 | 0.325837 | 0.648583 |
| 3 | 12_30843 | 6H | 22.4 | 0.487 | 0.336512 | 0.648583 |
| 3 | 11_10136 | 6H | 24.4 | 1.113 | 0.07709 | 0.582372 |
| 3 | 11_10868 | 6H | 24.4 | 1.125 | 0.282488 | 0.641355 |
| 3 | 11_20315 | 6H | 24.4 | 0.549 | 0.074989 | 0.582372 |
| 3 | 11_10676 | 6H | 28.4 | 0.006 | 0.98628 | 0.875778 |
| 3 | 12_30697 | 6H | 29.1 | 0.356 | 0.440555 | 0.708626 |
| 3 | 12_30673 | 6H | 30.1 | 0.259 | 0.550808 | 0.758798 |
| 3 | 12_31485 | 6H | 30.1 | 0.004 | 0.990832 | 0.875778 |
| 3 | 12_31308 | 6H | 30.7 | 0.207 | 0.620869 | 0.770332 |
| 3 | 11_10799 | 6H | 31.7 | 0.106 | 0.86896 | 0.866507 |
| 3 | 11_10994 | 6H | 31.7 | 0.061 | 0.78343 | 0.835461 |
| 3 | 11_10939 | 6H | 33.7 | 0.847 | 0.142233 | 0.59212 |
| 3 | 11_10427 | 6H | 34.4 | 0.056 | 0.879023 | 0.866507 |
| 3 | 12_30521 | 6H | 38.4 | 0.851 | 0.140929 | 0.59212 |
| 3 | 12_30361 | 6H | 40.8 | 1.196 | 0.06368 | 0.582372 |
| 3 | 11_10061 | 6H | 42.4 | 0.996 | 0.592925 | 0.765939 |
| 3 | 11_10129 | 6H | 42.4 | 0.657 | 0.220293 | 0.641355 |
| 3 | 11_10494 | 6H | 42.4 | 0.65 | 0.223872 | 0.641355 |
| 3 | 11_10882 | 6H | 42.4 | 0.168 | 0.98628 | 0.875778 |
| 3 | 11_20052 | 6H | 42.4 | 0.227 | 0.100925 | 0.582372 |
| 3 | 12_11455 | 6H | 42.4 | 0.251 | 0.717794 | 0.80385 |
| 3 | 12_30665 | 6H | 42.4 | 0.006 | 0.561048 | 0.759523 |
| 3 | 12_30783 | 6H | 42.4 | 0.144 | 0.679204 | 0.79115 |
| 3 | 11_10244 | 6H | 43.1 | 0.251 | 0.561048 | 0.759523 |
| 3 | 11_20936 | 6H | 43.1 | 0.188 | 0.648634 | 0.773384 |
| 3 | 11_10462 | 6H | 44.8 | 0.415 | 0.384592 | 0.672645 |
| 3 | 11_20743 | 6H | 44.8 | 0.415 | 0.384592 | 0.672645 |
| 3 | 11_10013 | 6H | 45.4 | 0.394 | 0.403645 | 0.692413 |
| 3 | 11_11097 | 6H | 45.4 | 0.394 | 0.403645 | 0.692413 |
| 3 | 12_10199 | 6H | 45.4 | 0.122 | 0.755092 | 0.824512 |
| 3 | 12_10575 | 6H | 45.4 | 0.321 | 0.755092 | 0.824512 |
| 3 | 12_10811 | 6H | 45.4 | 0.122 | 0.477529 | 0.725647 |
| 3 | 12_30658 | 6H | 48.7 | 0.394 | 0.403645 | 0.692413 |
| 3 | 11_20291 | 6H | 49.4 | 0.012 | 0.972747 | 0.875024 |
| 3 | 12_30510 | 6H | 49.4 | 0.012 | 0.972747 | 0.875024 |
| 3 | 11_11205 | 6H | 50.1 | 0.23 | 0.972747 | 0.875024 |
| 3 | 11_20675 | 6H | 50.1 | 0.012 | 0.57544 | 0.759523 |
| 3 | 12_30782 | 6H | 50.1 | 0.24 | 0.588844 | 0.765029 |
| 3 | 12_30569 | 6H | 51.4 | 0.158 | 0.695024 | 0.796791 |
| 3 | 11_10003 | 6H | 52.8 | 0.23 | 0.588844 | 0.765029 |
| 3 | 12_30473 | 6H | 52.8 | 0.158 | 0.695024 | 0.796791 |
| 3 | 11_10227 | 6H | 55.9 | 0.24 | 0.49545 | 0.736533 |
| 3 | 11_10377 | 6H | 55.9 | 0.307 | 0.57544 | 0.759523 |
| 3 | 11_10513 | 6H | 55.9 | 0.296 | 0.948419 | 0.873452 |
| 3 | 11_20835 | 6H | 55.9 | 0.305 | 0.493174 | 0.736164 |
| 3 | 12_31178 | 6H | 55.9 | 0.023 | 0.505825 | 0.744521 |
| 3 | 12_30144 | 6H | 56.5 | 0.246 | 0.567545 | 0.759523 |
| 3 | 12_30857 | 6H | 56.5 | 0.246 | 0.567545 | 0.759523 |
| 3 | 11_11067 | 6H | 58 | 2.191 | 0.220293 | 0.641355 |
| 3 | 12_11253 | 6H | 58 | 0.657 | 0.006442 | 0.298525 |
| 3 | 11_21339 | 6H | 58.5 | 0.546 | 0.284446 | 0.641355 |
| 3 | 11_10964 | 6H | 59.6 | 0.546 | 0.284446 | 0.641355 |
| 3 | 11_20266 | 6H | 59.6 | 0.549 | 0.282488 | 0.641355 |
| 3 | 11_10189 | 6H | 60.2 | 0.43 | 0.371535 | 0.661769 |
| 3 | 11_10270 | 6H | 60.2 | 0.194 | 0.874984 | 0.866507 |
| 3 | 11_10635 | 6H | 60.2 | 0.369 | 0.639735 | 0.773384 |
| 3 | 11_20058 | 6H | 60.2 | 0.058 | 0.427563 | 0.704312 |
| 3 | 11_21310 | 6H | 60.2 | 0.194 | 0.427563 | 0.704312 |
| 3 | 12_30346 | 6H | 60.2 | 0.369 | 0.639735 | 0.773384 |
| 3 | 11_21298 | 6H | 63.3 | 0.165 | 0.683912 | 0.792659 |
| 3 | 11_21069 | 6H | 64 | 0.797 | 0.159588 | 0.59212 |
| 3 | 11_10455 | 6H | 64.4 | 0.669 | 0.28119 | 0.641355 |
| 3 | 11_10781 | 6H | 64.4 | 2.664 | 0.214289 | 0.641355 |
| 3 | 11_20287 | 6H | 64.4 | 0.551 | 0.002168 | 0.211595 |
| 3 | 11_20904 | 6H | 64.4 | 0.228 | 0.591562 | 0.765029 |
| 3 | 11_21225 | 6H | 64.4 | 0.279 | 0.591562 | 0.765029 |
| 3 | 11_21293 | 6H | 64.4 | 0.228 | 0.526017 | 0.753599 |
| 3 | 12_30637 | 6H | 64.4 | 0.228 | 0.591562 | 0.765029 |
| 3 | 11_10040 | 6H | 65 | 1.806 | 0.259418 | 0.641355 |
| 3 | 11_10124 | 6H | 65 | 0.586 | 0.015631 | 0.42511 |
| 3 | 11_11261 | 6H | 65 | 0.014 | 0.968278 | 0.875024 |
| 3 | 11_20468 | 6H | 67.7 | 1.806 | 0.015631 | 0.42511 |
| 3 | 11_20636 | 6H | 67.7 | 1.806 | 0.015631 | 0.42511 |
| 3 | 11_21469 | 6H | 67.7 | 1.806 | 0.015631 | 0.42511 |
| 3 | 12_31289 | 6H | 69.4 | 1.806 | 0.015631 | 0.42511 |
| 3 | 11_20620 | 6H | 70 | 1.806 | 0.015631 | 0.42511 |
| 3 | 11_20892 | 6H | 70 | 0.02 | 0.954993 | 0.875024 |
| 3 | 11_11349 | 6H | 71.1 | 2.649 | 0.002244 | 0.211595 |
| 3 | 11_20784 | 6H | 71.1 | 0.001 | 0.9977 | 0.875778 |
| 3 | 12_31250 | 6H | 71.1 | 0.004 | 0.990832 | 0.875778 |
| 3 | 11_11459 | 6H | 71.9 | 2.649 | 0.002244 | 0.211595 |
| 3 | 11_21256 | 6H | 71.9 | 2.649 | 0.002244 | 0.211595 |
| 3 | 12_31101 | 6H | 71.9 | 0.001 | 0.9977 | 0.875778 |
| 3 | 11_10469 | 6H | 72.5 | 0.001 | 0.9977 | 0.875778 |
| 3 | 12_30940 | 6H | 72.5 | 0.081 | 0.9977 | 0.875778 |
| 3 | 12_31111 | 6H | 72.5 | 0.001 | 0.829851 | 0.861053 |
| 3 | 12_31088 | 6H | 74.5 | 0.001 | 0.9977 | 0.875778 |
| 3 | 11_20889 | 6H | 75.2 | 0.056 | 0.879023 | 0.866507 |
| 3 | 11_21224 | 6H | 77.9 | 0.054 | 0.88308 | 0.866507 |
| 3 | 11_10608 | 6H | 80.5 | 0.021 | 0.952796 | 0.875024 |
| 3 | 11_10220 | 6H | 81.2 | 0.046 | 0.899498 | 0.869833 |
| 3 | 11_11458 | 6H | 81.2 | 0.046 | 0.899498 | 0.869833 |
| 3 | 12_30698 | 6H | 86.9 | 1.598 | 0.025235 | 0.505273 |
| 3 | 11_10400 | 6H | 88.9 | 0.334 | 0.463447 | 0.717083 |
| 3 | 11_20783 | 6H | 88.9 | 0.208 | 0.619441 | 0.769383 |
| 3 | 11_21025 | 6H | 89.6 | 0.187 | 0.65013 | 0.774372 |
| 3 | 11_10202 | 6H | 90.2 | 0.642 | 0.228034 | 0.641355 |
| 3 | 12_31235 | 6H | 91.8 | 0.642 | 0.228034 | 0.641355 |
| 3 | 12_31225 | 6H | 93.1 | 1.514 | 0.03062 | 0.549592 |
| 3 | 11_11294 | 6H | 93.7 | 0.51 | 0.30903 | 0.643161 |
| 3 | 11_20728 | 6H | 93.7 | 0.193 | 0.64121 | 0.773384 |
| 3 | 11_10595 | 6H | 94.7 | 0.838 | 0.145211 | 0.59212 |
| 3 | 11_10978 | 6H | 94.7 | 0.612 | 0.325837 | 0.648583 |
| 3 | 11_20972 | 6H | 94.7 | 0.487 | 0.244343 | 0.641355 |
| 3 | 12_31432 | 6H | 94.7 | 0.02 | 0.954993 | 0.875024 |
| 3 | 11_10734 | 6H | 96.7 | 0.541 | 0.28774 | 0.641355 |
| 3 | 11_10015 | 6H | 97.4 | 0.541 | 0.28774 | 0.641355 |
| 3 | 11_10139 | 6H | 97.4 | 0.302 | 0.860994 | 0.865709 |
| 3 | 11_20118 | 6H | 97.4 | 0.065 | 0.935406 | 0.871139 |
| 3 | 11_20531 | 6H | 97.4 | 0.065 | 0.498885 | 0.738048 |
| 3 | 12_31044 | 6H | 97.4 | 0.135 | 0.877001 | 0.866507 |
| 3 | 12_31048 | 6H | 97.4 | 0.029 | 0.732825 | 0.813621 |
| 3 | 12_31049 | 6H | 97.4 | 0.057 | 0.877001 | 0.866507 |
| 3 | 12_31353 | 6H | 97.4 | 0.057 | 0.860994 | 0.865709 |
| 3 | 12_31115 | 6H | 100.1 | 0.722 | 0.189671 | 0.613564 |
| 3 | 11_20379 | 6H | 101.4 | 0.136 | 0.731139 | 0.812527 |
| 3 | 11_20036 | 6H | 105.6 | 0.144 | 0.961612 | 0.875024 |
| 3 | 11_20467 | 6H | 105.6 | 0.017 | 0.717794 | 0.80385 |
| 3 | 11_20725 | 6H | 105.6 | 0.144 | 0.717794 | 0.80385 |
| 3 | 11_21271 | 6H | 105.6 | 0.144 | 0.717794 | 0.80385 |
| 3 | 11_20355 | 6H | 110.3 | 0.266 | 0.542001 | 0.754654 |
| 3 | 12_30734 | 6H | 110.3 | 0.171 | 0.674528 | 0.788075 |
| 3 | 11_10239 | 6H | 112.3 | 1.13 | 0.97051 | 0.875024 |
| 3 | 11_11534 | 6H | 112.3 | 0.013 | 0.074131 | 0.582372 |
| 3 | 11_20558 | 6H | 112.3 | 1.13 | 0.273527 | 0.641355 |
| 3 | 11_20733 | 6H | 112.3 | 1.13 | 0.074131 | 0.582372 |
| 3 | 12_31495 | 6H | 112.3 | 0.563 | 0.074131 | 0.582372 |
| 3 | 11_10645 | 6H | 118.3 | 0.373 | 0.52723 | 0.753599 |
| 3 | 12_31277 | 6H | 118.3 | 0.278 | 0.423643 | 0.704312 |
| 3 | 11_10107 | 6H | 119 | 0.373 | 0.676083 | 0.789098 |
| 3 | 11_10175 | 6H | 119 | 0.17 | 0.423643 | 0.704312 |
| 3 | 12_10051 | 6H | 119 | 0.373 | 0.423643 | 0.704312 |
| 3 | 12_31392 | 6H | 119 | 0.165 | 0.683912 | 0.792659 |
| 3 | 11_21455 | 6H | 119.7 | 0.373 | 0.423643 | 0.704312 |
| 3 | 11_21467 | 6H | 119.7 | 0.165 | 0.683912 | 0.792659 |
| 3 | 11_11187 | 6H | 121.2 | 0.102 | 0.548277 | 0.758798 |
| 3 | 12_30057 | 6H | 121.2 | 0.369 | 0.790679 | 0.840875 |
| 3 | 12_31126 | 6H | 121.2 | 0.261 | 0.427563 | 0.704312 |
| 3 | 11_20005 | 6H | 122.5 | 0.084 | 0.824138 | 0.861053 |
| 3 | 11_10748 | 6H | 123.8 | 0.242 | 0.824138 | 0.861053 |
| 3 | 11_11488 | 6H | 123.8 | 0.084 | 0.572796 | 0.759523 |
| 3 | 11_20211 | 6H | 123.8 | 0.084 | 0.824138 | 0.861053 |
| 3 | 11_10828 | 6H | 124.8 | 0.259 | 0.765597 | 0.829391 |
| 3 | 11_20687 | 6H | 124.8 | 0.599 | 0.714496 | 0.80385 |
| 3 | 11_20868 | 6H | 124.8 | 0.116 | 0.251768 | 0.641355 |
| 3 | 12_30414 | 6H | 124.8 | 0.146 | 0.550808 | 0.758798 |
| 3 | 12_31283 | 6H | 124.8 | 0.108 | 0.77983 | 0.833152 |
| 3 | 12_31498 | 6H | 126.2 | 0.2 | 0.630957 | 0.773384 |
| 3 | 11_21112 | 6H | 126.8 | 0.599 | 0.251768 | 0.641355 |
| 3 | 11_10390 | 6H | 128.5 | 0.887 | 0.877001 | 0.866507 |
| 3 | 11_11111 | 6H | 128.5 | 0.057 | 0.129718 | 0.59212 |
| 3 | 11_20537 | 6H | 129.4 | 1.067 | 0.306902 | 0.641355 |
| 3 | 12_30627 | 6H | 129.4 | 0.513 | 0.085704 | 0.582372 |
| 3 | 12_30956 | 6H | 129.4 | 0.573 | 0.267301 | 0.641355 |
| 3 | 11_10700 | 7H | 0 | 1.076 | 0.289734 | 0.641355 |
| 3 | 11_11343 | 7H | 0 | 1.475 | 0.688652 | 0.79419 |
| 3 | 11_20076 | 7H | 0 | 1.065 | 0.210863 | 0.641355 |
| 3 | 11_20303 | 7H | 0 | 0.162 | 0.085901 | 0.582372 |
| 3 | 11_20623 | 7H | 0 | 1.044 | 0.893306 | 0.86886 |
| 3 | 11_20691 | 7H | 0 | 1.089 | 0.328095 | 0.648583 |
| 3 | 11_21419 | 7H | 0 | 0.676 | 0.083946 | 0.582372 |
| 3 | 11_21516 | 7H | 0 | 1.066 | 0.086099 | 0.582372 |
| 3 | 12_20016 | 7H | 0 | 0.049 | 0.08147 | 0.582372 |
| 3 | 12_30296 | 7H | 0 | 0.538 | 0.033497 | 0.563774 |
| 3 | 12_30959 | 7H | 0 | 0.484 | 0.090365 | 0.582372 |
| 3 | 12_30472 | 7H | 0.6 | 1.065 | 0.086099 | 0.582372 |
| 3 | 11_10121 | 7H | 1.9 | 1.426 | 0.037497 | 0.573415 |
| 3 | 11_10894 | 7H | 1.9 | 1.441 | 0.036224 | 0.573415 |
| 3 | 11_21307 | 7H | 1.9 | 1.426 | 0.037497 | 0.573415 |
| 3 | 11_20710 | 7H | 3.3 | 1.406 | 0.039264 | 0.573415 |
| 3 | 12_31350 | 7H | 3.3 | 0.189 | 0.647143 | 0.773384 |
| 3 | 11_20242 | 7H | 4.9 | 1.398 | 0.039994 | 0.573415 |
| 3 | 12_30836 | 7H | 4.9 | 1.406 | 0.039264 | 0.573415 |
| 3 | 12_11433 | 7H | 6.8 | 0.18 | 0.660693 | 0.782141 |
| 3 | 11_20307 | 7H | 9.8 | 1.418 | 0.038194 | 0.573415 |
| 3 | 11_20245 | 7H | 12.4 | 0.12 | 0.758578 | 0.824865 |
| 3 | 11_20722 | 7H | 19.2 | 0.635 | 0.23174 | 0.641355 |
| 3 | 11_21050 | 7H | 19.9 | 0.473 | 0.336512 | 0.648583 |
| 3 | 11_10025 | 7H | 21.1 | 0.439 | 0.363915 | 0.654711 |
| 3 | 11_20495 | 7H | 25.7 | 0.178 | 0.663743 | 0.783356 |
| 3 | 12_30329 | 7H | 28.3 | 0.043 | 0.905733 | 0.869833 |
| 3 | 11_10965 | 7H | 29.8 | 0.783 | 0.591562 | 0.765029 |
| 3 | 12_30780 | 7H | 29.8 | 0.228 | 0.164816 | 0.596278 |
| 3 | 11_10920 | 7H | 31.8 | 0.627 | 0.236048 | 0.641355 |
| 3 | 11_20162 | 7H | 31.8 | 0.627 | 0.841395 | 0.861565 |
| 3 | 12_30040 | 7H | 31.8 | 0.075 | 0.236048 | 0.641355 |
| 3 | 12_30063 | 7H | 31.8 | 0.627 | 0.236048 | 0.641355 |
| 3 | 11_10451 | 7H | 32.6 | 0.76 | 0.258226 | 0.641355 |
| 3 | 11_20758 | 7H | 32.6 | 0.588 | 0.17378 | 0.601743 |
| 3 | 11_10232 | 7H | 34.8 | 0.14 | 0.295801 | 0.641355 |
| 3 | 11_20192 | 7H | 34.8 | 0.445 | 0.724436 | 0.808171 |
| 3 | 11_20993 | 7H | 34.8 | 0.902 | 0.724436 | 0.808171 |
| 3 | 12_30083 | 7H | 34.8 | 0.14 | 0.358922 | 0.654711 |
| 3 | 12_30141 | 7H | 34.8 | 0.529 | 0.125314 | 0.59212 |
| 3 | 12_30242 | 7H | 36.8 | 0.501 | 0.315501 | 0.648493 |
| 3 | 11_20126 | 7H | 37.5 | 0.628 | 0.32434 | 0.648583 |
| 3 | 12_30893 | 7H | 37.5 | 1.116 | 0.749894 | 0.82313 |
| 3 | 12_30894 | 7H | 37.5 | 0.125 | 0.235505 | 0.641355 |
| 3 | 12_30895 | 7H | 37.5 | 0.489 | 0.07656 | 0.582372 |
| 3 | 11_10838 | 7H | 38.3 | 0.312 | 0.487529 | 0.730554 |
| 3 | 12_10218 | 7H | 39 | 0.317 | 0.481948 | 0.726881 |
| 3 | 12_31305 | 7H | 41 | 1.062 | 0.086696 | 0.582372 |
| 3 | 11_10576 | 7H | 41.9 | 0.831 | 0.147571 | 0.59212 |
| 3 | 11_10327 | 7H | 42.6 | 0.438 | 0.364754 | 0.654711 |
| 3 | 12_10979 | 7H | 43.4 | 0.438 | 0.364754 | 0.654711 |
| 3 | 12_10368 | 7H | 45.7 | 0.37 | 0.440555 | 0.708626 |
| 3 | 12_30143 | 7H | 45.7 | 0.356 | 0.42658 | 0.704312 |
| 3 | 11_21528 | 7H | 46.2 | 0.462 | 0.345144 | 0.648583 |
| 3 | 11_21491 | 7H | 48.9 | 0.19 | 0.645654 | 0.773384 |
| 3 | 11_21326 | 7H | 49.7 | 0.179 | 0.704693 | 0.799979 |
| 3 | 12_30528 | 7H | 49.7 | 0.152 | 0.662217 | 0.783146 |
| 3 | 11_20249 | 7H | 52.8 | 0.615 | 0.242661 | 0.641355 |
| 3 | 12_30545 | 7H | 53.6 | 1.178 | 0.299916 | 0.641355 |
| 3 | 12_30752 | 7H | 53.6 | 0.523 | 0.066374 | 0.582372 |
| 3 | 11_20074 | 7H | 54.4 | 0.944 | 0.113763 | 0.59212 |
| 3 | 11_20790 | 7H | 55.6 | 1.13 | 0.074131 | 0.582372 |
| 3 | 12_10696 | 7H | 55.6 | 1.097 | 0.079983 | 0.582372 |
| 3 | 12_30290 | 7H | 57.6 | 1.124 | 0.075162 | 0.582372 |
| 3 | 12_30576 | 7H | 58.6 | 0.828 | 0.148594 | 0.59212 |
| 3 | 11_11014 | 7H | 60.7 | 1.483 | 0.032885 | 0.561623 |
| 3 | 11_10346 | 7H | 61.3 | 1.268 | 0.172584 | 0.601743 |
| 3 | 12_10403 | 7H | 61.3 | 0.763 | 0.053951 | 0.582372 |
| 3 | 11_10721 | 7H | 62.9 | 0.739 | 0.18239 | 0.601743 |
| 3 | 11_20975 | 7H | 63.7 | 0.216 | 0.608135 | 0.769383 |
| 3 | 11_20671 | 7H | 68.5 | 0.811 | 0.636796 | 0.773384 |
| 3 | 12_10267 | 7H | 68.5 | 0.196 | 0.154525 | 0.59212 |
| 3 | 11_10431 | 7H | 71.1 | 0.156 | 0.36141 | 0.654711 |
| 3 | 11_11028 | 7H | 71.1 | 0.812 | 0.698232 | 0.79759 |
| 3 | 11_20060 | 7H | 71.1 | 0.281 | 0.5236 | 0.753599 |
| 3 | 11_20195 | 7H | 71.1 | 0.442 | 0.15417 | 0.59212 |
| 3 | 12_30125 | 7H | 71.7 | 0.21 | 0.616595 | 0.769383 |
| 3 | 12_11257 | 7H | 73.8 | 0.675 | 0.211349 | 0.641355 |
| 3 | 12_30496 | 7H | 73.8 | 0.579 | 0.674528 | 0.788075 |
| 3 | 12_30997 | 7H | 73.8 | 0.171 | 0.263633 | 0.641355 |
| 3 | 11_20885 | 7H | 74.5 | 0.998 | 0.100462 | 0.582372 |
| 3 | 12_31120 | 7H | 74.5 | 0.998 | 0.100462 | 0.582372 |
| 3 | 11_10924 | 7H | 77.8 | 0.463 | 0.34435 | 0.648583 |
| 3 | 12_10459 | 7H | 77.8 | 0.463 | 0.469894 | 0.717083 |
| 3 | 12_10698 | 7H | 77.8 | 0.328 | 0.34435 | 0.648583 |
| 3 | 12_30492 | 7H | 77.8 | 0.463 | 0.469894 | 0.717083 |
| 3 | 12_30760 | 7H | 77.8 | 0.463 | 0.34435 | 0.648583 |
| 3 | 12_30794 | 7H | 77.8 | 0.328 | 0.34435 | 0.648583 |
| 3 | 11_10073 | 7H | 78.2 | 0.328 | 0.364754 | 0.654711 |
| 3 | 12_11477 | 7H | 78.2 | 0.438 | 0.30479 | 0.641355 |
| 3 | 12_30004 | 7H | 78.2 | 0.516 | 0.469894 | 0.717083 |
| 3 | 12_31000 | 7H | 78.2 | 0.516 | 0.30479 | 0.641355 |
| 3 | 11_10055 | 7H | 79.6 | 0.328 | 0.469894 | 0.717083 |
| 3 | 11_10773 | 7H | 79.6 | 0.538 | 0.416869 | 0.703664 |
| 3 | 11_11145 | 7H | 79.6 | 0.328 | 0.364754 | 0.654711 |
| 3 | 11_11219 | 7H | 79.6 | 0.38 | 0.289734 | 0.641355 |
| 3 | 11_11352 | 7H | 79.6 | 0.387 | 0.364754 | 0.654711 |
| 3 | 11_21302 | 7H | 79.6 | 0.538 | 0.469894 | 0.717083 |
| 3 | 11_21335 | 7H | 79.6 | 0.438 | 0.410204 | 0.699531 |
| 3 | 12_10713 | 7H | 79.6 | 0.328 | 0.469894 | 0.717083 |
| 3 | 12_11536 | 7H | 79.6 | 0.438 | 0.30479 | 0.641355 |
| 3 | 12_30449 | 7H | 79.6 | 0.328 | 0.364754 | 0.654711 |
| 3 | 12_30475 | 7H | 79.6 | 0.538 | 0.289734 | 0.641355 |
| 3 | 12_30563 | 7H | 79.6 | 0.328 | 0.469894 | 0.717083 |
| 3 | 12_30574 | 7H | 79.6 | 0.438 | 0.289734 | 0.641355 |
| 3 | 12_30600 | 7H | 79.6 | 0.438 | 0.469894 | 0.717083 |
| 3 | 12_30835 | 7H | 79.6 | 1.435 | 0.469894 | 0.717083 |
| 3 | 12_31140 | 7H | 79.6 | 0.516 | 0.364754 | 0.654711 |
| 3 | 12_31345 | 7H | 79.6 | 0.328 | 0.036728 | 0.573415 |
| 3 | 12_31418 | 7H | 79.6 | 0.328 | 0.469894 | 0.717083 |
| 3 | 12_30565 | 7H | 80.9 | 0.814 | 0.153462 | 0.59212 |
| 3 | 11_11461 | 7H | 82.3 | 0.815 | 0.153109 | 0.59212 |
| 3 | 11_10069 | 7H | 83.4 | 4.714 | 1.93E-05 | 0.022436 |
| 3 | 11_10673 | 7H | 83.4 | 1.457 | 0.034914 | 0.573415 |
| 3 | 11_20205 | 7H | 83.4 | 0.651 | 0.350752 | 0.650698 |
| 3 | 11_20349 | 7H | 83.4 | 0.455 | 0.16293 | 0.593148 |
| 3 | 11_21079 | 7H | 83.4 | 0.788 | 0.223357 | 0.641355 |
| 3 | 12_10125 | 7H | 83.4 | 0.651 | 0.257632 | 0.641355 |
| 3 | 12_10982 | 7H | 83.4 | 0.589 | 0.257632 | 0.641355 |
| 3 | 12_11091 | 7H | 83.4 | 0.589 | 0.223357 | 0.641355 |
| 3 | 12_30506 | 7H | 83.4 | 0.455 | 0.350752 | 0.650698 |
| 3 | 11_10442 | 7H | 84.9 | 0.651 | 0.778037 | 0.832768 |
| 3 | 11_10531 | 7H | 84.9 | 0.651 | 0.778037 | 0.832768 |
| 3 | 11_11239 | 7H | 84.9 | 0.651 | 0.778037 | 0.832768 |
| 3 | 11_11445 | 7H | 84.9 | 0.109 | 0.223357 | 0.641355 |
| 3 | 11_20880 | 7H | 84.9 | 0.109 | 0.223357 | 0.641355 |
| 3 | 12_30998 | 7H | 84.9 | 0.109 | 0.223357 | 0.641355 |
| 3 | 11_20896 | 7H | 86.4 | 0.8 | 0.158489 | 0.59212 |
| 3 | 11_21330 | 7H | 86.4 | 0.804 | 0.157036 | 0.59212 |
| 3 | 12_30199 | 7H | 86.4 | 0.804 | 0.157036 | 0.59212 |
| 3 | 12_31137 | 7H | 86.4 | 0.804 | 0.157036 | 0.59212 |
| 3 | 11_21409 | 7H | 87.2 | 0.804 | 0.157036 | 0.59212 |
| 3 | 11_10143 | 7H | 88 | 0.838 | 0.145211 | 0.59212 |
| 3 | 11_10303 | 7H | 88 | 0.829 | 0.148252 | 0.59212 |
| 3 | 11_20083 | 7H | 88 | 0.808 | 0.155597 | 0.59212 |
| 3 | 12_30996 | 7H | 91.8 | 0.391 | 0.406443 | 0.694137 |
| 3 | 11_21201 | 7H | 98.5 | 0.481 | 0.33037 | 0.648583 |
| 3 | 11_21448 | 7H | 98.5 | 0.203 | 0.626614 | 0.773329 |
| 3 | 12_30806 | 7H | 99.7 | 0.204 | 0.625173 | 0.772371 |
| 3 | 12_31395 | 7H | 99.7 | 0.165 | 0.683912 | 0.792659 |
| 3 | 11_20808 | 7H | 101.3 | 0.845 | 0.142889 | 0.59212 |
| 3 | 11_20103 | 7H | 102.8 | 0.574 | 0.266686 | 0.641355 |
| 3 | 12_31261 | 7H | 107.1 | 0.957 | 0.110408 | 0.59212 |
| 3 | 11_20824 | 7H | 107.9 | 0.006 | 0.98628 | 0.875778 |
| 3 | 12_20684 | 7H | 109.7 | 0.055 | 0.881049 | 0.866507 |
| 3 | 11_10853 | 7H | 111 | 1.793 | 0.016106 | 0.42511 |
| 3 | 11_20092 | 7H | 111 | 1.741 | 0.018155 | 0.430287 |
| 3 | 11_20385 | 7H | 111 | 1.741 | 0.018155 | 0.430287 |
| 3 | 11_10563 | 7H | 112.5 | 1.164 | 0.214783 | 0.641355 |
| 3 | 12_10241 | 7H | 112.5 | 0.668 | 0.068549 | 0.582372 |
| 3 | 11_20247 | 7H | 116.3 | 0.027 | 0.939723 | 0.871665 |
| 3 | 12_30797 | 7H | 117 | 1.751 | 0.017742 | 0.430287 |
| 3 | 12_30368 | 7H | 118.9 | 0.912 | 0.122462 | 0.59212 |
| 3 | 12_30164 | 7H | 119.5 | 0.249 | 0.563638 | 0.759523 |
| 3 | 11_11243 | 7H | 122.1 | 0.057 | 0.877001 | 0.866507 |
| 3 | 12_10543 | 7H | 122.1 | 0.014 | 0.968278 | 0.875024 |
| 3 | 11_20354 | 7H | 125.2 | 0.035 | 0.922571 | 0.869833 |
| 3 | 11_10182 | 7H | 128.4 | 0.256 | 0.657658 | 0.779342 |
| 3 | 11_21229 | 7H | 128.4 | 0.182 | 0.554626 | 0.759523 |
| 3 | 12_11279 | 7H | 128.4 | 0.056 | 0.879023 | 0.866507 |
| 3 | 11_21209 | 7H | 129.9 | 0.405 | 0.39355 | 0.683169 |
| 3 | 11_10861 | 7H | 133.8 | 0.711 | 0.194536 | 0.620385 |
| 3 | 11_21104 | 7H | 133.8 | 0.067 | 0.857038 | 0.865709 |
| 3 | 11_10078 | 7H | 136.6 | 0.008 | 0.774462 | 0.83201 |
| 3 | 11_10797 | 7H | 136.6 | 0.176 | 0.981748 | 0.875778 |
| 3 | 11_21160 | 7H | 136.6 | 0.111 | 0.666807 | 0.783785 |
| 3 | 12_30380 | 7H | 138.2 | 0.019 | 0.957194 | 0.875024 |
| 3 | 11_10885 | 7H | 139.7 | 0.08 | 0.831764 | 0.861053 |
| 3 | 11_10454 | 7H | 140.2 | 0.924 | 0.870964 | 0.866507 |
| 3 | 11_20847 | 7H | 140.2 | 0.06 | 0.119124 | 0.59212 |
| 3 | 11_10687 | 7H | 141 | 0.498 | 0.317687 | 0.648583 |
| 3 | 11_20139 | 7H | 141.8 | 0.478 | 0.33266 | 0.648583 |
| 3 | 11_21280 | 7H | 141.8 | 0.478 | 0.712853 | 0.80385 |
| 3 | 12_30761 | 7H | 141.8 | 0.147 | 0.33266 | 0.648583 |
| 3 | 12_31325 | 7H | 143.7 | 0.42 | 0.380189 | 0.671009 |
| 3 | 11_11440 | 7H | 144.4 | 1.16 | 0.069183 | 0.582372 |
| 3 | 11_20414 | 7H | 144.4 | 0.06 | 0.90365 | 0.869833 |
| 3 | 11_20452 | 7H | 144.4 | 0.148 | 0.711214 | 0.803454 |
| 3 | 11_21363 | 7H | 144.4 | 0.044 | 0.26485 | 0.641355 |
| 3 | 12_30593 | 7H | 144.4 | 0.577 | 0.711214 | 0.803454 |
| 3 | 12_31166 | 7H | 144.4 | 0.148 | 0.870964 | 0.866507 |
| 3 | 11_11012 | 7H | 147.5 | 0.67 | 0.213796 | 0.641355 |
| 3 | 11_10130 | 7H | 147.5 | 0.684 | 0.207014 | 0.641355 |
| 3 | 11_10896 | 7H | 148.2 | 0.652 | 0.222844 | 0.641355 |
| 3 | 11_11275 | 7H | 149 | 0.615 | 0.242661 | 0.641355 |
| 3 | 11_20962 | 7H | 149.8 | 0.617 | 0.241546 | 0.641355 |
| 3 | 11_10999 | 7H | 161.4 | 1.059 | 0.087297 | 0.582372 |
| 3 | 12_30974 | 7H | 161.4 | 1.059 | 0.087297 | 0.582372 |
| 3 | 11_20170 | 7H | 161.5 | 0.525 | 0.298538 | 0.641355 |
| 3 | 11_10174 | 7H | 166.6 | 0.119 | 0.707946 | 0.802103 |
| 3 | 11_20365 | 7H | 166.6 | 0.615 | 0.760326 | 0.825993 |
| 3 | 12_30826 | 7H | 166.6 | 0.15 | 0.242661 | 0.641355 |
| 3 | 11_10654 | UNK | 0 | 0.675 | 0.893306 | 0.86886 |
| 3 | 11_20044 | UNK | 0 | 1.845 | 0.717794 | 0.80385 |
| 3 | 11_20125 | UNK | 0 | 0.001 | 0.74131 | 0.817571 |
| 3 | 11_20153 | UNK | 0 | 0.219 | 0.217771 | 0.641355 |
| 3 | 11_20339 | UNK | 0 | 0.001 | 0.574117 | 0.759523 |
| 3 | 11_20479 | UNK | 0 | 0.929 | 0.445656 | 0.71584 |
| 3 | 11_21095 | UNK | 0 | 1.268 | 0.9977 | 0.875778 |
| 3 | 12_10022 | UNK | 0 | 0.832 | 0.638264 | 0.773384 |
| 3 | 12_10032 | UNK | 0 | 0.989 | 0.297167 | 0.641355 |
| 3 | 12_10149 | UNK | 0 | 0.195 | 0.061802 | 0.582372 |
| 3 | 12_10257 | UNK | 0 | 1.259 | 0.185353 | 0.606353 |
| 3 | 12_10393 | UNK | 0 | 0.156 | 0.13213 | 0.59212 |
| 3 | 12_10420 | UNK | 0 | 0.014 | 0.463447 | 0.717083 |
| 3 | 12_10430 | UNK | 0 | 0.279 | 0.928966 | 0.869833 |
| 3 | 12_10491 | UNK | 0 | 0.054 | 0.822243 | 0.861053 |
| 3 | 12_10623 | UNK | 0 | 1.845 | 0.698232 | 0.79759 |
| 3 | 12_10704 | UNK | 0 | 0.853 | 0.465586 | 0.717083 |
| 3 | 12_10752 | UNK | 0 | 0.626 | 0.814704 | 0.857008 |
| 3 | 12_10981 | UNK | 0 | 0.279 | 0.831764 | 0.861053 |
| 3 | 12_11254 | UNK | 0 | 0.527 | 0.046559 | 0.582372 |
| 3 | 12_11255 | UNK | 0 | 1.398 | 0.608135 | 0.769383 |
| 3 | 12_11386 | UNK | 0 | 0.144 | 0.006442 | 0.298525 |
| 3 | 12_11468 | UNK | 0 | 1.209 | 0.055081 | 0.582372 |
| 3 | 12_20323 | UNK | 0 | 1.369 | 0.97051 | 0.875024 |
| 3 | 12_20359 | UNK | 0 | 0.216 | 0.164437 | 0.596278 |
| 3 | 12_20416 | UNK | 0 | 0.049 | 0.003097 | 0.211595 |
| 3 | 12_20632 | UNK | 0 | 0.034 | 0.603949 | 0.769383 |
| 3 | 12_20775 | UNK | 0 | 0.732 | 0.102565 | 0.582372 |
| 3 | 12_21522 | UNK | 0 | 0.879 | 0.071285 | 0.582372 |
| 3 | 12_30002 | UNK | 0 | 1.147 | 0.30761 | 0.641355 |
| 3 | 12_30050 | UNK | 0 | 0.563 | 0.336512 | 0.648583 |
| 3 | 12_30115 | UNK | 0 | 0.045 | 0.86896 | 0.866507 |
| 3 | 12_30118 | UNK | 0 | 0.784 | 0.199067 | 0.628212 |
| 3 | 12_30129 | UNK | 0 | 0.351 | 0.02673 | 0.51597 |
| 3 | 12_30145 | UNK | 0 | 0.58 | 0.829851 | 0.861053 |
| 3 | 12_30147 | UNK | 0 | 2.191 | 0.167494 | 0.601743 |
| 3 | 12_30186 | UNK | 0 | 0.714 | 0.384592 | 0.672645 |
| 3 | 12_30222 | UNK | 0 | 0.333 | 0.946237 | 0.873452 |
| 3 | 12_30285 | UNK | 0 | 0.512 | 0.273527 | 0.641355 |
| 3 | 12_30424 | UNK | 0 | 0.702 | 0.944061 | 0.8729 |
| 3 | 12_30477 | UNK | 0 | 0.013 | 0.193197 | 0.619791 |
| 3 | 12_30603 | UNK | 0 | 0.538 | 0.638264 | 0.773384 |
| 3 | 12_30606 | UNK | 0 | 0.332 | 0.117761 | 0.59212 |
| 3 | 12_30653 | UNK | 0 | 0.404 | 0.968278 | 0.875024 |
| 3 | 12_30655 | UNK | 0 | 0.089 | 0.140281 | 0.59212 |
| 3 | 12_30716 | UNK | 0 | 0.538 | 0.654636 | 0.777347 |
| 3 | 12_30793 | UNK | 0 | 0.645 | 0.505825 | 0.744521 |
| 3 | 12_30819 | UNK | 0 | 0.012 | 0.60256 | 0.769383 |
| 3 | 12_30822 | UNK | 0 | 0.473 | 0.226464 | 0.641355 |
| 3 | 12_30827 | UNK | 0 | 0.061 | 0.298538 | 0.641355 |
| 3 | 12_30877 | UNK | 0 | 0.08 | 0.155239 | 0.59212 |
| 3 | 12_30908 | UNK | 0 | 0.334 | 0.9977 | 0.875778 |
| 3 | 12_30917 | UNK | 0 | 0.22 | 0.49545 | 0.736533 |
| 3 | 12_30926 | UNK | 0 | 0.271 | 0.535797 | 0.754654 |
| 3 | 12_30939 | UNK | 0 | 0.274 | 0.9977 | 0.875778 |
| 3 | 12_30949 | UNK | 0 | 0.001 | 0.039994 | 0.573415 |
| 3 | 12_30957 | UNK | 0 | 0.584 | 0.924698 | 0.869833 |
| 3 | 12_30981 | UNK | 0 | 0.069 | 0.972747 | 0.875024 |
| 3 | 12_30999 | UNK | 0 | 1.332 | 0.147231 | 0.59212 |
| 3 | 12_31054 | UNK | 0 | 0.701 | 0.289734 | 0.641355 |
| 3 | 12_31055 | UNK | 0 | 1.573 | 0.289734 | 0.641355 |
| 3 | 12_31059 | UNK | 0 | 0.13 | 0.014289 | 0.42511 |
| 3 | 12_31065 | UNK | 0 | 0.662 | 0.014289 | 0.42511 |
| 3 | 12_31124 | UNK | 0 | 0.294 | 0.9977 | 0.875778 |
| 3 | 12_31127 | UNK | 0 | 0.081 | 0.236592 | 0.641355 |
| 3 | 12_31128 | UNK | 0 | 1.968 | 0.88308 | 0.866507 |
| 3 | 12_31151 | UNK | 0 | 0.809 | 0.526017 | 0.753599 |
| 3 | 12_31170 | UNK | 0 | 0.776 | 0.263027 | 0.641355 |
| 3 | 12_31181 | UNK | 0 | 0.334 | 0.077268 | 0.582372 |
| 3 | 12_31200 | UNK | 0 | 0.489 | 0.901571 | 0.869833 |
| 3 | 12_31202 | UNK | 0 | 0.241 | 0.010765 | 0.42511 |
| 3 | 12_31203 | UNK | 0 | 0.415 | 0.211349 | 0.641355 |
| 3 | 12_31219 | UNK | 0 | 0.001 | 0.526017 | 0.753599 |
| 3 | 12_31230 | UNK | 0 | 0.024 | 0.260615 | 0.641355 |
| 3 | 12_31239 | UNK | 0 | 0.025 | 0.32434 | 0.648583 |
| 3 | 12_31267 | UNK | 0 | 2.509 | 0.19861 | 0.628212 |
| 3 | 12_31279 | UNK | 0 | 0.184 | 0.464515 | 0.717083 |
| 3 | 12_31295 | UNK | 0 | 0.032 | 0.042756 | 0.582372 |
| 3 | 12_31327 | UNK | 0 | 0.195 | 0.394457 | 0.683721 |
| 3 | 12_31333 | UNK | 0 | 1.112 | 0.8531 | 0.863755 |
| 3 | 12_31357 | UNK | 0 | 0.085 | 0.053951 | 0.582372 |
| 3 | 12_31408 | UNK | 0 | 0.296 | 0.463447 | 0.717083 |
| 3 | 12_31414 | UNK | 0 | 0.305 | 0.532108 | 0.753599 |
| 3 | 12_31484 | UNK | 0 | 0.525 | 0.508159 | 0.747011 |
| 5 | 11_20373 | 1H | 0 | 0.436 | 0.366438 | 0.445072 |
| 5 | 12_30969 | 1H | 0 | 1.122 | 0.075509 | 0.238692 |
| 5 | 11_11223 | 1H | 0.8 | 0.187 | 0.65013 | 0.548015 |
| 5 | 11_20502 | 1H | 1.5 | 1.031 | 0.093111 | 0.263768 |
| 5 | 11_21067 | 1H | 1.5 | 0.132 | 0.737904 | 0.56349 |
| 5 | 11_10419 | 1H | 3.8 | 0.142 | 0.721108 | 0.561313 |
| 5 | 12_31144 | 1H | 3.8 | 0.018 | 0.959401 | 0.615801 |
| 5 | 12_10636 | 1H | 4.5 | 0.308 | 0.49204 | 0.501441 |
| 5 | 12_11011 | 1H | 6 | 0.306 | 0.494311 | 0.501441 |
| 5 | 12_30933 | 1H | 6 | 0.658 | 0.219786 | 0.376442 |
| 5 | 11_21174 | 1H | 8.3 | 2.048 | 0.008954 | 0.097182 |
| 5 | 12_30918 | 1H | 11.4 | 2.633 | 0.002328 | 0.065601 |
| 5 | 12_30951 | 1H | 11.4 | 1.605 | 0.024831 | 0.151306 |
| 5 | 12_30588 | 1H | 13.1 | 0.473 | 0.336512 | 0.431127 |
| 5 | 11_10332 | 1H | 15.4 | 0.508 | 0.310456 | 0.424187 |
| 5 | 12_30948 | 1H | 17.3 | 0.323 | 0.475335 | 0.501441 |
| 5 | 11_10030 | 1H | 18.1 | 2.934 | 0.001164 | 0.05355 |
| 5 | 11_20712 | 1H | 20.8 | 0.031 | 0.931108 | 0.61021 |
| 5 | 11_10873 | 1H | 20.9 | 0.117 | 0.763836 | 0.573322 |
| 5 | 11_10186 | 1H | 23.9 | 0.191 | 0.644169 | 0.548015 |
| 5 | 11_10757 | 1H | 23.9 | 1.894 | 0.012764 | 0.110093 |
| 5 | 11_10744 | 1H | 26.1 | 2.713 | 0.001936 | 0.060341 |
| 5 | 11_10764 | 1H | 41 | 1.068 | 0.085507 | 0.253372 |
| 5 | 12_30336 | 1H | 41.8 | 0.787 | 0.163305 | 0.316138 |
| 5 | 11_10275 | 1H | 42.5 | 1.028 | 0.093756 | 0.264027 |
| 5 | 11_10597 | 1H | 42.5 | 1.051 | 0.08892 | 0.259736 |
| 5 | 11_20514 | 1H | 42.5 | 0.829 | 0.148252 | 0.300864 |
| 5 | 11_11484 | 1H | 51.2 | 1.453 | 0.035237 | 0.16851 |
| 5 | 11_10833 | 1H | 52.5 | 1.453 | 0.035237 | 0.16851 |
| 5 | 11_21312 | 1H | 52.5 | 1.453 | 0.035237 | 0.16851 |
| 5 | 12_30592 | 1H | 52.5 | 1.453 | 0.035237 | 0.16851 |
| 5 | 12_31134 | 1H | 52.5 | 1.453 | 0.035237 | 0.16851 |
| 5 | 12_31272 | 1H | 52.5 | 1.453 | 0.035237 | 0.16851 |
| 5 | 11_10075 | 1H | 55.5 | 1.453 | 0.035237 | 0.16851 |
| 5 | 11_10552 | 1H | 59.7 | 2.451 | 0.00354 | 0.075488 |
| 5 | 12_30820 | 1H | 59.7 | 2.503 | 0.003141 | 0.07023 |
| 5 | 12_30821 | 1H | 59.7 | 2.534 | 0.002924 | 0.07023 |
| 5 | 11_10516 | 1H | 65.5 | 3.619 | 0.00024 | 0.017866 |
| 5 | 11_10043 | 1H | 66 | 3.619 | 0.00024 | 0.017866 |
| 5 | 11_10006 | 1H | 73.9 | 3.094 | 0.000805 | 0.040947 |
| 5 | 11_21126 | 1H | 73.9 | 1.815 | 0.015311 | 0.118322 |
| 5 | 11_20121 | 1H | 75.5 | 0.076 | 0.83946 | 0.580657 |
| 5 | 11_20990 | 1H | 75.5 | 2.624 | 0.002377 | 0.065601 |
| 5 | 11_11037 | 1H | 84.7 | 6.141 | 7.23E-07 | 0.000654 |
| 5 | 11_10434 | 1H | 86.2 | 3.93 | 0.000117 | 0.015302 |
| 5 | 12_30204 | 1H | 86.2 | 2.532 | 0.002938 | 0.07023 |
| 5 | 12_11144 | 1H | 87.6 | 2.391 | 0.004064 | 0.075505 |
| 5 | 11_10471 | 1H | 88.2 | 2.495 | 0.003199 | 0.07023 |
| 5 | 11_10830 | 1H | 88.2 | 2.605 | 0.002483 | 0.066631 |
| 5 | 11_20434 | 1H | 88.2 | 2.391 | 0.004064 | 0.075505 |
| 5 | 11_21192 | 1H | 88.2 | 2.728 | 0.001871 | 0.060341 |
| 5 | 11_21373 | 1H | 95.4 | 0.243 | 0.571479 | 0.527183 |
| 5 | 11_11277 | 1H | 97.7 | 0.029 | 0.935406 | 0.611123 |
| 5 | 11_10522 | 1H | 99.2 | 0.087 | 0.818465 | 0.576266 |
| 5 | 11_20169 | 1H | 100 | 0.081 | 0.829851 | 0.578798 |
| 5 | 11_20754 | 1H | 100 | 0.087 | 0.818465 | 0.576266 |
| 5 | 11_20780 | 1H | 105.1 | 1.111 | 0.077446 | 0.242899 |
| 5 | 11_20921 | 1H | 105.8 | 0.501 | 0.315501 | 0.426853 |
| 5 | 11_20625 | 1H | 106.6 | 0.78 | 0.165959 | 0.317458 |
| 5 | 11_20220 | 1H | 107.5 | 0.137 | 0.729458 | 0.562773 |
| 5 | 11_20844 | 1H | 108.3 | 0.137 | 0.729458 | 0.562773 |
| 5 | 12_30532 | 1H | 109.8 | 0.178 | 0.663743 | 0.551014 |
| 5 | 12_31526 | 1H | 112.9 | 0.235 | 0.582103 | 0.527723 |
| 5 | 11_21392 | 1H | 114.8 | 0.066 | 0.859014 | 0.586851 |
| 5 | 11_10338 | 1H | 117.8 | 1.361 | 0.043551 | 0.18546 |
| 5 | 11_10854 | 1H | 117.8 | 1.079 | 0.083368 | 0.25325 |
| 5 | 11_20908 | 1H | 121.1 | 0.225 | 0.595662 | 0.533775 |
| 5 | 11_21038 | 1H | 121.1 | 0.053 | 0.885116 | 0.597917 |
| 5 | 11_10586 | 1H | 121.8 | 0.272 | 0.534564 | 0.514332 |
| 5 | 12_11443 | 1H | 123.8 | 0.103 | 0.78886 | 0.575737 |
| 5 | 11_10722 | 1H | 125.3 | 0.451 | 0.353997 | 0.440105 |
| 5 | 11_21140 | 1H | 126 | 0.636 | 0.231207 | 0.38083 |
| 5 | 11_11481 | 1H | 126.5 | 0.09 | 0.812831 | 0.576266 |
| 5 | 12_10207 | 1H | 126.5 | 0.614 | 0.24322 | 0.385798 |
| 5 | 12_30403 | 1H | 126.5 | 1.168 | 0.06792 | 0.227028 |
| 5 | 12_31377 | 1H | 126.5 | 1.168 | 0.06792 | 0.227028 |
| 5 | 11_10644 | 1H | 127.1 | 0.631 | 0.233884 | 0.38083 |
| 5 | 11_20383 | 1H | 131.2 | 1.649 | 0.022439 | 0.141673 |
| 5 | 11_10041 | 1H | 135.6 | 0.045 | 0.901571 | 0.602294 |
| 5 | 11_11105 | 1H | 135.6 | 1.295 | 0.050699 | 0.203014 |
| 5 | 11_11509 | 1H | 135.6 | 0.267 | 0.540754 | 0.516685 |
| 5 | 11_20603 | 1H | 135.6 | 0.317 | 0.481948 | 0.501441 |
| 5 | 11_21384 | 1H | 135.6 | 0.159 | 0.693426 | 0.558208 |
| 5 | 11_20594 | 1H | 136.3 | 1.268 | 0.053951 | 0.205995 |
| 5 | 11_20138 | 1H | 137.8 | 1.099 | 0.079616 | 0.245635 |
| 5 | 11_20840 | 1H | 137.8 | 0.571 | 0.268534 | 0.39847 |
| 5 | 11_10590 | 1H | 138.3 | 1.068 | 0.085507 | 0.253372 |
| 5 | 11_20915 | 1H | 138.3 | 1.068 | 0.085507 | 0.253372 |
| 5 | 12_30231 | 1H | 138.3 | 1.174 | 0.066988 | 0.227028 |
| 5 | 11_20772 | 1H | 139.8 | 0.824 | 0.149969 | 0.303074 |
| 5 | 11_10443 | 1H | 140.5 | 1.068 | 0.085507 | 0.253372 |
| 5 | 12_30934 | 1H | 140.5 | 1.038 | 0.091622 | 0.262632 |
| 5 | 12_31081 | 1H | 140.5 | 1.174 | 0.066988 | 0.227028 |
| 5 | 11_10017 | 2H | 0 | 1.939 | 0.011508 | 0.105583 |
| 5 | 11_10352 | 2H | 0 | 0.03 | 0.933254 | 0.611123 |
| 5 | 11_10977 | 2H | 0 | 0.019 | 0.957194 | 0.615801 |
| 5 | 11_10996 | 2H | 0 | 0.039 | 0.914113 | 0.606479 |
| 5 | 11_11346 | 2H | 0 | 0.867 | 0.135831 | 0.299306 |
| 5 | 11_20099 | 2H | 0 | 0.165 | 0.683912 | 0.553663 |
| 5 | 11_20498 | 2H | 0 | 1.057 | 0.0877 | 0.257502 |
| 5 | 11_20609 | 2H | 0 | 0.466 | 0.341979 | 0.432398 |
| 5 | 11_21142 | 2H | 0 | 0.448 | 0.356451 | 0.440886 |
| 5 | 11_21184 | 2H | 0 | 0.153 | 0.703072 | 0.559354 |
| 5 | 12_31224 | 2H | 4.1 | 1.645 | 0.022646 | 0.142055 |
| 5 | 11_10326 | 2H | 6.5 | 1.288 | 0.051523 | 0.203014 |
| 5 | 11_11059 | 2H | 7.1 | 0.26 | 0.549541 | 0.519938 |
| 5 | 11_21377 | 2H | 8.6 | 0.007 | 0.984011 | 0.622303 |
| 5 | 12_30781 | 2H | 8.6 | 1.28 | 0.052481 | 0.2036 |
| 5 | 11_20563 | 2H | 9.3 | 1.798 | 0.015922 | 0.119231 |
| 5 | 12_11119 | 2H | 10.1 | 1 | 0.1 | 0.273369 |
| 5 | 12_30155 | 2H | 10.1 | 2.539 | 0.002891 | 0.07023 |
| 5 | 12_31497 | 2H | 10.1 | 1.17 | 0.067608 | 0.227028 |
| 5 | 11_20562 | 2H | 10.9 | 1.455 | 0.035075 | 0.16851 |
| 5 | 12_30631 | 2H | 15.2 | 0.859 | 0.138357 | 0.299306 |
| 5 | 11_10943 | 2H | 18.3 | 0.979 | 0.104954 | 0.278289 |
| 5 | 12_31284 | 2H | 19.5 | 0.748 | 0.178649 | 0.332001 |
| 5 | 11_10180 | 2H | 21.6 | 0.133 | 0.736207 | 0.56349 |
| 5 | 11_10216 | 2H | 26.5 | 0 | 1 | 0.626459 |
| 5 | 11_21015 | 2H | 27.3 | 0.142 | 0.721108 | 0.561313 |
| 5 | 11_21261 | 2H | 28.4 | 0.083 | 0.826038 | 0.577808 |
| 5 | 11_21265 | 2H | 28.4 | 0.083 | 0.826038 | 0.577808 |
| 5 | 11_21366 | 2H | 28.4 | 0.083 | 0.826038 | 0.577808 |
| 5 | 11_21187 | 2H | 29.1 | 0.1 | 0.794328 | 0.575737 |
| 5 | 11_10307 | 2H | 31 | 0.021 | 0.952796 | 0.615425 |
| 5 | 11_10787 | 2H | 31 | 0.088 | 0.816582 | 0.576266 |
| 5 | 11_10891 | 2H | 31 | 0.132 | 0.737904 | 0.56349 |
| 5 | 11_10987 | 2H | 31 | 0.248 | 0.564937 | 0.52575 |
| 5 | 11_20864 | 2H | 31.7 | 0.217 | 0.606736 | 0.53722 |
| 5 | 11_11110 | 2H | 32.2 | 0.094 | 0.805378 | 0.576266 |
| 5 | 11_21304 | 2H | 33.7 | 0.021 | 0.952796 | 0.615425 |
| 5 | 11_10525 | 2H | 38 | 1.466 | 0.034198 | 0.16851 |
| 5 | 11_11073 | 2H | 38.5 | 1.381 | 0.041591 | 0.183044 |
| 5 | 11_10178 | 2H | 39.1 | 0.115 | 0.767362 | 0.574629 |
| 5 | 11_10399 | 2H | 39.1 | 1.374 | 0.042267 | 0.183515 |
| 5 | 11_10919 | 2H | 39.1 | 0.027 | 0.939723 | 0.611295 |
| 5 | 12_10296 | 2H | 39.1 | 0.676 | 0.210863 | 0.368343 |
| 5 | 12_30657 | 2H | 39.1 | 0.076 | 0.83946 | 0.580657 |
| 5 | 11_10837 | 2H | 40.5 | 0.618 | 0.240991 | 0.384657 |
| 5 | 11_21153 | 2H | 40.9 | 0.082 | 0.827942 | 0.577884 |
| 5 | 11_10648 | 2H | 41.7 | 0.013 | 0.97051 | 0.619024 |
| 5 | 12_30432 | 2H | 41.7 | 0.092 | 0.809096 | 0.576266 |
| 5 | 11_21338 | 2H | 44.8 | 0.517 | 0.304089 | 0.42242 |
| 5 | 12_30363 | 2H | 45.5 | 0.335 | 0.462381 | 0.495326 |
| 5 | 11_11061 | 2H | 49 | 0.46 | 0.346737 | 0.43613 |
| 5 | 11_10498 | 2H | 49.1 | 0.437 | 0.365595 | 0.445072 |
| 5 | 11_10297 | 2H | 51.8 | 0.055 | 0.881049 | 0.596421 |
| 5 | 11_20674 | 2H | 51.8 | 1.08 | 0.083176 | 0.25325 |
| 5 | 11_10422 | 2H | 52.5 | 1.095 | 0.080353 | 0.245635 |
| 5 | 11_10638 | 2H | 52.5 | 0.423 | 0.377572 | 0.452445 |
| 5 | 11_11302 | 2H | 52.5 | 0.657 | 0.220293 | 0.376586 |
| 5 | 11_20929 | 2H | 52.5 | 1.095 | 0.080353 | 0.245635 |
| 5 | 11_11522 | 2H | 53.5 | 0.191 | 0.644169 | 0.548015 |
| 5 | 12_31474 | 2H | 53.5 | 0.22 | 0.60256 | 0.536969 |
| 5 | 11_10325 | 2H | 55 | 0.486 | 0.326588 | 0.428623 |
| 5 | 11_10733 | 2H | 55 | 0.319 | 0.479733 | 0.501441 |
| 5 | 11_20387 | 2H | 55 | 0.015 | 0.966051 | 0.618016 |
| 5 | 11_21388 | 2H | 55 | 0.216 | 0.608135 | 0.537473 |
| 5 | 12_20234 | 2H | 55 | 0.283 | 0.521195 | 0.505496 |
| 5 | 12_30259 | 2H | 55 | 0.468 | 0.340408 | 0.432398 |
| 5 | 11_11015 | 2H | 55.7 | 0.001 | 0.9977 | 0.62583 |
| 5 | 11_20748 | 2H | 56.3 | 0.264 | 0.544503 | 0.517706 |
| 5 | 11_20891 | 2H | 56.3 | 0.257 | 0.55335 | 0.522191 |
| 5 | 12_30251 | 2H | 56.3 | 0.264 | 0.544503 | 0.517706 |
| 5 | 11_10997 | 2H | 57.5 | 0.134 | 0.734514 | 0.56349 |
| 5 | 12_31288 | 2H | 58.2 | 0.257 | 0.55335 | 0.522191 |
| 5 | 11_20476 | 2H | 58.9 | 0.134 | 0.734514 | 0.56349 |
| 5 | 12_30042 | 2H | 58.9 | 0.41 | 0.389045 | 0.459997 |
| 5 | 11_10909 | 2H | 63.5 | 0.031 | 0.931108 | 0.61021 |
| 5 | 11_10651 | 2H | 68.2 | 2.201 | 0.006295 | 0.088131 |
| 5 | 11_10265 | 2H | 70.5 | 1.967 | 0.010789 | 0.105279 |
| 5 | 11_21258 | 2H | 70.5 | 1.977 | 0.010544 | 0.104653 |
| 5 | 11_21251 | 2H | 71.1 | 0.321 | 0.477529 | 0.501441 |
| 5 | 12_10719 | 2H | 71.1 | 0.472 | 0.337287 | 0.431549 |
| 5 | 11_21205 | 2H | 71.6 | 2.807 | 0.00156 | 0.060261 |
| 5 | 11_10196 | 2H | 78 | 1.327 | 0.047098 | 0.195264 |
| 5 | 11_10818 | 2H | 78 | 1.208 | 0.061944 | 0.224112 |
| 5 | 11_11435 | 2H | 78 | 1.023 | 0.094842 | 0.264027 |
| 5 | 11_20699 | 2H | 78 | 0.628 | 0.235505 | 0.380913 |
| 5 | 12_30696 | 2H | 78 | 1.2 | 0.063096 | 0.225742 |
| 5 | 12_31398 | 2H | 78 | 1.023 | 0.094842 | 0.264027 |
| 5 | 11_10619 | 2H | 82.8 | 1.51 | 0.030903 | 0.163127 |
| 5 | 11_10786 | 2H | 82.8 | 1.455 | 0.035075 | 0.16851 |
| 5 | 11_10823 | 2H | 82.8 | 1.38 | 0.041687 | 0.183044 |
| 5 | 11_11214 | 2H | 82.8 | 1.38 | 0.041687 | 0.183044 |
| 5 | 11_21242 | 2H | 82.8 | 1.491 | 0.032285 | 0.16851 |
| 5 | 11_11100 | 2H | 83.8 | 1.548 | 0.028314 | 0.156293 |
| 5 | 11_10287 | 2H | 85.9 | 2.327 | 0.00471 | 0.078623 |
| 5 | 12_30897 | 2H | 86.6 | 1.975 | 0.010593 | 0.104653 |
| 5 | 12_30901 | 2H | 86.6 | 1.975 | 0.010593 | 0.104653 |
| 5 | 12_31205 | 2H | 86.6 | 0.474 | 0.335738 | 0.430707 |
| 5 | 11_11533 | 2H | 87.3 | 0.404 | 0.394457 | 0.46469 |
| 5 | 11_10475 | 2H | 88.7 | 2.288 | 0.005152 | 0.080276 |
| 5 | 11_21037 | 2H | 88.7 | 2.414 | 0.003855 | 0.075505 |
| 5 | 11_21136 | 2H | 88.7 | 0.455 | 0.350752 | 0.438326 |
| 5 | 11_21245 | 2H | 89.3 | 2.204 | 0.006252 | 0.088131 |
| 5 | 12_31424 | 2H | 89.3 | 2.326 | 0.004721 | 0.078623 |
| 5 | 11_21351 | 2H | 90.1 | 2.303 | 0.004977 | 0.078822 |
| 5 | 12_10969 | 2H | 90.1 | 2.303 | 0.004977 | 0.078822 |
| 5 | 11_20080 | 2H | 95.6 | 1.589 | 0.025763 | 0.153625 |
| 5 | 11_21007 | 2H | 96.2 | 0.525 | 0.298538 | 0.417953 |
| 5 | 11_10138 | 2H | 96.8 | 1.824 | 0.014997 | 0.11778 |
| 5 | 11_11307 | 2H | 96.8 | 0.092 | 0.809096 | 0.576266 |
| 5 | 11_21175 | 2H | 96.8 | 0.092 | 0.809096 | 0.576266 |
| 5 | 12_10649 | 2H | 96.8 | 0.092 | 0.809096 | 0.576266 |
| 5 | 12_30216 | 2H | 96.8 | 0.079 | 0.833681 | 0.579378 |
| 5 | 11_20086 | 2H | 98.6 | 1.701 | 0.019907 | 0.135714 |
| 5 | 11_10398 | 2H | 100.4 | 0.194 | 0.639735 | 0.548015 |
| 5 | 11_10876 | 2H | 100.4 | 0.516 | 0.30479 | 0.42242 |
| 5 | 11_11250 | 2H | 100.4 | 0.383 | 0.414 | 0.476667 |
| 5 | 11_10900 | 2H | 101.8 | 0.366 | 0.430527 | 0.483574 |
| 5 | 12_30480 | 2H | 102.5 | 0.29 | 0.512861 | 0.501441 |
| 5 | 11_21340 | 2H | 103.7 | 0.238 | 0.578096 | 0.527723 |
| 5 | 11_10630 | 2H | 105.8 | 0.238 | 0.578096 | 0.527723 |
| 5 | 11_11323 | 2H | 106.5 | 0.638 | 0.230144 | 0.38083 |
| 5 | 12_30049 | 2H | 106.5 | 0.636 | 0.231207 | 0.38083 |
| 5 | 12_30555 | 2H | 106.5 | 0.638 | 0.230144 | 0.38083 |
| 5 | 11_11094 | 2H | 108.6 | 2.36 | 0.004365 | 0.076668 |
| 5 | 11_11480 | 2H | 108.6 | 0.594 | 0.254683 | 0.396173 |
| 5 | 11_20064 | 2H | 112.9 | 0.091 | 0.810961 | 0.576266 |
| 5 | 11_10128 | 2H | 113.5 | 1.882 | 0.013122 | 0.111192 |
| 5 | 11_10731 | 2H | 113.5 | 0.188 | 0.648634 | 0.548015 |
| 5 | 11_10988 | 2H | 113.5 | 0.127 | 0.746449 | 0.567771 |
| 5 | 11_10989 | 2H | 113.5 | 0.127 | 0.746449 | 0.567771 |
| 5 | 11_10990 | 2H | 113.5 | 0.188 | 0.648634 | 0.548015 |
| 5 | 11_11043 | 2H | 113.5 | 0.188 | 0.648634 | 0.548015 |
| 5 | 11_11118 | 2H | 113.5 | 0.703 | 0.198153 | 0.356453 |
| 5 | 11_21238 | 2H | 113.5 | 0.188 | 0.648634 | 0.548015 |
| 5 | 12_31402 | 2H | 113.5 | 0.127 | 0.746449 | 0.567771 |
| 5 | 11_10429 | 2H | 115.1 | 1.637 | 0.023067 | 0.143762 |
| 5 | 11_11236 | 2H | 115.8 | 0.678 | 0.209894 | 0.367314 |
| 5 | 11_10538 | 2H | 116.5 | 0.605 | 0.248313 | 0.390033 |
| 5 | 11_10707 | 2H | 116.5 | 0.536 | 0.291072 | 0.411677 |
| 5 | 11_20182 | 2H | 116.5 | 1.278 | 0.052723 | 0.203722 |
| 5 | 12_30459 | 2H | 116.5 | 0.448 | 0.356451 | 0.440886 |
| 5 | 12_31095 | 2H | 116.5 | 1.191 | 0.064417 | 0.226138 |
| 5 | 11_10404 | 2H | 117.2 | 0.315 | 0.484172 | 0.501441 |
| 5 | 11_10916 | 2H | 117.9 | 0.791 | 0.161808 | 0.313868 |
| 5 | 12_30598 | 2H | 119.3 | 1.132 | 0.07379 | 0.236816 |
| 5 | 12_31264 | 2H | 119.3 | 1.07 | 0.085114 | 0.253372 |
| 5 | 11_20141 | 2H | 121.5 | 1.024 | 0.094624 | 0.264027 |
| 5 | 11_21315 | 2H | 121.5 | 1.311 | 0.048865 | 0.198335 |
| 5 | 12_30097 | 2H | 121.5 | 1.026 | 0.094189 | 0.264027 |
| 5 | 12_30152 | 2H | 122.2 | 0.136 | 0.731139 | 0.562773 |
| 5 | 12_30636 | 2H | 122.2 | 0.747 | 0.179061 | 0.332001 |
| 5 | 11_10446 | 2H | 125.5 | 0.091 | 0.810961 | 0.576266 |
| 5 | 11_21370 | 2H | 125.5 | 0.602 | 0.250035 | 0.3921 |
| 5 | 12_31100 | 2H | 125.5 | 1.121 | 0.075683 | 0.238692 |
| 5 | 11_21406 | 2H | 126.4 | 1.121 | 0.075683 | 0.238692 |
| 5 | 11_11486 | 2H | 127.1 | 0.508 | 0.310456 | 0.424187 |
| 5 | 11_21459 | 2H | 127.1 | 0.026 | 0.94189 | 0.612291 |
| 5 | 12_20183 | 2H | 127.6 | 2.083 | 0.00826 | 0.091305 |
| 5 | 12_30310 | 2H | 127.6 | 0.874 | 0.13366 | 0.298186 |
| 5 | 12_30695 | 2H | 127.6 | 0.028 | 0.937562 | 0.611123 |
| 5 | 11_10656 | 2H | 128.3 | 2.14 | 0.007244 | 0.090761 |
| 5 | 11_21088 | 2H | 128.3 | 2.131 | 0.007396 | 0.090761 |
| 5 | 11_10065 | 2H | 130 | 0.488 | 0.325087 | 0.428623 |
| 5 | 11_10383 | 2H | 130 | 0.047 | 0.897429 | 0.601607 |
| 5 | 11_20215 | 2H | 130 | 0.488 | 0.325087 | 0.428623 |
| 5 | 12_30942 | 2H | 130 | 0.485 | 0.327341 | 0.428623 |
| 5 | 12_31268 | 2H | 130 | 0.491 | 0.322849 | 0.428623 |
| 5 | 11_10376 | 2H | 131.8 | 0.006 | 0.98628 | 0.622303 |
| 5 | 11_20895 | 2H | 131.8 | 0.629 | 0.234963 | 0.38083 |
| 5 | 12_10579 | 2H | 132.5 | 0.589 | 0.257632 | 0.398123 |
| 5 | 11_20715 | 2H | 133.9 | 0.024 | 0.946237 | 0.614291 |
| 5 | 11_20590 | 2H | 137.5 | 0.449 | 0.355631 | 0.440886 |
| 5 | 11_21274 | 2H | 137.5 | 0.016 | 0.963829 | 0.617004 |
| 5 | 12_31461 | 2H | 138.9 | 0.025 | 0.944061 | 0.61329 |
| 5 | 11_10551 | 2H | 139.7 | 0.082 | 0.827942 | 0.577884 |
| 5 | 11_10826 | 2H | 139.7 | 0.02 | 0.954993 | 0.615425 |
| 5 | 11_11262 | 2H | 139.7 | 0.004 | 0.990832 | 0.62314 |
| 5 | 11_20494 | 2H | 139.7 | 0.013 | 0.97051 | 0.619024 |
| 5 | 12_30352 | 2H | 139.7 | 0.01 | 0.977237 | 0.619024 |
| 5 | 12_30914 | 2H | 139.7 | 0.035 | 0.922571 | 0.60833 |
| 5 | 12_31209 | 2H | 139.7 | 0.013 | 0.97051 | 0.619024 |
| 5 | 11_10566 | 2H | 140.3 | 0.197 | 0.635331 | 0.548015 |
| 5 | 11_10315 | 2H | 141.3 | 0.375 | 0.421697 | 0.479246 |
| 5 | 11_11023 | 2H | 141.3 | 1.127 | 0.074645 | 0.238525 |
| 5 | 12_10447 | 2H | 141.3 | 0.533 | 0.293089 | 0.412117 |
| 5 | 12_10766 | 2H | 141.3 | 0.54 | 0.288403 | 0.4091 |
| 5 | 11_21250 | 2H | 144.3 | 1.192 | 0.064269 | 0.226138 |
| 5 | 12_10487 | 2H | 144.3 | 0.533 | 0.293089 | 0.412117 |
| 5 | 11_11380 | 2H | 145 | 0.388 | 0.409261 | 0.473468 |
| 5 | 11_20994 | 2H | 147.1 | 0.29 | 0.512861 | 0.501441 |
| 5 | 11_21346 | 2H | 147.1 | 0.115 | 0.767362 | 0.574629 |
| 5 | 11_20293 | 2H | 147.9 | 0.079 | 0.833681 | 0.579378 |
| 5 | 11_10791 | 2H | 150.7 | 0.109 | 0.778037 | 0.575737 |
| 5 | 12_30823 | 2H | 150.7 | 0.38 | 0.416869 | 0.477069 |
| 5 | 11_10072 | 2H | 151.4 | 0.053 | 0.885116 | 0.597917 |
| 5 | 12_31527 | 2H | 151.4 | 0.339 | 0.458142 | 0.494486 |
| 5 | 12_31300 | 2H | 152.8 | 0.394 | 0.403645 | 0.471489 |
| 5 | 12_31506 | 2H | 152.8 | 0.144 | 0.717794 | 0.561313 |
| 5 | 12_31180 | 2H | 155.3 | 0.346 | 0.450817 | 0.490415 |
| 5 | 11_10085 | 2H | 156.7 | 0.067 | 0.857038 | 0.58633 |
| 5 | 11_20561 | 2H | 156.7 | 0.144 | 0.717794 | 0.561313 |
| 5 | 11_20681 | 2H | 156.7 | 0.156 | 0.698232 | 0.559354 |
| 5 | 12_11050 | 2H | 156.7 | 0.156 | 0.698232 | 0.559354 |
| 5 | 12_30378 | 2H | 158.9 | 0.038 | 0.916221 | 0.607043 |
| 5 | 11_11411 | 3H | 0 | 0.877 | 0.132739 | 0.29682 |
| 5 | 11_20222 | 3H | 0 | 0.75 | 0.177828 | 0.332001 |
| 5 | 11_20952 | 3H | 0 | 0.908 | 0.123595 | 0.295467 |
| 5 | 12_31428 | 3H | 0 | 0.779 | 0.166341 | 0.317561 |
| 5 | 11_20797 | 3H | 2.3 | 0.845 | 0.142889 | 0.299306 |
| 5 | 11_20159 | 3H | 2.9 | 0.136 | 0.731139 | 0.562773 |
| 5 | 12_10103 | 3H | 2.9 | 0.475 | 0.334965 | 0.430707 |
| 5 | 12_31448 | 3H | 2.9 | 0.045 | 0.901571 | 0.602294 |
| 5 | 11_11453 | 3H | 6 | 0.005 | 0.988553 | 0.622518 |
| 5 | 11_20252 | 3H | 6 | 0.171 | 0.674528 | 0.553663 |
| 5 | 12_31409 | 3H | 6.7 | 0.133 | 0.736207 | 0.56349 |
| 5 | 11_21190 | 3H | 8.2 | 0.497 | 0.31842 | 0.427213 |
| 5 | 11_21398 | 3H | 8.2 | 0.346 | 0.450817 | 0.490415 |
| 5 | 11_21027 | 3H | 8.9 | 2.536 | 0.002911 | 0.07023 |
| 5 | 11_20976 | 3H | 9.6 | 0.022 | 0.950605 | 0.615425 |
| 5 | 12_30818 | 3H | 9.6 | 0.124 | 0.751623 | 0.569465 |
| 5 | 11_10112 | 3H | 10.8 | 1.16 | 0.069183 | 0.228092 |
| 5 | 11_10886 | 3H | 10.8 | 0.006 | 0.98628 | 0.622303 |
| 5 | 11_20595 | 3H | 12.5 | 0.197 | 0.635331 | 0.548015 |
| 5 | 12_30113 | 3H | 15.6 | 0.882 | 0.13122 | 0.295467 |
| 5 | 11_20172 | 3H | 16.3 | 0.644 | 0.226987 | 0.38083 |
| 5 | 11_10565 | 3H | 19.1 | 0.2 | 0.630957 | 0.548015 |
| 5 | 11_20742 | 3H | 19.1 | 0.112 | 0.772681 | 0.575737 |
| 5 | 11_20982 | 3H | 22.7 | 0.146 | 0.714496 | 0.561313 |
| 5 | 12_30192 | 3H | 23.4 | 0.164 | 0.685488 | 0.553663 |
| 5 | 11_20794 | 3H | 26.9 | 0.045 | 0.901571 | 0.602294 |
| 5 | 12_30284 | 3H | 28.4 | 0.187 | 0.65013 | 0.548015 |
| 5 | 11_20607 | 3H | 32.8 | 0.634 | 0.232274 | 0.38083 |
| 5 | 12_30571 | 3H | 32.8 | 0.184 | 0.654636 | 0.54909 |
| 5 | 11_10672 | 3H | 37.2 | 0.391 | 0.406443 | 0.473041 |
| 5 | 12_30925 | 3H | 37.2 | 0.176 | 0.666807 | 0.551014 |
| 5 | 12_10968 | 3H | 38.7 | 2.435 | 0.003673 | 0.075488 |
| 5 | 11_10081 | 3H | 39.5 | 0.191 | 0.644169 | 0.548015 |
| 5 | 11_10710 | 3H | 39.5 | 0.176 | 0.666807 | 0.551014 |
| 5 | 11_10825 | 3H | 39.5 | 0.492 | 0.322107 | 0.428588 |
| 5 | 11_20410 | 3H | 39.5 | 0.492 | 0.322107 | 0.428588 |
| 5 | 12_30953 | 3H | 41 | 0.588 | 0.258226 | 0.398123 |
| 5 | 12_31159 | 3H | 41 | 0.137 | 0.729458 | 0.562773 |
| 5 | 11_10863 | 3H | 41.7 | 0.188 | 0.648634 | 0.548015 |
| 5 | 11_20193 | 3H | 42.1 | 1.621 | 0.023933 | 0.148201 |
| 5 | 11_21533 | 3H | 43.2 | 1.649 | 0.022439 | 0.141673 |
| 5 | 11_11002 | 3H | 44 | 1.667 | 0.021528 | 0.13957 |
| 5 | 12_30913 | 3H | 44.8 | 1.48 | 0.033113 | 0.16851 |
| 5 | 12_30785 | 3H | 45.5 | 0.499 | 0.316957 | 0.427213 |
| 5 | 12_31475 | 3H | 45.5 | 0.294 | 0.508159 | 0.501441 |
| 5 | 11_10601 | 3H | 46.3 | 1.265 | 0.054325 | 0.206606 |
| 5 | 12_30064 | 3H | 46.3 | 2.637 | 0.002307 | 0.065601 |
| 5 | 12_30609 | 3H | 48.6 | 0.328 | 0.469894 | 0.498811 |
| 5 | 12_31122 | 3H | 48.6 | 0.299 | 0.502343 | 0.501441 |
| 5 | 11_11313 | 3H | 51.7 | 0.658 | 0.219786 | 0.376442 |
| 5 | 11_21197 | 3H | 51.7 | 0.148 | 0.711214 | 0.560385 |
| 5 | 11_11086 | 3H | 53.3 | 0.783 | 0.164816 | 0.317156 |
| 5 | 12_30618 | 3H | 53.3 | 0.786 | 0.163682 | 0.316233 |
| 5 | 11_10137 | 3H | 54.4 | 0.883 | 0.130918 | 0.295467 |
| 5 | 11_10328 | 3H | 54.4 | 0.883 | 0.130918 | 0.295467 |
| 5 | 11_11501 | 3H | 54.4 | 0.883 | 0.130918 | 0.295467 |
| 5 | 11_20970 | 3H | 54.4 | 0.883 | 0.130918 | 0.295467 |
| 5 | 12_30039 | 3H | 54.4 | 0.883 | 0.130918 | 0.295467 |
| 5 | 12_30130 | 3H | 54.4 | 0.883 | 0.130918 | 0.295467 |
| 5 | 12_30318 | 3H | 54.4 | 0.883 | 0.130918 | 0.295467 |
| 5 | 12_31012 | 3H | 55.6 | 0.846 | 0.142561 | 0.299306 |
| 5 | 12_31502 | 3H | 55.6 | 0.883 | 0.130918 | 0.295467 |
| 5 | 11_10008 | 3H | 56.4 | 0.883 | 0.130918 | 0.295467 |
| 5 | 11_10011 | 3H | 56.4 | 0.85 | 0.141254 | 0.299306 |
| 5 | 11_10456 | 3H | 56.4 | 0.883 | 0.130918 | 0.295467 |
| 5 | 11_10620 | 3H | 56.4 | 0.961 | 0.109396 | 0.282557 |
| 5 | 11_10925 | 3H | 56.4 | 0.961 | 0.109396 | 0.282557 |
| 5 | 11_10926 | 3H | 56.4 | 0.099 | 0.796159 | 0.575737 |
| 5 | 11_10966 | 3H | 56.4 | 0.961 | 0.109396 | 0.282557 |
| 5 | 11_11124 | 3H | 56.4 | 0.883 | 0.130918 | 0.295467 |
| 5 | 11_11125 | 3H | 56.4 | 0.846 | 0.142561 | 0.299306 |
| 5 | 11_11283 | 3H | 56.4 | 0.846 | 0.142561 | 0.299306 |
| 5 | 11_11337 | 3H | 56.4 | 0.883 | 0.130918 | 0.295467 |
| 5 | 11_11530 | 3H | 56.4 | 0.846 | 0.142561 | 0.299306 |
| 5 | 11_20002 | 3H | 56.4 | 0.961 | 0.109396 | 0.282557 |
| 5 | 11_20288 | 3H | 56.4 | 0.846 | 0.142561 | 0.299306 |
| 5 | 11_20333 | 3H | 56.4 | 0.977 | 0.105439 | 0.278289 |
| 5 | 11_20428 | 3H | 56.4 | 0.883 | 0.130918 | 0.295467 |
| 5 | 11_20439 | 3H | 56.4 | 0.883 | 0.130918 | 0.295467 |
| 5 | 11_20486 | 3H | 56.4 | 0.961 | 0.109396 | 0.282557 |
| 5 | 11_20583 | 3H | 56.4 | 0.846 | 0.142561 | 0.299306 |
| 5 | 11_20796 | 3H | 56.4 | 0.883 | 0.130918 | 0.295467 |
| 5 | 11_20801 | 3H | 56.4 | 0.846 | 0.142561 | 0.299306 |
| 5 | 11_20856 | 3H | 56.4 | 0.883 | 0.130918 | 0.295467 |
| 5 | 11_21062 | 3H | 56.4 | 0.883 | 0.130918 | 0.295467 |
| 5 | 11_21147 | 3H | 56.4 | 0.883 | 0.130918 | 0.295467 |
| 5 | 11_21435 | 3H | 56.4 | 0.846 | 0.142561 | 0.299306 |
| 5 | 11_21472 | 3H | 56.4 | 0.846 | 0.142561 | 0.299306 |
| 5 | 12_31017 | 3H | 56.4 | 0.85 | 0.141254 | 0.299306 |
| 5 | 12_31214 | 3H | 56.4 | 0.846 | 0.142561 | 0.299306 |
| 5 | 12_31281 | 3H | 56.4 | 0.154 | 0.701455 | 0.559354 |
| 5 | 12_31368 | 3H | 56.4 | 0.099 | 0.796159 | 0.575737 |
| 5 | 11_20444 | 3H | 57.1 | 0.029 | 0.935406 | 0.611123 |
| 5 | 11_10225 | 3H | 58 | 1.549 | 0.028249 | 0.156293 |
| 5 | 11_11401 | 3H | 58 | 0.099 | 0.796159 | 0.575737 |
| 5 | 11_11016 | 3H | 58.6 | 2.115 | 0.007674 | 0.090761 |
| 5 | 11_20276 | 3H | 58.6 | 2.115 | 0.007674 | 0.090761 |
| 5 | 12_31011 | 3H | 58.6 | 2.115 | 0.007674 | 0.090761 |
| 5 | 12_31393 | 3H | 58.6 | 2.115 | 0.007674 | 0.090761 |
| 5 | 11_10158 | 3H | 59.9 | 0.747 | 0.179061 | 0.332001 |
| 5 | 11_10373 | 3H | 59.9 | 0.747 | 0.179061 | 0.332001 |
| 5 | 11_10653 | 3H | 59.9 | 2.115 | 0.007674 | 0.090761 |
| 5 | 11_21511 | 3H | 59.9 | 1.549 | 0.028249 | 0.156293 |
| 5 | 12_30829 | 3H | 59.9 | 0.747 | 0.179061 | 0.332001 |
| 5 | 11_10728 | 3H | 63 | 0.575 | 0.266073 | 0.39847 |
| 5 | 11_10281 | 3H | 64.2 | 2.82 | 0.001514 | 0.060261 |
| 5 | 11_11191 | 3H | 64.2 | 0.708 | 0.195885 | 0.35369 |
| 5 | 11_21120 | 3H | 64.2 | 0.735 | 0.184077 | 0.338702 |
| 5 | 11_10335 | 3H | 65.5 | 0.494 | 0.320627 | 0.427798 |
| 5 | 11_11391 | 3H | 65.5 | 0.368 | 0.428549 | 0.482492 |
| 5 | 11_20704 | 3H | 65.5 | 0.836 | 0.145881 | 0.299306 |
| 5 | 11_21502 | 3H | 65.5 | 0.548 | 0.283139 | 0.407012 |
| 5 | 11_21305 | 3H | 67.6 | 0.351 | 0.445656 | 0.4881 |
| 5 | 11_20931 | 3H | 68.3 | 0.516 | 0.30479 | 0.42242 |
| 5 | 12_30616 | 3H | 68.3 | 0.629 | 0.234963 | 0.38083 |
| 5 | 12_30788 | 3H | 68.3 | 0.634 | 0.232274 | 0.38083 |
| 5 | 12_31153 | 3H | 68.3 | 0.715 | 0.192753 | 0.351319 |
| 5 | 11_10172 | 3H | 69.6 | 0.495 | 0.31989 | 0.427404 |
| 5 | 11_11394 | 3H | 69.6 | 0.273 | 0.533335 | 0.51366 |
| 5 | 11_20017 | 3H | 69.6 | 0.508 | 0.310456 | 0.424187 |
| 5 | 12_31242 | 3H | 69.6 | 0.2 | 0.630957 | 0.548015 |
| 5 | 11_11314 | 3H | 70.2 | 0.176 | 0.666807 | 0.551014 |
| 5 | 11_11241 | 3H | 70.7 | 2.08 | 0.008318 | 0.091305 |
| 5 | 11_20273 | 3H | 70.7 | 0.164 | 0.685488 | 0.553663 |
| 5 | 11_20877 | 3H | 70.7 | 0.164 | 0.685488 | 0.553663 |
| 5 | 12_31323 | 3H | 70.7 | 0.164 | 0.685488 | 0.553663 |
| 5 | 12_30754 | 3H | 71.5 | 1.649 | 0.022439 | 0.141673 |
| 5 | 11_20694 | 3H | 72.3 | 0.02 | 0.954993 | 0.615425 |
| 5 | 12_31529 | 3H | 72.3 | 0.531 | 0.294442 | 0.413417 |
| 5 | 11_10350 | 3H | 73.5 | 2.1 | 0.007943 | 0.091108 |
| 5 | 12_31356 | 3H | 73.5 | 2.025 | 0.009441 | 0.100044 |
| 5 | 11_20521 | 3H | 74.2 | 0.672 | 0.212814 | 0.370597 |
| 5 | 12_30399 | 3H | 74.8 | 0.835 | 0.146218 | 0.299306 |
| 5 | 11_20566 | 3H | 75.5 | 2.352 | 0.004446 | 0.076699 |
| 5 | 11_10683 | 3H | 76.2 | 1.187 | 0.065013 | 0.226138 |
| 5 | 11_20695 | 3H | 76.2 | 1.187 | 0.065013 | 0.226138 |
| 5 | 11_20778 | 3H | 76.2 | 0.176 | 0.666807 | 0.551014 |
| 5 | 12_10609 | 3H | 76.2 | 1.187 | 0.065013 | 0.226138 |
| 5 | 12_30743 | 3H | 76.2 | 0.176 | 0.666807 | 0.551014 |
| 5 | 12_31346 | 3H | 77 | 1.183 | 0.065615 | 0.22637 |
| 5 | 11_10047 | 3H | 78.5 | 0.283 | 0.521195 | 0.505496 |
| 5 | 11_20362 | 3H | 78.5 | 0.302 | 0.498885 | 0.501441 |
| 5 | 11_20597 | 3H | 78.5 | 0.16 | 0.691831 | 0.557388 |
| 5 | 12_11454 | 3H | 78.5 | 0.922 | 0.119674 | 0.295467 |
| 5 | 11_20115 | 3H | 80.9 | 2.392 | 0.004055 | 0.075505 |
| 5 | 12_30170 | 3H | 80.9 | 2.439 | 0.003639 | 0.075488 |
| 5 | 11_20093 | 3H | 81.7 | 0.25 | 0.562341 | 0.524345 |
| 5 | 11_21358 | 3H | 81.7 | 3 | 0.001 | 0.0483 |
| 5 | 12_30677 | 3H | 81.7 | 2.508 | 0.003105 | 0.07023 |
| 5 | 12_31262 | 3H | 81.7 | 0.067 | 0.857038 | 0.58633 |
| 5 | 12_30278 | 3H | 83.2 | 0.005 | 0.988553 | 0.622518 |
| 5 | 11_20063 | 3H | 86 | 0.322 | 0.476431 | 0.501441 |
| 5 | 11_10628 | 3H | 87.2 | 0.291 | 0.511682 | 0.501441 |
| 5 | 11_21348 | 3H | 87.2 | 0.782 | 0.165196 | 0.317255 |
| 5 | 11_20136 | 3H | 88.8 | 0.068 | 0.855067 | 0.585811 |
| 5 | 11_21294 | 3H | 88.8 | 0.21 | 0.616595 | 0.542469 |
| 5 | 12_10134 | 3H | 89.3 | 0.149 | 0.709578 | 0.559553 |
| 5 | 12_30663 | 3H | 89.3 | 0.23 | 0.588844 | 0.530618 |
| 5 | 11_20659 | 3H | 91.2 | 1.562 | 0.027416 | 0.156293 |
| 5 | 12_10583 | 3H | 91.9 | 1.415 | 0.038459 | 0.173996 |
| 5 | 11_10747 | 3H | 93.4 | 0.92 | 0.120226 | 0.295467 |
| 5 | 11_11021 | 3H | 93.4 | 0.965 | 0.108393 | 0.282557 |
| 5 | 12_31367 | 3H | 95.4 | 0.465 | 0.342768 | 0.432828 |
| 5 | 12_30250 | 3H | 96.2 | 1.869 | 0.013521 | 0.112595 |
| 5 | 11_20130 | 3H | 98.5 | 1.132 | 0.07379 | 0.236816 |
| 5 | 11_20628 | 3H | 98.5 | 0.361 | 0.435512 | 0.484683 |
| 5 | 11_10515 | 3H | 99.9 | 1.283 | 0.052119 | 0.203014 |
| 5 | 11_10184 | 3H | 100.7 | 1.262 | 0.054702 | 0.207222 |
| 5 | 11_21381 | 3H | 102.2 | 1.185 | 0.065313 | 0.226138 |
| 5 | 11_21517 | 3H | 103 | 1.921 | 0.011995 | 0.106795 |
| 5 | 12_10344 | 3H | 104.5 | 0.107 | 0.781628 | 0.575737 |
| 5 | 12_10662 | 3H | 104.5 | 1.712 | 0.019409 | 0.135714 |
| 5 | 12_30342 | 3H | 104.5 | 0.132 | 0.737904 | 0.56349 |
| 5 | 11_21513 | 3H | 109.1 | 0.905 | 0.124452 | 0.295467 |
| 5 | 11_20023 | 3H | 111.4 | 1.932 | 0.011695 | 0.105583 |
| 5 | 11_21161 | 3H | 111.4 | 1.932 | 0.011695 | 0.105583 |
| 5 | 11_21212 | 3H | 111.4 | 1.974 | 0.010617 | 0.104653 |
| 5 | 12_30423 | 3H | 111.4 | 0.524 | 0.299227 | 0.418311 |
| 5 | 11_10312 | 3H | 114 | 0.929 | 0.117761 | 0.295467 |
| 5 | 11_10753 | 3H | 114 | 0.978 | 0.105196 | 0.278289 |
| 5 | 11_11503 | 3H | 114 | 0.376 | 0.420727 | 0.478706 |
| 5 | 11_20168 | 3H | 114 | 0.36 | 0.436516 | 0.484683 |
| 5 | 11_20523 | 3H | 114 | 0.594 | 0.254683 | 0.396173 |
| 5 | 11_21277 | 3H | 114 | 0.566 | 0.271644 | 0.400977 |
| 5 | 12_10100 | 3H | 114 | 0.36 | 0.436516 | 0.484683 |
| 5 | 12_30276 | 3H | 114 | 0.415 | 0.384592 | 0.456407 |
| 5 | 12_30375 | 3H | 114.8 | 0.415 | 0.384592 | 0.456407 |
| 5 | 12_31329 | 3H | 115.5 | 0.269 | 0.53827 | 0.515842 |
| 5 | 11_10584 | 3H | 117.1 | 2.277 | 0.005284 | 0.081028 |
| 5 | 12_30927 | 3H | 117.1 | 2.735 | 0.001841 | 0.060341 |
| 5 | 11_11330 | 3H | 120.6 | 1.934 | 0.011641 | 0.105583 |
| 5 | 11_10918 | 3H | 123.7 | 0.317 | 0.481948 | 0.501441 |
| 5 | 11_21405 | 3H | 123.7 | 0.361 | 0.435512 | 0.484683 |
| 5 | 11_11172 | 3H | 126.3 | 0.802 | 0.157761 | 0.309382 |
| 5 | 12_31525 | 3H | 126.3 | 0.914 | 0.121899 | 0.295467 |
| 5 | 11_20650 | 3H | 127.1 | 0.497 | 0.31842 | 0.427213 |
| 5 | 12_30096 | 3H | 127.1 | 0.616 | 0.242103 | 0.384657 |
| 5 | 12_31238 | 3H | 127.1 | 0.497 | 0.31842 | 0.427213 |
| 5 | 12_30081 | 3H | 128.6 | 0.062 | 0.866962 | 0.589363 |
| 5 | 11_10280 | 3H | 130.2 | 0.182 | 0.657658 | 0.54909 |
| 5 | 11_11141 | 3H | 130.8 | 0.182 | 0.657658 | 0.54909 |
| 5 | 11_20612 | 3H | 131.6 | 1.429 | 0.037239 | 0.173996 |
| 5 | 12_30973 | 3H | 133.1 | 0.302 | 0.498885 | 0.501441 |
| 5 | 11_20527 | 3H | 134.3 | 0.633 | 0.232809 | 0.38083 |
| 5 | 11_10381 | 3H | 136.7 | 0.498 | 0.317687 | 0.427213 |
| 5 | 11_11127 | 3H | 136.7 | 0.381 | 0.415911 | 0.477069 |
| 5 | 11_21428 | 3H | 136.7 | 0.39 | 0.40738 | 0.473468 |
| 5 | 12_10122 | 3H | 136.7 | 0.414 | 0.385478 | 0.456898 |
| 5 | 11_20920 | 3H | 140.9 | 0.429 | 0.372392 | 0.449101 |
| 5 | 11_21427 | 3H | 141.5 | 0.785 | 0.164059 | 0.316329 |
| 5 | 11_10631 | 3H | 144.6 | 0.351 | 0.445656 | 0.4881 |
| 5 | 11_21266 | 3H | 148.9 | 0.425 | 0.375837 | 0.452445 |
| 5 | 11_21272 | 3H | 150.4 | 0.775 | 0.16788 | 0.319237 |
| 5 | 12_21376 | 3H | 151.2 | 1.149 | 0.070958 | 0.230792 |
| 5 | 12_30841 | 3H | 151.2 | 0.994 | 0.101391 | 0.273586 |
| 5 | 12_31500 | 3H | 152 | 0.994 | 0.101391 | 0.273586 |
| 5 | 11_20155 | 3H | 155.1 | 0.81 | 0.154882 | 0.309349 |
| 5 | 11_11436 | 3H | 155.8 | 0.554 | 0.279254 | 0.404437 |
| 5 | 11_10646 | 3H | 162.2 | 0.514 | 0.306196 | 0.422921 |
| 5 | 11_10702 | 3H | 162.2 | 0.302 | 0.498885 | 0.501441 |
| 5 | 11_21008 | 3H | 162.2 | 0.018 | 0.959401 | 0.615801 |
| 5 | 12_30271 | 3H | 162.2 | 0.074 | 0.843335 | 0.580657 |
| 5 | 12_30767 | 3H | 162.2 | 0.053 | 0.885116 | 0.597917 |
| 5 | 11_20605 | 3H | 166.2 | 0.038 | 0.916221 | 0.607043 |
| 5 | 11_10681 | 3H | 167.8 | 0.81 | 0.154882 | 0.309349 |
| 5 | 11_11410 | 3H | 167.8 | 0.385 | 0.412098 | 0.475043 |
| 5 | 12_31388 | 3H | 167.8 | 0.212 | 0.613762 | 0.540962 |
| 5 | 11_10694 | 3H | 168.4 | 0.201 | 0.629506 | 0.548015 |
| 5 | 11_20057 | 3H | 168.4 | 0.09 | 0.812831 | 0.576266 |
| 5 | 11_21267 | 3H | 168.4 | 0.028 | 0.937562 | 0.611123 |
| 5 | 12_10014 | 3H | 168.4 | 0.637 | 0.230675 | 0.38083 |
| 5 | 12_30736 | 3H | 168.4 | 0.667 | 0.215278 | 0.372019 |
| 5 | 11_11516 | 3H | 169.3 | 0.687 | 0.205589 | 0.364402 |
| 5 | 11_10283 | 3H | 173.2 | 0.079 | 0.833681 | 0.579378 |
| 5 | 11_10343 | 3H | 173.2 | 0.864 | 0.136773 | 0.299306 |
| 5 | 11_21362 | 3H | 173.2 | 0.048 | 0.895365 | 0.601607 |
| 5 | 12_20345 | 3H | 173.2 | 0.079 | 0.833681 | 0.579378 |
| 5 | 12_30135 | 3H | 173.2 | 1.317 | 0.048195 | 0.198111 |
| 5 | 11_10028 | 4H | 0 | 0.218 | 0.605341 | 0.536969 |
| 5 | 11_10247 | 4H | 0 | 2.108 | 0.007798 | 0.090761 |
| 5 | 11_20668 | 4H | 0 | 0.214 | 0.610942 | 0.538968 |
| 5 | 11_20740 | 4H | 0 | 0.682 | 0.20797 | 0.366779 |
| 5 | 12_30764 | 4H | 0.7 | 0.292 | 0.510505 | 0.501441 |
| 5 | 12_31324 | 4H | 0.7 | 0.345 | 0.451856 | 0.490441 |
| 5 | 11_20145 | 4H | 1.6 | 0.299 | 0.502343 | 0.501441 |
| 5 | 11_10409 | 4H | 3.7 | 0.183 | 0.656145 | 0.54909 |
| 5 | 11_21056 | 4H | 3.7 | 0.241 | 0.574117 | 0.527183 |
| 5 | 11_21228 | 4H | 3.7 | 0.28 | 0.524808 | 0.50798 |
| 5 | 11_11345 | 4H | 5.5 | 0.138 | 0.72778 | 0.562773 |
| 5 | 11_10319 | 4H | 8.2 | 0.323 | 0.475335 | 0.501441 |
| 5 | 12_31458 | 4H | 12 | 0.223 | 0.598412 | 0.535246 |
| 5 | 12_30540 | 4H | 15.8 | 0.184 | 0.654636 | 0.54909 |
| 5 | 11_10113 | 4H | 19.5 | 1.108 | 0.077983 | 0.243792 |
| 5 | 11_10738 | 4H | 19.5 | 1.292 | 0.051051 | 0.203014 |
| 5 | 11_10223 | 4H | 20.1 | 0.641 | 0.22856 | 0.38083 |
| 5 | 11_20557 | 4H | 20.1 | 0.388 | 0.409261 | 0.473468 |
| 5 | 11_21359 | 4H | 20.1 | 0.388 | 0.409261 | 0.473468 |
| 5 | 11_10221 | 4H | 21.6 | 0.995 | 0.101158 | 0.273586 |
| 5 | 11_21385 | 4H | 23.1 | 1.147 | 0.071285 | 0.231079 |
| 5 | 11_10132 | 4H | 24.6 | 0.479 | 0.331895 | 0.428623 |
| 5 | 11_20210 | 4H | 24.6 | 0.479 | 0.331895 | 0.428623 |
| 5 | 12_10395 | 4H | 24.6 | 0.296 | 0.505825 | 0.501441 |
| 5 | 11_10031 | 4H | 28.4 | 1.503 | 0.031405 | 0.164877 |
| 5 | 11_21374 | 4H | 28.4 | 0.031 | 0.931108 | 0.61021 |
| 5 | 11_21122 | 4H | 33.4 | 0.173 | 0.671429 | 0.55247 |
| 5 | 11_20411 | 4H | 36.4 | 0.031 | 0.931108 | 0.61021 |
| 5 | 11_11180 | 4H | 41 | 0.65 | 0.223872 | 0.379404 |
| 5 | 12_30328 | 4H | 41 | 1.558 | 0.027669 | 0.156293 |
| 5 | 11_10668 | 4H | 44.9 | 0.901 | 0.125603 | 0.295467 |
| 5 | 11_10793 | 4H | 44.9 | 0.908 | 0.123595 | 0.295467 |
| 5 | 11_20939 | 4H | 46.4 | 0.908 | 0.123595 | 0.295467 |
| 5 | 11_21490 | 4H | 46.4 | 0.908 | 0.123595 | 0.295467 |
| 5 | 11_10261 | 4H | 48.5 | 2.411 | 0.003882 | 0.075505 |
| 5 | 11_10432 | 4H | 48.5 | 1.688 | 0.020512 | 0.135714 |
| 5 | 11_10577 | 4H | 48.5 | 1.688 | 0.020512 | 0.135714 |
| 5 | 11_10667 | 4H | 48.5 | 2.021 | 0.009528 | 0.100044 |
| 5 | 11_10756 | 4H | 48.5 | 1.688 | 0.020512 | 0.135714 |
| 5 | 11_10942 | 4H | 48.5 | 0.218 | 0.605341 | 0.536969 |
| 5 | 11_20782 | 4H | 48.5 | 1.688 | 0.020512 | 0.135714 |
| 5 | 11_21071 | 4H | 48.5 | 1.688 | 0.020512 | 0.135714 |
| 5 | 11_21073 | 4H | 48.5 | 1.688 | 0.020512 | 0.135714 |
| 5 | 12_10195 | 4H | 48.5 | 1.684 | 0.020701 | 0.136038 |
| 5 | 12_30331 | 4H | 48.5 | 1.416 | 0.038371 | 0.173996 |
| 5 | 12_30878 | 4H | 48.5 | 1.688 | 0.020512 | 0.135714 |
| 5 | 12_31360 | 4H | 48.5 | 1.423 | 0.037757 | 0.173996 |
| 5 | 12_31382 | 4H | 48.5 | 1.423 | 0.037757 | 0.173996 |
| 5 | 12_30777 | 4H | 49.5 | 1.688 | 0.020512 | 0.135714 |
| 5 | 11_20289 | 4H | 50.4 | 0.379 | 0.41783 | 0.477069 |
| 5 | 12_30605 | 4H | 50.4 | 0.423 | 0.377572 | 0.452445 |
| 5 | 12_30684 | 4H | 50.4 | 0.489 | 0.32434 | 0.428623 |
| 5 | 11_20496 | 4H | 51.3 | 0.513 | 0.306902 | 0.422921 |
| 5 | 11_10946 | 4H | 52.8 | 0.443 | 0.360579 | 0.443718 |
| 5 | 12_30427 | 4H | 53.5 | 0.443 | 0.360579 | 0.443718 |
| 5 | 11_11244 | 4H | 55 | 0.694 | 0.202302 | 0.36056 |
| 5 | 11_10046 | 4H | 55.6 | 0.573 | 0.267301 | 0.39847 |
| 5 | 11_10262 | 4H | 55.6 | 1.049 | 0.089331 | 0.259736 |
| 5 | 11_10527 | 4H | 55.6 | 0.462 | 0.345144 | 0.434692 |
| 5 | 11_10568 | 4H | 55.6 | 0.573 | 0.267301 | 0.39847 |
| 5 | 12_30995 | 4H | 55.6 | 0.083 | 0.826038 | 0.577808 |
| 5 | 11_20482 | 4H | 59.4 | 1.246 | 0.056754 | 0.210865 |
| 5 | 11_21400 | 4H | 61 | 1.029 | 0.093541 | 0.264027 |
| 5 | 12_30237 | 4H | 61 | 1.174 | 0.066988 | 0.227028 |
| 5 | 11_20906 | 4H | 65 | 0.234 | 0.583445 | 0.527723 |
| 5 | 12_30620 | 4H | 65 | 1.73 | 0.018621 | 0.134237 |
| 5 | 12_30455 | 4H | 65.8 | 1.781 | 0.016558 | 0.122097 |
| 5 | 11_10010 | 4H | 66 | 0.234 | 0.583445 | 0.527723 |
| 5 | 12_30905 | 4H | 66 | 0.234 | 0.583445 | 0.527723 |
| 5 | 12_30906 | 4H | 66 | 0.234 | 0.583445 | 0.527723 |
| 5 | 12_31385 | 4H | 66 | 0.234 | 0.583445 | 0.527723 |
| 5 | 12_31493 | 4H | 66 | 0.251 | 0.561048 | 0.524151 |
| 5 | 11_10606 | 4H | 67.5 | 0.234 | 0.583445 | 0.527723 |
| 5 | 11_20072 | 4H | 67.5 | 0.623 | 0.238232 | 0.382279 |
| 5 | 11_10627 | 4H | 68.2 | 0.164 | 0.685488 | 0.553663 |
| 5 | 11_20451 | 4H | 68.2 | 0.164 | 0.685488 | 0.553663 |
| 5 | 11_20580 | 4H | 68.2 | 0.983 | 0.103992 | 0.278289 |
| 5 | 12_30693 | 4H | 68.2 | 0.164 | 0.685488 | 0.553663 |
| 5 | 12_31536 | 4H | 68.2 | 0.164 | 0.685488 | 0.553663 |
| 5 | 11_11513 | 4H | 69.5 | 0.164 | 0.685488 | 0.553663 |
| 5 | 11_21504 | 4H | 69.5 | 1.515 | 0.030549 | 0.162146 |
| 5 | 11_10467 | 4H | 72.1 | 1.095 | 0.080353 | 0.245635 |
| 5 | 12_31362 | 4H | 73.6 | 0.031 | 0.931108 | 0.61021 |
| 5 | 11_10829 | 4H | 73.8 | 2.18 | 0.006607 | 0.090761 |
| 5 | 11_10309 | 4H | 76 | 1.601 | 0.025061 | 0.151306 |
| 5 | 11_11004 | 4H | 77.3 | 1.355 | 0.044157 | 0.18546 |
| 5 | 11_21332 | 4H | 77.3 | 2.108 | 0.007798 | 0.090761 |
| 5 | 11_21353 | 4H | 77.3 | 1.355 | 0.044157 | 0.18546 |
| 5 | 12_30136 | 4H | 77.3 | 1.355 | 0.044157 | 0.18546 |
| 5 | 12_31231 | 4H | 77.3 | 1.355 | 0.044157 | 0.18546 |
| 5 | 11_10523 | 4H | 78.8 | 0.501 | 0.315501 | 0.426853 |
| 5 | 12_31148 | 4H | 78.8 | 0.501 | 0.315501 | 0.426853 |
| 5 | 11_11500 | 4H | 79.6 | 0.881 | 0.131523 | 0.295467 |
| 5 | 11_20197 | 4H | 81.7 | 1.254 | 0.055719 | 0.209432 |
| 5 | 11_10724 | 4H | 82.4 | 2.724 | 0.001888 | 0.060341 |
| 5 | 12_31246 | 4H | 83.5 | 1.56 | 0.027542 | 0.156293 |
| 5 | 12_30390 | 4H | 84.3 | 0.662 | 0.217771 | 0.374985 |
| 5 | 11_20178 | 4H | 86.3 | 0.119 | 0.760326 | 0.572021 |
| 5 | 11_20765 | 4H | 87.5 | 1.178 | 0.066374 | 0.227028 |
| 5 | 11_20358 | 4H | 88.2 | 0.982 | 0.104232 | 0.278289 |
| 5 | 11_10588 | 4H | 89.4 | 1.416 | 0.038371 | 0.173996 |
| 5 | 11_20384 | 4H | 91.8 | 0.095 | 0.803526 | 0.576266 |
| 5 | 11_20732 | 4H | 92.4 | 0.421 | 0.379315 | 0.452927 |
| 5 | 12_10271 | 4H | 96.6 | 0.5 | 0.316228 | 0.427213 |
| 5 | 12_30117 | 4H | 96.6 | 0.268 | 0.539511 | 0.516518 |
| 5 | 12_30554 | 4H | 96.6 | 0.037 | 0.918333 | 0.608025 |
| 5 | 11_20119 | 4H | 99.3 | 0.801 | 0.158125 | 0.309382 |
| 5 | 11_10614 | 4H | 100.7 | 0.203 | 0.626614 | 0.547295 |
| 5 | 11_11470 | 4H | 100.7 | 0.802 | 0.157761 | 0.309382 |
| 5 | 12_30158 | 4H | 100.7 | 0.018 | 0.959401 | 0.615801 |
| 5 | 11_10510 | 4H | 102.4 | 0.363 | 0.433511 | 0.484129 |
| 5 | 12_30990 | 4H | 103.1 | 0.374 | 0.422669 | 0.479745 |
| 5 | 11_20974 | 4H | 106 | 0.386 | 0.41115 | 0.474517 |
| 5 | 11_21130 | 4H | 116.8 | 0.17 | 0.676083 | 0.553663 |
| 5 | 11_10269 | 4H | 117.6 | 0.24 | 0.57544 | 0.527723 |
| 5 | 11_10610 | 4H | 119.1 | 0.18 | 0.660693 | 0.549724 |
| 5 | 11_20007 | 4H | 119.1 | 0.446 | 0.358096 | 0.441789 |
| 5 | 11_20272 | 4H | 119.1 | 0.528 | 0.296483 | 0.415679 |
| 5 | 12_30425 | 4H | 119.1 | 0.446 | 0.358096 | 0.441789 |
| 5 | 12_30476 | 4H | 119.1 | 0.18 | 0.660693 | 0.549724 |
| 5 | 12_30873 | 4H | 119.1 | 0.407 | 0.391742 | 0.462619 |
| 5 | 11_10387 | 4H | 119.8 | 0.588 | 0.258226 | 0.398123 |
| 5 | 12_30006 | 4H | 119.8 | 0.388 | 0.409261 | 0.473468 |
| 5 | 12_30239 | 4H | 119.8 | 0.256 | 0.554626 | 0.522191 |
| 5 | 12_31422 | 4H | 120.6 | 0.76 | 0.17378 | 0.327235 |
| 5 | 11_11186 | 4H | 121.8 | 0.119 | 0.760326 | 0.572021 |
| 5 | 11_11019 | 4H | 123.3 | 1.899 | 0.012618 | 0.109813 |
| 5 | 12_30824 | 4H | 123.3 | 1.777 | 0.016711 | 0.122293 |
| 5 | 12_30825 | 4H | 123.3 | 1.735 | 0.018408 | 0.133698 |
| 5 | 11_10251 | 5H | 0 | 1.469 | 0.033963 | 0.16851 |
| 5 | 11_10405 | 5H | 0 | 1.273 | 0.053333 | 0.204445 |
| 5 | 11_10593 | 5H | 0 | 0 | 1 | 0.626459 |
| 5 | 11_11361 | 5H | 0 | 0.14 | 0.724436 | 0.562092 |
| 5 | 11_20386 | 5H | 0 | 0.496 | 0.319154 | 0.427404 |
| 5 | 11_20644 | 5H | 0 | 0.187 | 0.65013 | 0.548015 |
| 5 | 11_21244 | 5H | 0 | 0.616 | 0.242103 | 0.384657 |
| 5 | 11_21514 | 5H | 0 | 1.919 | 0.01205 | 0.106795 |
| 5 | 11_20226 | 5H | 2.1 | 0.332 | 0.465586 | 0.495326 |
| 5 | 11_20894 | 5H | 2.1 | 1.17 | 0.067608 | 0.227028 |
| 5 | 12_30976 | 5H | 2.1 | 0.332 | 0.465586 | 0.495326 |
| 5 | 11_20553 | 5H | 2.8 | 0.345 | 0.451856 | 0.490441 |
| 5 | 12_30543 | 5H | 2.8 | 0.466 | 0.341979 | 0.432398 |
| 5 | 12_30975 | 5H | 5 | 0.271 | 0.535797 | 0.514492 |
| 5 | 12_31023 | 5H | 5 | 0.047 | 0.897429 | 0.601607 |
| 5 | 12_30001 | 5H | 5.7 | 0.332 | 0.465586 | 0.495326 |
| 5 | 12_30591 | 5H | 5.7 | 0.059 | 0.872971 | 0.592614 |
| 5 | 12_30979 | 5H | 5.7 | 0.332 | 0.465586 | 0.495326 |
| 5 | 11_20206 | 5H | 6.4 | 0.671 | 0.213305 | 0.370597 |
| 5 | 12_31022 | 5H | 6.4 | 0.316 | 0.483059 | 0.501441 |
| 5 | 11_21202 | 5H | 7 | 0.277 | 0.528445 | 0.509968 |
| 5 | 11_11381 | 5H | 7.5 | 0.113 | 0.770904 | 0.575737 |
| 5 | 11_20533 | 5H | 17.4 | 0.698 | 0.200447 | 0.358578 |
| 5 | 12_31094 | 5H | 18.1 | 0.698 | 0.200447 | 0.358578 |
| 5 | 11_20010 | 5H | 18.7 | 0.834 | 0.146555 | 0.299306 |
| 5 | 12_30714 | 5H | 19.4 | 0.254 | 0.557186 | 0.523072 |
| 5 | 11_10695 | 5H | 25.2 | 0.541 | 0.28774 | 0.4091 |
| 5 | 11_20873 | 5H | 26.3 | 0.062 | 0.866962 | 0.589363 |
| 5 | 11_21065 | 5H | 26.3 | 0.466 | 0.341979 | 0.432398 |
| 5 | 12_30167 | 5H | 26.3 | 0.479 | 0.331895 | 0.428623 |
| 5 | 11_10974 | 5H | 27 | 0.185 | 0.653131 | 0.54909 |
| 5 | 11_11048 | 5H | 29.9 | 0.042 | 0.907821 | 0.605213 |
| 5 | 11_21324 | 5H | 31 | 0.791 | 0.161808 | 0.313868 |
| 5 | 11_10688 | 5H | 34.2 | 2.035 | 0.009226 | 0.099023 |
| 5 | 12_10499 | 5H | 34.2 | 0.605 | 0.248313 | 0.390033 |
| 5 | 11_10580 | 5H | 35.7 | 0.308 | 0.49204 | 0.501441 |
| 5 | 11_10621 | 5H | 37.1 | 0.355 | 0.44157 | 0.487494 |
| 5 | 11_20845 | 5H | 40 | 0.715 | 0.192753 | 0.351319 |
| 5 | 11_20980 | 5H | 40 | 0.664 | 0.21677 | 0.373929 |
| 5 | 11_21391 | 5H | 40 | 0.687 | 0.205589 | 0.364402 |
| 5 | 12_30707 | 5H | 43.1 | 0.441 | 0.362243 | 0.444889 |
| 5 | 11_20729 | 5H | 44.1 | 0.196 | 0.636796 | 0.548015 |
| 5 | 11_10260 | 5H | 46.2 | 0.158 | 0.695024 | 0.559029 |
| 5 | 11_10955 | 5H | 46.2 | 1.829 | 0.014825 | 0.117657 |
| 5 | 11_20766 | 5H | 46.2 | 1.829 | 0.014825 | 0.117657 |
| 5 | 11_20987 | 5H | 46.2 | 0.062 | 0.866962 | 0.589363 |
| 5 | 12_30654 | 5H | 46.2 | 0.479 | 0.331895 | 0.428623 |
| 5 | 11_11432 | 5H | 47.4 | 0.085 | 0.822243 | 0.577808 |
| 5 | 12_31257 | 5H | 48.1 | 0.242 | 0.572796 | 0.527183 |
| 5 | 11_11198 | 5H | 48.8 | 0.365 | 0.431519 | 0.483574 |
| 5 | 11_21401 | 5H | 48.8 | 0.356 | 0.440555 | 0.486929 |
| 5 | 12_10923 | 5H | 48.8 | 0.167 | 0.680769 | 0.553663 |
| 5 | 12_31155 | 5H | 48.8 | 0.027 | 0.939723 | 0.611295 |
| 5 | 11_10116 | 5H | 50.3 | 0.245 | 0.568853 | 0.527183 |
| 5 | 11_20841 | 5H | 50.3 | 0.089 | 0.814704 | 0.576266 |
| 5 | 11_21308 | 5H | 50.3 | 0.142 | 0.721108 | 0.561313 |
| 5 | 11_21447 | 5H | 50.3 | 0.107 | 0.781628 | 0.575737 |
| 5 | 12_30354 | 5H | 50.3 | 0.245 | 0.568853 | 0.527183 |
| 5 | 12_30454 | 5H | 50.3 | 0.254 | 0.557186 | 0.523072 |
| 5 | 12_30729 | 5H | 50.3 | 0.089 | 0.814704 | 0.576266 |
| 5 | 12_31390 | 5H | 50.3 | 0.271 | 0.535797 | 0.514492 |
| 5 | 12_31512 | 5H | 50.3 | 0.254 | 0.557186 | 0.523072 |
| 5 | 11_20697 | 5H | 50.7 | 0.02 | 0.954993 | 0.615425 |
| 5 | 11_10157 | 5H | 51 | 0.572 | 0.267917 | 0.39847 |
| 5 | 11_10177 | 5H | 51 | 0.572 | 0.267917 | 0.39847 |
| 5 | 11_10240 | 5H | 51 | 0.572 | 0.267917 | 0.39847 |
| 5 | 11_10252 | 5H | 51 | 0.572 | 0.267917 | 0.39847 |
| 5 | 11_10318 | 5H | 51 | 0.572 | 0.267917 | 0.39847 |
| 5 | 11_10856 | 5H | 51 | 0.572 | 0.267917 | 0.39847 |
| 5 | 11_10995 | 5H | 51 | 0.074 | 0.843335 | 0.580657 |
| 5 | 11_20129 | 5H | 51 | 0.979 | 0.104954 | 0.278289 |
| 5 | 11_20700 | 5H | 51 | 0.074 | 0.843335 | 0.580657 |
| 5 | 11_20708 | 5H | 51 | 0.836 | 0.145881 | 0.299306 |
| 5 | 11_20958 | 5H | 51 | 0.979 | 0.104954 | 0.278289 |
| 5 | 11_21011 | 5H | 51 | 0.074 | 0.843335 | 0.580657 |
| 5 | 11_21040 | 5H | 51 | 0.572 | 0.267917 | 0.39847 |
| 5 | 11_21350 | 5H | 51 | 0.074 | 0.843335 | 0.580657 |
| 5 | 12_30337 | 5H | 51 | 0.726 | 0.187932 | 0.345137 |
| 5 | 12_30768 | 5H | 51 | 0.572 | 0.267917 | 0.39847 |
| 5 | 12_30792 | 5H | 51 | 0.631 | 0.233884 | 0.38083 |
| 5 | 12_31317 | 5H | 51 | 0.074 | 0.843335 | 0.580657 |
| 5 | 11_10058 | 5H | 51.3 | 0.235 | 0.582103 | 0.527723 |
| 5 | 11_10481 | 5H | 51.3 | 0.572 | 0.267917 | 0.39847 |
| 5 | 11_10913 | 5H | 51.3 | 0.572 | 0.267917 | 0.39847 |
| 5 | 12_30575 | 5H | 51.3 | 0.866 | 0.136145 | 0.299306 |
| 5 | 12_30709 | 5H | 51.3 | 0.478 | 0.33266 | 0.429037 |
| 5 | 12_30728 | 5H | 51.3 | 1.473 | 0.033651 | 0.16851 |
| 5 | 11_10661 | 5H | 51.6 | 1.005 | 0.098855 | 0.27129 |
| 5 | 11_11128 | 5H | 51.6 | 0.06 | 0.870964 | 0.591667 |
| 5 | 11_11469 | 5H | 51.6 | 0.545 | 0.285102 | 0.408618 |
| 5 | 11_11506 | 5H | 51.6 | 0.04 | 0.912011 | 0.605916 |
| 5 | 11_20179 | 5H | 51.6 | 0.585 | 0.260016 | 0.39847 |
| 5 | 11_20332 | 5H | 51.6 | 0.195 | 0.638264 | 0.548015 |
| 5 | 11_20461 | 5H | 51.6 | 0.074 | 0.843335 | 0.580657 |
| 5 | 11_20524 | 5H | 51.6 | 0.572 | 0.267917 | 0.39847 |
| 5 | 11_20737 | 5H | 51.6 | 0.086 | 0.820352 | 0.577174 |
| 5 | 11_21215 | 5H | 51.6 | 0.235 | 0.582103 | 0.527723 |
| 5 | 11_21260 | 5H | 51.6 | 0.195 | 0.638264 | 0.548015 |
| 5 | 12_30408 | 5H | 51.6 | 0.167 | 0.680769 | 0.553663 |
| 5 | 12_30717 | 5H | 51.6 | 0.118 | 0.762079 | 0.572448 |
| 5 | 12_31259 | 5H | 51.6 | 0.235 | 0.582103 | 0.527723 |
| 5 | 11_11260 | 5H | 52 | 0.168 | 0.679204 | 0.553663 |
| 5 | 11_21318 | 5H | 53.2 | 0.012 | 0.972747 | 0.619024 |
| 5 | 12_30214 | 5H | 53.9 | 0.035 | 0.922571 | 0.60833 |
| 5 | 11_21536 | 5H | 56.8 | 0.998 | 0.100462 | 0.273369 |
| 5 | 12_31117 | 5H | 56.8 | 0.998 | 0.100462 | 0.273369 |
| 5 | 11_20105 | 5H | 58 | 0.189 | 0.647143 | 0.548015 |
| 5 | 11_21148 | 5H | 58 | 0.189 | 0.647143 | 0.548015 |
| 5 | 12_10264 | 5H | 58 | 0.719 | 0.190985 | 0.349416 |
| 5 | 11_20306 | 5H | 58.7 | 2.133 | 0.007362 | 0.090761 |
| 5 | 11_10671 | 5H | 59.4 | 0.372 | 0.42462 | 0.479745 |
| 5 | 11_10840 | 5H | 59.4 | 0.372 | 0.42462 | 0.479745 |
| 5 | 11_11240 | 5H | 59.4 | 1.284 | 0.052 | 0.203014 |
| 5 | 11_20372 | 5H | 59.4 | 0.372 | 0.42462 | 0.479745 |
| 5 | 11_20501 | 5H | 59.4 | 1.284 | 0.052 | 0.203014 |
| 5 | 11_20961 | 5H | 59.4 | 1.284 | 0.052 | 0.203014 |
| 5 | 12_10034 | 5H | 59.4 | 1.312 | 0.048753 | 0.198335 |
| 5 | 12_30111 | 5H | 59.4 | 0.616 | 0.242103 | 0.384657 |
| 5 | 12_30538 | 5H | 59.4 | 2.36 | 0.004365 | 0.076668 |
| 5 | 12_30644 | 5H | 59.4 | 0.952 | 0.111686 | 0.286939 |
| 5 | 12_30700 | 5H | 59.4 | 1.064 | 0.086298 | 0.254158 |
| 5 | 12_30747 | 5H | 59.4 | 1.073 | 0.084528 | 0.253372 |
| 5 | 12_31340 | 5H | 59.4 | 1.284 | 0.052 | 0.203014 |
| 5 | 12_31503 | 5H | 59.4 | 0.337 | 0.460257 | 0.495109 |
| 5 | 11_11159 | 5H | 60.7 | 0.047 | 0.897429 | 0.601607 |
| 5 | 11_11221 | 5H | 60.7 | 0.587 | 0.258821 | 0.398123 |
| 5 | 11_21200 | 5H | 60.7 | 0.587 | 0.258821 | 0.398123 |
| 5 | 11_21508 | 5H | 60.7 | 0.627 | 0.236048 | 0.380913 |
| 5 | 12_30515 | 5H | 60.7 | 0.37 | 0.42658 | 0.481397 |
| 5 | 12_31033 | 5H | 60.7 | 0.047 | 0.897429 | 0.601607 |
| 5 | 11_20265 | 5H | 62.1 | 0.018 | 0.959401 | 0.615801 |
| 5 | 11_21344 | 5H | 63.3 | 0.554 | 0.279254 | 0.404437 |
| 5 | 12_30745 | 5H | 64 | 0.607 | 0.247172 | 0.389508 |
| 5 | 11_20713 | 5H | 65.5 | 1.013 | 0.097051 | 0.268628 |
| 5 | 12_31477 | 5H | 65.5 | 0.068 | 0.855067 | 0.585811 |
| 5 | 11_21275 | 5H | 67.5 | 0.235 | 0.582103 | 0.527723 |
| 5 | 11_21239 | 5H | 69.3 | 0.115 | 0.767362 | 0.574629 |
| 5 | 11_10641 | 5H | 69.9 | 0.034 | 0.924698 | 0.609317 |
| 5 | 12_30080 | 5H | 69.9 | 0.131 | 0.739605 | 0.564343 |
| 5 | 11_11249 | 5H | 70.5 | 0.021 | 0.952796 | 0.615425 |
| 5 | 11_20392 | 5H | 70.5 | 0.021 | 0.952796 | 0.615425 |
| 5 | 12_30007 | 5H | 70.5 | 0.003 | 0.993116 | 0.62417 |
| 5 | 11_20367 | 5H | 75.4 | 0.218 | 0.605341 | 0.536969 |
| 5 | 11_20236 | 5H | 80.6 | 0.63 | 0.234423 | 0.38083 |
| 5 | 11_20246 | 5H | 85.2 | 1.859 | 0.013836 | 0.114233 |
| 5 | 11_11355 | 5H | 86.6 | 0.192 | 0.642688 | 0.548015 |
| 5 | 11_20645 | 5H | 87.3 | 2.901 | 0.001256 | 0.055151 |
| 5 | 11_21480 | 5H | 89.4 | 1.542 | 0.028708 | 0.157567 |
| 5 | 12_31427 | 5H | 90.8 | 1.344 | 0.04529 | 0.189394 |
| 5 | 11_11290 | 5H | 94.4 | 0.745 | 0.179887 | 0.332258 |
| 5 | 11_21150 | 5H | 94.4 | 0.02 | 0.954993 | 0.615425 |
| 5 | 11_20497 | 5H | 95.8 | 0.68 | 0.20893 | 0.366956 |
| 5 | 11_20526 | 5H | 99.6 | 0.333 | 0.464515 | 0.495326 |
| 5 | 12_31271 | 5H | 99.6 | 0.237 | 0.579429 | 0.527723 |
| 5 | 11_10518 | 5H | 100.3 | 0.166 | 0.682339 | 0.553663 |
| 5 | 11_11473 | 5H | 100.3 | 2.002 | 0.009954 | 0.103394 |
| 5 | 11_20097 | 5H | 100.3 | 5.692 | 2.03E-06 | 0.000654 |
| 5 | 11_20449 | 5H | 100.3 | 0.137 | 0.729458 | 0.562773 |
| 5 | 12_30533 | 5H | 100.3 | 0.14 | 0.724436 | 0.562092 |
| 5 | 12_30834 | 5H | 100.3 | 0.181 | 0.659174 | 0.549724 |
| 5 | 11_20850 | 5H | 102.1 | 2.267 | 0.005408 | 0.08162 |
| 5 | 12_10408 | 5H | 102.1 | 0.8 | 0.158489 | 0.309382 |
| 5 | 11_10622 | 5H | 103 | 0.354 | 0.442588 | 0.487503 |
| 5 | 11_21421 | 5H | 103.7 | 1.039 | 0.091411 | 0.262632 |
| 5 | 11_10414 | 5H | 103.9 | 0.626 | 0.236592 | 0.380913 |
| 5 | 11_20327 | 5H | 103.9 | 0.626 | 0.236592 | 0.380913 |
| 5 | 11_11350 | 5H | 104.5 | 0.354 | 0.442588 | 0.487503 |
| 5 | 12_30098 | 5H | 105.2 | 0.457 | 0.34914 | 0.436878 |
| 5 | 11_20018 | 5H | 106.1 | 0.457 | 0.34914 | 0.436878 |
| 5 | 11_20134 | 5H | 106.2 | 3.939 | 0.000115 | 0.015302 |
| 5 | 11_10024 | 5H | 107.6 | 1.375 | 0.04217 | 0.183515 |
| 5 | 11_20549 | 5H | 108 | 0.573 | 0.267301 | 0.39847 |
| 5 | 11_10834 | 5H | 108.2 | 0.313 | 0.486407 | 0.501441 |
| 5 | 11_20320 | 5H | 108.2 | 1.219 | 0.060395 | 0.219329 |
| 5 | 11_21314 | 5H | 108.2 | 1.807 | 0.015596 | 0.118513 |
| 5 | 12_10844 | 5H | 108.2 | 0.679 | 0.209411 | 0.367135 |
| 5 | 12_30854 | 5H | 108.2 | 0.508 | 0.310456 | 0.424187 |
| 5 | 12_30855 | 5H | 108.2 | 1.373 | 0.042364 | 0.183515 |
| 5 | 12_31417 | 5H | 108.2 | 0.217 | 0.606736 | 0.53722 |
| 5 | 11_20795 | 5H | 108.6 | 0.8 | 0.158489 | 0.309382 |
| 5 | 11_21168 | 5H | 109.6 | 1.165 | 0.068391 | 0.227813 |
| 5 | 11_21061 | 5H | 110.3 | 0.199 | 0.632412 | 0.548015 |
| 5 | 11_11273 | 5H | 111.7 | 0.031 | 0.931108 | 0.61021 |
| 5 | 11_10477 | 5H | 113.1 | 0.241 | 0.574117 | 0.527183 |
| 5 | 12_30456 | 5H | 113.1 | 0.344 | 0.452898 | 0.49102 |
| 5 | 11_11200 | 5H | 117.5 | 0.508 | 0.310456 | 0.424187 |
| 5 | 11_10094 | 5H | 122.4 | 3.846 | 0.000143 | 0.015302 |
| 5 | 11_20629 | 5H | 122.4 | 3.846 | 0.000143 | 0.015302 |
| 5 | 11_20127 | 5H | 123.5 | 0.387 | 0.410204 | 0.473992 |
| 5 | 12_30524 | 5H | 123.5 | 0.807 | 0.155955 | 0.309349 |
| 5 | 12_31278 | 5H | 124.2 | 0.485 | 0.327341 | 0.428623 |
| 5 | 11_11507 | 5H | 125.8 | 0.595 | 0.254097 | 0.396173 |
| 5 | 12_30377 | 5H | 128.7 | 0.006 | 0.98628 | 0.622303 |
| 5 | 11_10360 | 5H | 129.4 | 0.851 | 0.140929 | 0.299306 |
| 5 | 11_20003 | 5H | 129.4 | 0.851 | 0.140929 | 0.299306 |
| 5 | 11_20347 | 5H | 129.4 | 0.1 | 0.794328 | 0.575737 |
| 5 | 11_20653 | 5H | 129.4 | 0.609 | 0.246037 | 0.388352 |
| 5 | 11_21203 | 5H | 129.4 | 0.596 | 0.253513 | 0.396173 |
| 5 | 11_21325 | 5H | 129.4 | 0.701 | 0.199067 | 0.357433 |
| 5 | 12_11535 | 5H | 129.4 | 0.47 | 0.338844 | 0.432398 |
| 5 | 12_30590 | 5H | 129.4 | 0.098 | 0.797995 | 0.575737 |
| 5 | 12_30611 | 5H | 129.4 | 0.35 | 0.446684 | 0.488139 |
| 5 | 11_10805 | 5H | 130.1 | 0.23 | 0.588844 | 0.530618 |
| 5 | 11_20300 | 5H | 130.1 | 0.313 | 0.486407 | 0.501441 |
| 5 | 11_11375 | 5H | 130.8 | 0.934 | 0.116413 | 0.295467 |
| 5 | 12_30067 | 5H | 131.6 | 0.735 | 0.184077 | 0.338702 |
| 5 | 11_10705 | 5H | 132.6 | 0.581 | 0.262422 | 0.39847 |
| 5 | 11_20259 | 5H | 132.6 | 1.099 | 0.079616 | 0.245635 |
| 5 | 11_20298 | 5H | 132.6 | 0.826 | 0.149279 | 0.302314 |
| 5 | 11_20487 | 5H | 134.6 | 4.135 | 7.33E-05 | 0.014158 |
| 5 | 11_10095 | 5H | 137.2 | 0.667 | 0.215278 | 0.372019 |
| 5 | 11_10855 | 5H | 137.2 | 1.809 | 0.015524 | 0.118513 |
| 5 | 11_20884 | 5H | 137.2 | 1.89 | 0.012883 | 0.110128 |
| 5 | 11_21241 | 5H | 137.2 | 0.581 | 0.262422 | 0.39847 |
| 5 | 12_30929 | 5H | 137.2 | 0.439 | 0.363915 | 0.444889 |
| 5 | 12_31237 | 5H | 137.2 | 0.561 | 0.274789 | 0.400977 |
| 5 | 11_10755 | 5H | 142.2 | 1.186 | 0.065163 | 0.226138 |
| 5 | 11_10845 | 5H | 142.2 | 0.479 | 0.331895 | 0.428623 |
| 5 | 11_11071 | 5H | 142.2 | 0.197 | 0.635331 | 0.548015 |
| 5 | 11_11532 | 5H | 142.2 | 0.479 | 0.331895 | 0.428623 |
| 5 | 11_21289 | 5H | 142.2 | 0.479 | 0.331895 | 0.428623 |
| 5 | 12_31366 | 5H | 142.2 | 0.197 | 0.635331 | 0.548015 |
| 5 | 11_10819 | 5H | 143.9 | 0.434 | 0.368129 | 0.445072 |
| 5 | 11_20375 | 5H | 143.9 | 0.434 | 0.368129 | 0.445072 |
| 5 | 12_30556 | 5H | 143.9 | 0.886 | 0.130017 | 0.295467 |
| 5 | 11_10292 | 5H | 144.6 | 0.434 | 0.368129 | 0.445072 |
| 5 | 12_31217 | 5H | 144.6 | 0.434 | 0.368129 | 0.445072 |
| 5 | 11_11092 | 5H | 145.3 | 0.705 | 0.197242 | 0.355478 |
| 5 | 11_10104 | 5H | 146 | 0.156 | 0.698232 | 0.559354 |
| 5 | 11_20568 | 5H | 146 | 1.397 | 0.040087 | 0.17845 |
| 5 | 11_20676 | 5H | 146 | 0.153 | 0.703072 | 0.559354 |
| 5 | 11_20731 | 5H | 146 | 0.153 | 0.703072 | 0.559354 |
| 5 | 11_21077 | 5H | 146 | 1.56 | 0.027542 | 0.156293 |
| 5 | 12_30400 | 5H | 149.1 | 0.314 | 0.485289 | 0.501441 |
| 5 | 12_30580 | 5H | 149.1 | 1.412 | 0.038726 | 0.173996 |
| 5 | 11_20388 | 5H | 150.3 | 0.373 | 0.423643 | 0.479745 |
| 5 | 11_10080 | 5H | 151.4 | 1.144 | 0.071779 | 0.231903 |
| 5 | 11_11441 | 5H | 151.4 | 1.274 | 0.053211 | 0.204445 |
| 5 | 12_10904 | 5H | 151.4 | 0.764 | 0.172187 | 0.324868 |
| 5 | 12_31206 | 5H | 151.4 | 1.412 | 0.038726 | 0.173996 |
| 5 | 11_10217 | 5H | 153.5 | 1.529 | 0.02958 | 0.158747 |
| 5 | 11_10589 | 5H | 153.5 | 1.529 | 0.02958 | 0.158747 |
| 5 | 11_20104 | 5H | 153.5 | 1.529 | 0.02958 | 0.158747 |
| 5 | 11_21355 | 5H | 153.5 | 1.12 | 0.075858 | 0.238692 |
| 5 | 12_10016 | 5H | 153.5 | 0.764 | 0.172187 | 0.324868 |
| 5 | 12_30830 | 5H | 153.5 | 0.921 | 0.11995 | 0.295467 |
| 5 | 11_11497 | 5H | 155.1 | 2.096 | 0.008017 | 0.091108 |
| 5 | 11_10901 | 5H | 158.4 | 1.251 | 0.056105 | 0.210067 |
| 5 | 11_10820 | 5H | 159.1 | 0.681 | 0.208449 | 0.366779 |
| 5 | 11_11185 | 5H | 159.1 | 0.671 | 0.213305 | 0.370597 |
| 5 | 11_21041 | 5H | 159.1 | 0.681 | 0.208449 | 0.366779 |
| 5 | 11_10161 | 5H | 159.8 | 0.897 | 0.126765 | 0.295467 |
| 5 | 11_10528 | 5H | 159.8 | 1.05 | 0.089125 | 0.259736 |
| 5 | 11_10536 | 5H | 159.8 | 0.1 | 0.794328 | 0.575737 |
| 5 | 11_10582 | 5H | 159.8 | 0.04 | 0.912011 | 0.605916 |
| 5 | 11_10741 | 5H | 159.8 | 0.897 | 0.126765 | 0.295467 |
| 5 | 11_10902 | 5H | 159.8 | 0.897 | 0.126765 | 0.295467 |
| 5 | 11_20078 | 5H | 159.8 | 0.04 | 0.912011 | 0.605916 |
| 5 | 11_20545 | 5H | 159.8 | 0.594 | 0.254683 | 0.396173 |
| 5 | 11_21024 | 5H | 159.8 | 0.01 | 0.977237 | 0.619024 |
| 5 | 11_21452 | 5H | 159.8 | 1.048 | 0.089536 | 0.259736 |
| 5 | 12_30165 | 5H | 159.8 | 1.45 | 0.035481 | 0.168842 |
| 5 | 12_30759 | 5H | 159.8 | 2.146 | 0.007145 | 0.090761 |
| 5 | 11_10336 | 5H | 161.6 | 0.119 | 0.760326 | 0.572021 |
| 5 | 11_11464 | 5H | 161.6 | 1.438 | 0.036475 | 0.172722 |
| 5 | 11_20646 | 5H | 161.6 | 1.187 | 0.065013 | 0.226138 |
| 5 | 11_20988 | 5H | 161.6 | 0.535 | 0.291743 | 0.412022 |
| 5 | 11_21018 | 5H | 161.6 | 0.222 | 0.599791 | 0.535983 |
| 5 | 12_30162 | 5H | 161.6 | 0.262 | 0.547016 | 0.518565 |
| 5 | 12_30642 | 5H | 161.6 | 1.187 | 0.065013 | 0.226138 |
| 5 | 12_31375 | 5H | 161.6 | 0.877 | 0.132739 | 0.29682 |
| 5 | 11_20334 | 5H | 166.6 | 0.042 | 0.907821 | 0.605213 |
| 5 | 11_20934 | 5H | 166.6 | 0.97 | 0.107152 | 0.281274 |
| 5 | 11_20829 | 5H | 168.8 | 1.332 | 0.046559 | 0.19386 |
| 5 | 12_30666 | 5H | 169.5 | 0.064 | 0.862979 | 0.588311 |
| 5 | 11_11216 | 5H | 171.7 | 1.204 | 0.062517 | 0.224504 |
| 5 | 11_20546 | 5H | 172.4 | 0.143 | 0.719449 | 0.561313 |
| 5 | 11_20686 | 5H | 172.4 | 0.293 | 0.509331 | 0.501441 |
| 5 | 11_10869 | 5H | 173.1 | 0.092 | 0.809096 | 0.576266 |
| 5 | 11_10778 | 5H | 175.9 | 0.093 | 0.807235 | 0.576266 |
| 5 | 11_10600 | 5H | 176.6 | 0.204 | 0.625173 | 0.547295 |
| 5 | 11_21012 | 5H | 176.6 | 0.093 | 0.807235 | 0.576266 |
| 5 | 12_20867 | 5H | 176.6 | 0.071 | 0.849181 | 0.583019 |
| 5 | 12_21290 | 5H | 176.6 | 0.071 | 0.849181 | 0.583019 |
| 5 | 11_21141 | 5H | 177.1 | 0.088 | 0.816582 | 0.576266 |
| 5 | 11_20536 | 5H | 177.7 | 0.098 | 0.797995 | 0.575737 |
| 5 | 12_21009 | 5H | 177.7 | 0.071 | 0.849181 | 0.583019 |
| 5 | 12_11010 | 5H | 178.4 | 0.808 | 0.155597 | 0.309349 |
| 5 | 12_11450 | 5H | 178.4 | 0.808 | 0.155597 | 0.309349 |
| 5 | 12_20816 | 5H | 178.4 | 0.12 | 0.758578 | 0.572021 |
| 5 | 11_10254 | 5H | 179.1 | 0.412 | 0.387258 | 0.458445 |
| 5 | 11_21138 | 5H | 179.6 | 0.481 | 0.33037 | 0.428623 |
| 5 | 12_30656 | 5H | 179.6 | 0.921 | 0.11995 | 0.295467 |
| 5 | 11_10736 | 5H | 180.7 | 0.164 | 0.685488 | 0.553663 |
| 5 | 12_30494 | 5H | 180.7 | 0.097 | 0.799834 | 0.575737 |
| 5 | 11_10236 | 5H | 181.4 | 0.364 | 0.432514 | 0.483574 |
| 5 | 11_20022 | 5H | 181.4 | 1.467 | 0.034119 | 0.16851 |
| 5 | 11_20897 | 5H | 182.9 | 1.077 | 0.083753 | 0.253372 |
| 5 | 12_30577 | 5H | 182.9 | 0.912 | 0.122462 | 0.295467 |
| 5 | 12_30769 | 5H | 182.9 | 0.556 | 0.277971 | 0.404398 |
| 5 | 12_31352 | 5H | 182.9 | 0.873 | 0.133968 | 0.298186 |
| 5 | 11_21155 | 5H | 187.4 | 0.631 | 0.233884 | 0.38083 |
| 5 | 11_20786 | 5H | 189.6 | 1.515 | 0.030549 | 0.162146 |
| 5 | 12_31292 | 5H | 189.6 | 1.594 | 0.025468 | 0.15281 |
| 5 | 12_31210 | 5H | 192 | 1.405 | 0.039355 | 0.176004 |
| 5 | 12_31481 | 5H | 192 | 2.256 | 0.005546 | 0.082426 |
| 5 | 12_10857 | 5H | 194.8 | 2.224 | 0.00597 | 0.08608 |
| 5 | 11_20402 | 5H | 195.4 | 1.221 | 0.060117 | 0.219145 |
| 5 | 11_20132 | 5H | 196.1 | 0.247 | 0.566239 | 0.525949 |
| 5 | 12_10322 | 5H | 196.1 | 1.008 | 0.098175 | 0.27019 |
| 5 | 12_30958 | 5H | 196.1 | 2.08 | 0.008318 | 0.091305 |
| 5 | 12_31123 | 5H | 196.8 | 1.009 | 0.097949 | 0.27019 |
| 5 | 11_10496 | 6H | 0 | 1.981 | 0.010447 | 0.104653 |
| 5 | 11_11329 | 6H | 0 | 0.028 | 0.937562 | 0.611123 |
| 5 | 11_11406 | 6H | 0 | 1.206 | 0.06223 | 0.224307 |
| 5 | 11_20212 | 6H | 0 | 0.351 | 0.445656 | 0.4881 |
| 5 | 11_20232 | 6H | 0 | 1.469 | 0.033963 | 0.16851 |
| 5 | 11_20292 | 6H | 0 | 0.182 | 0.657658 | 0.54909 |
| 5 | 11_20336 | 6H | 0 | 0.152 | 0.704693 | 0.559354 |
| 5 | 11_20465 | 6H | 0 | 1.364 | 0.043251 | 0.18546 |
| 5 | 12_30319 | 6H | 0 | 0.122 | 0.755092 | 0.571645 |
| 5 | 11_20493 | 6H | 1.3 | 1.487 | 0.032584 | 0.16851 |
| 5 | 11_20881 | 6H | 1.3 | 0.9 | 0.125893 | 0.295467 |
| 5 | 11_20886 | 6H | 1.3 | 1.413 | 0.038637 | 0.173996 |
| 5 | 11_10669 | 6H | 2.3 | 0.453 | 0.352371 | 0.439781 |
| 5 | 11_10120 | 6H | 3.1 | 0.482 | 0.32961 | 0.428623 |
| 5 | 11_21521 | 6H | 3.1 | 0.351 | 0.445656 | 0.4881 |
| 5 | 11_20882 | 6H | 3.2 | 0.44 | 0.363078 | 0.444889 |
| 5 | 11_20294 | 6H | 5.4 | 0.335 | 0.462381 | 0.495326 |
| 5 | 11_21204 | 6H | 6.1 | 1.126 | 0.074817 | 0.238525 |
| 5 | 11_20262 | 6H | 8.1 | 1.415 | 0.038459 | 0.173996 |
| 5 | 11_11479 | 6H | 12.5 | 0.215 | 0.609537 | 0.53822 |
| 5 | 12_30842 | 6H | 17 | 0.019 | 0.957194 | 0.615801 |
| 5 | 11_10023 | 6H | 22.4 | 0.803 | 0.157398 | 0.309382 |
| 5 | 11_21246 | 6H | 22.4 | 1.585 | 0.026002 | 0.154095 |
| 5 | 12_30843 | 6H | 22.4 | 0.803 | 0.157398 | 0.309382 |
| 5 | 11_10136 | 6H | 24.4 | 1.838 | 0.014521 | 0.117657 |
| 5 | 11_10868 | 6H | 24.4 | 3.197 | 0.000635 | 0.036102 |
| 5 | 11_20315 | 6H | 24.4 | 1.676 | 0.021086 | 0.137631 |
| 5 | 11_10676 | 6H | 28.4 | 1.173 | 0.067143 | 0.227028 |
| 5 | 12_30697 | 6H | 29.1 | 0.066 | 0.859014 | 0.586851 |
| 5 | 12_30673 | 6H | 30.1 | 0.028 | 0.937562 | 0.611123 |
| 5 | 12_31308 | 6H | 30.7 | 0.028 | 0.937562 | 0.611123 |
| 5 | 11_10799 | 6H | 31.7 | 2.733 | 0.001849 | 0.060341 |
| 5 | 11_10939 | 6H | 33.7 | 2.727 | 0.001875 | 0.060341 |
| 5 | 11_10427 | 6H | 34.4 | 4.169 | 6.78E-05 | 0.014158 |
| 5 | 12_30521 | 6H | 38.4 | 1.804 | 0.015704 | 0.118513 |
| 5 | 12_30361 | 6H | 40.8 | 0.077 | 0.837529 | 0.580657 |
| 5 | 11_10061 | 6H | 42.4 | 0.168 | 0.679204 | 0.553663 |
| 5 | 11_10129 | 6H | 42.4 | 0.201 | 0.629506 | 0.548015 |
| 5 | 11_10494 | 6H | 42.4 | 0.027 | 0.939723 | 0.611295 |
| 5 | 11_10882 | 6H | 42.4 | 0.002 | 0.995405 | 0.625202 |
| 5 | 11_20052 | 6H | 42.4 | 0.229 | 0.590201 | 0.530851 |
| 5 | 11_21030 | 6H | 42.4 | 0.229 | 0.590201 | 0.530851 |
| 5 | 12_11455 | 6H | 42.4 | 0.056 | 0.879023 | 0.595885 |
| 5 | 12_30665 | 6H | 42.4 | 0.298 | 0.503501 | 0.501441 |
| 5 | 12_30783 | 6H | 42.4 | 0.082 | 0.827942 | 0.577884 |
| 5 | 11_10244 | 6H | 43.1 | 0.298 | 0.503501 | 0.501441 |
| 5 | 11_20936 | 6H | 43.1 | 0.263 | 0.545758 | 0.518389 |
| 5 | 11_10462 | 6H | 44.8 | 1.043 | 0.090573 | 0.261175 |
| 5 | 11_20743 | 6H | 44.8 | 1.043 | 0.090573 | 0.261175 |
| 5 | 11_10013 | 6H | 45.4 | 0.635 | 0.23174 | 0.38083 |
| 5 | 11_10817 | 6H | 45.4 | 0.635 | 0.23174 | 0.38083 |
| 5 | 11_11097 | 6H | 45.4 | 0.635 | 0.23174 | 0.38083 |
| 5 | 11_20707 | 6H | 45.4 | 0.635 | 0.23174 | 0.38083 |
| 5 | 12_10910 | 6H | 45.4 | 0.837 | 0.145546 | 0.299306 |
| 5 | 12_30317 | 6H | 45.4 | 0.635 | 0.23174 | 0.38083 |
| 5 | 11_10539 | 6H | 46.1 | 0.635 | 0.23174 | 0.38083 |
| 5 | 11_10461 | 6H | 48.7 | 0.837 | 0.145546 | 0.299306 |
| 5 | 12_30133 | 6H | 48.7 | 0.837 | 0.145546 | 0.299306 |
| 5 | 12_30316 | 6H | 48.7 | 0.635 | 0.23174 | 0.38083 |
| 5 | 12_30658 | 6H | 48.7 | 0.635 | 0.23174 | 0.38083 |
| 5 | 11_20291 | 6H | 49.4 | 0.837 | 0.145546 | 0.299306 |
| 5 | 12_30510 | 6H | 49.4 | 0.837 | 0.145546 | 0.299306 |
| 5 | 12_31092 | 6H | 49.4 | 0.816 | 0.152757 | 0.306783 |
| 5 | 12_31274 | 6H | 49.4 | 0.837 | 0.145546 | 0.299306 |
| 5 | 12_31433 | 6H | 49.4 | 0.837 | 0.145546 | 0.299306 |
| 5 | 11_11205 | 6H | 50.1 | 0.837 | 0.145546 | 0.299306 |
| 5 | 12_30569 | 6H | 51.4 | 0.856 | 0.139316 | 0.299306 |
| 5 | 11_20720 | 6H | 52.8 | 0.149 | 0.709578 | 0.559553 |
| 5 | 12_30120 | 6H | 52.8 | 0.242 | 0.572796 | 0.527183 |
| 5 | 12_30430 | 6H | 52.8 | 0.149 | 0.709578 | 0.559553 |
| 5 | 12_30473 | 6H | 52.8 | 0.857 | 0.138995 | 0.299306 |
| 5 | 11_21473 | 6H | 53.3 | 0.149 | 0.709578 | 0.559553 |
| 5 | 11_21158 | 6H | 54 | 0.149 | 0.709578 | 0.559553 |
| 5 | 11_10962 | 6H | 54.6 | 0.242 | 0.572796 | 0.527183 |
| 5 | 11_20651 | 6H | 54.6 | 0.323 | 0.475335 | 0.501441 |
| 5 | 11_20656 | 6H | 54.6 | 0.358 | 0.438531 | 0.485803 |
| 5 | 11_21014 | 6H | 54.6 | 0.242 | 0.572796 | 0.527183 |
| 5 | 12_30021 | 6H | 54.6 | 0.264 | 0.544503 | 0.517706 |
| 5 | 12_30441 | 6H | 54.6 | 0.155 | 0.699842 | 0.559354 |
| 5 | 12_30802 | 6H | 54.6 | 0.251 | 0.561048 | 0.524151 |
| 5 | 12_31004 | 6H | 54.6 | 0.292 | 0.510505 | 0.501441 |
| 5 | 12_31005 | 6H | 54.6 | 0.149 | 0.709578 | 0.559553 |
| 5 | 11_10954 | 6H | 55 | 0.292 | 0.510505 | 0.501441 |
| 5 | 11_11312 | 6H | 55 | 0.292 | 0.510505 | 0.501441 |
| 5 | 11_20600 | 6H | 55 | 0.292 | 0.510505 | 0.501441 |
| 5 | 12_30508 | 6H | 55.4 | 0.292 | 0.510505 | 0.501441 |
| 5 | 11_10323 | 6H | 55.6 | 0.292 | 0.510505 | 0.501441 |
| 5 | 11_10749 | 6H | 55.6 | 0.292 | 0.510505 | 0.501441 |
| 5 | 11_10848 | 6H | 55.6 | 0.292 | 0.510505 | 0.501441 |
| 5 | 11_20329 | 6H | 55.6 | 0.292 | 0.510505 | 0.501441 |
| 5 | 11_20567 | 6H | 55.6 | 0.291 | 0.511682 | 0.501441 |
| 5 | 11_20572 | 6H | 55.6 | 0.292 | 0.510505 | 0.501441 |
| 5 | 11_20785 | 6H | 55.6 | 0.292 | 0.510505 | 0.501441 |
| 5 | 11_20799 | 6H | 55.6 | 0.292 | 0.510505 | 0.501441 |
| 5 | 11_20854 | 6H | 55.6 | 0.292 | 0.510505 | 0.501441 |
| 5 | 11_20946 | 6H | 55.6 | 0.292 | 0.510505 | 0.501441 |
| 5 | 11_21124 | 6H | 55.6 | 0.291 | 0.511682 | 0.501441 |
| 5 | 11_21216 | 6H | 55.6 | 0.292 | 0.510505 | 0.501441 |
| 5 | 12_30765 | 6H | 55.6 | 0.292 | 0.510505 | 0.501441 |
| 5 | 12_30837 | 6H | 55.6 | 0.292 | 0.510505 | 0.501441 |
| 5 | 12_31187 | 6H | 55.6 | 0.292 | 0.510505 | 0.501441 |
| 5 | 12_31249 | 6H | 55.6 | 0.292 | 0.510505 | 0.501441 |
| 5 | 12_31443 | 6H | 55.6 | 0.36 | 0.436516 | 0.484683 |
| 5 | 11_10227 | 6H | 55.9 | 0.341 | 0.456037 | 0.493316 |
| 5 | 11_10377 | 6H | 55.9 | 0.297 | 0.504661 | 0.501441 |
| 5 | 11_10513 | 6H | 55.9 | 0.01 | 0.977237 | 0.619024 |
| 5 | 11_20835 | 6H | 55.9 | 0.341 | 0.456037 | 0.493316 |
| 5 | 12_30311 | 6H | 55.9 | 0.291 | 0.511682 | 0.501441 |
| 5 | 12_30465 | 6H | 55.9 | 0.292 | 0.510505 | 0.501441 |
| 5 | 12_30511 | 6H | 55.9 | 0.292 | 0.510505 | 0.501441 |
| 5 | 12_30596 | 6H | 55.9 | 0.292 | 0.510505 | 0.501441 |
| 5 | 12_30681 | 6H | 55.9 | 0.292 | 0.510505 | 0.501441 |
| 5 | 12_30749 | 6H | 55.9 | 0.292 | 0.510505 | 0.501441 |
| 5 | 12_30856 | 6H | 55.9 | 0.292 | 0.510505 | 0.501441 |
| 5 | 12_31006 | 6H | 55.9 | 0.337 | 0.460257 | 0.495109 |
| 5 | 12_31178 | 6H | 55.9 | 0.351 | 0.445656 | 0.4881 |
| 5 | 12_30144 | 6H | 56.5 | 0.216 | 0.608135 | 0.537473 |
| 5 | 12_30857 | 6H | 56.5 | 0.307 | 0.493174 | 0.501441 |
| 5 | 11_11067 | 6H | 58 | 0.105 | 0.785236 | 0.575737 |
| 5 | 11_21339 | 6H | 58.5 | 0.105 | 0.785236 | 0.575737 |
| 5 | 11_10964 | 6H | 59.6 | 0.15 | 0.707946 | 0.559553 |
| 5 | 11_20266 | 6H | 59.6 | 0.105 | 0.785236 | 0.575737 |
| 5 | 11_10189 | 6H | 60.2 | 0.457 | 0.34914 | 0.436878 |
| 5 | 11_10270 | 6H | 60.2 | 0.365 | 0.431519 | 0.483574 |
| 5 | 11_10635 | 6H | 60.2 | 0.466 | 0.341979 | 0.432398 |
| 5 | 11_20058 | 6H | 60.2 | 0.466 | 0.341979 | 0.432398 |
| 5 | 11_21310 | 6H | 60.2 | 0.466 | 0.341979 | 0.432398 |
| 5 | 12_30346 | 6H | 60.2 | 0.2 | 0.630957 | 0.548015 |
| 5 | 11_11483 | 6H | 63.3 | 0.709 | 0.195434 | 0.353538 |
| 5 | 11_21298 | 6H | 63.3 | 0.709 | 0.195434 | 0.353538 |
| 5 | 11_21069 | 6H | 64 | 0.422 | 0.378443 | 0.452445 |
| 5 | 11_10455 | 6H | 64.4 | 0.379 | 0.41783 | 0.477069 |
| 5 | 11_10781 | 6H | 64.4 | 0.282 | 0.522396 | 0.506153 |
| 5 | 11_10040 | 6H | 65 | 0.219 | 0.603949 | 0.536969 |
| 5 | 11_10124 | 6H | 65 | 0.536 | 0.291072 | 0.411677 |
| 5 | 11_11261 | 6H | 65 | 0.18 | 0.660693 | 0.549724 |
| 5 | 11_20468 | 6H | 67.7 | 0.244 | 0.570164 | 0.527183 |
| 5 | 12_31289 | 6H | 69.4 | 0.046 | 0.899498 | 0.602158 |
| 5 | 11_20620 | 6H | 70 | 0.046 | 0.899498 | 0.602158 |
| 5 | 11_20673 | 6H | 70 | 0.781 | 0.165577 | 0.317356 |
| 5 | 11_20892 | 6H | 70 | 1.318 | 0.048084 | 0.198111 |
| 5 | 11_11349 | 6H | 71.1 | 0.062 | 0.866962 | 0.589363 |
| 5 | 11_20784 | 6H | 71.1 | 0.218 | 0.605341 | 0.536969 |
| 5 | 12_31250 | 6H | 71.1 | 0.151 | 0.706318 | 0.559553 |
| 5 | 12_31101 | 6H | 71.9 | 0.38 | 0.416869 | 0.477069 |
| 5 | 11_10469 | 6H | 72.5 | 0.483 | 0.328852 | 0.428623 |
| 5 | 12_30940 | 6H | 72.5 | 0.463 | 0.34435 | 0.434258 |
| 5 | 12_31111 | 6H | 72.5 | 0.224 | 0.597035 | 0.53451 |
| 5 | 12_31088 | 6H | 74.5 | 0.152 | 0.704693 | 0.559354 |
| 5 | 11_20889 | 6H | 75.2 | 0.102 | 0.790679 | 0.575737 |
| 5 | 11_20746 | 6H | 76.5 | 0.016 | 0.963829 | 0.617004 |
| 5 | 12_30573 | 6H | 76.5 | 0.219 | 0.603949 | 0.536969 |
| 5 | 11_21224 | 6H | 77.9 | 1.555 | 0.027861 | 0.156293 |
| 5 | 11_10608 | 6H | 80.5 | 1.101 | 0.07925 | 0.245635 |
| 5 | 11_10220 | 6H | 81.2 | 0.563 | 0.273527 | 0.400977 |
| 5 | 11_11458 | 6H | 81.2 | 0.563 | 0.273527 | 0.400977 |
| 5 | 11_10331 | 6H | 81.2 | 0.549 | 0.282488 | 0.407012 |
| 5 | 11_10185 | 6H | 81.9 | 0.556 | 0.277971 | 0.404398 |
| 5 | 11_11246 | 6H | 81.9 | 0.549 | 0.282488 | 0.407012 |
| 5 | 11_20346 | 6H | 82.6 | 0.459 | 0.347536 | 0.436567 |
| 5 | 11_21404 | 6H | 82.6 | 1.703 | 0.019815 | 0.135714 |
| 5 | 12_30698 | 6H | 86.9 | 0.087 | 0.818465 | 0.576266 |
| 5 | 11_10400 | 6H | 88.9 | 1.791 | 0.016181 | 0.120236 |
| 5 | 11_20783 | 6H | 88.9 | 3.252 | 0.00056 | 0.033795 |
| 5 | 11_21025 | 6H | 89.6 | 3.34 | 0.000457 | 0.029436 |
| 5 | 11_10202 | 6H | 90.2 | 1.935 | 0.011614 | 0.105583 |
| 5 | 12_31235 | 6H | 91.8 | 1.914 | 0.01219 | 0.107049 |
| 5 | 11_11294 | 6H | 93.7 | 0.871 | 0.134586 | 0.298874 |
| 5 | 11_20728 | 6H | 93.7 | 2.524 | 0.002992 | 0.07023 |
| 5 | 11_10595 | 6H | 94.7 | 0.103 | 0.78886 | 0.575737 |
| 5 | 11_10978 | 6H | 94.7 | 0.402 | 0.396278 | 0.465699 |
| 5 | 11_20972 | 6H | 94.7 | 0.25 | 0.562341 | 0.524345 |
| 5 | 12_31432 | 6H | 94.7 | 0.296 | 0.505825 | 0.501441 |
| 5 | 11_10734 | 6H | 96.7 | 0.546 | 0.284446 | 0.408284 |
| 5 | 11_10015 | 6H | 97.4 | 0.683 | 0.207491 | 0.366779 |
| 5 | 11_10139 | 6H | 97.4 | 0.241 | 0.574117 | 0.527183 |
| 5 | 11_20118 | 6H | 97.4 | 0.121 | 0.756833 | 0.572021 |
| 5 | 11_20531 | 6H | 97.4 | 0.548 | 0.283139 | 0.407012 |
| 5 | 12_30151 | 6H | 97.4 | 0.485 | 0.327341 | 0.428623 |
| 5 | 12_31044 | 6H | 97.4 | 0.517 | 0.304089 | 0.42242 |
| 5 | 12_31048 | 6H | 97.4 | 0.517 | 0.304089 | 0.42242 |
| 5 | 12_31049 | 6H | 97.4 | 0.517 | 0.304089 | 0.42242 |
| 5 | 12_31353 | 6H | 97.4 | 0.241 | 0.574117 | 0.527183 |
| 5 | 12_31115 | 6H | 100.1 | 0.11 | 0.776247 | 0.575737 |
| 5 | 11_20379 | 6H | 101.4 | 0.571 | 0.268534 | 0.39847 |
| 5 | 11_20036 | 6H | 105.6 | 0.62 | 0.239883 | 0.384291 |
| 5 | 11_20467 | 6H | 105.6 | 0.227 | 0.592925 | 0.531816 |
| 5 | 11_20725 | 6H | 105.6 | 0.227 | 0.592925 | 0.531816 |
| 5 | 11_21271 | 6H | 105.6 | 0.203 | 0.626614 | 0.547295 |
| 5 | 11_20355 | 6H | 110.3 | 0.349 | 0.447713 | 0.488139 |
| 5 | 12_30734 | 6H | 110.3 | 0.713 | 0.193642 | 0.352276 |
| 5 | 12_20448 | 6H | 111 | 0.197 | 0.635331 | 0.548015 |
| 5 | 11_10239 | 6H | 112.3 | 3.636 | 0.000231 | 0.017866 |
| 5 | 11_11534 | 6H | 112.3 | 0.301 | 0.500035 | 0.501441 |
| 5 | 11_20558 | 6H | 112.3 | 1.83 | 0.014791 | 0.117657 |
| 5 | 11_20733 | 6H | 112.3 | 0.301 | 0.500035 | 0.501441 |
| 5 | 12_31495 | 6H | 112.3 | 0.233 | 0.58479 | 0.528444 |
| 5 | 11_10645 | 6H | 118.3 | 0.033 | 0.92683 | 0.609889 |
| 5 | 12_31277 | 6H | 118.3 | 0.176 | 0.666807 | 0.551014 |
| 5 | 11_10107 | 6H | 119 | 0.45 | 0.354813 | 0.440552 |
| 5 | 11_10175 | 6H | 119 | 0.337 | 0.460257 | 0.495109 |
| 5 | 12_10051 | 6H | 119 | 0.273 | 0.533335 | 0.51366 |
| 5 | 12_31392 | 6H | 119 | 0.709 | 0.195434 | 0.353538 |
| 5 | 11_21455 | 6H | 119.7 | 0.221 | 0.601174 | 0.536722 |
| 5 | 11_21467 | 6H | 119.7 | 0.541 | 0.28774 | 0.4091 |
| 5 | 12_30057 | 6H | 121.2 | 0.087 | 0.818465 | 0.576266 |
| 5 | 12_31126 | 6H | 121.2 | 0.064 | 0.862979 | 0.588311 |
| 5 | 11_20005 | 6H | 122.5 | 0.141 | 0.72277 | 0.562092 |
| 5 | 11_10748 | 6H | 123.8 | 0.178 | 0.663743 | 0.551014 |
| 5 | 11_20211 | 6H | 123.8 | 0.08 | 0.831764 | 0.579378 |
| 5 | 11_10828 | 6H | 124.8 | 0.004 | 0.990832 | 0.62314 |
| 5 | 11_20687 | 6H | 124.8 | 0.126 | 0.74817 | 0.568601 |
| 5 | 11_20868 | 6H | 124.8 | 0.16 | 0.691831 | 0.557388 |
| 5 | 12_30414 | 6H | 124.8 | 0.571 | 0.268534 | 0.39847 |
| 5 | 12_31283 | 6H | 124.8 | 0.036 | 0.92045 | 0.608176 |
| 5 | 12_31498 | 6H | 126.2 | 0.163 | 0.687068 | 0.554476 |
| 5 | 11_21112 | 6H | 126.8 | 0.198 | 0.63387 | 0.548015 |
| 5 | 11_10390 | 6H | 128.5 | 0.669 | 0.214289 | 0.371639 |
| 5 | 11_11111 | 6H | 128.5 | 0.283 | 0.521195 | 0.505496 |
| 5 | 11_20537 | 6H | 129.4 | 0.406 | 0.392645 | 0.46312 |
| 5 | 12_30627 | 6H | 129.4 | 2.365 | 0.004315 | 0.076668 |
| 5 | 12_30956 | 6H | 129.4 | 1.564 | 0.02729 | 0.156293 |
| 5 | 11_10209 | 7H | 0 | 0.106 | 0.78343 | 0.575737 |
| 5 | 11_10547 | 7H | 0 | 0.143 | 0.719449 | 0.561313 |
| 5 | 11_10700 | 7H | 0 | 0.095 | 0.803526 | 0.576266 |
| 5 | 11_10949 | 7H | 0 | 0.084 | 0.824138 | 0.577808 |
| 5 | 11_10956 | 7H | 0 | 0.913 | 0.12218 | 0.295467 |
| 5 | 11_10971 | 7H | 0 | 0.232 | 0.586138 | 0.529168 |
| 5 | 11_11222 | 7H | 0 | 0.035 | 0.922571 | 0.60833 |
| 5 | 11_11343 | 7H | 0 | 0.189 | 0.647143 | 0.548015 |
| 5 | 11_20076 | 7H | 0 | 0.065 | 0.860994 | 0.587788 |
| 5 | 11_20303 | 7H | 0 | 0.19 | 0.645654 | 0.548015 |
| 5 | 11_20311 | 7H | 0 | 0.44 | 0.363078 | 0.444889 |
| 5 | 11_20584 | 7H | 0 | 0.316 | 0.483059 | 0.501441 |
| 5 | 11_20623 | 7H | 0 | 0.044 | 0.90365 | 0.603265 |
| 5 | 11_20691 | 7H | 0 | 0.305 | 0.49545 | 0.501441 |
| 5 | 11_20998 | 7H | 0 | 1.828 | 0.014859 | 0.117657 |
| 5 | 11_21419 | 7H | 0 | 0.34 | 0.457088 | 0.493901 |
| 5 | 11_21516 | 7H | 0 | 0.349 | 0.447713 | 0.488139 |
| 5 | 12_30296 | 7H | 0 | 0.016 | 0.963829 | 0.617004 |
| 5 | 12_30959 | 7H | 0 | 0.12 | 0.758578 | 0.572021 |
| 5 | 11_10682 | 7H | 0.6 | 0.047 | 0.897429 | 0.601607 |
| 5 | 11_21443 | 7H | 0.6 | 0.286 | 0.517607 | 0.505059 |
| 5 | 12_30472 | 7H | 0.6 | 0.133 | 0.736207 | 0.56349 |
| 5 | 11_10121 | 7H | 1.9 | 0.187 | 0.65013 | 0.548015 |
| 5 | 11_21307 | 7H | 1.9 | 0.155 | 0.699842 | 0.559354 |
| 5 | 12_20201 | 7H | 1.9 | 0.334 | 0.463447 | 0.495326 |
| 5 | 11_20710 | 7H | 3.3 | 0.204 | 0.625173 | 0.547295 |
| 5 | 12_31350 | 7H | 3.3 | 0.391 | 0.406443 | 0.473041 |
| 5 | 11_20242 | 7H | 4.9 | 0.261 | 0.548277 | 0.51925 |
| 5 | 12_30836 | 7H | 4.9 | 0.308 | 0.49204 | 0.501441 |
| 5 | 12_31173 | 7H | 4.9 | 0.021 | 0.952796 | 0.615425 |
| 5 | 11_11495 | 7H | 6.1 | 0.154 | 0.701455 | 0.559354 |
| 5 | 12_10406 | 7H | 6.8 | 0.252 | 0.559758 | 0.524151 |
| 5 | 12_11433 | 7H | 6.8 | 0.071 | 0.849181 | 0.583019 |
| 5 | 11_20534 | 7H | 6.8 | 0.283 | 0.521195 | 0.505496 |
| 5 | 11_20307 | 7H | 9.8 | 0.552 | 0.280543 | 0.405696 |
| 5 | 12_31450 | 7H | 12.4 | 0.203 | 0.626614 | 0.547295 |
| 5 | 12_20227 | 7H | 12.5 | 0.397 | 0.400867 | 0.469378 |
| 5 | 11_10841 | 7H | 15 | 0.928 | 0.118032 | 0.295467 |
| 5 | 11_10851 | 7H | 15 | 0.256 | 0.554626 | 0.522191 |
| 5 | 12_30851 | 7H | 15 | 1.232 | 0.058614 | 0.21611 |
| 5 | 12_31285 | 7H | 15 | 0.196 | 0.636796 | 0.548015 |
| 5 | 11_20755 | 7H | 15.9 | 0.209 | 0.618016 | 0.543224 |
| 5 | 11_20014 | 7H | 17.2 | 0.542 | 0.287078 | 0.4091 |
| 5 | 11_21437 | 7H | 17.2 | 0.542 | 0.287078 | 0.4091 |
| 5 | 11_20722 | 7H | 19.2 | 0.911 | 0.122744 | 0.295467 |
| 5 | 11_21050 | 7H | 19.9 | 0.01 | 0.977237 | 0.619024 |
| 5 | 11_10025 | 7H | 21.1 | 0.823 | 0.150314 | 0.303139 |
| 5 | 12_30329 | 7H | 28.3 | 0.721 | 0.190108 | 0.348471 |
| 5 | 11_10965 | 7H | 29.8 | 0.188 | 0.648634 | 0.548015 |
| 5 | 12_30780 | 7H | 29.8 | 0.971 | 0.106906 | 0.281274 |
| 5 | 11_10920 | 7H | 31.8 | 1.26 | 0.054954 | 0.207366 |
| 5 | 11_20162 | 7H | 31.8 | 0.397 | 0.400867 | 0.469378 |
| 5 | 11_20507 | 7H | 31.8 | 0.206 | 0.6223 | 0.545996 |
| 5 | 12_30040 | 7H | 31.8 | 0.513 | 0.306902 | 0.422921 |
| 5 | 12_30063 | 7H | 31.8 | 0.513 | 0.306902 | 0.422921 |
| 5 | 11_10451 | 7H | 32.6 | 0.103 | 0.78886 | 0.575737 |
| 5 | 11_20758 | 7H | 32.6 | 0.188 | 0.648634 | 0.548015 |
| 5 | 12_30702 | 7H | 32.6 | 0.29 | 0.512861 | 0.501441 |
| 5 | 11_10232 | 7H | 34.8 | 0.357 | 0.439542 | 0.486365 |
| 5 | 11_20192 | 7H | 34.8 | 0.052 | 0.887156 | 0.598877 |
| 5 | 11_20993 | 7H | 34.8 | 0.468 | 0.340408 | 0.432398 |
| 5 | 12_30083 | 7H | 34.8 | 1.104 | 0.078705 | 0.245254 |
| 5 | 12_30141 | 7H | 34.8 | 0.188 | 0.648634 | 0.548015 |
| 5 | 12_30219 | 7H | 34.8 | 0.774 | 0.168267 | 0.319344 |
| 5 | 12_30242 | 7H | 36.8 | 0.27 | 0.537032 | 0.515166 |
| 5 | 11_20126 | 7H | 37.5 | 0.109 | 0.778037 | 0.575737 |
| 5 | 12_30893 | 7H | 37.5 | 1.575 | 0.026607 | 0.156293 |
| 5 | 12_30894 | 7H | 37.5 | 0.609 | 0.246037 | 0.388352 |
| 5 | 12_30895 | 7H | 37.5 | 0.839 | 0.144877 | 0.299306 |
| 5 | 12_10218 | 7H | 39 | 1.032 | 0.092897 | 0.263768 |
| 5 | 11_10056 | 7H | 40.2 | 0.074 | 0.843335 | 0.580657 |
| 5 | 12_31305 | 7H | 41 | 1.249 | 0.056364 | 0.210222 |
| 5 | 11_10576 | 7H | 41.9 | 1.531 | 0.029444 | 0.158747 |
| 5 | 11_10327 | 7H | 42.6 | 1.033 | 0.092683 | 0.263768 |
| 5 | 12_30065 | 7H | 42.6 | 0.138 | 0.72778 | 0.562773 |
| 5 | 12_10979 | 7H | 43.4 | 0.894 | 0.127644 | 0.295467 |
| 5 | 12_30143 | 7H | 45.7 | 1.878 | 0.013243 | 0.111245 |
| 5 | 11_21326 | 7H | 49.7 | 0.277 | 0.528445 | 0.509968 |
| 5 | 12_30528 | 7H | 49.7 | 0.247 | 0.566239 | 0.525949 |
| 5 | 11_20249 | 7H | 52.8 | 0.439 | 0.363915 | 0.444889 |
| 5 | 12_30545 | 7H | 53.6 | 0.135 | 0.732825 | 0.56349 |
| 5 | 12_30752 | 7H | 53.6 | 0.692 | 0.203236 | 0.361557 |
| 5 | 11_10772 | 7H | 54.4 | 0.267 | 0.540754 | 0.516685 |
| 5 | 11_20790 | 7H | 55.6 | 0.175 | 0.668344 | 0.551812 |
| 5 | 12_10696 | 7H | 55.6 | 0.807 | 0.155955 | 0.309349 |
| 5 | 11_10726 | 7H | 56.8 | 0.001 | 0.9977 | 0.62583 |
| 5 | 11_20113 | 7H | 56.8 | 0.334 | 0.463447 | 0.495326 |
| 5 | 12_30290 | 7H | 57.6 | 0.799 | 0.158855 | 0.309382 |
| 5 | 12_30576 | 7H | 58.6 | 0.119 | 0.760326 | 0.572021 |
| 5 | 11_11014 | 7H | 60.7 | 0.051 | 0.889201 | 0.599838 |
| 5 | 11_10346 | 7H | 61.3 | 0.005 | 0.988553 | 0.622518 |
| 5 | 12_10403 | 7H | 61.3 | 0.033 | 0.92683 | 0.609889 |
| 5 | 11_10721 | 7H | 62.9 | 0.393 | 0.404576 | 0.472005 |
| 5 | 11_20975 | 7H | 63.7 | 0.12 | 0.758578 | 0.572021 |
| 5 | 12_30149 | 7H | 63.7 | 0.265 | 0.54325 | 0.517706 |
| 5 | 11_20671 | 7H | 68.5 | 0.451 | 0.353997 | 0.440105 |
| 5 | 11_21270 | 7H | 68.5 | 0.094 | 0.805378 | 0.576266 |
| 5 | 11_10431 | 7H | 71.1 | 0.016 | 0.963829 | 0.617004 |
| 5 | 11_11028 | 7H | 71.1 | 1.654 | 0.022182 | 0.141673 |
| 5 | 11_20060 | 7H | 71.1 | 1.725 | 0.018836 | 0.134786 |
| 5 | 12_30125 | 7H | 71.7 | 0.821 | 0.151008 | 0.303904 |
| 5 | 11_10153 | 7H | 73.8 | 0.011 | 0.97499 | 0.619024 |
| 5 | 11_10299 | 7H | 73.8 | 0.155 | 0.699842 | 0.559354 |
| 5 | 11_11122 | 7H | 73.8 | 0.132 | 0.737904 | 0.56349 |
| 5 | 12_30496 | 7H | 73.8 | 0.248 | 0.564937 | 0.52575 |
| 5 | 12_30997 | 7H | 73.8 | 0.483 | 0.328852 | 0.428623 |
| 5 | 12_31452 | 7H | 73.8 | 0.101 | 0.792501 | 0.575737 |
| 5 | 11_20885 | 7H | 74.5 | 0.561 | 0.274789 | 0.400977 |
| 5 | 12_31120 | 7H | 74.5 | 0.591 | 0.256448 | 0.398123 |
| 5 | 12_30344 | 7H | 76.1 | 0.609 | 0.246037 | 0.388352 |
| 5 | 11_10256 | 7H | 77.8 | 0.654 | 0.22182 | 0.376586 |
| 5 | 11_10394 | 7H | 77.8 | 0.097 | 0.799834 | 0.575737 |
| 5 | 11_10924 | 7H | 77.8 | 0.295 | 0.506991 | 0.501441 |
| 5 | 11_20828 | 7H | 77.8 | 0.097 | 0.799834 | 0.575737 |
| 5 | 11_20879 | 7H | 77.8 | 0.378 | 0.418794 | 0.477069 |
| 5 | 11_20893 | 7H | 77.8 | 0.097 | 0.799834 | 0.575737 |
| 5 | 11_20911 | 7H | 77.8 | 0.097 | 0.799834 | 0.575737 |
| 5 | 12_10222 | 7H | 77.8 | 0.127 | 0.746449 | 0.567771 |
| 5 | 12_10459 | 7H | 77.8 | 0.087 | 0.818465 | 0.576266 |
| 5 | 12_10698 | 7H | 77.8 | 0.295 | 0.506991 | 0.501441 |
| 5 | 12_30053 | 7H | 77.8 | 0.097 | 0.799834 | 0.575737 |
| 5 | 12_30411 | 7H | 77.8 | 0.097 | 0.799834 | 0.575737 |
| 5 | 12_30486 | 7H | 77.8 | 0.097 | 0.799834 | 0.575737 |
| 5 | 12_30492 | 7H | 77.8 | 0.164 | 0.685488 | 0.553663 |
| 5 | 12_30544 | 7H | 77.8 | 0.097 | 0.799834 | 0.575737 |
| 5 | 12_30760 | 7H | 77.8 | 0.285 | 0.5188 | 0.505202 |
| 5 | 12_30794 | 7H | 77.8 | 0.295 | 0.506991 | 0.501441 |
| 5 | 12_31227 | 7H | 77.8 | 0.097 | 0.799834 | 0.575737 |
| 5 | 11_10073 | 7H | 78.2 | 0.561 | 0.274789 | 0.400977 |
| 5 | 12_30004 | 7H | 78.2 | 0.087 | 0.818465 | 0.576266 |
| 5 | 12_30481 | 7H | 78.2 | 0.17 | 0.676083 | 0.553663 |
| 5 | 11_10055 | 7H | 79.6 | 0.04 | 0.912011 | 0.605916 |
| 5 | 11_10370 | 7H | 79.6 | 0.097 | 0.799834 | 0.575737 |
| 5 | 11_10773 | 7H | 79.6 | 0.561 | 0.274789 | 0.400977 |
| 5 | 11_11145 | 7H | 79.6 | 0.561 | 0.274789 | 0.400977 |
| 5 | 11_11219 | 7H | 79.6 | 0.147 | 0.712853 | 0.560762 |
| 5 | 11_11352 | 7H | 79.6 | 0.561 | 0.274789 | 0.400977 |
| 5 | 11_20200 | 7H | 79.6 | 0.097 | 0.799834 | 0.575737 |
| 5 | 11_20460 | 7H | 79.6 | 0.401 | 0.397192 | 0.466205 |
| 5 | 11_21302 | 7H | 79.6 | 0.087 | 0.818465 | 0.576266 |
| 5 | 11_21335 | 7H | 79.6 | 0.829 | 0.148252 | 0.300864 |
| 5 | 11_21494 | 7H | 79.6 | 0.097 | 0.799834 | 0.575737 |
| 5 | 12_10581 | 7H | 79.6 | 0.378 | 0.418794 | 0.477069 |
| 5 | 12_10713 | 7H | 79.6 | 0.04 | 0.912011 | 0.605916 |
| 5 | 12_11146 | 7H | 79.6 | 0.378 | 0.418794 | 0.477069 |
| 5 | 12_30389 | 7H | 79.6 | 0.097 | 0.799834 | 0.575737 |
| 5 | 12_30445 | 7H | 79.6 | 0.097 | 0.799834 | 0.575737 |
| 5 | 12_30449 | 7H | 79.6 | 0.561 | 0.274789 | 0.400977 |
| 5 | 12_30475 | 7H | 79.6 | 0.147 | 0.712853 | 0.560762 |
| 5 | 12_30550 | 7H | 79.6 | 0.097 | 0.799834 | 0.575737 |
| 5 | 12_30563 | 7H | 79.6 | 0.105 | 0.785236 | 0.575737 |
| 5 | 12_30574 | 7H | 79.6 | 0.105 | 0.785236 | 0.575737 |
| 5 | 12_30589 | 7H | 79.6 | 0.654 | 0.22182 | 0.376586 |
| 5 | 12_30600 | 7H | 79.6 | 0.087 | 0.818465 | 0.576266 |
| 5 | 12_30621 | 7H | 79.6 | 0.097 | 0.799834 | 0.575737 |
| 5 | 12_30835 | 7H | 79.6 | 0.088 | 0.816582 | 0.576266 |
| 5 | 12_31140 | 7H | 79.6 | 0.561 | 0.274789 | 0.400977 |
| 5 | 12_31215 | 7H | 79.6 | 0.097 | 0.799834 | 0.575737 |
| 5 | 12_31345 | 7H | 79.6 | 0.654 | 0.22182 | 0.376586 |
| 5 | 12_31418 | 7H | 79.6 | 0.087 | 0.818465 | 0.576266 |
| 5 | 11_10534 | 7H | 80.9 | 0.451 | 0.353997 | 0.440105 |
| 5 | 11_20205 | 7H | 83.4 | 1.16 | 0.069183 | 0.228092 |
| 5 | 12_10125 | 7H | 83.4 | 1.156 | 0.069823 | 0.229419 |
| 5 | 12_10982 | 7H | 83.4 | 1.16 | 0.069183 | 0.228092 |
| 5 | 12_30506 | 7H | 83.4 | 1.152 | 0.070469 | 0.229977 |
| 5 | 11_10442 | 7H | 84.9 | 1.935 | 0.011614 | 0.105583 |
| 5 | 11_10531 | 7H | 84.9 | 1.935 | 0.011614 | 0.105583 |
| 5 | 11_11239 | 7H | 84.9 | 1.935 | 0.011614 | 0.105583 |
| 5 | 11_11445 | 7H | 84.9 | 0.183 | 0.656145 | 0.54909 |
| 5 | 11_20880 | 7H | 84.9 | 0.183 | 0.656145 | 0.54909 |
| 5 | 12_30998 | 7H | 84.9 | 0.183 | 0.656145 | 0.54909 |
| 5 | 11_20042 | 7H | 86.4 | 0.943 | 0.114025 | 0.291397 |
| 5 | 11_20230 | 7H | 86.4 | 1.474 | 0.033574 | 0.16851 |
| 5 | 11_20896 | 7H | 86.4 | 0.01 | 0.977237 | 0.619024 |
| 5 | 11_21330 | 7H | 86.4 | 0.077 | 0.837529 | 0.580657 |
| 5 | 12_30199 | 7H | 86.4 | 1.604 | 0.024889 | 0.151306 |
| 5 | 12_31137 | 7H | 86.4 | 0.01 | 0.977237 | 0.619024 |
| 5 | 12_31199 | 7H | 86.4 | 0.883 | 0.130918 | 0.295467 |
| 5 | 11_21409 | 7H | 87.2 | 0.125 | 0.749894 | 0.568601 |
| 5 | 11_10143 | 7H | 88 | 0.125 | 0.749894 | 0.568601 |
| 5 | 11_10303 | 7H | 88 | 0.125 | 0.749894 | 0.568601 |
| 5 | 11_20083 | 7H | 88 | 0.165 | 0.683912 | 0.553663 |
| 5 | 12_11437 | 7H | 91.8 | 0.887 | 0.129718 | 0.295467 |
| 5 | 12_20685 | 7H | 91.8 | 0.887 | 0.129718 | 0.295467 |
| 5 | 12_30301 | 7H | 91.8 | 0.887 | 0.129718 | 0.295467 |
| 5 | 11_21201 | 7H | 98.5 | 0.395 | 0.402717 | 0.470974 |
| 5 | 11_21448 | 7H | 98.5 | 0.142 | 0.721108 | 0.561313 |
| 5 | 12_30806 | 7H | 99.7 | 0.325 | 0.473151 | 0.501167 |
| 5 | 12_31395 | 7H | 99.7 | 0.118 | 0.762079 | 0.572448 |
| 5 | 11_20808 | 7H | 101.3 | 0.152 | 0.704693 | 0.559354 |
| 5 | 11_20103 | 7H | 102.8 | 0.541 | 0.28774 | 0.4091 |
| 5 | 12_30630 | 7H | 103.6 | 0.438 | 0.364754 | 0.444889 |
| 5 | 12_31294 | 7H | 103.6 | 0.33 | 0.467735 | 0.497065 |
| 5 | 12_31440 | 7H | 103.6 | 0.438 | 0.364754 | 0.444889 |
| 5 | 11_10169 | 7H | 104.8 | 0.833 | 0.146893 | 0.299363 |
| 5 | 12_31261 | 7H | 107.1 | 0.111 | 0.774462 | 0.575737 |
| 5 | 11_20824 | 7H | 107.9 | 0.554 | 0.279254 | 0.404437 |
| 5 | 12_20684 | 7H | 109.7 | 0.435 | 0.367282 | 0.445072 |
| 5 | 11_10853 | 7H | 111 | 0.068 | 0.855067 | 0.585811 |
| 5 | 11_20092 | 7H | 111 | 0.366 | 0.430527 | 0.483574 |
| 5 | 11_20385 | 7H | 111 | 0.083 | 0.826038 | 0.577808 |
| 5 | 11_10563 | 7H | 112.5 | 0.047 | 0.897429 | 0.601607 |
| 5 | 11_20570 | 7H | 112.5 | 0.099 | 0.796159 | 0.575737 |
| 5 | 12_10241 | 7H | 112.5 | 0.264 | 0.544503 | 0.517706 |
| 5 | 12_30362 | 7H | 114.8 | 0.084 | 0.824138 | 0.577808 |
| 5 | 11_20247 | 7H | 116.3 | 0.211 | 0.615177 | 0.541714 |
| 5 | 12_30797 | 7H | 117 | 2.826 | 0.001493 | 0.060261 |
| 5 | 12_30368 | 7H | 118.9 | 0.286 | 0.517607 | 0.505059 |
| 5 | 12_30164 | 7H | 119.5 | 1.429 | 0.037239 | 0.173996 |
| 5 | 11_11243 | 7H | 122.1 | 1.152 | 0.070469 | 0.229977 |
| 5 | 11_20354 | 7H | 125.2 | 0.982 | 0.104232 | 0.278289 |
| 5 | 12_31535 | 7H | 125.2 | 0.834 | 0.146555 | 0.299306 |
| 5 | 11_21229 | 7H | 128.4 | 0.057 | 0.877001 | 0.594932 |
| 5 | 11_21209 | 7H | 129.9 | 1.562 | 0.027416 | 0.156293 |
| 5 | 11_10861 | 7H | 133.8 | 2.235 | 0.005821 | 0.085199 |
| 5 | 11_21104 | 7H | 133.8 | 1.071 | 0.084918 | 0.253372 |
| 5 | 11_10078 | 7H | 136.6 | 0.011 | 0.97499 | 0.619024 |
| 5 | 11_10797 | 7H | 136.6 | 0.05 | 0.891251 | 0.600382 |
| 5 | 12_30380 | 7H | 138.2 | 0.05 | 0.891251 | 0.600382 |
| 5 | 11_10885 | 7H | 139.7 | 0.313 | 0.486407 | 0.501441 |
| 5 | 11_10454 | 7H | 140.2 | 0.144 | 0.717794 | 0.561313 |
| 5 | 11_20847 | 7H | 140.2 | 0.419 | 0.381066 | 0.453896 |
| 5 | 12_10973 | 7H | 140.2 | 0.184 | 0.654636 | 0.54909 |
| 5 | 11_10687 | 7H | 141 | 0.419 | 0.381066 | 0.453896 |
| 5 | 11_20139 | 7H | 141.8 | 0.502 | 0.314775 | 0.426853 |
| 5 | 12_30761 | 7H | 141.8 | 0.502 | 0.314775 | 0.426853 |
| 5 | 12_31325 | 7H | 143.7 | 0.422 | 0.378443 | 0.452445 |
| 5 | 11_11440 | 7H | 144.4 | 0.54 | 0.288403 | 0.4091 |
| 5 | 11_20452 | 7H | 144.4 | 0.036 | 0.92045 | 0.608176 |
| 5 | 11_21363 | 7H | 144.4 | 0.207 | 0.620869 | 0.545236 |
| 5 | 12_30593 | 7H | 144.4 | 0.036 | 0.92045 | 0.608176 |
| 5 | 12_31166 | 7H | 144.4 | 0.008 | 0.981748 | 0.621473 |
| 5 | 11_11012 | 7H | 147.5 | 0.696 | 0.201372 | 0.359567 |
| 5 | 11_10130 | 7H | 147.5 | 0.75 | 0.177828 | 0.332001 |
| 5 | 11_10896 | 7H | 148.2 | 0.299 | 0.502343 | 0.501441 |
| 5 | 11_11275 | 7H | 149 | 0.1 | 0.794328 | 0.575737 |
| 5 | 11_20962 | 7H | 149.8 | 0.193 | 0.64121 | 0.548015 |
| 5 | 11_21086 | 7H | 159.3 | 2.671 | 0.002133 | 0.064391 |
| 5 | 11_10174 | 7H | 166.6 | 0.236 | 0.580764 | 0.527723 |
| 5 | 11_20365 | 7H | 166.6 | 0.2 | 0.630957 | 0.548015 |
| 5 | 11_11276 | UNK | 0 | 0.745 | 0.179887 | 0.332258 |
| 5 | 11_20044 | UNK | 0 | 0.423 | 0.377572 | 0.452445 |
| 5 | 11_20125 | UNK | 0 | 0.256 | 0.554626 | 0.522191 |
| 5 | 11_20131 | UNK | 0 | 0.039 | 0.914113 | 0.606479 |
| 5 | 11_20153 | UNK | 0 | 0.285 | 0.5188 | 0.505202 |
| 5 | 11_20309 | UNK | 0 | 2.118 | 0.007621 | 0.090761 |
| 5 | 11_20339 | UNK | 0 | 0.382 | 0.414954 | 0.477069 |
| 5 | 11_20479 | UNK | 0 | 0.144 | 0.717794 | 0.561313 |
| 5 | 11_20922 | UNK | 0 | 1.817 | 0.015241 | 0.118322 |
| 5 | 11_21095 | UNK | 0 | 1.031 | 0.093111 | 0.263768 |
| 5 | 11_21390 | UNK | 0 | 0.228 | 0.591562 | 0.53158 |
| 5 | 12_10032 | UNK | 0 | 0.293 | 0.509331 | 0.501441 |
| 5 | 12_10149 | UNK | 0 | 0.77 | 0.169824 | 0.321667 |
| 5 | 12_10151 | UNK | 0 | 1.239 | 0.057677 | 0.21347 |
| 5 | 12_10367 | UNK | 0 | 0.647 | 0.225424 | 0.380698 |
| 5 | 12_10393 | UNK | 0 | 0.647 | 0.225424 | 0.380698 |
| 5 | 12_10420 | UNK | 0 | 0.113 | 0.770904 | 0.575737 |
| 5 | 12_10430 | UNK | 0 | 1.019 | 0.095719 | 0.265704 |
| 5 | 12_10623 | UNK | 0 | 0.113 | 0.770904 | 0.575737 |
| 5 | 12_10704 | UNK | 0 | 0.298 | 0.503501 | 0.501441 |
| 5 | 12_10735 | UNK | 0 | 3.14 | 0.000724 | 0.038878 |
| 5 | 12_11368 | UNK | 0 | 0.49 | 0.323594 | 0.428623 |
| 5 | 12_11408 | UNK | 0 | 0.423 | 0.377572 | 0.452445 |
| 5 | 12_11468 | UNK | 0 | 0.099 | 0.796159 | 0.575737 |
| 5 | 12_20295 | UNK | 0 | 0.799 | 0.158855 | 0.309382 |
| 5 | 12_20359 | UNK | 0 | 0.507 | 0.311172 | 0.424565 |
| 5 | 12_20416 | UNK | 0 | 1.284 | 0.052 | 0.203014 |
| 5 | 12_20775 | UNK | 0 | 0.136 | 0.731139 | 0.562773 |
| 5 | 12_20803 | UNK | 0 | 0.843 | 0.143549 | 0.299306 |
| 5 | 12_20985 | UNK | 0 | 0.624 | 0.237684 | 0.382034 |
| 5 | 12_21157 | UNK | 0 | 0.489 | 0.32434 | 0.428623 |
| 5 | 12_21288 | UNK | 0 | 0.153 | 0.703072 | 0.559354 |
| 5 | 12_30008 | UNK | 0 | 0.508 | 0.310456 | 0.424187 |
| 5 | 12_30050 | UNK | 0 | 0.916 | 0.121339 | 0.295467 |
| 5 | 12_30129 | UNK | 0 | 1.312 | 0.048753 | 0.198335 |
| 5 | 12_30147 | UNK | 0 | 3.407 | 0.000392 | 0.02703 |
| 5 | 12_30166 | UNK | 0 | 0.143 | 0.719449 | 0.561313 |
| 5 | 12_30186 | UNK | 0 | 0.326 | 0.472063 | 0.500563 |
| 5 | 12_30221 | UNK | 0 | 0.02 | 0.954993 | 0.615425 |
| 5 | 12_30222 | UNK | 0 | 0.944 | 0.113763 | 0.291397 |
| 5 | 12_30254 | UNK | 0 | 0.01 | 0.977237 | 0.619024 |
| 5 | 12_30260 | UNK | 0 | 0.571 | 0.268534 | 0.39847 |
| 5 | 12_30285 | UNK | 0 | 0.416 | 0.383707 | 0.456407 |
| 5 | 12_30351 | UNK | 0 | 0.534 | 0.292415 | 0.412117 |
| 5 | 12_30409 | UNK | 0 | 0.836 | 0.145881 | 0.299306 |
| 5 | 12_30424 | UNK | 0 | 0.431 | 0.370681 | 0.447597 |
| 5 | 12_30477 | UNK | 0 | 3.619 | 0.00024 | 0.017866 |
| 5 | 12_30502 | UNK | 0 | 5.859 | 1.38E-06 | 0.000654 |
| 5 | 12_30594 | UNK | 0 | 0.495 | 0.31989 | 0.427404 |
| 5 | 12_30597 | UNK | 0 | 0.908 | 0.123595 | 0.295467 |
| 5 | 12_30603 | UNK | 0 | 0.306 | 0.494311 | 0.501441 |
| 5 | 12_30622 | UNK | 0 | 0.883 | 0.130918 | 0.295467 |
| 5 | 12_30646 | UNK | 0 | 0.296 | 0.505825 | 0.501441 |
| 5 | 12_30653 | UNK | 0 | 0.571 | 0.268534 | 0.39847 |
| 5 | 12_30655 | UNK | 0 | 0.262 | 0.547016 | 0.518565 |
| 5 | 12_30716 | UNK | 0 | 1.366 | 0.043053 | 0.18546 |
| 5 | 12_30748 | UNK | 0 | 0.14 | 0.724436 | 0.562092 |
| 5 | 12_30793 | UNK | 0 | 0.251 | 0.561048 | 0.524151 |
| 5 | 12_30819 | UNK | 0 | 0.587 | 0.258821 | 0.398123 |
| 5 | 12_30822 | UNK | 0 | 0.252 | 0.559758 | 0.524151 |
| 5 | 12_30877 | UNK | 0 | 1.563 | 0.027353 | 0.156293 |
| 5 | 12_30908 | UNK | 0 | 0.474 | 0.335738 | 0.430707 |
| 5 | 12_30916 | UNK | 0 | 0.369 | 0.427563 | 0.481944 |
| 5 | 12_30917 | UNK | 0 | 0.278 | 0.52723 | 0.509814 |
| 5 | 12_30939 | UNK | 0 | 0.474 | 0.335738 | 0.430707 |
| 5 | 12_30941 | UNK | 0 | 0.143 | 0.719449 | 0.561313 |
| 5 | 12_30949 | UNK | 0 | 1.601 | 0.025061 | 0.151306 |
| 5 | 12_30957 | UNK | 0 | 1.225 | 0.059566 | 0.218787 |
| 5 | 12_30967 | UNK | 0 | 1.291 | 0.051168 | 0.203014 |
| 5 | 12_30981 | UNK | 0 | 0.359 | 0.437522 | 0.485243 |
| 5 | 12_30982 | UNK | 0 | 0.332 | 0.465586 | 0.495326 |
| 5 | 12_30999 | UNK | 0 | 0.173 | 0.671429 | 0.55247 |
| 5 | 12_31054 | UNK | 0 | 0.095 | 0.803526 | 0.576266 |
| 5 | 12_31055 | UNK | 0 | 0.155 | 0.699842 | 0.559354 |
| 5 | 12_31084 | UNK | 0 | 0.011 | 0.97499 | 0.619024 |
| 5 | 12_31113 | UNK | 0 | 2.307 | 0.004932 | 0.078822 |
| 5 | 12_31124 | UNK | 0 | 0.953 | 0.11143 | 0.286939 |
| 5 | 12_31128 | UNK | 0 | 0.138 | 0.72778 | 0.562773 |
| 5 | 12_31151 | UNK | 0 | 0.403 | 0.395367 | 0.465194 |
| 5 | 12_31170 | UNK | 0 | 0.921 | 0.11995 | 0.295467 |
| 5 | 12_31181 | UNK | 0 | 0.501 | 0.315501 | 0.426853 |
| 5 | 12_31200 | UNK | 0 | 0.17 | 0.676083 | 0.553663 |
| 5 | 12_31219 | UNK | 0 | 0.349 | 0.447713 | 0.488139 |
| 5 | 12_31230 | UNK | 0 | 1.066 | 0.085901 | 0.253764 |
| 5 | 12_31240 | UNK | 0 | 0.619 | 0.240436 | 0.384539 |
| 5 | 12_31267 | UNK | 0 | 0.776 | 0.167494 | 0.319131 |
| 5 | 12_31275 | UNK | 0 | 0.756 | 0.175388 | 0.32962 |
| 5 | 12_31279 | UNK | 0 | 0.654 | 0.22182 | 0.376586 |
| 5 | 12_31310 | UNK | 0 | 0.192 | 0.642688 | 0.548015 |
| 5 | 12_31326 | UNK | 0 | 0.364 | 0.432514 | 0.483574 |
| 5 | 12_31327 | UNK | 0 | 2.122 | 0.007551 | 0.090761 |
| 5 | 12_31333 | UNK | 0 | 0.105 | 0.785236 | 0.575737 |
| 5 | 12_31357 | UNK | 0 | 0.006 | 0.98628 | 0.622303 |
| 5 | 12_31408 | UNK | 0 | 0.173 | 0.671429 | 0.55247 |
| 5 | 12_31410 | UNK | 0 | 0.479 | 0.331895 | 0.428623 |
| 5 | 12_31411 | UNK | 0 | 0.111 | 0.774462 | 0.575737 |
| 5 | 12_31414 | UNK | 0 | 0.908 | 0.123595 | 0.295467 |
| 5 | 12_31431 | UNK | 0 | 0.836 | 0.145881 | 0.299306 |
| 5 | 12_31479 | UNK | 0 | 0.01 | 0.977237 | 0.619024 |
| 5 | 12_31511 | UNK | 0 | 0.055 | 0.881049 | 0.596421 |
| 5 | 12_31519 | UNK | 0 | 1.221 | 0.060117 | 0.219145 |
| 5 | 12_31521 | UNK | 0 | 0.174 | 0.669885 | 0.55247 |
| 5 | 12_31523 | UNK | 0 | 0.076 | 0.83946 | 0.580657 |
| 5 | 12_31528 | UNK | 0 | 0.658 | 0.219786 | 0.376442 |
