# Supplementary material for: A Genome-Wide Association Study for Culm Cellulose Content in Barley Reveals Candidate Genes Co-Expressed with Members of the CELLULOSE SYNTHASE A Gene Family
Source: PLoS One. 2015 Jul 8;10(7):e0130890. doi: 10.1371/journal.pone.0130890 (PMC4496100; doi:10.1371/journal.pone.0130890)
Supplement: S5 Table — Regression analysis of the rice orthologs of these genes is included for comparison. (DOCX) [file pone.0130890.s007.docx]

| Group | Candidate | Cell wall gene | R Square | *p* - value | |
| --- | --- | --- | --- | --- | --- |
| 1 | *HvCobra1* | *HvCesA1* | 0.81 | 0.000 | *** |
|  |  | *HvCesA2* | 0.75 | 0.000 | *** |
|  |  | *HvCesA6* | 0.79 | 0.000 | *** |
| 1 | *HvCslF6* | *HvCesA1* | 0.84 | 0.000 | *** |
|  |  | *HvCesA2* | 0.88 | 0.000 | *** |
|  |  | *HvCesA6* | 0.93 | 0.000 | *** |
| 1 | *HvCesA9* | *HvCesA1* | 0.91 | 0.000 | *** |
|  |  | *HvCesA2* | 0.91 | 0.000 | *** |
|  |  | *HvCesA6* | 0.95 | 0.000 | *** |
| 2 | *HvGT1* | *HvCesA4* | 0.66 | 0.006 | ** |
|  |  | *HvCesA8* | 0.61 | 0.012 | * |
|  |  | *HvCesA7* | 0.70 | 0.003 | ** |
| 2 | *HvChitinase* | *HvCesA4* | 0.75 | 0.001 | *** |
|  |  | *HvCesA8* | 0.70 | 0.000 | *** |
|  |  | *HvCesA7* | 0.77 | 0.000 | *** |
| 1 | *OsCobra1* | *OsCesA8* | 0.94 | 0.000 | *** |
|  |  | *OsCesA3* | 0.74 | 0.001 | *** |
|  |  | *OsCesA1* | 0.85 | 0.000 | *** |
| 1 | *OsCslF6* | *OsCesA8* | 0.73 | 0.001 | *** |
|  |  | *OsCesA3* | 0.83 | 0.000 | *** |
|  |  | *OsCesA1* | 0.59 | 0.017 | * |
| 2 | *OsGT1* | *OsCesA7* | 0.62 | 0.010 | ** |
|  |  | *OsCesA9* | 0.65 | 0.007 | ** |
|  |  | *OsCesA4* | 0.49 | 0.053 | * |
| 2 | *OsChitinase1* | *OsCesA7* | n/a | n/a |  |
|  |  | *OsCesA9* | n/a | n/a |  |
|  |  | *OsCesA4* | n/a | n/a |  |
|  |  |  |  |  |  |
|  |  |  |  |  |  |
| **0.05** | * |  |  |  |  |
| **0.01** | ** |  |  |  |  |
| **0.001** | *** |  |  |  |  |
