# Supplementary material for: A Genome-Wide Association Study for Culm Cellulose Content in Barley Reveals Candidate Genes Co-Expressed with Members of the CELLULOSE SYNTHASE A Gene Family
Source: PLoS One. 2015 Jul 8;10(7):e0130890. doi: 10.1371/journal.pone.0130890 (PMC4496100; doi:10.1371/journal.pone.0130890)
Supplement: S6 Table — Marker positions are as described in Comadran et al [36]. (DOCX) [file pone.0130890.s008.docx]

|  |  |  |  |  |  |
| --- | --- | --- | --- | --- | --- |
| Marker | Chr | cM | - log 10( p) | p-value | q-value |
| 11_20149 | 1H | 0 | 0.14 | 0.73 | 1 |
| 11_20373 | 1H | 0 | 0.60 | 0.25 | 1 |
| 12_30969 | 1H | 0 | 0.45 | 0.35 | 1 |
| 11_11223 | 1H | 0.75 | 1.46 | 0.03 | 1 |
| 11_10895 | 1H | 0.77 | 0.01 | 0.97 | 1 |
| 11_21354 | 1H | 0.77 | 0.01 | 0.98 | 1 |
| 12_11311 | 1H | 0.95 | 0.11 | 0.78 | 1 |
| 11_20502 | 1H | 1.51 | 0.49 | 0.32 | 1 |
| 11_21067 | 1H | 1.51 | 1.36 | 0.04 | 1 |
| 11_10419 | 1H | 3.75 | 0.47 | 0.34 | 1 |
| 12_10410 | 1H | 3.75 | 0.06 | 0.88 | 1 |
| 12_31144 | 1H | 3.75 | 0.27 | 0.54 | 1 |
| 12_10636 | 1H | 4.51 | 0.83 | 0.15 | 1 |
| 12_11011 | 1H | 6.03 | 0.86 | 0.14 | 1 |
| 12_30933 | 1H | 6.03 | 0.68 | 0.21 | 1 |
| 11_21174 | 1H | 8.29 | 0.20 | 0.63 | 1 |
| 11_21226 | 1H | 8.77 | 0.09 | 0.81 | 1 |
| 12_30817 | 1H | 10.67 | 0.06 | 0.87 | 1 |
| 12_30950 | 1H | 10.67 | 0.00 | 0.99 | 1 |
| 12_31149 | 1H | 10.67 | 0.04 | 0.92 | 1 |
| 12_30918 | 1H | 11.42 | 0.16 | 0.70 | 1 |
| 12_30951 | 1H | 11.42 | 0.17 | 0.67 | 1 |
| 12_30588 | 1H | 13.05 | 0.29 | 0.51 | 1 |
| 11_10332 | 1H | 15.4 | 1.49 | 0.03 | 1 |
| 11_10775 | 1H | 17.26 | 0.03 | 0.93 | 1 |
| 11_20749 | 1H | 17.26 | 0.06 | 0.87 | 1 |
| 12_30948 | 1H | 17.26 | 0.31 | 0.49 | 1 |
| 11_10030 | 1H | 18.05 | 0.20 | 0.63 | 1 |
| 11_20371 | 1H | 18.05 | 0.31 | 0.49 | 1 |
| 11_20712 | 1H | 20.82 | 0.26 | 0.55 | 1 |
| 11_10873 | 1H | 20.89 | 0.31 | 0.49 | 1 |
| 11_10186 | 1H | 23.86 | 0.27 | 0.53 | 1 |
| 11_10757 | 1H | 23.86 | 0.00 | 0.99 | 1 |
| 11_10744 | 1H | 26.11 | 0.39 | 0.40 | 1 |
| 11_21048 | 1H | 26.58 | 0.14 | 0.73 | 1 |
| 12_31276 | 1H | 27.35 | 0.19 | 0.65 | 1 |
| 11_20617 | 1H | 33.61 | 0.26 | 0.55 | 1 |
| 11_10760 | 1H | 34.83 | 0.12 | 0.77 | 1 |
| 11_10814 | 1H | 35.45 | 0.29 | 0.52 | 1 |
| 11_10238 | 1H | 36.95 | 0.02 | 0.96 | 1 |
| 12_11357 | 1H | 36.95 | 0.00 | 1.00 | 1 |
| 12_31177 | 1H | 36.95 | 0.17 | 0.68 | 1 |
| 11_10764 | 1H | 40.99 | 0.07 | 0.85 | 1 |
| 12_11498 | 1H | 40.99 | 0.03 | 0.94 | 1 |
| 12_30336 | 1H | 41.76 | 0.15 | 0.71 | 1 |
| 11_10275 | 1H | 42.52 | 0.07 | 0.86 | 1 |
| 11_10597 | 1H | 42.52 | 0.07 | 0.84 | 1 |
| 11_20514 | 1H | 42.52 | 0.05 | 0.89 | 1 |
| 12_11266 | 1H | 42.52 | 0.00 | 1.00 | 1 |
| 12_30268 | 1H | 42.52 | 0.00 | 1.00 | 1 |
| 11_21134 | 1H | 43.28 | 0.00 | 1.00 | 1 |
| 12_10314 | 1H | 45.13 | 0.06 | 0.87 | 1 |
| 11_10259 | 1H | 47.47 | 0.17 | 0.67 | 1 |
| 11_10294 | 1H | 47.47 | 0.03 | 0.93 | 1 |
| 11_10526 | 1H | 47.47 | 0.16 | 0.69 | 1 |
| 12_10235 | 1H | 47.47 | 0.01 | 0.97 | 1 |
| 12_30683 | 1H | 47.47 | 0.22 | 0.60 | 1 |
| 12_30796 | 1H | 47.47 | 0.02 | 0.95 | 1 |
| 12_31467 | 1H | 47.47 | 0.22 | 0.60 | 1 |
| 11_10957 | 1H | 49.34 | 0.00 | 0.99 | 1 |
| 11_11064 | 1H | 49.34 | 0.02 | 0.95 | 1 |
| 11_20855 | 1H | 49.34 | 0.02 | 0.95 | 1 |
| 12_11301 | 1H | 49.34 | 0.10 | 0.80 | 1 |
| 11_10470 | 1H | 49.7 | 0.01 | 0.97 | 1 |
| 11_11162 | 1H | 49.7 | 0.00 | 1.00 | 1 |
| 12_10159 | 1H | 49.7 | 0.01 | 0.98 | 1 |
| 12_30436 | 1H | 49.7 | 0.06 | 0.87 | 1 |
| 12_30438 | 1H | 49.7 | 0.01 | 0.98 | 1 |
| 12_30762 | 1H | 49.7 | 0.06 | 0.86 | 1 |
| 11_10438 | 1H | 50 | 0.04 | 0.91 | 1 |
| 11_11287 | 1H | 50 | 0.02 | 0.97 | 1 |
| 11_11478 | 1H | 50 | 0.02 | 0.96 | 1 |
| 11_20660 | 1H | 50 | 0.03 | 0.92 | 1 |
| 12_30498 | 1H | 50 | 0.00 | 1.00 | 1 |
| 12_30562 | 1H | 50 | 0.04 | 0.91 | 1 |
| 11_20427 | 1H | 50.6 | 0.05 | 0.90 | 1 |
| 11_20698 | 1H | 50.6 | 0.08 | 0.82 | 1 |
| 11_20757 | 1H | 50.6 | 0.04 | 0.92 | 1 |
| 11_21193 | 1H | 50.6 | 0.06 | 0.88 | 1 |
| 12_10300 | 1H | 50.6 | 0.06 | 0.88 | 1 |
| 12_10506 | 1H | 50.6 | 0.05 | 0.89 | 1 |
| 12_10938 | 1H | 50.6 | 0.36 | 0.43 | 1 |
| 12_11036 | 1H | 50.6 | 0.00 | 1.00 | 1 |
| 12_21408 | 1H | 50.6 | 0.00 | 1.00 | 1 |
| 12_30043 | 1H | 50.6 | 0.06 | 0.88 | 1 |
| 12_30404 | 1H | 50.6 | 0.06 | 0.87 | 1 |
| 12_30462 | 1H | 50.6 | 0.00 | 1.00 | 1 |
| 12_31208 | 1H | 50.6 | 0.02 | 0.96 | 1 |
| 12_31381 | 1H | 50.6 | 0.01 | 0.97 | 1 |
| 11_11484 | 1H | 51.23 | 0.03 | 0.92 | 1 |
| 11_21357 | 1H | 51.7 | 0.19 | 0.64 | 1 |
| 11_10833 | 1H | 52.46 | 0.04 | 0.91 | 1 |
| 11_20810 | 1H | 52.46 | 0.09 | 0.81 | 1 |
| 11_20912 | 1H | 52.46 | 0.05 | 0.89 | 1 |
| 11_21000 | 1H | 52.46 | 0.18 | 0.66 | 1 |
| 11_21312 | 1H | 52.46 | 0.01 | 0.97 | 1 |
| 12_11169 | 1H | 52.46 | 0.13 | 0.73 | 1 |
| 12_30059 | 1H | 52.46 | 0.00 | 1.00 | 1 |
| 12_30350 | 1H | 52.46 | 0.06 | 0.86 | 1 |
| 12_30592 | 1H | 52.46 | 0.01 | 0.98 | 1 |
| 12_31134 | 1H | 52.46 | 0.05 | 0.89 | 1 |
| 12_31272 | 1H | 52.46 | 0.03 | 0.92 | 1 |
| 12_11107 | 1H | 53.22 | 0.00 | 1.00 | 1 |
| 12_30522 | 1H | 53.22 | 0.11 | 0.77 | 1 |
| 11_11359 | 1H | 54.73 | 0.05 | 0.88 | 1 |
| 11_21217 | 1H | 54.73 | 0.14 | 0.73 | 1 |
| 12_11217 | 1H | 54.73 | 0.00 | 1.00 | 1 |
| 12_30672 | 1H | 54.73 | 0.11 | 0.79 | 1 |
| 12_30786 | 1H | 54.73 | 0.02 | 0.97 | 1 |
| 11_10075 | 1H | 55.49 | 0.00 | 1.00 | 1 |
| 11_10293 | 1H | 55.49 | 0.04 | 0.92 | 1 |
| 11_10520 | 1H | 55.49 | 0.06 | 0.87 | 1 |
| 11_10933 | 1H | 55.49 | 0.00 | 0.99 | 1 |
| 11_11256 | 1H | 55.49 | 0.09 | 0.82 | 1 |
| 11_20798 | 1H | 55.49 | 0.01 | 0.97 | 1 |
| 11_21361 | 1H | 55.49 | 0.01 | 0.97 | 1 |
| 12_30110 | 1H | 55.49 | 0.01 | 0.98 | 1 |
| 12_30243 | 1H | 55.49 | 0.09 | 0.82 | 1 |
| 12_30348 | 1H | 55.49 | 0.09 | 0.81 | 1 |
| 12_30406 | 1H | 55.49 | 0.02 | 0.95 | 1 |
| 12_30478 | 1H | 55.49 | 0.02 | 0.95 | 1 |
| 12_30499 | 1H | 55.49 | 0.03 | 0.94 | 1 |
| 12_30694 | 1H | 55.49 | 0.01 | 0.98 | 1 |
| 12_30750 | 1H | 55.49 | 0.04 | 0.91 | 1 |
| 12_30710 | 1H | 56.25 | 0.05 | 0.89 | 1 |
| 11_10324 | 1H | 57.01 | 0.00 | 0.99 | 1 |
| 12_10198 | 1H | 57.77 | 0.08 | 0.83 | 1 |
| 11_21053 | 1H | 58.9 | 0.07 | 0.86 | 1 |
| 12_30343 | 1H | 58.9 | 0.03 | 0.92 | 1 |
| 11_10552 | 1H | 59.71 | 0.04 | 0.91 | 1 |
| 11_10768 | 1H | 59.71 | 0.01 | 0.98 | 1 |
| 11_21333 | 1H | 59.71 | 0.00 | 1.00 | 1 |
| 12_11444 | 1H | 59.71 | 0.23 | 0.59 | 1 |
| 12_30820 | 1H | 59.71 | 0.16 | 0.68 | 1 |
| 12_30821 | 1H | 59.71 | 0.16 | 0.69 | 1 |
| 11_20997 | 1H | 60.19 | 0.02 | 0.95 | 1 |
| 11_20095 | 1H | 60.77 | 0.24 | 0.57 | 1 |
| 12_10201 | 1H | 60.77 | 0.42 | 0.38 | 1 |
| 12_21463 | 1H | 60.77 | 0.00 | 1.00 | 1 |
| 12_30753 | 1H | 60.77 | 0.09 | 0.81 | 1 |
| 11_10617 | 1H | 61.53 | 0.82 | 0.15 | 1 |
| 11_10798 | 1H | 61.53 | 0.81 | 0.16 | 1 |
| 11_11049 | 1H | 61.53 | 0.81 | 0.15 | 1 |
| 12_30304 | 1H | 61.53 | 0.81 | 0.15 | 1 |
| 12_30744 | 1H | 61.53 | 0.00 | 1.00 | 1 |
| 12_21131 | 1H | 62.78 | 0.00 | 1.00 | 1 |
| 12_31179 | 1H | 63.54 | 0.05 | 0.90 | 1 |
| 12_31401 | 1H | 63.54 | 0.05 | 0.90 | 1 |
| 11_20432 | 1H | 64.3 | 0.03 | 0.93 | 1 |
| 11_20642 | 1H | 64.91 | 0.50 | 0.31 | 1 |
| 11_21431 | 1H | 64.91 | 0.03 | 0.94 | 1 |
| 11_10516 | 1H | 65.53 | 0.29 | 0.51 | 1 |
| 11_10043 | 1H | 65.96 | 0.23 | 0.59 | 1 |
| 11_10002 | 1H | 66.7 | 0.15 | 0.71 | 1 |
| 11_11367 | 1H | 66.7 | 0.22 | 0.60 | 1 |
| 11_20956 | 1H | 66.7 | 0.03 | 0.94 | 1 |
| 11_21219 | 1H | 66.7 | 0.21 | 0.61 | 1 |
| 12_10960 | 1H | 66.7 | 0.00 | 1.00 | 1 |
| 12_11062 | 1H | 66.7 | 0.00 | 1.00 | 1 |
| 12_30295 | 1H | 66.7 | 0.01 | 0.98 | 1 |
| 12_31464 | 1H | 66.7 | 0.14 | 0.73 | 1 |
| 11_20290 | 1H | 69.53 | 0.02 | 0.95 | 1 |
| 12_10166 | 1H | 69.53 | 0.45 | 0.36 | 1 |
| 12_20696 | 1H | 69.53 | 0.00 | 1.00 | 1 |
| 12_30298 | 1H | 69.53 | 0.76 | 0.17 | 1 |
| 12_30505 | 1H | 70.29 | 0.17 | 0.68 | 1 |
| 11_10686 | 1H | 71.43 | 0.07 | 0.86 | 1 |
| 11_11075 | 1H | 71.43 | 0.37 | 0.43 | 1 |
| 12_21176 | 1H | 71.43 | 0.00 | 1.00 | 1 |
| 12_30742 | 1H | 71.43 | 0.10 | 0.80 | 1 |
| 12_11267 | 1H | 72.43 | 0.69 | 0.20 | 1 |
| 11_10006 | 1H | 73.94 | 0.66 | 0.22 | 1 |
| 11_21126 | 1H | 73.94 | 0.69 | 0.20 | 1 |
| 11_10279 | 1H | 75.45 | 0.64 | 0.23 | 1 |
| 11_20121 | 1H | 75.45 | 0.12 | 0.76 | 1 |
| 11_20990 | 1H | 75.45 | 0.06 | 0.87 | 1 |
| 11_20657 | 1H | 77.29 | 0.39 | 0.40 | 1 |
| 11_10466 | 1H | 80.26 | 0.07 | 0.85 | 1 |
| 12_30072 | 1H | 83.3 | 0.00 | 0.99 | 1 |
| 11_11037 | 1H | 84.69 | 0.01 | 0.97 | 1 |
| 12_11463 | 1H | 85.43 | 0.04 | 0.92 | 1 |
| 11_10434 | 1H | 86.19 | 0.01 | 0.97 | 1 |
| 12_30204 | 1H | 86.19 | 0.04 | 0.91 | 1 |
| 12_11144 | 1H | 87.62 | 0.03 | 0.94 | 1 |
| 11_10471 | 1H | 88.23 | 0.03 | 0.94 | 1 |
| 11_10830 | 1H | 88.23 | 0.14 | 0.73 | 1 |
| 11_20434 | 1H | 88.23 | 0.12 | 0.75 | 1 |
| 11_20550 | 1H | 88.23 | 0.23 | 0.58 | 1 |
| 11_21192 | 1H | 88.23 | 0.03 | 0.93 | 1 |
| 11_11189 | 1H | 90.97 | 0.15 | 0.70 | 1 |
| 11_20792 | 1H | 90.97 | 0.12 | 0.77 | 1 |
| 11_21446 | 1H | 92.04 | 0.01 | 0.98 | 1 |
| 12_10535 | 1H | 92.04 | 0.14 | 0.72 | 1 |
| 11_20475 | 1H | 92.8 | 0.02 | 0.95 | 1 |
| 12_30546 | 1H | 92.8 | 0.27 | 0.54 | 1 |
| 11_10433 | 1H | 93.95 | 0.06 | 0.86 | 1 |
| 12_11008 | 1H | 93.95 | 0.15 | 0.70 | 1 |
| 12_31163 | 1H | 93.95 | 0.02 | 0.96 | 1 |
| 11_21373 | 1H | 95.42 | 0.02 | 0.96 | 1 |
| 11_10396 | 1H | 96.92 | 0.17 | 0.68 | 1 |
| 11_20769 | 1H | 96.92 | 0.05 | 0.89 | 1 |
| 11_11277 | 1H | 97.68 | 0.03 | 0.94 | 1 |
| 12_31319 | 1H | 97.68 | 0.14 | 0.72 | 1 |
| 11_10522 | 1H | 99.19 | 0.46 | 0.35 | 1 |
| 11_20169 | 1H | 99.95 | 0.54 | 0.29 | 1 |
| 11_20754 | 1H | 99.95 | 0.56 | 0.27 | 1 |
| 11_10357 | 1H | 100.69 | 0.18 | 0.66 | 1 |
| 11_20267 | 1H | 101.45 | 0.16 | 0.69 | 1 |
| 11_20909 | 1H | 101.45 | 0.15 | 0.70 | 1 |
| 12_11173 | 1H | 101.45 | 0.05 | 0.89 | 1 |
| 12_20187 | 1H | 101.45 | 0.16 | 0.69 | 1 |
| 12_20613 | 1H | 101.45 | 0.00 | 1.00 | 1 |
| 12_21020 | 1H | 101.45 | 0.00 | 1.00 | 1 |
| 12_31152 | 1H | 101.45 | 0.18 | 0.66 | 1 |
| 12_30191 | 1H | 104.35 | 0.00 | 1.00 | 1 |
| 11_20780 | 1H | 105.1 | 0.16 | 0.69 | 1 |
| 11_20921 | 1H | 105.85 | 0.02 | 0.95 | 1 |
| 11_20625 | 1H | 106.6 | 0.02 | 0.96 | 1 |
| 11_20220 | 1H | 107.55 | 0.12 | 0.77 | 1 |
| 11_20844 | 1H | 108.31 | 0.12 | 0.77 | 1 |
| 11_20021 | 1H | 109.82 | 0.11 | 0.77 | 1 |
| 12_30532 | 1H | 109.82 | 0.06 | 0.87 | 1 |
| 11_10789 | 1H | 112.54 | 0.51 | 0.31 | 1 |
| 12_31526 | 1H | 112.91 | 0.23 | 0.59 | 1 |
| 12_30014 | 1H | 113.3 | 0.04 | 0.91 | 1 |
| 11_21392 | 1H | 114.84 | 0.15 | 0.71 | 1 |
| 12_10905 | 1H | 114.84 | 0.10 | 0.80 | 1 |
| 12_11409 | 1H | 115.92 | 0.00 | 1.00 | 1 |
| 11_10729 | 1H | 116.31 | 0.17 | 0.68 | 1 |
| 11_10338 | 1H | 117.8 | 0.09 | 0.81 | 1 |
| 11_10854 | 1H | 117.8 | 0.05 | 0.89 | 1 |
| 11_20959 | 1H | 117.8 | 0.12 | 0.75 | 1 |
| 12_30257 | 1H | 117.8 | 0.19 | 0.64 | 1 |
| 11_10911 | 1H | 120.51 | 0.10 | 0.80 | 1 |
| 11_20908 | 1H | 121.12 | 0.38 | 0.42 | 1 |
| 11_21038 | 1H | 121.12 | 0.01 | 0.98 | 1 |
| 12_21172 | 1H | 121.12 | 0.15 | 0.71 | 1 |
| 11_10586 | 1H | 121.77 | 0.32 | 0.47 | 1 |
| 12_11457 | 1H | 122.53 | 0.00 | 1.00 | 1 |
| 12_11443 | 1H | 123.75 | 0.03 | 0.93 | 1 |
| 11_10722 | 1H | 125.27 | 0.25 | 0.56 | 1 |
| 11_21140 | 1H | 126.01 | 0.05 | 0.89 | 1 |
| 11_11481 | 1H | 126.48 | 0.25 | 0.56 | 1 |
| 12_10207 | 1H | 126.48 | 0.26 | 0.55 | 1 |
| 12_30403 | 1H | 126.48 | 0.07 | 0.86 | 1 |
| 12_31377 | 1H | 126.48 | 0.07 | 0.86 | 1 |
| 11_10644 | 1H | 127.1 | 0.12 | 0.76 | 1 |
| 12_10808 | 1H | 127.38 | 0.25 | 0.56 | 1 |
| 12_31387 | 1H | 127.38 | 0.10 | 0.80 | 1 |
| 11_11038 | 1H | 128.14 | 0.11 | 0.78 | 1 |
| 11_20133 | 1H | 128.14 | 0.04 | 0.90 | 1 |
| 12_10693 | 1H | 128.14 | 0.01 | 0.97 | 1 |
| 11_10903 | 1H | 129.64 | 0.00 | 1.00 | 1 |
| 11_20383 | 1H | 131.15 | 0.03 | 0.93 | 1 |
| 11_10782 | 1H | 131.89 | 0.60 | 0.25 | 1 |
| 12_11271 | 1H | 132.53 | 0.00 | 1.00 | 1 |
| 12_21105 | 1H | 134.05 | 0.00 | 1.00 | 1 |
| 11_10041 | 1H | 135.56 | 0.10 | 0.79 | 1 |
| 11_11105 | 1H | 135.56 | 0.03 | 0.93 | 1 |
| 11_11509 | 1H | 135.56 | 0.07 | 0.85 | 1 |
| 11_20603 | 1H | 135.56 | 0.68 | 0.21 | 1 |
| 11_21384 | 1H | 135.56 | 0.83 | 0.15 | 1 |
| 12_11496 | 1H | 135.56 | 0.03 | 0.93 | 1 |
| 12_20599 | 1H | 135.56 | 1.01 | 0.10 | 1 |
| 12_30277 | 1H | 135.56 | 1.01 | 0.10 | 1 |
| 12_30517 | 1H | 135.56 | 1.02 | 0.10 | 1 |
| 11_20594 | 1H | 136.31 | 0.05 | 0.90 | 1 |
| 11_20138 | 1H | 137.83 | 0.07 | 0.84 | 1 |
| 11_20840 | 1H | 137.83 | 0.10 | 0.79 | 1 |
| 11_10590 | 1H | 138.31 | 0.02 | 0.95 | 1 |
| 11_20915 | 1H | 138.31 | 0.02 | 0.95 | 1 |
| 12_10746 | 1H | 138.31 | 0.01 | 0.98 | 1 |
| 12_30231 | 1H | 138.31 | 0.06 | 0.87 | 1 |
| 11_20772 | 1H | 139.79 | 0.10 | 0.79 | 1 |
| 11_10443 | 1H | 140.53 | 0.02 | 0.95 | 1 |
| 12_20429 | 1H | 140.53 | 0.00 | 1.00 | 1 |
| 12_30934 | 1H | 140.53 | 0.02 | 0.95 | 1 |
| 12_31081 | 1H | 140.53 | 0.04 | 0.91 | 1 |
| 11_10017 | 2H | 0 | 0.19 | 0.64 | 1 |
| 11_10057 | 2H | 0 | 0.20 | 0.63 | 1 |
| 11_10101 | 2H | 0 | 2.10 | 0.01 | 1 |
| 11_10194 | 2H | 0 | 0.20 | 0.63 | 1 |
| 11_10243 | 2H | 0 | 0.21 | 0.62 | 1 |
| 11_10352 | 2H | 0 | 0.10 | 0.79 | 1 |
| 11_10770 | 2H | 0 | 0.00 | 1.00 | 1 |
| 11_10977 | 2H | 0 | 0.18 | 0.66 | 1 |
| 11_10996 | 2H | 0 | 0.01 | 0.99 | 1 |
| 11_11346 | 2H | 0 | 0.66 | 0.22 | 1 |
| 11_20099 | 2H | 0 | 0.02 | 0.95 | 1 |
| 11_20498 | 2H | 0 | 0.15 | 0.72 | 1 |
| 11_20609 | 2H | 0 | 0.11 | 0.78 | 1 |
| 11_20631 | 2H | 0 | 0.28 | 0.53 | 1 |
| 11_20862 | 2H | 0 | 0.21 | 0.62 | 1 |
| 11_21142 | 2H | 0 | 0.08 | 0.82 | 1 |
| 11_21181 | 2H | 0 | 0.02 | 0.96 | 1 |
| 11_21184 | 2H | 0 | 0.03 | 0.94 | 1 |
| 11_21507 | 2H | 0 | 0.08 | 0.83 | 1 |
| 12_10970 | 2H | 0 | 0.03 | 0.93 | 1 |
| 12_31446 | 2H | 0 | 0.04 | 0.90 | 1 |
| 12_10718 | 2H | 2.14 | 0.00 | 1.00 | 1 |
| 12_21415 | 2H | 2.86 | 0.00 | 1.00 | 1 |
| 12_11030 | 2H | 3.57 | 0.00 | 1.00 | 1 |
| 12_31224 | 2H | 4.08 | 0.46 | 0.35 | 1 |
| 11_10326 | 2H | 6.45 | 0.53 | 0.29 | 1 |
| 11_11059 | 2H | 7.14 | 0.01 | 0.98 | 1 |
| 12_10230 | 2H | 7.14 | 0.00 | 1.00 | 1 |
| 11_21377 | 2H | 8.57 | 0.01 | 0.97 | 1 |
| 12_10592 | 2H | 8.57 | 0.33 | 0.47 | 1 |
| 12_30781 | 2H | 8.57 | 0.05 | 0.89 | 1 |
| 11_20563 | 2H | 9.28 | 0.14 | 0.73 | 1 |
| 11_21416 | 2H | 10.06 | 0.26 | 0.54 | 1 |
| 12_10502 | 2H | 10.06 | 0.01 | 0.97 | 1 |
| 12_11119 | 2H | 10.06 | 0.13 | 0.75 | 1 |
| 12_30155 | 2H | 10.06 | 0.16 | 0.70 | 1 |
| 12_30402 | 2H | 10.06 | 0.49 | 0.33 | 1 |
| 12_30775 | 2H | 10.06 | 0.00 | 1.00 | 1 |
| 12_31497 | 2H | 10.06 | 0.02 | 0.95 | 1 |
| 11_20562 | 2H | 10.94 | 0.08 | 0.84 | 1 |
| 12_30631 | 2H | 15.15 | 0.04 | 0.91 | 1 |
| 11_20107 | 2H | 17.85 | 0.07 | 0.86 | 1 |
| 11_20724 | 2H | 18.2 | 0.04 | 0.91 | 1 |
| 11_10943 | 2H | 18.32 | 0.01 | 0.97 | 1 |
| 11_11040 | 2H | 19.27 | 0.00 | 0.99 | 1 |
| 12_31284 | 2H | 19.47 | 0.08 | 0.84 | 1 |
| 11_10180 | 2H | 21.61 | 0.28 | 0.52 | 1 |
| 12_10777 | 2H | 21.61 | 0.10 | 0.80 | 1 |
| 11_10216 | 2H | 26.53 | 0.65 | 0.22 | 1 |
| 12_30871 | 2H | 26.57 | 0.03 | 0.93 | 1 |
| 12_30872 | 2H | 26.57 | 0.03 | 0.93 | 1 |
| 11_20394 | 2H | 27.29 | 0.12 | 0.75 | 1 |
| 11_21015 | 2H | 27.29 | 0.27 | 0.53 | 1 |
| 11_21261 | 2H | 28.44 | 0.00 | 0.99 | 1 |
| 11_21265 | 2H | 28.44 | 0.01 | 0.98 | 1 |
| 11_21366 | 2H | 28.44 | 0.01 | 0.99 | 1 |
| 11_21187 | 2H | 29.15 | 0.26 | 0.54 | 1 |
| 12_20593 | 2H | 30.31 | 0.18 | 0.66 | 1 |
| 11_10307 | 2H | 31.02 | 0.41 | 0.39 | 1 |
| 11_10787 | 2H | 31.02 | 0.71 | 0.20 | 1 |
| 11_10891 | 2H | 31.02 | 0.36 | 0.44 | 1 |
| 11_10987 | 2H | 31.02 | 0.69 | 0.20 | 1 |
| 12_20368 | 2H | 31.02 | 0.18 | 0.66 | 1 |
| 11_20864 | 2H | 31.72 | 1.00 | 0.10 | 1 |
| 11_11110 | 2H | 32.17 | 0.26 | 0.55 | 1 |
| 12_21049 | 2H | 32.33 | 0.00 | 1.00 | 1 |
| 12_11452 | 2H | 32.63 | 0.00 | 1.00 | 1 |
| 12_11304 | 2H | 33.02 | 0.44 | 0.36 | 1 |
| 12_10847 | 2H | 33.73 | 0.04 | 0.90 | 1 |
| 11_21304 | 2H | 33.74 | 0.13 | 0.75 | 1 |
| 11_10525 | 2H | 38.03 | 0.08 | 0.84 | 1 |
| 11_11073 | 2H | 38.48 | 0.20 | 0.63 | 1 |
| 11_10178 | 2H | 39.1 | 0.01 | 0.97 | 1 |
| 11_10399 | 2H | 39.1 | 0.14 | 0.73 | 1 |
| 11_10919 | 2H | 39.1 | 0.07 | 0.85 | 1 |
| 12_10296 | 2H | 39.1 | 0.02 | 0.95 | 1 |
| 12_20326 | 2H | 39.1 | 0.22 | 0.60 | 1 |
| 12_30420 | 2H | 39.1 | 0.03 | 0.94 | 1 |
| 12_30657 | 2H | 39.1 | 0.06 | 0.87 | 1 |
| 11_10837 | 2H | 40.5 | 0.00 | 1.00 | 1 |
| 11_21153 | 2H | 40.94 | 0.19 | 0.64 | 1 |
| 11_10648 | 2H | 41.66 | 0.01 | 0.98 | 1 |
| 12_30432 | 2H | 41.66 | 0.05 | 0.89 | 1 |
| 11_21338 | 2H | 44.84 | 0.15 | 0.70 | 1 |
| 12_11493 | 2H | 45.55 | 0.03 | 0.93 | 1 |
| 12_30363 | 2H | 45.55 | 0.23 | 0.59 | 1 |
| 12_10715 | 2H | 46.26 | 0.00 | 1.00 | 1 |
| 12_30029 | 2H | 46.26 | 0.00 | 1.00 | 1 |
| 12_30379 | 2H | 46.26 | 0.03 | 0.94 | 1 |
| 12_10486 | 2H | 46.98 | 0.00 | 1.00 | 1 |
| 12_30491 | 2H | 46.98 | 0.03 | 0.94 | 1 |
| 12_11340 | 2H | 47.69 | 0.00 | 1.00 | 1 |
| 12_20281 | 2H | 47.69 | 0.00 | 1.00 | 1 |
| 11_11061 | 2H | 49.03 | 0.25 | 0.56 | 1 |
| 12_30703 | 2H | 49.03 | 0.33 | 0.47 | 1 |
| 11_10498 | 2H | 49.07 | 0.02 | 0.96 | 1 |
| 11_11505 | 2H | 49.07 | 0.07 | 0.85 | 1 |
| 11_21005 | 2H | 50.49 | 0.16 | 0.69 | 1 |
| 11_11054 | 2H | 50.6 | 0.25 | 0.56 | 1 |
| 11_10234 | 2H | 51.75 | 0.16 | 0.69 | 1 |
| 11_10297 | 2H | 51.75 | 0.14 | 0.72 | 1 |
| 11_20674 | 2H | 51.75 | 0.16 | 0.69 | 1 |
| 12_10156 | 2H | 51.75 | 0.00 | 1.00 | 1 |
| 12_11428 | 2H | 51.75 | 0.00 | 1.00 | 1 |
| 12_20688 | 2H | 51.75 | 0.00 | 1.00 | 1 |
| 12_30604 | 2H | 51.75 | 0.27 | 0.53 | 1 |
| 11_10422 | 2H | 52.47 | 0.04 | 0.92 | 1 |
| 11_10638 | 2H | 52.47 | 0.12 | 0.75 | 1 |
| 11_11302 | 2H | 52.47 | 0.11 | 0.78 | 1 |
| 11_20929 | 2H | 52.47 | 0.04 | 0.92 | 1 |
| 12_11131 | 2H | 52.47 | 0.00 | 1.00 | 1 |
| 11_11400 | 2H | 53.53 | 0.01 | 0.98 | 1 |
| 11_11522 | 2H | 53.53 | 0.08 | 0.83 | 1 |
| 12_31474 | 2H | 53.53 | 0.07 | 0.85 | 1 |
| 11_10147 | 2H | 54.95 | 0.05 | 0.89 | 1 |
| 11_10325 | 2H | 54.95 | 0.08 | 0.83 | 1 |
| 11_10733 | 2H | 54.95 | 0.07 | 0.86 | 1 |
| 11_20387 | 2H | 54.95 | 0.05 | 0.90 | 1 |
| 11_21096 | 2H | 54.95 | 0.00 | 1.00 | 1 |
| 11_21388 | 2H | 54.95 | 0.09 | 0.82 | 1 |
| 12_10927 | 2H | 54.95 | 0.00 | 1.00 | 1 |
| 12_20234 | 2H | 54.95 | 0.07 | 0.85 | 1 |
| 12_20235 | 2H | 54.95 | 0.00 | 1.00 | 1 |
| 12_20917 | 2H | 54.95 | 0.00 | 1.00 | 1 |
| 12_30195 | 2H | 54.95 | 0.00 | 1.00 | 1 |
| 12_30259 | 2H | 54.95 | 0.08 | 0.83 | 1 |
| 12_30338 | 2H | 54.95 | 0.02 | 0.96 | 1 |
| 11_11015 | 2H | 55.67 | 0.03 | 0.93 | 1 |
| 12_11272 | 2H | 55.67 | 0.12 | 0.75 | 1 |
| 11_20748 | 2H | 56.28 | 0.19 | 0.65 | 1 |
| 11_20891 | 2H | 56.28 | 0.21 | 0.62 | 1 |
| 12_30251 | 2H | 56.28 | 0.19 | 0.64 | 1 |
| 12_10558 | 2H | 56.99 | 0.00 | 1.00 | 1 |
| 12_30557 | 2H | 56.99 | 0.00 | 1.00 | 1 |
| 11_10997 | 2H | 57.54 | 0.17 | 0.68 | 1 |
| 11_10602 | 2H | 58.24 | 0.18 | 0.66 | 1 |
| 11_10796 | 2H | 58.24 | 0.27 | 0.54 | 1 |
| 11_11133 | 2H | 58.24 | 0.10 | 0.79 | 1 |
| 11_20500 | 2H | 58.24 | 0.12 | 0.76 | 1 |
| 12_10485 | 2H | 58.24 | 0.13 | 0.74 | 1 |
| 12_30634 | 2H | 58.24 | 0.23 | 0.58 | 1 |
| 12_31288 | 2H | 58.24 | 0.19 | 0.64 | 1 |
| 11_10012 | 2H | 58.9 | 0.05 | 0.90 | 1 |
| 11_10070 | 2H | 58.9 | 0.38 | 0.41 | 1 |
| 11_10679 | 2H | 58.9 | 0.22 | 0.60 | 1 |
| 11_10947 | 2H | 58.9 | 0.00 | 1.00 | 1 |
| 11_11046 | 2H | 58.9 | 0.38 | 0.41 | 1 |
| 11_11354 | 2H | 58.9 | 0.10 | 0.80 | 1 |
| 11_20039 | 2H | 58.9 | 0.01 | 0.97 | 1 |
| 11_20160 | 2H | 58.9 | 0.08 | 0.82 | 1 |
| 11_20417 | 2H | 58.9 | 0.10 | 0.80 | 1 |
| 11_20458 | 2H | 58.9 | 0.09 | 0.81 | 1 |
| 11_20476 | 2H | 58.9 | 0.17 | 0.67 | 1 |
| 11_21286 | 2H | 58.9 | 0.27 | 0.53 | 1 |
| 12_10099 | 2H | 58.9 | 0.10 | 0.80 | 1 |
| 12_10948 | 2H | 58.9 | 0.50 | 0.31 | 1 |
| 12_11155 | 2H | 58.9 | 0.00 | 1.00 | 1 |
| 12_20181 | 2H | 58.9 | 0.00 | 1.00 | 1 |
| 12_30042 | 2H | 58.9 | 0.07 | 0.86 | 1 |
| 12_30179 | 2H | 58.9 | 0.11 | 0.77 | 1 |
| 12_30582 | 2H | 58.9 | 0.14 | 0.72 | 1 |
| 12_30772 | 2H | 58.9 | 0.00 | 1.00 | 1 |
| 12_30828 | 2H | 58.9 | 0.04 | 0.92 | 1 |
| 12_31175 | 2H | 58.9 | 0.14 | 0.72 | 1 |
| 11_10624 | 2H | 59.21 | 0.05 | 0.89 | 1 |
| 12_10154 | 2H | 59.21 | 0.00 | 1.00 | 1 |
| 12_10474 | 2H | 59.21 | 0.10 | 0.80 | 1 |
| 12_10883 | 2H | 59.21 | 0.00 | 1.00 | 1 |
| 12_11369 | 2H | 59.21 | 0.00 | 1.00 | 1 |
| 12_30206 | 2H | 59.21 | 0.00 | 1.00 | 1 |
| 12_30853 | 2H | 59.21 | 0.28 | 0.53 | 1 |
| 11_10317 | 2H | 59.9 | 0.14 | 0.72 | 1 |
| 11_10358 | 2H | 59.9 | 0.50 | 0.31 | 1 |
| 11_11178 | 2H | 59.9 | 0.14 | 0.72 | 1 |
| 11_11211 | 2H | 59.9 | 0.00 | 1.00 | 1 |
| 11_20032 | 2H | 59.9 | 0.07 | 0.85 | 1 |
| 11_20251 | 2H | 59.9 | 0.51 | 0.31 | 1 |
| 11_20669 | 2H | 59.9 | 0.02 | 0.96 | 1 |
| 12_11288 | 2H | 59.9 | 0.20 | 0.63 | 1 |
| 12_20196 | 2H | 59.9 | 0.00 | 1.00 | 1 |
| 12_21337 | 2H | 59.9 | 0.00 | 1.00 | 1 |
| 12_30068 | 2H | 59.9 | 0.20 | 0.63 | 1 |
| 12_30514 | 2H | 59.9 | 0.11 | 0.77 | 1 |
| 12_30561 | 2H | 59.9 | 0.14 | 0.72 | 1 |
| 12_31189 | 2H | 59.9 | 0.00 | 1.00 | 1 |
| 12_31218 | 2H | 59.9 | 0.00 | 1.00 | 1 |
| 11_11384 | 2H | 60.68 | 0.51 | 0.31 | 1 |
| 11_10436 | 2H | 62.82 | 0.31 | 0.49 | 1 |
| 11_10750 | 2H | 62.82 | 0.00 | 1.00 | 1 |
| 11_11206 | 2H | 62.82 | 0.00 | 1.00 | 1 |
| 11_20690 | 2H | 62.82 | 0.36 | 0.43 | 1 |
| 12_10035 | 2H | 62.82 | 0.05 | 0.89 | 1 |
| 12_10640 | 2H | 62.82 | 0.31 | 0.49 | 1 |
| 11_10191 | 2H | 63.53 | 0.02 | 0.96 | 1 |
| 11_10632 | 2H | 63.53 | 0.18 | 0.67 | 1 |
| 11_10685 | 2H | 63.53 | 0.02 | 0.95 | 1 |
| 11_10692 | 2H | 63.53 | 0.10 | 0.80 | 1 |
| 11_10909 | 2H | 63.53 | 0.06 | 0.88 | 1 |
| 11_20390 | 2H | 63.53 | 0.04 | 0.90 | 1 |
| 11_20438 | 2H | 63.53 | 0.00 | 1.00 | 1 |
| 11_20532 | 2H | 63.53 | 0.02 | 0.95 | 1 |
| 11_20585 | 2H | 63.53 | 0.05 | 0.90 | 1 |
| 11_20887 | 2H | 63.53 | 0.05 | 0.89 | 1 |
| 11_21399 | 2H | 63.53 | 0.02 | 0.96 | 1 |
| 12_11278 | 2H | 63.53 | 0.00 | 1.00 | 1 |
| 12_11324 | 2H | 63.53 | 0.05 | 0.89 | 1 |
| 12_11504 | 2H | 63.53 | 0.10 | 0.79 | 1 |
| 12_30108 | 2H | 63.53 | 0.02 | 0.95 | 1 |
| 12_30265 | 2H | 63.53 | 0.02 | 0.96 | 1 |
| 12_30275 | 2H | 63.53 | 0.10 | 0.79 | 1 |
| 12_30323 | 2H | 63.53 | 0.10 | 0.80 | 1 |
| 12_30724 | 2H | 63.53 | 0.00 | 1.00 | 1 |
| 11_11430 | 2H | 64.24 | 0.00 | 1.00 | 1 |
| 11_20374 | 2H | 64.24 | 0.20 | 0.63 | 1 |
| 12_11316 | 2H | 64.24 | 0.05 | 0.90 | 1 |
| 12_21476 | 2H | 64.24 | 0.05 | 0.89 | 1 |
| 12_31252 | 2H | 64.24 | 0.02 | 0.95 | 1 |
| 12_31256 | 2H | 64.24 | 0.18 | 0.67 | 1 |
| 11_21094 | 2H | 65.67 | 0.06 | 0.88 | 1 |
| 11_11072 | 2H | 66.12 | 0.32 | 0.48 | 1 |
| 12_11347 | 2H | 66.12 | 0.05 | 0.89 | 1 |
| 11_21166 | 2H | 66.83 | 0.04 | 0.92 | 1 |
| 11_21110 | 2H | 67.54 | 0.11 | 0.77 | 1 |
| 12_11121 | 2H | 67.54 | 0.00 | 1.00 | 1 |
| 11_10651 | 2H | 68.24 | 0.02 | 0.95 | 1 |
| 12_10545 | 2H | 69.13 | 0.17 | 0.67 | 1 |
| 12_11096 | 2H | 69.16 | 0.20 | 0.63 | 1 |
| 11_21144 | 2H | 69.25 | 0.00 | 1.00 | 1 |
| 11_10265 | 2H | 70.54 | 0.17 | 0.68 | 1 |
| 11_21258 | 2H | 70.54 | 0.18 | 0.67 | 1 |
| 11_20833 | 2H | 71.12 | 1.60 | 0.02 | 1 |
| 11_21251 | 2H | 71.12 | 0.03 | 0.94 | 1 |
| 12_10719 | 2H | 71.12 | 0.00 | 1.00 | 1 |
| 12_30205 | 2H | 71.12 | 0.04 | 0.91 | 1 |
| 12_31020 | 2H | 71.12 | 0.35 | 0.45 | 1 |
| 12_31021 | 2H | 71.12 | 0.16 | 0.69 | 1 |
| 11_21205 | 2H | 71.56 | 0.35 | 0.45 | 1 |
| 12_10717 | 2H | 71.56 | 0.35 | 0.45 | 1 |
| 11_20667 | 2H | 72.33 | 0.23 | 0.59 | 1 |
| 11_20528 | 2H | 73.04 | 0.22 | 0.61 | 1 |
| 11_11402 | 2H | 73.75 | 0.19 | 0.64 | 1 |
| 11_20947 | 2H | 73.75 | 1.37 | 0.04 | 1 |
| 12_10650 | 2H | 73.75 | 0.36 | 0.44 | 1 |
| 12_31383 | 2H | 73.75 | 0.16 | 0.69 | 1 |
| 11_20419 | 2H | 74.37 | 0.20 | 0.63 | 1 |
| 11_20960 | 2H | 74.37 | 1.48 | 0.03 | 1 |
| 12_30674 | 2H | 74.37 | 1.48 | 0.03 | 1 |
| 12_31380 | 2H | 74.37 | 1.48 | 0.03 | 1 |
| 12_31394 | 2H | 74.37 | 1.55 | 0.03 | 1 |
| 11_10952 | 2H | 75.18 | 1.03 | 0.09 | 1 |
| 11_20734 | 2H | 75.18 | 1.01 | 0.10 | 1 |
| 12_30178 | 2H | 75.89 | 0.88 | 0.13 | 1 |
| 11_10196 | 2H | 78.03 | 0.61 | 0.25 | 1 |
| 11_10818 | 2H | 78.03 | 0.80 | 0.16 | 1 |
| 11_11435 | 2H | 78.03 | 0.32 | 0.48 | 1 |
| 11_20699 | 2H | 78.03 | 0.76 | 0.17 | 1 |
| 12_11388 | 2H | 78.03 | 0.00 | 1.00 | 1 |
| 12_30696 | 2H | 78.03 | 0.66 | 0.22 | 1 |
| 12_31398 | 2H | 78.03 | 0.32 | 0.48 | 1 |
| 12_20489 | 2H | 79.19 | 0.79 | 0.16 | 1 |
| 12_31445 | 2H | 79.19 | 1.77 | 0.02 | 1 |
| 12_10859 | 2H | 81.33 | 1.81 | 0.02 | 1 |
| 12_31293 | 2H | 81.33 | 0.40 | 0.40 | 1 |
| 11_10619 | 2H | 82.75 | 0.26 | 0.55 | 1 |
| 11_10786 | 2H | 82.75 | 0.34 | 0.46 | 1 |
| 11_10823 | 2H | 82.75 | 0.32 | 0.48 | 1 |
| 11_11214 | 2H | 82.75 | 0.32 | 0.48 | 1 |
| 11_21242 | 2H | 82.75 | 0.27 | 0.53 | 1 |
| 11_11100 | 2H | 83.82 | 0.37 | 0.43 | 1 |
| 11_20781 | 2H | 83.82 | 0.14 | 0.72 | 1 |
| 11_10287 | 2H | 85.92 | 0.47 | 0.33 | 1 |
| 11_20340 | 2H | 85.92 | 0.12 | 0.75 | 1 |
| 11_10213 | 2H | 86.63 | 1.19 | 0.06 | 1 |
| 12_30896 | 2H | 86.63 | 1.17 | 0.07 | 1 |
| 12_30897 | 2H | 86.63 | 0.26 | 0.55 | 1 |
| 12_30899 | 2H | 86.63 | 1.19 | 0.06 | 1 |
| 12_30900 | 2H | 86.63 | 1.31 | 0.05 | 1 |
| 12_30901 | 2H | 86.63 | 0.43 | 0.37 | 1 |
| 12_31205 | 2H | 86.63 | 0.40 | 0.40 | 1 |
| 11_11533 | 2H | 87.33 | 0.40 | 0.40 | 1 |
| 11_10475 | 2H | 88.74 | 0.19 | 0.65 | 1 |
| 11_21037 | 2H | 88.74 | 0.06 | 0.86 | 1 |
| 11_21136 | 2H | 88.74 | 0.45 | 0.36 | 1 |
| 11_21245 | 2H | 89.32 | 0.05 | 0.90 | 1 |
| 12_31424 | 2H | 89.32 | 0.19 | 0.64 | 1 |
| 11_11058 | 2H | 90.1 | 0.90 | 0.13 | 1 |
| 11_21351 | 2H | 90.1 | 0.03 | 0.94 | 1 |
| 12_10969 | 2H | 90.1 | 0.03 | 0.94 | 1 |
| 12_20793 | 2H | 90.82 | 0.90 | 0.13 | 1 |
| 11_10214 | 2H | 93.5 | 0.50 | 0.31 | 1 |
| 11_20080 | 2H | 95.64 | 0.26 | 0.55 | 1 |
| 11_21007 | 2H | 96.25 | 0.17 | 0.68 | 1 |
| 11_10138 | 2H | 96.82 | 0.05 | 0.89 | 1 |
| 11_11307 | 2H | 96.82 | 0.01 | 0.97 | 1 |
| 11_21175 | 2H | 96.82 | 0.09 | 0.81 | 1 |
| 12_10649 | 2H | 96.82 | 0.04 | 0.92 | 1 |
| 12_11285 | 2H | 96.82 | 0.08 | 0.83 | 1 |
| 12_30216 | 2H | 96.82 | 0.09 | 0.82 | 1 |
| 11_20086 | 2H | 98.59 | 0.04 | 0.90 | 1 |
| 11_10398 | 2H | 100.37 | 0.15 | 0.71 | 1 |
| 11_10876 | 2H | 100.37 | 0.28 | 0.52 | 1 |
| 11_11250 | 2H | 100.37 | 0.26 | 0.55 | 1 |
| 12_30200 | 2H | 100.37 | 0.00 | 1.00 | 1 |
| 11_10900 | 2H | 101.78 | 0.14 | 0.72 | 1 |
| 12_11466 | 2H | 101.78 | 0.00 | 1.00 | 1 |
| 12_21527 | 2H | 101.78 | 0.00 | 1.00 | 1 |
| 12_30095 | 2H | 102.49 | 0.00 | 1.00 | 1 |
| 12_30480 | 2H | 102.49 | 0.21 | 0.61 | 1 |
| 11_21340 | 2H | 103.67 | 0.06 | 0.87 | 1 |
| 11_10630 | 2H | 105.77 | 0.01 | 0.98 | 1 |
| 11_11323 | 2H | 106.46 | 0.05 | 0.89 | 1 |
| 12_30049 | 2H | 106.46 | 0.06 | 0.88 | 1 |
| 12_30555 | 2H | 106.46 | 0.05 | 0.89 | 1 |
| 11_11094 | 2H | 108.61 | 0.32 | 0.47 | 1 |
| 11_11480 | 2H | 108.61 | 0.04 | 0.91 | 1 |
| 11_20064 | 2H | 112.91 | 0.07 | 0.85 | 1 |
| 11_10128 | 2H | 113.48 | 0.40 | 0.40 | 1 |
| 11_10731 | 2H | 113.48 | 0.09 | 0.81 | 1 |
| 11_10988 | 2H | 113.48 | 0.06 | 0.86 | 1 |
| 11_10989 | 2H | 113.48 | 0.06 | 0.86 | 1 |
| 11_10990 | 2H | 113.48 | 0.07 | 0.84 | 1 |
| 11_11043 | 2H | 113.48 | 0.09 | 0.81 | 1 |
| 11_11118 | 2H | 113.48 | 0.18 | 0.66 | 1 |
| 11_21238 | 2H | 113.48 | 0.15 | 0.71 | 1 |
| 12_31402 | 2H | 113.48 | 0.08 | 0.83 | 1 |
| 12_21396 | 2H | 113.92 | 0.17 | 0.67 | 1 |
| 11_10429 | 2H | 115.08 | 0.03 | 0.93 | 1 |
| 12_20989 | 2H | 115.08 | 0.00 | 1.00 | 1 |
| 11_11236 | 2H | 115.78 | 0.18 | 0.66 | 1 |
| 11_10538 | 2H | 116.49 | 0.40 | 0.40 | 1 |
| 11_10707 | 2H | 116.49 | 0.01 | 0.98 | 1 |
| 11_20182 | 2H | 116.49 | 0.07 | 0.85 | 1 |
| 12_30459 | 2H | 116.49 | 0.00 | 0.99 | 1 |
| 12_31095 | 2H | 116.49 | 0.13 | 0.74 | 1 |
| 11_10404 | 2H | 117.2 | 0.32 | 0.48 | 1 |
| 12_10739 | 2H | 117.7 | 0.10 | 0.79 | 1 |
| 11_10916 | 2H | 117.91 | 0.35 | 0.45 | 1 |
| 11_10780 | 2H | 119.05 | 0.14 | 0.72 | 1 |
| 12_31406 | 2H | 119.05 | 0.03 | 0.92 | 1 |
| 12_30598 | 2H | 119.31 | 0.18 | 0.66 | 1 |
| 12_31264 | 2H | 119.31 | 0.17 | 0.67 | 1 |
| 11_21220 | 2H | 120.02 | 0.18 | 0.66 | 1 |
| 11_20511 | 2H | 120.8 | 0.11 | 0.78 | 1 |
| 11_11365 | 2H | 121.5 | 0.01 | 0.97 | 1 |
| 11_20141 | 2H | 121.5 | 0.00 | 0.99 | 1 |
| 11_21315 | 2H | 121.5 | 0.16 | 0.70 | 1 |
| 12_30097 | 2H | 121.5 | 0.03 | 0.93 | 1 |
| 12_30152 | 2H | 122.21 | 0.02 | 0.95 | 1 |
| 12_30636 | 2H | 122.21 | 0.16 | 0.69 | 1 |
| 11_10446 | 2H | 125.46 | 0.24 | 0.58 | 1 |
| 11_21370 | 2H | 125.46 | 0.28 | 0.53 | 1 |
| 12_31100 | 2H | 125.46 | 0.36 | 0.43 | 1 |
| 11_20480 | 2H | 126.03 | 0.06 | 0.88 | 1 |
| 11_21440 | 2H | 126.03 | 0.25 | 0.57 | 1 |
| 11_21406 | 2H | 126.37 | 0.36 | 0.43 | 1 |
| 11_11486 | 2H | 127.06 | 0.13 | 0.74 | 1 |
| 11_21459 | 2H | 127.06 | 0.44 | 0.36 | 1 |
| 11_10109 | 2H | 127.64 | 0.37 | 0.43 | 1 |
| 12_20183 | 2H | 127.64 | 0.12 | 0.76 | 1 |
| 12_30310 | 2H | 127.64 | 0.01 | 0.97 | 1 |
| 12_30695 | 2H | 127.64 | 0.30 | 0.51 | 1 |
| 11_10656 | 2H | 128.26 | 0.11 | 0.77 | 1 |
| 11_20366 | 2H | 128.26 | 0.06 | 0.86 | 1 |
| 11_21088 | 2H | 128.26 | 0.11 | 0.78 | 1 |
| 12_30690 | 2H | 128.26 | 0.06 | 0.87 | 1 |
| 11_21125 | 2H | 129.31 | 0.12 | 0.76 | 1 |
| 11_10065 | 2H | 130.01 | 0.03 | 0.94 | 1 |
| 11_10383 | 2H | 130.01 | 0.15 | 0.71 | 1 |
| 11_20215 | 2H | 130.01 | 0.00 | 0.99 | 1 |
| 12_10164 | 2H | 130.01 | 0.13 | 0.73 | 1 |
| 12_10472 | 2H | 130.01 | 0.00 | 1.00 | 1 |
| 12_30678 | 2H | 130.01 | 0.06 | 0.88 | 1 |
| 12_30942 | 2H | 130.01 | 0.15 | 0.70 | 1 |
| 12_31268 | 2H | 130.01 | 0.14 | 0.73 | 1 |
| 11_10376 | 2H | 131.77 | 0.06 | 0.88 | 1 |
| 11_20895 | 2H | 131.77 | 0.58 | 0.26 | 1 |
| 12_10579 | 2H | 132.48 | 0.57 | 0.27 | 1 |
| 11_11227 | 2H | 133.22 | 0.17 | 0.67 | 1 |
| 11_20715 | 2H | 133.94 | 0.62 | 0.24 | 1 |
| 12_30106 | 2H | 133.94 | 0.53 | 0.30 | 1 |
| 12_30396 | 2H | 133.94 | 0.52 | 0.30 | 1 |
| 12_30248 | 2H | 136.8 | 0.17 | 0.67 | 1 |
| 11_20590 | 2H | 137.51 | 0.04 | 0.91 | 1 |
| 11_21274 | 2H | 137.51 | 0.21 | 0.62 | 1 |
| 12_30341 | 2H | 137.51 | 0.68 | 0.21 | 1 |
| 12_20027 | 2H | 138.94 | 0.00 | 1.00 | 1 |
| 12_31461 | 2H | 138.94 | 0.01 | 0.98 | 1 |
| 11_10551 | 2H | 139.65 | 0.94 | 0.11 | 1 |
| 11_10625 | 2H | 139.65 | 1.02 | 0.10 | 1 |
| 11_10826 | 2H | 139.65 | 0.03 | 0.94 | 1 |
| 11_11262 | 2H | 139.65 | 0.85 | 0.14 | 1 |
| 11_20494 | 2H | 139.65 | 0.03 | 0.94 | 1 |
| 12_30352 | 2H | 139.65 | 0.02 | 0.95 | 1 |
| 12_30914 | 2H | 139.65 | 0.01 | 0.97 | 1 |
| 12_31209 | 2H | 139.65 | 0.02 | 0.95 | 1 |
| 11_10566 | 2H | 140.27 | 0.10 | 0.79 | 1 |
| 11_10315 | 2H | 141.28 | 1.07 | 0.08 | 1 |
| 11_11023 | 2H | 141.28 | 0.19 | 0.65 | 1 |
| 12_10447 | 2H | 141.28 | 0.71 | 0.20 | 1 |
| 12_10766 | 2H | 141.28 | 0.26 | 0.55 | 1 |
| 11_21250 | 2H | 144.31 | 0.24 | 0.58 | 1 |
| 12_10487 | 2H | 144.31 | 0.15 | 0.71 | 1 |
| 12_10950 | 2H | 144.31 | 0.32 | 0.48 | 1 |
| 11_11380 | 2H | 145.03 | 1.28 | 0.05 | 1 |
| 11_20994 | 2H | 147.12 | 0.32 | 0.48 | 1 |
| 11_21346 | 2H | 147.12 | 0.17 | 0.67 | 1 |
| 12_10181 | 2H | 147.12 | 0.02 | 0.95 | 1 |
| 11_20293 | 2H | 147.94 | 0.04 | 0.92 | 1 |
| 11_21299 | 2H | 149.36 | 1.38 | 0.04 | 1 |
| 11_20943 | 2H | 149.61 | 1.65 | 0.02 | 1 |
| 11_10791 | 2H | 150.67 | 0.08 | 0.84 | 1 |
| 11_21436 | 2H | 150.67 | 0.92 | 0.12 | 1 |
| 12_30823 | 2H | 150.67 | 0.47 | 0.34 | 1 |
| 11_10072 | 2H | 151.37 | 0.61 | 0.24 | 1 |
| 12_31527 | 2H | 151.37 | 0.61 | 0.25 | 1 |
| 12_10937 | 2H | 152.79 | 0.00 | 1.00 | 1 |
| 12_31300 | 2H | 152.79 | 1.77 | 0.02 | 1 |
| 12_31506 | 2H | 152.79 | 0.01 | 0.99 | 1 |
| 11_10329 | 2H | 155.3 | 0.00 | 1.00 | 1 |
| 11_21099 | 2H | 155.3 | 0.71 | 0.20 | 1 |
| 11_21453 | 2H | 155.3 | 0.71 | 0.20 | 1 |
| 12_31180 | 2H | 155.3 | 0.07 | 0.86 | 1 |
| 11_10085 | 2H | 156.72 | 0.73 | 0.18 | 1 |
| 11_20561 | 2H | 156.72 | 0.76 | 0.17 | 1 |
| 11_20681 | 2H | 156.72 | 0.07 | 0.84 | 1 |
| 12_11050 | 2H | 156.72 | 0.01 | 0.99 | 1 |
| 12_30378 | 2H | 158.87 | 0.54 | 0.29 | 1 |
| 12_30102 | 2H | 160.29 | 1.39 | 0.04 | 1 |
| 11_10044 | 3H | 0 | 0.21 | 0.62 | 1 |
| 11_10813 | 3H | 0 | 2.61 | 0.00 | 1 |
| 11_11411 | 3H | 0 | 0.81 | 0.15 | 1 |
| 11_20222 | 3H | 0 | 0.08 | 0.84 | 1 |
| 11_20952 | 3H | 0 | 0.12 | 0.76 | 1 |
| 12_20090 | 3H | 0 | 0.00 | 1.00 | 1 |
| 12_31428 | 3H | 0 | 0.10 | 0.79 | 1 |
| 11_20797 | 3H | 2.29 | 0.17 | 0.68 | 1 |
| 11_20159 | 3H | 2.9 | 0.26 | 0.55 | 1 |
| 12_10103 | 3H | 2.9 | 1.27 | 0.05 | 1 |
| 12_31448 | 3H | 2.9 | 1.09 | 0.08 | 1 |
| 12_11434 | 3H | 3.71 | 0.00 | 1.00 | 1 |
| 11_11453 | 3H | 6.03 | 0.25 | 0.56 | 1 |
| 11_20252 | 3H | 6.03 | 0.08 | 0.84 | 1 |
| 12_31409 | 3H | 6.7 | 0.43 | 0.38 | 1 |
| 11_21190 | 3H | 8.23 | 0.13 | 0.74 | 1 |
| 11_21398 | 3H | 8.23 | 0.33 | 0.47 | 1 |
| 11_21027 | 3H | 8.86 | 0.07 | 0.85 | 1 |
| 11_20976 | 3H | 9.63 | 0.41 | 0.39 | 1 |
| 12_30818 | 3H | 9.63 | 0.21 | 0.62 | 1 |
| 11_10112 | 3H | 10.8 | 0.05 | 0.89 | 1 |
| 11_10886 | 3H | 10.8 | 0.06 | 0.87 | 1 |
| 12_11310 | 3H | 10.8 | 0.20 | 0.64 | 1 |
| 12_30910 | 3H | 10.8 | 0.35 | 0.45 | 1 |
| 11_20595 | 3H | 12.46 | 0.42 | 0.38 | 1 |
| 12_30915 | 3H | 13.23 | 0.09 | 0.81 | 1 |
| 12_10571 | 3H | 15.55 | 0.46 | 0.35 | 1 |
| 12_30113 | 3H | 15.55 | 0.22 | 0.61 | 1 |
| 11_20172 | 3H | 16.33 | 0.26 | 0.55 | 1 |
| 11_10565 | 3H | 19.15 | 0.75 | 0.18 | 1 |
| 11_20742 | 3H | 19.15 | 1.04 | 0.09 | 1 |
| 11_20982 | 3H | 22.68 | 0.10 | 0.79 | 1 |
| 12_30192 | 3H | 23.45 | 0.10 | 0.80 | 1 |
| 11_20552 | 3H | 24.22 | 0.16 | 0.70 | 1 |
| 11_20794 | 3H | 26.9 | 0.31 | 0.49 | 1 |
| 11_20455 | 3H | 28.44 | 0.13 | 0.74 | 1 |
| 12_11237 | 3H | 28.44 | 0.00 | 1.00 | 1 |
| 12_30284 | 3H | 28.44 | 0.07 | 0.85 | 1 |
| 11_10026 | 3H | 32.83 | 0.08 | 0.84 | 1 |
| 11_20607 | 3H | 32.83 | 0.60 | 0.25 | 1 |
| 12_11414 | 3H | 32.83 | 0.16 | 0.69 | 1 |
| 12_11527 | 3H | 32.83 | 0.00 | 1.00 | 1 |
| 12_21268 | 3H | 32.83 | 0.00 | 1.00 | 1 |
| 12_30571 | 3H | 32.83 | 0.24 | 0.57 | 1 |
| 12_30431 | 3H | 35.22 | 0.14 | 0.72 | 1 |
| 11_10672 | 3H | 37.17 | 0.13 | 0.74 | 1 |
| 12_30925 | 3H | 37.17 | 0.43 | 0.37 | 1 |
| 12_10968 | 3H | 38.68 | 0.76 | 0.18 | 1 |
| 11_10081 | 3H | 39.45 | 0.56 | 0.28 | 1 |
| 11_10710 | 3H | 39.45 | 0.38 | 0.42 | 1 |
| 11_10825 | 3H | 39.45 | 0.23 | 0.58 | 1 |
| 11_20410 | 3H | 39.45 | 0.23 | 0.58 | 1 |
| 12_30953 | 3H | 41 | 0.15 | 0.72 | 1 |
| 12_31159 | 3H | 41 | 0.09 | 0.81 | 1 |
| 12_31298 | 3H | 41 | 0.24 | 0.57 | 1 |
| 11_10863 | 3H | 41.68 | 0.12 | 0.77 | 1 |
| 11_20193 | 3H | 42.06 | 0.31 | 0.49 | 1 |
| 11_21145 | 3H | 42.47 | 0.08 | 0.83 | 1 |
| 11_20647 | 3H | 43.23 | 0.85 | 0.14 | 1 |
| 11_21533 | 3H | 43.23 | 0.08 | 0.83 | 1 |
| 11_11002 | 3H | 43.99 | 0.01 | 0.98 | 1 |
| 12_11117 | 3H | 44.76 | 0.00 | 1.00 | 1 |
| 12_21506 | 3H | 44.76 | 0.00 | 1.00 | 1 |
| 12_30913 | 3H | 44.76 | 0.10 | 0.80 | 1 |
| 12_30785 | 3H | 45.54 | 0.32 | 0.48 | 1 |
| 12_31475 | 3H | 45.54 | 0.16 | 0.70 | 1 |
| 11_10601 | 3H | 46.31 | 0.13 | 0.74 | 1 |
| 12_30064 | 3H | 46.31 | 0.31 | 0.50 | 1 |
| 11_20356 | 3H | 47.09 | 0.75 | 0.18 | 1 |
| 11_20719 | 3H | 48.63 | 0.75 | 0.18 | 1 |
| 11_21101 | 3H | 48.63 | 0.36 | 0.43 | 1 |
| 11_21189 | 3H | 48.63 | 0.30 | 0.50 | 1 |
| 12_30474 | 3H | 48.63 | 0.30 | 0.50 | 1 |
| 12_30609 | 3H | 48.63 | 0.06 | 0.86 | 1 |
| 12_30737 | 3H | 48.63 | 0.30 | 0.50 | 1 |
| 12_31009 | 3H | 48.63 | 0.15 | 0.70 | 1 |
| 12_31122 | 3H | 48.63 | 0.31 | 0.49 | 1 |
| 12_11069 | 3H | 49.41 | 0.00 | 1.00 | 1 |
| 12_20264 | 3H | 49.41 | 0.00 | 1.00 | 1 |
| 12_30467 | 3H | 49.41 | 0.00 | 1.00 | 1 |
| 12_11102 | 3H | 50.18 | 0.00 | 1.00 | 1 |
| 12_30583 | 3H | 50.18 | 0.33 | 0.47 | 1 |
| 12_30721 | 3H | 50.18 | 0.00 | 1.00 | 1 |
| 12_30922 | 3H | 50.96 | 0.16 | 0.70 | 1 |
| 12_30923 | 3H | 50.96 | 0.33 | 0.47 | 1 |
| 12_31015 | 3H | 50.96 | 0.33 | 0.47 | 1 |
| 11_10380 | 3H | 51.73 | 0.00 | 1.00 | 1 |
| 11_11313 | 3H | 51.73 | 0.65 | 0.22 | 1 |
| 11_21109 | 3H | 51.73 | 0.11 | 0.77 | 1 |
| 11_21197 | 3H | 51.73 | 0.37 | 0.43 | 1 |
| 12_10233 | 3H | 51.73 | 0.00 | 1.00 | 1 |
| 12_11511 | 3H | 51.73 | 0.00 | 1.00 | 1 |
| 12_30680 | 3H | 51.73 | 0.11 | 0.77 | 1 |
| 11_11258 | 3H | 52.5 | 0.18 | 0.66 | 1 |
| 11_20866 | 3H | 52.5 | 0.04 | 0.92 | 1 |
| 11_21129 | 3H | 52.5 | 0.52 | 0.30 | 1 |
| 12_30799 | 3H | 52.5 | 0.52 | 0.30 | 1 |
| 11_11086 | 3H | 53.27 | 0.08 | 0.83 | 1 |
| 12_30618 | 3H | 53.27 | 0.07 | 0.84 | 1 |
| 11_10137 | 3H | 54.4 | 0.88 | 0.13 | 1 |
| 11_10328 | 3H | 54.4 | 0.90 | 0.13 | 1 |
| 11_11099 | 3H | 54.4 | 0.23 | 0.58 | 1 |
| 11_11501 | 3H | 54.4 | 0.88 | 0.13 | 1 |
| 11_11502 | 3H | 54.4 | 0.01 | 0.97 | 1 |
| 11_20970 | 3H | 54.4 | 0.88 | 0.13 | 1 |
| 12_10391 | 3H | 54.4 | 0.00 | 1.00 | 1 |
| 12_11265 | 3H | 54.4 | 0.00 | 1.00 | 1 |
| 12_11295 | 3H | 54.4 | 0.00 | 1.00 | 1 |
| 12_11518 | 3H | 54.4 | 0.15 | 0.71 | 1 |
| 12_20574 | 3H | 54.4 | 0.33 | 0.47 | 1 |
| 12_20591 | 3H | 54.4 | 0.00 | 1.00 | 1 |
| 12_21411 | 3H | 54.4 | 0.00 | 1.00 | 1 |
| 12_21475 | 3H | 54.4 | 0.17 | 0.67 | 1 |
| 12_30039 | 3H | 54.4 | 1.09 | 0.08 | 1 |
| 12_30130 | 3H | 54.4 | 1.09 | 0.08 | 1 |
| 12_30262 | 3H | 54.4 | 0.00 | 1.00 | 1 |
| 12_30318 | 3H | 54.4 | 0.88 | 0.13 | 1 |
| 12_31008 | 3H | 54.4 | 0.01 | 0.97 | 1 |
| 12_31372 | 3H | 54.4 | 0.21 | 0.62 | 1 |
| 12_10155 | 3H | 55.57 | 0.05 | 0.90 | 1 |
| 12_10784 | 3H | 55.57 | 0.00 | 1.00 | 1 |
| 12_30809 | 3H | 55.57 | 0.33 | 0.47 | 1 |
| 12_31010 | 3H | 55.57 | 0.15 | 0.71 | 1 |
| 12_31012 | 3H | 55.57 | 0.99 | 0.10 | 1 |
| 12_31014 | 3H | 55.57 | 0.15 | 0.71 | 1 |
| 12_31502 | 3H | 55.57 | 0.90 | 0.13 | 1 |
| 11_10008 | 3H | 56.4 | 1.09 | 0.08 | 1 |
| 11_10011 | 3H | 56.4 | 1.60 | 0.03 | 1 |
| 11_10224 | 3H | 56.4 | 0.16 | 0.69 | 1 |
| 11_10456 | 3H | 56.4 | 0.88 | 0.13 | 1 |
| 11_10620 | 3H | 56.4 | 0.98 | 0.10 | 1 |
| 11_10925 | 3H | 56.4 | 0.98 | 0.10 | 1 |
| 11_10926 | 3H | 56.4 | 1.22 | 0.06 | 1 |
| 11_10966 | 3H | 56.4 | 0.95 | 0.11 | 1 |
| 11_11124 | 3H | 56.4 | 0.90 | 0.13 | 1 |
| 11_11125 | 3H | 56.4 | 0.99 | 0.10 | 1 |
| 11_11283 | 3H | 56.4 | 0.99 | 0.10 | 1 |
| 11_11337 | 3H | 56.4 | 0.90 | 0.13 | 1 |
| 11_11530 | 3H | 56.4 | 0.99 | 0.10 | 1 |
| 11_20002 | 3H | 56.4 | 1.00 | 0.10 | 1 |
| 11_20102 | 3H | 56.4 | 0.16 | 0.70 | 1 |
| 11_20288 | 3H | 56.4 | 1.01 | 0.10 | 1 |
| 11_20333 | 3H | 56.4 | 0.88 | 0.13 | 1 |
| 11_20428 | 3H | 56.4 | 1.09 | 0.08 | 1 |
| 11_20439 | 3H | 56.4 | 1.09 | 0.08 | 1 |
| 11_20486 | 3H | 56.4 | 0.96 | 0.11 | 1 |
| 11_20583 | 3H | 56.4 | 0.98 | 0.11 | 1 |
| 11_20796 | 3H | 56.4 | 1.09 | 0.08 | 1 |
| 11_20801 | 3H | 56.4 | 0.99 | 0.10 | 1 |
| 11_20856 | 3H | 56.4 | 0.87 | 0.14 | 1 |
| 11_20890 | 3H | 56.4 | 0.21 | 0.62 | 1 |
| 11_21062 | 3H | 56.4 | 1.09 | 0.08 | 1 |
| 11_21147 | 3H | 56.4 | 0.90 | 0.13 | 1 |
| 11_21435 | 3H | 56.4 | 0.98 | 0.11 | 1 |
| 11_21472 | 3H | 56.4 | 0.98 | 0.11 | 1 |
| 12_11482 | 3H | 56.4 | 0.00 | 1.00 | 1 |
| 12_20721 | 3H | 56.4 | 0.00 | 1.00 | 1 |
| 12_30126 | 3H | 56.4 | 0.15 | 0.71 | 1 |
| 12_31017 | 3H | 56.4 | 1.56 | 0.03 | 1 |
| 12_31214 | 3H | 56.4 | 1.01 | 0.10 | 1 |
| 12_31281 | 3H | 56.4 | 0.70 | 0.20 | 1 |
| 12_31368 | 3H | 56.4 | 1.21 | 0.06 | 1 |
| 11_20444 | 3H | 57.12 | 0.11 | 0.78 | 1 |
| 11_10225 | 3H | 58.01 | 0.15 | 0.70 | 1 |
| 11_11401 | 3H | 58.01 | 0.50 | 0.32 | 1 |
| 12_20108 | 3H | 58.01 | 0.08 | 0.84 | 1 |
| 11_11016 | 3H | 58.64 | 0.32 | 0.48 | 1 |
| 11_20276 | 3H | 58.64 | 0.32 | 0.48 | 1 |
| 11_20995 | 3H | 58.64 | 0.48 | 0.33 | 1 |
| 12_30009 | 3H | 58.64 | 0.46 | 0.34 | 1 |
| 12_31011 | 3H | 58.64 | 0.32 | 0.48 | 1 |
| 12_31016 | 3H | 58.64 | 0.10 | 0.79 | 1 |
| 12_31393 | 3H | 58.64 | 0.32 | 0.48 | 1 |
| 11_10158 | 3H | 59.89 | 0.38 | 0.42 | 1 |
| 11_10373 | 3H | 59.89 | 0.37 | 0.43 | 1 |
| 11_10653 | 3H | 59.89 | 0.02 | 0.95 | 1 |
| 11_21511 | 3H | 59.89 | 0.14 | 0.73 | 1 |
| 12_10678 | 3H | 59.89 | 0.00 | 1.00 | 1 |
| 12_10850 | 3H | 59.89 | 0.00 | 1.00 | 1 |
| 12_11429 | 3H | 59.89 | 0.00 | 1.00 | 1 |
| 12_21064 | 3H | 59.89 | 0.00 | 1.00 | 1 |
| 12_30829 | 3H | 59.89 | 0.37 | 0.43 | 1 |
| 11_10728 | 3H | 62.99 | 0.34 | 0.46 | 1 |
| 11_10281 | 3H | 64.19 | 0.05 | 0.90 | 1 |
| 11_11191 | 3H | 64.19 | 0.36 | 0.44 | 1 |
| 11_21120 | 3H | 64.19 | 0.05 | 0.90 | 1 |
| 12_11084 | 3H | 64.19 | 0.06 | 0.87 | 1 |
| 12_11150 | 3H | 64.19 | 0.06 | 0.87 | 1 |
| 12_20863 | 3H | 64.19 | 0.00 | 1.00 | 1 |
| 12_30088 | 3H | 64.19 | 0.05 | 0.88 | 1 |
| 11_10335 | 3H | 65.52 | 0.12 | 0.76 | 1 |
| 11_10839 | 3H | 65.52 | 1.98 | 0.01 | 1 |
| 11_11391 | 3H | 65.52 | 0.27 | 0.53 | 1 |
| 11_20704 | 3H | 65.52 | 0.26 | 0.55 | 1 |
| 11_21502 | 3H | 65.52 | 0.27 | 0.54 | 1 |
| 12_11152 | 3H | 65.52 | 0.00 | 1.00 | 1 |
| 12_11284 | 3H | 65.52 | 0.00 | 1.00 | 1 |
| 12_20608 | 3H | 65.52 | 0.00 | 1.00 | 1 |
| 12_30005 | 3H | 65.52 | 0.07 | 0.86 | 1 |
| 11_21305 | 3H | 67.57 | 0.74 | 0.18 | 1 |
| 11_20931 | 3H | 68.32 | 0.73 | 0.18 | 1 |
| 12_30616 | 3H | 68.32 | 0.19 | 0.64 | 1 |
| 12_30788 | 3H | 68.32 | 0.12 | 0.76 | 1 |
| 12_31153 | 3H | 68.32 | 0.13 | 0.74 | 1 |
| 11_10172 | 3H | 69.6 | 0.24 | 0.57 | 1 |
| 11_11394 | 3H | 69.6 | 0.08 | 0.83 | 1 |
| 11_20017 | 3H | 69.6 | 0.77 | 0.17 | 1 |
| 12_31242 | 3H | 69.6 | 0.53 | 0.29 | 1 |
| 11_11314 | 3H | 70.23 | 1.55 | 0.03 | 1 |
| 11_11241 | 3H | 70.71 | 2.15 | 0.01 | 1 |
| 11_20273 | 3H | 70.71 | 0.17 | 0.67 | 1 |
| 11_20877 | 3H | 70.71 | 0.17 | 0.68 | 1 |
| 12_31323 | 3H | 70.71 | 0.17 | 0.67 | 1 |
| 12_30754 | 3H | 71.49 | 0.10 | 0.80 | 1 |
| 11_20694 | 3H | 72.26 | 0.71 | 0.19 | 1 |
| 12_10452 | 3H | 72.26 | 0.33 | 0.46 | 1 |
| 12_31529 | 3H | 72.26 | 0.14 | 0.73 | 1 |
| 11_10350 | 3H | 73.53 | 2.87 | 0.00 | 0.813054 |
| 12_31356 | 3H | 73.53 | 2.98 | 0.00 | 0.813054 |
| 11_20521 | 3H | 74.15 | 1.93 | 0.01 | 1 |
| 11_10276 | 3H | 74.78 | 0.23 | 0.58 | 1 |
| 12_30399 | 3H | 74.78 | 1.05 | 0.09 | 1 |
| 11_20566 | 3H | 75.45 | 2.10 | 0.01 | 1 |
| 11_10683 | 3H | 76.2 | 0.15 | 0.71 | 1 |
| 11_20695 | 3H | 76.2 | 0.14 | 0.72 | 1 |
| 11_20778 | 3H | 76.2 | 1.23 | 0.06 | 1 |
| 12_10609 | 3H | 76.2 | 0.15 | 0.70 | 1 |
| 12_30743 | 3H | 76.2 | 1.23 | 0.06 | 1 |
| 12_31346 | 3H | 76.98 | 2.22 | 0.01 | 1 |
| 11_10047 | 3H | 78.53 | 0.23 | 0.59 | 1 |
| 11_20362 | 3H | 78.53 | 0.15 | 0.71 | 1 |
| 11_20597 | 3H | 78.53 | 0.14 | 0.72 | 1 |
| 12_11454 | 3H | 78.53 | 0.92 | 0.12 | 1 |
| 12_21194 | 3H | 78.53 | 0.00 | 1.00 | 1 |
| 11_20115 | 3H | 80.89 | 0.26 | 0.55 | 1 |
| 12_30170 | 3H | 80.89 | 0.33 | 0.47 | 1 |
| 11_20093 | 3H | 81.66 | 0.39 | 0.41 | 1 |
| 11_21358 | 3H | 81.66 | 0.17 | 0.67 | 1 |
| 12_20849 | 3H | 81.66 | 0.68 | 0.21 | 1 |
| 12_30677 | 3H | 81.66 | 0.18 | 0.66 | 1 |
| 12_31262 | 3H | 81.66 | 0.05 | 0.89 | 1 |
| 12_30278 | 3H | 83.23 | 0.29 | 0.51 | 1 |
| 12_11517 | 3H | 83.67 | 0.12 | 0.76 | 1 |
| 11_20063 | 3H | 85.99 | 0.49 | 0.32 | 1 |
| 12_11138 | 3H | 85.99 | 0.14 | 0.73 | 1 |
| 11_10444 | 3H | 87.24 | 0.07 | 0.85 | 1 |
| 11_10628 | 3H | 87.24 | 0.37 | 0.42 | 1 |
| 11_21348 | 3H | 87.24 | 0.08 | 0.82 | 1 |
| 12_31299 | 3H | 87.24 | 0.08 | 0.83 | 1 |
| 11_20136 | 3H | 88.82 | 0.39 | 0.41 | 1 |
| 11_21294 | 3H | 88.82 | 0.04 | 0.91 | 1 |
| 12_10134 | 3H | 89.31 | 0.09 | 0.81 | 1 |
| 12_30663 | 3H | 89.31 | 0.07 | 0.85 | 1 |
| 12_31018 | 3H | 89.31 | 0.74 | 0.18 | 1 |
| 12_30325 | 3H | 90.48 | 0.07 | 0.86 | 1 |
| 11_20659 | 3H | 91.25 | 0.09 | 0.82 | 1 |
| 11_10253 | 3H | 91.88 | 0.42 | 0.38 | 1 |
| 12_10583 | 3H | 91.88 | 0.34 | 0.45 | 1 |
| 11_10747 | 3H | 93.43 | 0.10 | 0.79 | 1 |
| 11_11021 | 3H | 93.43 | 0.41 | 0.39 | 1 |
| 12_31367 | 3H | 95.37 | 0.20 | 0.64 | 1 |
| 12_30250 | 3H | 96.15 | 0.03 | 0.93 | 1 |
| 12_30090 | 3H | 97.71 | 0.12 | 0.76 | 1 |
| 11_20130 | 3H | 98.49 | 0.08 | 0.83 | 1 |
| 11_20628 | 3H | 98.49 | 0.38 | 0.41 | 1 |
| 11_20999 | 3H | 98.49 | 0.05 | 0.89 | 1 |
| 11_21438 | 3H | 98.49 | 0.06 | 0.87 | 1 |
| 11_20626 | 3H | 99.12 | 0.11 | 0.78 | 1 |
| 11_10515 | 3H | 99.89 | 0.26 | 0.55 | 1 |
| 11_10184 | 3H | 100.66 | 0.06 | 0.87 | 1 |
| 11_21083 | 3H | 101.43 | 0.05 | 0.90 | 1 |
| 11_21381 | 3H | 102.21 | 0.35 | 0.44 | 1 |
| 11_21517 | 3H | 102.98 | 0.16 | 0.70 | 1 |
| 11_21495 | 3H | 104.53 | 0.06 | 0.86 | 1 |
| 12_10344 | 3H | 104.53 | 0.22 | 0.61 | 1 |
| 12_10662 | 3H | 104.53 | 0.12 | 0.76 | 1 |
| 12_30342 | 3H | 104.53 | 0.22 | 0.61 | 1 |
| 11_21493 | 3H | 105.3 | 0.09 | 0.81 | 1 |
| 12_30119 | 3H | 106.84 | 0.07 | 0.84 | 1 |
| 11_20009 | 3H | 107.63 | 0.31 | 0.49 | 1 |
| 11_21513 | 3H | 109.14 | 0.07 | 0.86 | 1 |
| 11_20023 | 3H | 111.42 | 0.10 | 0.80 | 1 |
| 11_21161 | 3H | 111.42 | 0.05 | 0.88 | 1 |
| 11_21212 | 3H | 111.42 | 0.18 | 0.67 | 1 |
| 12_11392 | 3H | 111.42 | 0.00 | 1.00 | 1 |
| 12_30423 | 3H | 111.42 | 0.03 | 0.93 | 1 |
| 11_10312 | 3H | 114 | 0.03 | 0.93 | 1 |
| 11_10753 | 3H | 114 | 0.01 | 0.97 | 1 |
| 11_11503 | 3H | 114 | 0.23 | 0.59 | 1 |
| 11_20168 | 3H | 114 | 0.26 | 0.55 | 1 |
| 11_20523 | 3H | 114 | 0.05 | 0.90 | 1 |
| 11_21277 | 3H | 114 | 0.15 | 0.71 | 1 |
| 12_10100 | 3H | 114 | 0.26 | 0.55 | 1 |
| 12_30276 | 3H | 114 | 0.24 | 0.58 | 1 |
| 12_30375 | 3H | 114.78 | 0.29 | 0.51 | 1 |
| 12_31329 | 3H | 115.55 | 0.23 | 0.59 | 1 |
| 11_10584 | 3H | 117.1 | 0.27 | 0.53 | 1 |
| 12_30927 | 3H | 117.1 | 0.01 | 0.99 | 1 |
| 12_10680 | 3H | 117.87 | 0.00 | 1.00 | 1 |
| 11_11330 | 3H | 120.59 | 0.15 | 0.70 | 1 |
| 12_30223 | 3H | 120.59 | 0.50 | 0.32 | 1 |
| 12_31220 | 3H | 120.59 | 0.46 | 0.35 | 1 |
| 12_31269 | 3H | 121.36 | 0.68 | 0.21 | 1 |
| 11_20944 | 3H | 122.14 | 1.08 | 0.08 | 1 |
| 11_10918 | 3H | 123.68 | 0.06 | 0.87 | 1 |
| 11_21405 | 3H | 123.68 | 0.06 | 0.87 | 1 |
| 12_30732 | 3H | 123.68 | 0.80 | 0.16 | 1 |
| 12_31499 | 3H | 123.68 | 0.81 | 0.15 | 1 |
| 12_30274 | 3H | 124.84 | 0.17 | 0.67 | 1 |
| 11_10754 | 3H | 126.27 | 0.07 | 0.85 | 1 |
| 11_10821 | 3H | 126.27 | 0.15 | 0.70 | 1 |
| 11_11172 | 3H | 126.27 | 0.09 | 0.81 | 1 |
| 12_11338 | 3H | 126.27 | 0.00 | 1.00 | 1 |
| 12_30084 | 3H | 126.27 | 0.74 | 0.18 | 1 |
| 12_30924 | 3H | 126.27 | 0.13 | 0.75 | 1 |
| 12_31525 | 3H | 126.27 | 0.25 | 0.56 | 1 |
| 11_10867 | 3H | 127.1 | 0.64 | 0.23 | 1 |
| 11_20650 | 3H | 127.1 | 0.19 | 0.64 | 1 |
| 12_20413 | 3H | 127.1 | 0.00 | 1.00 | 1 |
| 12_30096 | 3H | 127.1 | 0.55 | 0.28 | 1 |
| 12_31238 | 3H | 127.1 | 0.19 | 0.65 | 1 |
| 12_30081 | 3H | 128.64 | 0.07 | 0.85 | 1 |
| 11_10280 | 3H | 130.19 | 0.58 | 0.26 | 1 |
| 11_20343 | 3H | 130.19 | 0.54 | 0.29 | 1 |
| 11_11141 | 3H | 130.82 | 0.58 | 0.26 | 1 |
| 11_11196 | 3H | 130.82 | 0.39 | 0.41 | 1 |
| 11_20662 | 3H | 130.82 | 0.38 | 0.42 | 1 |
| 12_10188 | 3H | 130.82 | 0.46 | 0.35 | 1 |
| 12_10505 | 3H | 130.82 | 0.54 | 0.29 | 1 |
| 12_20369 | 3H | 130.82 | 0.16 | 0.70 | 1 |
| 11_10842 | 3H | 131.59 | 0.17 | 0.67 | 1 |
| 11_20612 | 3H | 131.59 | 0.46 | 0.35 | 1 |
| 12_30973 | 3H | 133.14 | 0.12 | 0.76 | 1 |
| 11_20527 | 3H | 134.31 | 0.41 | 0.39 | 1 |
| 12_30092 | 3H | 134.31 | 0.34 | 0.46 | 1 |
| 11_10381 | 3H | 136.66 | 0.27 | 0.54 | 1 |
| 11_11127 | 3H | 136.66 | 0.57 | 0.27 | 1 |
| 11_21428 | 3H | 136.66 | 0.29 | 0.51 | 1 |
| 12_10122 | 3H | 136.66 | 0.07 | 0.85 | 1 |
| 12_30860 | 3H | 136.66 | 0.10 | 0.80 | 1 |
| 11_20085 | 3H | 137.28 | 0.11 | 0.78 | 1 |
| 12_11154 | 3H | 138.83 | 0.39 | 0.41 | 1 |
| 12_31251 | 3H | 138.83 | 1.22 | 0.06 | 1 |
| 12_31496 | 3H | 140.37 | 0.42 | 0.38 | 1 |
| 11_20920 | 3H | 140.91 | 1.02 | 0.10 | 1 |
| 11_20851 | 3H | 141.54 | 0.55 | 0.28 | 1 |
| 11_21427 | 3H | 141.54 | 0.94 | 0.12 | 1 |
| 12_11047 | 3H | 141.54 | 0.01 | 0.97 | 1 |
| 12_30367 | 3H | 141.54 | 0.35 | 0.45 | 1 |
| 12_30137 | 3H | 142.32 | 0.79 | 0.16 | 1 |
| 11_10631 | 3H | 144.64 | 0.17 | 0.68 | 1 |
| 12_21386 | 3H | 145.89 | 0.03 | 0.93 | 1 |
| 12_11297 | 3H | 147.43 | 0.16 | 0.69 | 1 |
| 11_21266 | 3H | 148.89 | 0.05 | 0.88 | 1 |
| 12_31161 | 3H | 148.94 | 0.33 | 0.46 | 1 |
| 12_21531 | 3H | 149.71 | 0.13 | 0.75 | 1 |
| 11_21272 | 3H | 150.37 | 0.07 | 0.86 | 1 |
| 12_21376 | 3H | 151.22 | 0.06 | 0.88 | 1 |
| 12_30841 | 3H | 151.22 | 0.01 | 0.98 | 1 |
| 12_31500 | 3H | 151.99 | 0.17 | 0.67 | 1 |
| 12_20421 | 3H | 154.31 | 0.03 | 0.93 | 1 |
| 11_20155 | 3H | 155.09 | 0.06 | 0.87 | 1 |
| 12_20505 | 3H | 155.09 | 0.02 | 0.95 | 1 |
| 11_11436 | 3H | 155.85 | 0.33 | 0.46 | 1 |
| 12_21345 | 3H | 155.85 | 0.00 | 1.00 | 1 |
| 12_30370 | 3H | 155.85 | 0.00 | 1.00 | 1 |
| 12_30921 | 3H | 155.85 | 0.08 | 0.82 | 1 |
| 11_10935 | 3H | 160.08 | 0.00 | 1.00 | 1 |
| 12_11510 | 3H | 160.08 | 0.01 | 0.97 | 1 |
| 11_10646 | 3H | 162.15 | 0.21 | 0.61 | 1 |
| 11_10702 | 3H | 162.15 | 0.21 | 0.61 | 1 |
| 11_21008 | 3H | 162.15 | 1.09 | 0.08 | 1 |
| 12_20198 | 3H | 162.15 | 0.05 | 0.89 | 1 |
| 12_30271 | 3H | 162.15 | 0.55 | 0.28 | 1 |
| 12_30767 | 3H | 162.15 | 0.14 | 0.72 | 1 |
| 11_20605 | 3H | 166.22 | 0.29 | 0.52 | 1 |
| 11_10681 | 3H | 167.77 | 0.28 | 0.52 | 1 |
| 11_11410 | 3H | 167.77 | 0.05 | 0.89 | 1 |
| 12_10629 | 3H | 167.77 | 0.00 | 1.00 | 1 |
| 12_31388 | 3H | 167.77 | 0.12 | 0.76 | 1 |
| 11_10694 | 3H | 168.4 | 0.35 | 0.45 | 1 |
| 11_20057 | 3H | 168.4 | 0.07 | 0.85 | 1 |
| 11_21267 | 3H | 168.4 | 0.12 | 0.77 | 1 |
| 12_10014 | 3H | 168.4 | 0.40 | 0.40 | 1 |
| 12_30736 | 3H | 168.4 | 0.12 | 0.76 | 1 |
| 11_11516 | 3H | 169.32 | 0.27 | 0.54 | 1 |
| 12_20176 | 3H | 170.09 | 0.00 | 1.00 | 1 |
| 12_30229 | 3H | 170.09 | 0.00 | 1.00 | 1 |
| 12_21500 | 3H | 171.64 | 0.00 | 1.00 | 1 |
| 12_30055 | 3H | 172.41 | 0.01 | 0.98 | 1 |
| 11_10767 | 3H | 172.42 | 0.00 | 1.00 | 1 |
| 11_10283 | 3H | 173.17 | 0.33 | 0.46 | 1 |
| 11_10343 | 3H | 173.17 | 0.01 | 0.97 | 1 |
| 11_21362 | 3H | 173.17 | 0.12 | 0.75 | 1 |
| 12_20345 | 3H | 173.17 | 0.34 | 0.45 | 1 |
| 12_30135 | 3H | 173.17 | 0.14 | 0.72 | 1 |
| 11_10028 | 4H | 0 | 0.15 | 0.70 | 1 |
| 11_10247 | 4H | 0 | 0.02 | 0.96 | 1 |
| 11_10379 | 4H | 0 | 1.42 | 0.04 | 1 |
| 11_10509 | 4H | 0 | 0.31 | 0.50 | 1 |
| 11_10751 | 4H | 0 | 0.03 | 0.93 | 1 |
| 11_10809 | 4H | 0 | 0.25 | 0.56 | 1 |
| 11_10846 | 4H | 0 | 1.06 | 0.09 | 1 |
| 11_10914 | 4H | 0 | 0.95 | 0.11 | 1 |
| 11_20135 | 4H | 0 | 0.24 | 0.57 | 1 |
| 11_20450 | 4H | 0 | 0.24 | 0.57 | 1 |
| 11_20472 | 4H | 0 | 0.07 | 0.86 | 1 |
| 11_20668 | 4H | 0 | 0.02 | 0.95 | 1 |
| 11_20740 | 4H | 0 | 0.52 | 0.30 | 1 |
| 11_21254 | 4H | 0 | 0.01 | 0.99 | 1 |
| 12_21117 | 4H | 0 | 1.09 | 0.08 | 1 |
| 12_30764 | 4H | 0.74 | 0.20 | 0.64 | 1 |
| 12_31324 | 4H | 0.74 | 0.12 | 0.76 | 1 |
| 11_20145 | 4H | 1.64 | 0.08 | 0.83 | 1 |
| 11_10208 | 4H | 3.74 | 0.30 | 0.50 | 1 |
| 11_10409 | 4H | 3.74 | 0.22 | 0.60 | 1 |
| 11_21056 | 4H | 3.74 | 0.21 | 0.61 | 1 |
| 11_21228 | 4H | 3.74 | 0.01 | 0.97 | 1 |
| 11_11345 | 4H | 5.55 | 0.19 | 0.64 | 1 |
| 12_20274 | 4H | 7.06 | 0.00 | 1.00 | 1 |
| 12_30140 | 4H | 7.06 | 0.00 | 1.00 | 1 |
| 12_31486 | 4H | 7.06 | 0.05 | 0.89 | 1 |
| 11_10319 | 4H | 8.25 | 0.09 | 0.81 | 1 |
| 12_11485 | 4H | 11.27 | 0.21 | 0.61 | 1 |
| 11_10490 | 4H | 12.02 | 0.40 | 0.40 | 1 |
| 12_11300 | 4H | 12.02 | 0.00 | 1.00 | 1 |
| 12_31458 | 4H | 12.02 | 0.36 | 0.44 | 1 |
| 12_30540 | 4H | 15.75 | 0.06 | 0.88 | 1 |
| 12_30150 | 4H | 18.01 | 0.04 | 0.91 | 1 |
| 11_10113 | 4H | 19.52 | 0.13 | 0.75 | 1 |
| 11_10738 | 4H | 19.52 | 0.16 | 0.69 | 1 |
| 11_10223 | 4H | 20.12 | 0.25 | 0.56 | 1 |
| 11_10574 | 4H | 20.12 | 0.00 | 1.00 | 1 |
| 11_20557 | 4H | 20.12 | 0.27 | 0.54 | 1 |
| 11_21359 | 4H | 20.12 | 0.48 | 0.33 | 1 |
| 11_10221 | 4H | 21.61 | 0.44 | 0.36 | 1 |
| 11_11199 | 4H | 22.36 | 0.12 | 0.76 | 1 |
| 11_11136 | 4H | 23.1 | 0.08 | 0.83 | 1 |
| 11_21385 | 4H | 23.1 | 0.11 | 0.78 | 1 |
| 12_21458 | 4H | 23.85 | 0.00 | 1.00 | 1 |
| 11_10132 | 4H | 24.59 | 0.21 | 0.61 | 1 |
| 11_20210 | 4H | 24.59 | 0.22 | 0.60 | 1 |
| 11_20422 | 4H | 24.59 | 0.12 | 0.77 | 1 |
| 12_10395 | 4H | 24.59 | 0.12 | 0.76 | 1 |
| 12_10562 | 4H | 24.59 | 0.00 | 0.99 | 1 |
| 12_10626 | 4H | 24.59 | 0.00 | 1.00 | 1 |
| 12_11060 | 4H | 24.59 | 0.00 | 1.00 | 1 |
| 12_11175 | 4H | 24.59 | 0.00 | 1.00 | 1 |
| 12_30394 | 4H | 24.59 | 0.00 | 1.00 | 1 |
| 11_20109 | 4H | 26.19 | 1.54 | 0.03 | 1 |
| 11_20302 | 4H | 26.19 | 0.08 | 0.83 | 1 |
| 11_20680 | 4H | 26.19 | 1.42 | 0.04 | 1 |
| 11_21070 | 4H | 26.19 | 0.11 | 0.78 | 1 |
| 11_21418 | 4H | 26.19 | 1.41 | 0.04 | 1 |
| 11_20777 | 4H | 26.66 | 0.18 | 0.67 | 1 |
| 11_20001 | 4H | 28.15 | 0.14 | 0.73 | 1 |
| 11_10031 | 4H | 28.4 | 0.21 | 0.62 | 1 |
| 11_21374 | 4H | 28.4 | 0.00 | 0.99 | 1 |
| 12_30907 | 4H | 28.4 | 0.00 | 1.00 | 1 |
| 12_31313 | 4H | 31.43 | 0.34 | 0.46 | 1 |
| 12_31164 | 4H | 32.45 | 0.03 | 0.94 | 1 |
| 11_21122 | 4H | 33.38 | 0.08 | 0.84 | 1 |
| 11_21397 | 4H | 33.38 | 0.17 | 0.67 | 1 |
| 12_30863 | 4H | 33.38 | 0.07 | 0.86 | 1 |
| 12_30864 | 4H | 33.38 | 0.06 | 0.87 | 1 |
| 12_30865 | 4H | 33.38 | 0.07 | 0.86 | 1 |
| 11_20411 | 4H | 36.37 | 0.00 | 0.99 | 1 |
| 11_21389 | 4H | 36.37 | 0.07 | 0.84 | 1 |
| 12_10810 | 4H | 36.37 | 0.00 | 1.00 | 1 |
| 12_10860 | 4H | 36.37 | 0.03 | 0.93 | 1 |
| 12_31524 | 4H | 37.12 | 0.11 | 0.79 | 1 |
| 12_10171 | 4H | 38.63 | 0.83 | 0.15 | 1 |
| 12_10347 | 4H | 38.63 | 0.83 | 0.15 | 1 |
| 11_20012 | 4H | 39.76 | 0.05 | 0.89 | 1 |
| 11_20114 | 4H | 40.36 | 1.56 | 0.03 | 1 |
| 11_20180 | 4H | 40.36 | 1.74 | 0.02 | 1 |
| 12_10063 | 4H | 40.36 | 0.13 | 0.75 | 1 |
| 12_10371 | 4H | 40.36 | 1.76 | 0.02 | 1 |
| 12_20011 | 4H | 40.36 | 0.00 | 1.00 | 1 |
| 12_20240 | 4H | 40.36 | 0.01 | 0.97 | 1 |
| 11_11180 | 4H | 40.96 | 0.06 | 0.87 | 1 |
| 12_30328 | 4H | 40.96 | 0.07 | 0.85 | 1 |
| 11_10048 | 4H | 42.45 | 0.15 | 0.70 | 1 |
| 12_30187 | 4H | 42.45 | 0.00 | 1.00 | 1 |
| 12_30993 | 4H | 43.78 | 0.05 | 0.90 | 1 |
| 11_10668 | 4H | 44.94 | 0.42 | 0.38 | 1 |
| 11_10793 | 4H | 44.94 | 0.29 | 0.51 | 1 |
| 11_20939 | 4H | 46.41 | 0.33 | 0.46 | 1 |
| 11_21490 | 4H | 46.41 | 0.31 | 0.49 | 1 |
| 12_11112 | 4H | 46.41 | 0.00 | 1.00 | 1 |
| 11_11405 | 4H | 47.6 | 0.23 | 0.58 | 1 |
| 11_10093 | 4H | 48.5 | 0.18 | 0.66 | 1 |
| 11_10261 | 4H | 48.5 | 0.62 | 0.24 | 1 |
| 11_10432 | 4H | 48.5 | 0.00 | 1.00 | 1 |
| 11_10577 | 4H | 48.5 | 0.64 | 0.23 | 1 |
| 11_10667 | 4H | 48.5 | 0.22 | 0.60 | 1 |
| 11_10756 | 4H | 48.5 | 0.64 | 0.23 | 1 |
| 11_10942 | 4H | 48.5 | 0.06 | 0.88 | 1 |
| 11_20269 | 4H | 48.5 | 1.18 | 0.07 | 1 |
| 11_20782 | 4H | 48.5 | 0.18 | 0.67 | 1 |
| 11_20853 | 4H | 48.5 | 1.19 | 0.07 | 1 |
| 11_21071 | 4H | 48.5 | 0.15 | 0.71 | 1 |
| 11_21073 | 4H | 48.5 | 0.64 | 0.23 | 1 |
| 12_10195 | 4H | 48.5 | 0.00 | 1.00 | 1 |
| 12_21029 | 4H | 48.5 | 0.00 | 1.00 | 1 |
| 12_21442 | 4H | 48.5 | 0.00 | 0.99 | 1 |
| 12_30331 | 4H | 48.5 | 0.92 | 0.12 | 1 |
| 12_30488 | 4H | 48.5 | 0.01 | 0.99 | 1 |
| 12_30525 | 4H | 48.5 | 0.00 | 1.00 | 1 |
| 12_30878 | 4H | 48.5 | 0.00 | 0.99 | 1 |
| 12_31026 | 4H | 48.5 | 0.00 | 1.00 | 1 |
| 12_31360 | 4H | 48.5 | 0.27 | 0.54 | 1 |
| 12_31382 | 4H | 48.5 | 0.92 | 0.12 | 1 |
| 12_30777 | 4H | 49.5 | 0.65 | 0.22 | 1 |
| 11_20289 | 4H | 50.4 | 0.87 | 0.14 | 1 |
| 12_10607 | 4H | 50.4 | 0.00 | 1.00 | 1 |
| 12_11190 | 4H | 50.4 | 0.00 | 0.99 | 1 |
| 12_20525 | 4H | 50.4 | 0.00 | 1.00 | 1 |
| 12_20831 | 4H | 50.4 | 0.00 | 0.99 | 1 |
| 12_21137 | 4H | 50.4 | 0.00 | 1.00 | 1 |
| 12_30450 | 4H | 50.4 | 0.20 | 0.63 | 1 |
| 12_30605 | 4H | 50.4 | 1.13 | 0.07 | 1 |
| 12_30684 | 4H | 50.4 | 0.03 | 0.94 | 1 |
| 12_30866 | 4H | 50.4 | 0.22 | 0.60 | 1 |
| 12_31156 | 4H | 50.4 | 0.11 | 0.78 | 1 |
| 11_10411 | 4H | 51.3 | 0.20 | 0.63 | 1 |
| 11_10480 | 4H | 51.3 | 0.20 | 0.63 | 1 |
| 11_11042 | 4H | 51.3 | 0.34 | 0.45 | 1 |
| 11_20496 | 4H | 51.3 | 0.11 | 0.77 | 1 |
| 12_11063 | 4H | 51.3 | 0.70 | 0.20 | 1 |
| 11_10946 | 4H | 52.75 | 0.00 | 1.00 | 1 |
| 12_30427 | 4H | 53.5 | 0.32 | 0.48 | 1 |
| 11_10881 | 4H | 54.25 | 0.23 | 0.59 | 1 |
| 11_11114 | 4H | 54.25 | 1.31 | 0.05 | 1 |
| 11_20610 | 4H | 54.25 | 0.48 | 0.33 | 1 |
| 11_11244 | 4H | 54.98 | 0.18 | 0.66 | 1 |
| 11_10046 | 4H | 55.63 | 0.31 | 0.50 | 1 |
| 11_10262 | 4H | 55.63 | 0.21 | 0.61 | 1 |
| 11_10527 | 4H | 55.63 | 0.15 | 0.71 | 1 |
| 11_10568 | 4H | 55.63 | 0.31 | 0.50 | 1 |
| 11_20020 | 4H | 55.63 | 0.44 | 0.36 | 1 |
| 11_20363 | 4H | 55.63 | 0.22 | 0.60 | 1 |
| 11_20412 | 4H | 55.63 | 0.23 | 0.59 | 1 |
| 11_21481 | 4H | 55.63 | 0.19 | 0.64 | 1 |
| 12_10088 | 4H | 55.63 | 0.20 | 0.63 | 1 |
| 12_10426 | 4H | 55.63 | 0.64 | 0.23 | 1 |
| 12_11108 | 4H | 55.63 | 0.00 | 1.00 | 1 |
| 12_11232 | 4H | 55.63 | 0.00 | 1.00 | 1 |
| 12_20898 | 4H | 55.63 | 0.00 | 1.00 | 1 |
| 12_30060 | 4H | 55.63 | 0.21 | 0.62 | 1 |
| 12_30839 | 4H | 55.63 | 0.20 | 0.64 | 1 |
| 12_30995 | 4H | 55.63 | 0.39 | 0.40 | 1 |
| 12_31297 | 4H | 55.63 | 0.20 | 0.63 | 1 |
| 12_31462 | 4H | 55.63 | 0.37 | 0.43 | 1 |
| 11_20361 | 4H | 59.37 | 1.10 | 0.08 | 1 |
| 11_20482 | 4H | 59.37 | 0.76 | 0.17 | 1 |
| 11_21010 | 4H | 59.37 | 0.88 | 0.13 | 1 |
| 11_21191 | 4H | 61.04 | 0.19 | 0.64 | 1 |
| 11_21400 | 4H | 61.04 | 0.60 | 0.25 | 1 |
| 12_30054 | 4H | 61.04 | 0.68 | 0.21 | 1 |
| 12_30237 | 4H | 61.04 | 0.12 | 0.76 | 1 |
| 11_21087 | 4H | 62.1 | 0.21 | 0.62 | 1 |
| 11_11207 | 4H | 62.83 | 0.14 | 0.72 | 1 |
| 11_20453 | 4H | 62.83 | 0.18 | 0.66 | 1 |
| 11_21296 | 4H | 62.83 | 0.18 | 0.66 | 1 |
| 12_11427 | 4H | 62.83 | 0.00 | 1.00 | 1 |
| 12_31186 | 4H | 62.83 | 0.01 | 0.98 | 1 |
| 11_20820 | 4H | 63.56 | 0.01 | 0.98 | 1 |
| 11_20723 | 4H | 64.31 | 0.01 | 0.98 | 1 |
| 11_10052 | 4H | 65.05 | 1.15 | 0.07 | 1 |
| 11_10639 | 4H | 65.05 | 1.17 | 0.07 | 1 |
| 11_11224 | 4H | 65.05 | 0.07 | 0.86 | 1 |
| 11_11229 | 4H | 65.05 | 0.95 | 0.11 | 1 |
| 11_11431 | 4H | 65.05 | 0.78 | 0.17 | 1 |
| 11_20062 | 4H | 65.05 | 0.01 | 0.98 | 1 |
| 11_20906 | 4H | 65.05 | 0.88 | 0.13 | 1 |
| 11_20924 | 4H | 65.05 | 1.16 | 0.07 | 1 |
| 12_10053 | 4H | 65.05 | 0.00 | 1.00 | 1 |
| 12_30620 | 4H | 65.05 | 0.10 | 0.79 | 1 |
| 12_31515 | 4H | 65.05 | 0.01 | 0.97 | 1 |
| 12_30455 | 4H | 65.8 | 0.10 | 0.79 | 1 |
| 11_10010 | 4H | 66 | 0.84 | 0.14 | 1 |
| 12_30755 | 4H | 66 | 0.73 | 0.19 | 1 |
| 12_30904 | 4H | 66 | 0.73 | 0.19 | 1 |
| 12_30905 | 4H | 66 | 0.84 | 0.15 | 1 |
| 12_30906 | 4H | 66 | 0.85 | 0.14 | 1 |
| 12_31385 | 4H | 66 | 0.86 | 0.14 | 1 |
| 12_31493 | 4H | 66 | 0.08 | 0.83 | 1 |
| 11_10606 | 4H | 67.46 | 0.71 | 0.20 | 1 |
| 11_20072 | 4H | 67.46 | 0.52 | 0.30 | 1 |
| 11_10627 | 4H | 68.21 | 0.15 | 0.70 | 1 |
| 11_20451 | 4H | 68.21 | 0.22 | 0.60 | 1 |
| 11_20580 | 4H | 68.21 | 0.70 | 0.20 | 1 |
| 12_30693 | 4H | 68.21 | 0.22 | 0.60 | 1 |
| 12_31536 | 4H | 68.21 | 0.18 | 0.67 | 1 |
| 11_11513 | 4H | 69.51 | 0.48 | 0.33 | 1 |
| 11_21504 | 4H | 69.51 | 1.14 | 0.07 | 1 |
| 11_10467 | 4H | 72.08 | 0.27 | 0.54 | 1 |
| 12_31258 | 4H | 73.57 | 0.00 | 1.00 | 1 |
| 12_31362 | 4H | 73.57 | 0.16 | 0.69 | 1 |
| 11_10829 | 4H | 73.84 | 0.12 | 0.76 | 1 |
| 11_10090 | 4H | 76.03 | 0.31 | 0.49 | 1 |
| 11_10309 | 4H | 76.03 | 0.08 | 0.83 | 1 |
| 11_20815 | 4H | 76.03 | 0.65 | 0.22 | 1 |
| 12_20143 | 4H | 76.03 | 0.19 | 0.64 | 1 |
| 11_11004 | 4H | 77.31 | 0.22 | 0.61 | 1 |
| 11_21332 | 4H | 77.31 | 0.02 | 0.96 | 1 |
| 11_21353 | 4H | 77.31 | 0.21 | 0.61 | 1 |
| 12_30136 | 4H | 77.31 | 0.21 | 0.61 | 1 |
| 12_31231 | 4H | 77.31 | 0.22 | 0.61 | 1 |
| 11_10523 | 4H | 78.77 | 0.07 | 0.86 | 1 |
| 12_31148 | 4H | 78.77 | 0.06 | 0.88 | 1 |
| 11_11500 | 4H | 79.58 | 0.05 | 0.89 | 1 |
| 12_10170 | 4H | 79.58 | 0.00 | 1.00 | 1 |
| 12_30226 | 4H | 79.58 | 0.00 | 1.00 | 1 |
| 11_20670 | 4H | 80.79 | 0.41 | 0.39 | 1 |
| 12_11526 | 4H | 80.95 | 0.00 | 1.00 | 1 |
| 11_20197 | 4H | 81.69 | 0.49 | 0.32 | 1 |
| 11_10724 | 4H | 82.42 | 0.35 | 0.45 | 1 |
| 12_31246 | 4H | 83.55 | 0.04 | 0.91 | 1 |
| 11_10723 | 4H | 84.3 | 0.43 | 0.37 | 1 |
| 12_30390 | 4H | 84.3 | 0.03 | 0.94 | 1 |
| 12_10670 | 4H | 85.04 | 0.04 | 0.92 | 1 |
| 11_11213 | 4H | 86.27 | 0.13 | 0.74 | 1 |
| 11_20178 | 4H | 86.27 | 0.14 | 0.73 | 1 |
| 11_11398 | 4H | 87.49 | 0.61 | 0.25 | 1 |
| 11_20765 | 4H | 87.49 | 0.11 | 0.79 | 1 |
| 11_20358 | 4H | 88.22 | 0.16 | 0.70 | 1 |
| 11_10588 | 4H | 89.39 | 0.30 | 0.50 | 1 |
| 12_30138 | 4H | 90.29 | 0.00 | 1.00 | 1 |
| 11_20384 | 4H | 91.78 | 0.02 | 0.95 | 1 |
| 11_20732 | 4H | 92.38 | 0.27 | 0.54 | 1 |
| 12_30142 | 4H | 93.13 | 0.00 | 1.00 | 1 |
| 12_30232 | 4H | 93.13 | 0.00 | 1.00 | 1 |
| 11_10785 | 4H | 93.87 | 0.02 | 0.96 | 1 |
| 11_11292 | 4H | 96.59 | 0.02 | 0.96 | 1 |
| 11_20838 | 4H | 96.59 | 0.19 | 0.64 | 1 |
| 12_10271 | 4H | 96.59 | 0.00 | 0.99 | 1 |
| 12_10824 | 4H | 96.59 | 0.20 | 0.63 | 1 |
| 12_30046 | 4H | 96.59 | 0.00 | 1.00 | 1 |
| 12_30117 | 4H | 96.59 | 0.10 | 0.79 | 1 |
| 12_30554 | 4H | 96.59 | 0.24 | 0.58 | 1 |
| 12_30584 | 4H | 96.59 | 1.16 | 0.07 | 1 |
| 11_21243 | 4H | 97.06 | 0.19 | 0.64 | 1 |
| 11_20762 | 4H | 98.55 | 0.56 | 0.28 | 1 |
| 12_10666 | 4H | 98.55 | 0.57 | 0.27 | 1 |
| 11_20119 | 4H | 99.28 | 0.52 | 0.30 | 1 |
| 11_10614 | 4H | 100.74 | 0.32 | 0.47 | 1 |
| 11_11470 | 4H | 100.74 | 0.26 | 0.55 | 1 |
| 12_11183 | 4H | 100.74 | 0.07 | 0.86 | 1 |
| 12_30158 | 4H | 100.74 | 0.06 | 0.87 | 1 |
| 12_30987 | 4H | 100.74 | 0.66 | 0.22 | 1 |
| 12_30988 | 4H | 100.74 | 2.50 | 0.00 | 1 |
| 11_20454 | 4H | 101.62 | 0.55 | 0.28 | 1 |
| 11_20515 | 4H | 101.62 | 0.44 | 0.36 | 1 |
| 11_10510 | 4H | 102.37 | 0.08 | 0.83 | 1 |
| 11_10334 | 4H | 103.11 | 0.04 | 0.92 | 1 |
| 11_21111 | 4H | 103.11 | 0.00 | 1.00 | 1 |
| 12_11139 | 4H | 103.11 | 0.00 | 1.00 | 1 |
| 12_30990 | 4H | 103.11 | 0.99 | 0.10 | 1 |
| 12_31139 | 4H | 103.11 | 0.93 | 0.12 | 1 |
| 11_20974 | 4H | 106.03 | 0.07 | 0.85 | 1 |
| 12_11194 | 4H | 107.12 | 0.55 | 0.28 | 1 |
| 12_30385 | 4H | 107.86 | 0.45 | 0.35 | 1 |
| 11_10123 | 4H | 108.7 | 0.18 | 0.66 | 1 |
| 12_31138 | 4H | 111.07 | 0.43 | 0.37 | 1 |
| 11_10712 | 4H | 111.66 | 0.07 | 0.86 | 1 |
| 12_11233 | 4H | 111.66 | 0.00 | 1.00 | 1 |
| 12_20237 | 4H | 111.66 | 0.56 | 0.28 | 1 |
| 11_11299 | 4H | 111.68 | 0.25 | 0.56 | 1 |
| 11_20701 | 4H | 113.17 | 0.19 | 0.65 | 1 |
| 12_11235 | 4H | 113.17 | 0.00 | 1.00 | 1 |
| 11_11066 | 4H | 113.92 | 0.11 | 0.78 | 1 |
| 11_21035 | 4H | 113.92 | 0.19 | 0.64 | 1 |
| 11_10611 | 4H | 114.66 | 0.12 | 0.76 | 1 |
| 11_10697 | 4H | 114.66 | 0.02 | 0.96 | 1 |
| 12_30146 | 4H | 114.66 | 0.00 | 1.00 | 1 |
| 11_21130 | 4H | 116.85 | 0.05 | 0.90 | 1 |
| 11_10269 | 4H | 117.6 | 0.09 | 0.81 | 1 |
| 11_20224 | 4H | 117.6 | 0.12 | 0.75 | 1 |
| 11_21210 | 4H | 117.6 | 0.27 | 0.53 | 1 |
| 12_20760 | 4H | 118.34 | 0.00 | 1.00 | 1 |
| 11_10610 | 4H | 119.09 | 0.06 | 0.87 | 1 |
| 11_20007 | 4H | 119.09 | 0.05 | 0.90 | 1 |
| 11_20272 | 4H | 119.09 | 0.04 | 0.91 | 1 |
| 12_21450 | 4H | 119.09 | 0.00 | 1.00 | 1 |
| 12_30425 | 4H | 119.09 | 0.06 | 0.86 | 1 |
| 12_30476 | 4H | 119.09 | 0.06 | 0.87 | 1 |
| 12_30873 | 4H | 119.09 | 0.05 | 0.89 | 1 |
| 11_10387 | 4H | 119.84 | 0.27 | 0.54 | 1 |
| 12_30006 | 4H | 119.84 | 0.95 | 0.11 | 1 |
| 12_30239 | 4H | 119.84 | 1.07 | 0.09 | 1 |
| 12_31422 | 4H | 120.58 | 0.13 | 0.73 | 1 |
| 11_11186 | 4H | 121.83 | 0.10 | 0.80 | 1 |
| 11_11019 | 4H | 123.29 | 0.03 | 0.93 | 1 |
| 11_20013 | 4H | 123.29 | 0.21 | 0.62 | 1 |
| 11_20089 | 4H | 123.29 | 0.12 | 0.76 | 1 |
| 12_30824 | 4H | 123.29 | 0.03 | 0.93 | 1 |
| 12_30825 | 4H | 123.29 | 0.03 | 0.93 | 1 |
| 11_10251 | 5H | 0 | 0.05 | 0.89 | 1 |
| 11_10405 | 5H | 0 | 0.37 | 0.43 | 1 |
| 11_10593 | 5H | 0 | 0.03 | 0.93 | 1 |
| 11_10870 | 5H | 0 | 0.18 | 0.66 | 1 |
| 11_11361 | 5H | 0 | 0.16 | 0.69 | 1 |
| 11_11448 | 5H | 0 | 0.45 | 0.36 | 1 |
| 11_20386 | 5H | 0 | 0.13 | 0.74 | 1 |
| 11_20644 | 5H | 0 | 0.08 | 0.84 | 1 |
| 11_21207 | 5H | 0 | 0.00 | 1.00 | 1 |
| 11_21244 | 5H | 0 | 0.13 | 0.75 | 1 |
| 11_21514 | 5H | 0 | 0.53 | 0.29 | 1 |
| 12_30163 | 5H | 0 | 0.00 | 1.00 | 1 |
| 11_20226 | 5H | 2.09 | 0.09 | 0.82 | 1 |
| 11_20894 | 5H | 2.09 | 0.09 | 0.81 | 1 |
| 12_30976 | 5H | 2.09 | 0.05 | 0.90 | 1 |
| 11_20553 | 5H | 2.81 | 0.10 | 0.79 | 1 |
| 12_30543 | 5H | 2.81 | 0.02 | 0.95 | 1 |
| 12_30975 | 5H | 4.96 | 0.09 | 0.81 | 1 |
| 12_31023 | 5H | 4.96 | 0.15 | 0.70 | 1 |
| 12_30001 | 5H | 5.68 | 0.07 | 0.85 | 1 |
| 12_30591 | 5H | 5.68 | 0.01 | 0.98 | 1 |
| 12_30979 | 5H | 5.68 | 0.08 | 0.83 | 1 |
| 11_20206 | 5H | 6.4 | 0.49 | 0.33 | 1 |
| 12_30977 | 5H | 6.4 | 0.14 | 0.73 | 1 |
| 12_31022 | 5H | 6.4 | 0.10 | 0.79 | 1 |
| 11_21202 | 5H | 7.03 | 0.04 | 0.91 | 1 |
| 11_11381 | 5H | 7.48 | 0.28 | 0.53 | 1 |
| 11_21221 | 5H | 9.33 | 0.53 | 0.30 | 1 |
| 11_20533 | 5H | 17.38 | 0.21 | 0.61 | 1 |
| 12_31094 | 5H | 18.09 | 0.21 | 0.61 | 1 |
| 11_20010 | 5H | 18.72 | 0.08 | 0.83 | 1 |
| 12_30714 | 5H | 19.44 | 0.13 | 0.75 | 1 |
| 11_10695 | 5H | 25.23 | 1.22 | 0.06 | 1 |
| 11_20873 | 5H | 26.28 | 0.54 | 0.29 | 1 |
| 11_21065 | 5H | 26.28 | 0.22 | 0.60 | 1 |
| 12_30167 | 5H | 26.28 | 0.24 | 0.57 | 1 |
| 11_10974 | 5H | 27 | 0.53 | 0.29 | 1 |
| 11_21426 | 5H | 27 | 0.01 | 0.99 | 1 |
| 12_30531 | 5H | 27.72 | 0.01 | 0.99 | 1 |
| 11_11048 | 5H | 29.9 | 0.51 | 0.31 | 1 |
| 12_20576 | 5H | 29.97 | 0.00 | 1.00 | 1 |
| 11_21324 | 5H | 30.99 | 0.30 | 0.50 | 1 |
| 12_10530 | 5H | 33.09 | 1.04 | 0.09 | 1 |
| 11_10688 | 5H | 34.25 | 1.56 | 0.03 | 1 |
| 12_10499 | 5H | 34.25 | 0.95 | 0.11 | 1 |
| 11_10580 | 5H | 35.69 | 1.11 | 0.08 | 1 |
| 11_10621 | 5H | 37.11 | 1.10 | 0.08 | 1 |
| 12_30410 | 5H | 37.11 | 0.71 | 0.19 | 1 |
| 11_20845 | 5H | 39.97 | 0.46 | 0.35 | 1 |
| 11_20980 | 5H | 39.97 | 0.43 | 0.37 | 1 |
| 11_21391 | 5H | 39.97 | 0.43 | 0.37 | 1 |
| 12_10864 | 5H | 41.64 | 0.01 | 0.97 | 1 |
| 12_20770 | 5H | 42.32 | 0.17 | 0.67 | 1 |
| 11_21253 | 5H | 43.14 | 0.78 | 0.17 | 1 |
| 12_30707 | 5H | 43.14 | 0.74 | 0.18 | 1 |
| 11_20729 | 5H | 44.12 | 0.40 | 0.40 | 1 |
| 11_10260 | 5H | 46.23 | 0.06 | 0.86 | 1 |
| 11_10955 | 5H | 46.23 | 0.44 | 0.36 | 1 |
| 11_20571 | 5H | 46.23 | 0.47 | 0.34 | 1 |
| 11_20766 | 5H | 46.23 | 0.10 | 0.79 | 1 |
| 11_20903 | 5H | 46.23 | 0.62 | 0.24 | 1 |
| 11_20987 | 5H | 46.23 | 1.11 | 0.08 | 1 |
| 12_30654 | 5H | 46.23 | 0.97 | 0.11 | 1 |
| 12_31312 | 5H | 46.23 | 0.60 | 0.25 | 1 |
| 11_11432 | 5H | 47.39 | 0.61 | 0.24 | 1 |
| 12_30105 | 5H | 47.39 | 0.03 | 0.93 | 1 |
| 12_31492 | 5H | 47.39 | 0.12 | 0.77 | 1 |
| 12_21372 | 5H | 48.11 | 0.00 | 1.00 | 1 |
| 12_31257 | 5H | 48.11 | 0.29 | 0.51 | 1 |
| 11_11198 | 5H | 48.83 | 0.15 | 0.70 | 1 |
| 11_21401 | 5H | 48.83 | 0.15 | 0.71 | 1 |
| 12_10923 | 5H | 48.83 | 0.15 | 0.70 | 1 |
| 12_31155 | 5H | 48.83 | 0.17 | 0.68 | 1 |
| 11_10116 | 5H | 50.27 | 0.15 | 0.71 | 1 |
| 11_20841 | 5H | 50.27 | 0.23 | 0.58 | 1 |
| 11_21308 | 5H | 50.27 | 0.15 | 0.70 | 1 |
| 11_21447 | 5H | 50.27 | 0.23 | 0.58 | 1 |
| 12_30354 | 5H | 50.27 | 0.16 | 0.70 | 1 |
| 12_30454 | 5H | 50.27 | 0.14 | 0.72 | 1 |
| 12_30729 | 5H | 50.27 | 0.23 | 0.59 | 1 |
| 12_31390 | 5H | 50.27 | 0.14 | 0.72 | 1 |
| 12_31512 | 5H | 50.27 | 0.14 | 0.72 | 1 |
| 11_20697 | 5H | 50.72 | 0.27 | 0.54 | 1 |
| 11_10157 | 5H | 51 | 0.11 | 0.77 | 1 |
| 11_10177 | 5H | 51 | 0.12 | 0.77 | 1 |
| 11_10240 | 5H | 51 | 0.12 | 0.77 | 1 |
| 11_10252 | 5H | 51 | 0.12 | 0.77 | 1 |
| 11_10318 | 5H | 51 | 0.12 | 0.77 | 1 |
| 11_10856 | 5H | 51 | 0.12 | 0.77 | 1 |
| 11_10995 | 5H | 51 | 0.21 | 0.62 | 1 |
| 11_20129 | 5H | 51 | 0.08 | 0.84 | 1 |
| 11_20700 | 5H | 51 | 0.21 | 0.62 | 1 |
| 11_20708 | 5H | 51 | 0.13 | 0.75 | 1 |
| 11_20958 | 5H | 51 | 0.09 | 0.82 | 1 |
| 11_21011 | 5H | 51 | 0.21 | 0.62 | 1 |
| 11_21040 | 5H | 51 | 0.12 | 0.77 | 1 |
| 11_21350 | 5H | 51 | 0.21 | 0.62 | 1 |
| 12_11399 | 5H | 51 | 0.00 | 1.00 | 1 |
| 12_11462 | 5H | 51 | 0.01 | 0.97 | 1 |
| 12_30337 | 5H | 51 | 0.12 | 0.77 | 1 |
| 12_30768 | 5H | 51 | 0.12 | 0.77 | 1 |
| 12_30792 | 5H | 51 | 0.12 | 0.77 | 1 |
| 12_31317 | 5H | 51 | 0.49 | 0.32 | 1 |
| 11_10058 | 5H | 51.3 | 0.50 | 0.32 | 1 |
| 11_10481 | 5H | 51.3 | 0.12 | 0.77 | 1 |
| 11_10913 | 5H | 51.3 | 0.12 | 0.77 | 1 |
| 12_30575 | 5H | 51.3 | 0.37 | 0.43 | 1 |
| 12_30709 | 5H | 51.3 | 0.16 | 0.69 | 1 |
| 12_30728 | 5H | 51.3 | 0.06 | 0.87 | 1 |
| 12_31520 | 5H | 51.3 | 0.01 | 0.97 | 1 |
| 11_10661 | 5H | 51.6 | 0.39 | 0.41 | 1 |
| 11_11128 | 5H | 51.6 | 0.07 | 0.86 | 1 |
| 11_11469 | 5H | 51.6 | 0.11 | 0.77 | 1 |
| 11_11506 | 5H | 51.6 | 0.60 | 0.25 | 1 |
| 11_20179 | 5H | 51.6 | 0.12 | 0.77 | 1 |
| 11_20332 | 5H | 51.6 | 0.28 | 0.52 | 1 |
| 11_20461 | 5H | 51.6 | 0.50 | 0.32 | 1 |
| 11_20524 | 5H | 51.6 | 0.12 | 0.77 | 1 |
| 11_20737 | 5H | 51.6 | 0.33 | 0.46 | 1 |
| 11_21215 | 5H | 51.6 | 0.50 | 0.32 | 1 |
| 11_21260 | 5H | 51.6 | 0.27 | 0.53 | 1 |
| 12_30109 | 5H | 51.6 | 0.00 | 1.00 | 1 |
| 12_30408 | 5H | 51.6 | 0.48 | 0.33 | 1 |
| 12_30717 | 5H | 51.6 | 0.28 | 0.53 | 1 |
| 12_31259 | 5H | 51.6 | 0.49 | 0.32 | 1 |
| 11_11260 | 5H | 52.02 | 0.51 | 0.31 | 1 |
| 11_21318 | 5H | 53.18 | 0.18 | 0.66 | 1 |
| 12_30214 | 5H | 53.9 | 0.52 | 0.30 | 1 |
| 12_20059 | 5H | 55.34 | 0.00 | 1.00 | 1 |
| 11_21536 | 5H | 56.77 | 0.36 | 0.44 | 1 |
| 12_10508 | 5H | 56.77 | 0.17 | 0.67 | 1 |
| 12_31117 | 5H | 56.77 | 0.32 | 0.48 | 1 |
| 11_20239 | 5H | 57.36 | 0.10 | 0.79 | 1 |
| 11_20105 | 5H | 57.98 | 0.72 | 0.19 | 1 |
| 11_21148 | 5H | 57.98 | 0.75 | 0.18 | 1 |
| 12_10079 | 5H | 57.98 | 0.37 | 0.43 | 1 |
| 12_10264 | 5H | 57.98 | 0.02 | 0.95 | 1 |
| 11_20306 | 5H | 58.7 | 0.00 | 0.99 | 1 |
| 11_10671 | 5H | 59.4 | 0.76 | 0.17 | 1 |
| 11_10840 | 5H | 59.4 | 0.77 | 0.17 | 1 |
| 11_11240 | 5H | 59.4 | 0.31 | 0.49 | 1 |
| 11_20283 | 5H | 59.4 | 0.00 | 0.99 | 1 |
| 11_20372 | 5H | 59.4 | 0.76 | 0.17 | 1 |
| 11_20501 | 5H | 59.4 | 0.21 | 0.62 | 1 |
| 11_20961 | 5H | 59.4 | 0.27 | 0.54 | 1 |
| 12_10034 | 5H | 59.4 | 0.27 | 0.54 | 1 |
| 12_10899 | 5H | 59.4 | 0.00 | 0.99 | 1 |
| 12_11151 | 5H | 59.4 | 0.00 | 1.00 | 1 |
| 12_11363 | 5H | 59.4 | 0.00 | 1.00 | 1 |
| 12_11512 | 5H | 59.4 | 0.00 | 1.00 | 1 |
| 12_20981 | 5H | 59.4 | 0.00 | 1.00 | 1 |
| 12_30111 | 5H | 59.4 | 0.13 | 0.74 | 1 |
| 12_30538 | 5H | 59.4 | 1.19 | 0.06 | 1 |
| 12_30644 | 5H | 59.4 | 0.37 | 0.42 | 1 |
| 12_30700 | 5H | 59.4 | 0.60 | 0.25 | 1 |
| 12_30747 | 5H | 59.4 | 0.64 | 0.23 | 1 |
| 12_31062 | 5H | 59.4 | 0.00 | 1.00 | 1 |
| 12_31064 | 5H | 59.4 | 0.00 | 1.00 | 1 |
| 12_31183 | 5H | 59.4 | 1.40 | 0.04 | 1 |
| 12_31340 | 5H | 59.4 | 0.21 | 0.62 | 1 |
| 12_31451 | 5H | 59.4 | 0.00 | 1.00 | 1 |
| 12_31503 | 5H | 59.4 | 0.78 | 0.17 | 1 |
| 11_11159 | 5H | 60.74 | 0.06 | 0.88 | 1 |
| 11_11221 | 5H | 60.74 | 0.15 | 0.71 | 1 |
| 11_21200 | 5H | 60.74 | 0.15 | 0.71 | 1 |
| 11_21508 | 5H | 60.74 | 0.35 | 0.45 | 1 |
| 12_10725 | 5H | 60.74 | 0.01 | 0.98 | 1 |
| 12_11089 | 5H | 60.74 | 0.00 | 1.00 | 1 |
| 12_11385 | 5H | 60.74 | 0.04 | 0.91 | 1 |
| 12_30011 | 5H | 60.74 | 0.00 | 1.00 | 1 |
| 12_30515 | 5H | 60.74 | 0.13 | 0.74 | 1 |
| 12_31032 | 5H | 60.74 | 0.00 | 1.00 | 1 |
| 12_31033 | 5H | 60.74 | 0.06 | 0.88 | 1 |
| 12_31034 | 5H | 60.74 | 0.00 | 1.00 | 1 |
| 12_31035 | 5H | 60.74 | 0.00 | 1.00 | 1 |
| 12_31280 | 5H | 60.74 | 0.04 | 0.91 | 1 |
| 11_20265 | 5H | 62.15 | 0.01 | 0.99 | 1 |
| 11_11281 | 5H | 63.31 | 0.03 | 0.93 | 1 |
| 11_20441 | 5H | 63.31 | 0.44 | 0.36 | 1 |
| 11_21344 | 5H | 63.31 | 0.27 | 0.53 | 1 |
| 12_30745 | 5H | 64.04 | 0.03 | 0.94 | 1 |
| 11_20713 | 5H | 65.49 | 0.04 | 0.91 | 1 |
| 12_31477 | 5H | 65.49 | 0.03 | 0.94 | 1 |
| 12_21036 | 5H | 66.32 | 0.00 | 1.00 | 1 |
| 11_21275 | 5H | 67.54 | 0.03 | 0.94 | 1 |
| 11_21121 | 5H | 68.35 | 0.26 | 0.55 | 1 |
| 11_21239 | 5H | 69.34 | 0.64 | 0.23 | 1 |
| 11_10641 | 5H | 69.9 | 0.11 | 0.78 | 1 |
| 12_10203 | 5H | 69.9 | 0.00 | 1.00 | 1 |
| 12_20278 | 5H | 69.9 | 0.00 | 1.00 | 1 |
| 12_30080 | 5H | 69.9 | 0.29 | 0.52 | 1 |
| 11_11249 | 5H | 70.48 | 0.37 | 0.43 | 1 |
| 11_20392 | 5H | 70.48 | 0.37 | 0.43 | 1 |
| 12_30007 | 5H | 70.48 | 0.37 | 0.42 | 1 |
| 12_20350 | 5H | 72.29 | 0.17 | 0.68 | 1 |
| 12_10548 | 5H | 74.06 | 0.00 | 1.00 | 1 |
| 12_20297 | 5H | 74.78 | 0.90 | 0.13 | 1 |
| 12_30719 | 5H | 74.78 | 0.00 | 1.00 | 1 |
| 11_20367 | 5H | 75.4 | 0.25 | 0.56 | 1 |
| 11_21001 | 5H | 75.4 | 0.23 | 0.59 | 1 |
| 12_10953 | 5H | 75.4 | 0.00 | 1.00 | 1 |
| 11_21309 | 5H | 76.3 | 0.24 | 0.58 | 1 |
| 12_20818 | 5H | 76.42 | 0.00 | 1.00 | 1 |
| 11_20236 | 5H | 80.61 | 0.23 | 0.58 | 1 |
| 11_21133 | 5H | 80.61 | 0.06 | 0.86 | 1 |
| 12_10634 | 5H | 80.61 | 0.06 | 0.86 | 1 |
| 12_21128 | 5H | 80.61 | 0.00 | 1.00 | 1 |
| 12_10633 | 5H | 82.76 | 0.00 | 1.00 | 1 |
| 11_20096 | 5H | 84.51 | 0.00 | 1.00 | 1 |
| 11_10167 | 5H | 85.21 | 0.00 | 1.00 | 1 |
| 11_20246 | 5H | 85.21 | 0.44 | 0.37 | 1 |
| 12_10140 | 5H | 85.21 | 0.00 | 1.00 | 1 |
| 11_20736 | 5H | 85.93 | 0.00 | 1.00 | 1 |
| 12_11515 | 5H | 85.93 | 0.00 | 1.00 | 1 |
| 11_11355 | 5H | 86.63 | 1.56 | 0.03 | 1 |
| 11_20645 | 5H | 87.35 | 0.25 | 0.56 | 1 |
| 11_21445 | 5H | 87.35 | 0.00 | 1.00 | 1 |
| 12_21186 | 5H | 87.35 | 0.00 | 1.00 | 1 |
| 12_30314 | 5H | 87.35 | 0.05 | 0.90 | 1 |
| 11_21480 | 5H | 89.38 | 0.27 | 0.54 | 1 |
| 12_31427 | 5H | 90.84 | 1.18 | 0.07 | 1 |
| 12_10674 | 5H | 91.56 | 0.00 | 1.00 | 1 |
| 12_31361 | 5H | 91.56 | 0.04 | 0.91 | 1 |
| 11_11290 | 5H | 94.43 | 0.18 | 0.66 | 1 |
| 11_21150 | 5H | 94.43 | 1.28 | 0.05 | 1 |
| 12_10930 | 5H | 94.43 | 0.28 | 0.52 | 1 |
| 12_11106 | 5H | 94.43 | 0.14 | 0.73 | 1 |
| 12_21497 | 5H | 94.43 | 0.11 | 0.78 | 1 |
| 11_10578 | 5H | 95.08 | 0.27 | 0.53 | 1 |
| 11_20497 | 5H | 95.78 | 0.03 | 0.94 | 1 |
| 11_20526 | 5H | 99.56 | 0.00 | 1.00 | 1 |
| 12_31271 | 5H | 99.56 | 0.00 | 1.00 | 1 |
| 11_10518 | 5H | 100.28 | 0.21 | 0.62 | 1 |
| 11_10771 | 5H | 100.28 | 0.26 | 0.55 | 1 |
| 11_11473 | 5H | 100.28 | 0.08 | 0.83 | 1 |
| 11_20097 | 5H | 100.28 | 0.28 | 0.53 | 1 |
| 11_20449 | 5H | 100.28 | 0.06 | 0.86 | 1 |
| 12_11134 | 5H | 100.28 | 0.00 | 1.00 | 1 |
| 12_30533 | 5H | 100.28 | 0.14 | 0.72 | 1 |
| 12_30834 | 5H | 100.28 | 0.23 | 0.59 | 1 |
| 11_20850 | 5H | 102.06 | 0.04 | 0.91 | 1 |
| 12_10408 | 5H | 102.06 | 0.06 | 0.86 | 1 |
| 11_10622 | 5H | 103.01 | 0.41 | 0.39 | 1 |
| 11_21421 | 5H | 103.72 | 0.02 | 0.96 | 1 |
| 11_10414 | 5H | 103.92 | 0.75 | 0.18 | 1 |
| 11_20327 | 5H | 103.92 | 0.65 | 0.22 | 1 |
| 11_11350 | 5H | 104.5 | 0.00 | 1.00 | 1 |
| 12_20403 | 5H | 105.22 | 0.00 | 1.00 | 1 |
| 12_30098 | 5H | 105.22 | 0.13 | 0.75 | 1 |
| 11_20018 | 5H | 106.09 | 0.13 | 0.75 | 1 |
| 11_20134 | 5H | 106.16 | 0.38 | 0.42 | 1 |
| 11_10024 | 5H | 107.59 | 0.22 | 0.61 | 1 |
| 11_20549 | 5H | 108.01 | 0.14 | 0.73 | 1 |
| 11_10834 | 5H | 108.18 | 0.30 | 0.50 | 1 |
| 11_20320 | 5H | 108.18 | 0.16 | 0.70 | 1 |
| 11_21314 | 5H | 108.18 | 0.35 | 0.44 | 1 |
| 11_21321 | 5H | 108.18 | 0.48 | 0.33 | 1 |
| 12_10077 | 5H | 108.18 | 0.59 | 0.26 | 1 |
| 12_10844 | 5H | 108.18 | 0.24 | 0.57 | 1 |
| 12_30846 | 5H | 108.18 | 0.53 | 0.30 | 1 |
| 12_30847 | 5H | 108.18 | 0.53 | 0.30 | 1 |
| 12_30850 | 5H | 108.18 | 0.00 | 1.00 | 1 |
| 12_30852 | 5H | 108.18 | 0.55 | 0.28 | 1 |
| 12_30854 | 5H | 108.18 | 0.55 | 0.28 | 1 |
| 12_30855 | 5H | 108.18 | 0.34 | 0.45 | 1 |
| 12_31417 | 5H | 108.18 | 0.19 | 0.65 | 1 |
| 11_20795 | 5H | 108.63 | 0.51 | 0.31 | 1 |
| 11_21168 | 5H | 109.56 | 0.31 | 0.49 | 1 |
| 11_20805 | 5H | 110.26 | 0.41 | 0.39 | 1 |
| 11_21061 | 5H | 110.26 | 0.04 | 0.92 | 1 |
| 12_10507 | 5H | 110.26 | 0.23 | 0.59 | 1 |
| 12_30705 | 5H | 110.26 | 0.24 | 0.58 | 1 |
| 12_31236 | 5H | 110.26 | 0.37 | 0.43 | 1 |
| 11_11273 | 5H | 111.68 | 0.09 | 0.82 | 1 |
| 11_10477 | 5H | 113.11 | 0.02 | 0.96 | 1 |
| 12_30056 | 5H | 113.11 | 0.00 | 1.00 | 1 |
| 12_30456 | 5H | 113.11 | 0.49 | 0.33 | 1 |
| 11_11341 | 5H | 113.83 | 0.01 | 0.98 | 1 |
| 12_30619 | 5H | 113.83 | 0.49 | 0.33 | 1 |
| 11_11200 | 5H | 117.47 | 1.19 | 0.06 | 1 |
| 12_11245 | 5H | 118.77 | 0.11 | 0.78 | 1 |
| 11_10094 | 5H | 122.38 | 0.57 | 0.27 | 1 |
| 11_20629 | 5H | 122.38 | 0.51 | 0.31 | 1 |
| 11_20637 | 5H | 123.08 | 0.03 | 0.93 | 1 |
| 12_11298 | 5H | 123.33 | 0.01 | 0.98 | 1 |
| 11_20127 | 5H | 123.52 | 0.11 | 0.79 | 1 |
| 12_30524 | 5H | 123.52 | 0.14 | 0.73 | 1 |
| 12_31278 | 5H | 124.23 | 0.09 | 0.81 | 1 |
| 11_11507 | 5H | 125.81 | 0.33 | 0.47 | 1 |
| 12_20045 | 5H | 127.24 | 0.01 | 0.98 | 1 |
| 11_11456 | 5H | 127.96 | 0.16 | 0.70 | 1 |
| 12_30377 | 5H | 128.69 | 0.46 | 0.35 | 1 |
| 11_10360 | 5H | 129.41 | 0.28 | 0.52 | 1 |
| 11_11024 | 5H | 129.41 | 0.00 | 1.00 | 1 |
| 11_20003 | 5H | 129.41 | 0.26 | 0.55 | 1 |
| 11_20347 | 5H | 129.41 | 0.47 | 0.34 | 1 |
| 11_20653 | 5H | 129.41 | 0.27 | 0.54 | 1 |
| 11_21203 | 5H | 129.41 | 0.33 | 0.47 | 1 |
| 11_21325 | 5H | 129.41 | 0.03 | 0.93 | 1 |
| 12_10228 | 5H | 129.41 | 0.00 | 1.00 | 1 |
| 12_11535 | 5H | 129.41 | 0.00 | 0.99 | 1 |
| 12_30169 | 5H | 129.41 | 0.02 | 0.96 | 1 |
| 12_30590 | 5H | 129.41 | 0.43 | 0.37 | 1 |
| 12_30611 | 5H | 129.41 | 0.33 | 0.47 | 1 |
| 11_10805 | 5H | 130.13 | 0.51 | 0.31 | 1 |
| 11_20300 | 5H | 130.13 | 0.40 | 0.39 | 1 |
| 11_11375 | 5H | 130.84 | 0.23 | 0.59 | 1 |
| 12_30067 | 5H | 131.56 | 0.13 | 0.75 | 1 |
| 12_11472 | 5H | 132.48 | 0.16 | 0.70 | 1 |
| 11_10705 | 5H | 132.63 | 0.35 | 0.45 | 1 |
| 11_11090 | 5H | 132.63 | 0.03 | 0.94 | 1 |
| 11_20259 | 5H | 132.63 | 0.12 | 0.75 | 1 |
| 11_20298 | 5H | 132.63 | 0.28 | 0.53 | 1 |
| 11_21177 | 5H | 132.63 | 0.05 | 0.89 | 1 |
| 11_21247 | 5H | 132.63 | 0.01 | 0.97 | 1 |
| 12_21471 | 5H | 132.63 | 0.17 | 0.67 | 1 |
| 11_20487 | 5H | 134.6 | 0.06 | 0.88 | 1 |
| 11_10783 | 5H | 135.72 | 0.30 | 0.50 | 1 |
| 12_30883 | 5H | 135.72 | 0.04 | 0.92 | 1 |
| 12_30668 | 5H | 136.43 | 0.24 | 0.57 | 1 |
| 12_30869 | 5H | 136.43 | 0.24 | 0.57 | 1 |
| 11_10095 | 5H | 137.16 | 0.24 | 0.57 | 1 |
| 11_10855 | 5H | 137.16 | 0.01 | 0.97 | 1 |
| 11_11080 | 5H | 137.16 | 0.28 | 0.53 | 1 |
| 11_20884 | 5H | 137.16 | 0.01 | 0.98 | 1 |
| 11_21241 | 5H | 137.16 | 0.32 | 0.48 | 1 |
| 12_30929 | 5H | 137.16 | 2.50 | 0.00 | 1 |
| 12_30930 | 5H | 137.16 | 0.20 | 0.63 | 1 |
| 12_31237 | 5H | 137.16 | 0.22 | 0.60 | 1 |
| 12_30635 | 5H | 140.76 | 0.01 | 0.98 | 1 |
| 11_10755 | 5H | 142.2 | 0.16 | 0.70 | 1 |
| 11_10845 | 5H | 142.2 | 0.12 | 0.76 | 1 |
| 11_11071 | 5H | 142.2 | 0.13 | 0.75 | 1 |
| 11_11532 | 5H | 142.2 | 0.15 | 0.72 | 1 |
| 11_21289 | 5H | 142.2 | 0.09 | 0.82 | 1 |
| 12_31366 | 5H | 142.2 | 0.13 | 0.75 | 1 |
| 11_10819 | 5H | 143.92 | 0.04 | 0.91 | 1 |
| 11_20375 | 5H | 143.92 | 0.04 | 0.91 | 1 |
| 12_30556 | 5H | 143.92 | 0.01 | 0.99 | 1 |
| 11_10292 | 5H | 144.63 | 0.06 | 0.87 | 1 |
| 12_31217 | 5H | 144.63 | 0.06 | 0.88 | 1 |
| 11_11092 | 5H | 145.35 | 0.11 | 0.77 | 1 |
| 12_30833 | 5H | 145.35 | 2.20 | 0.01 | 1 |
| 11_10104 | 5H | 146 | 0.08 | 0.83 | 1 |
| 11_20568 | 5H | 146 | 0.03 | 0.94 | 1 |
| 11_20676 | 5H | 146 | 0.08 | 0.84 | 1 |
| 11_20731 | 5H | 146 | 0.09 | 0.81 | 1 |
| 11_21077 | 5H | 146 | 0.02 | 0.96 | 1 |
| 12_20830 | 5H | 146 | 0.00 | 1.00 | 1 |
| 12_31182 | 5H | 146 | 0.00 | 1.00 | 1 |
| 12_31234 | 5H | 146 | 0.00 | 1.00 | 1 |
| 11_10557 | 5H | 147.4 | 1.44 | 0.04 | 1 |
| 12_30400 | 5H | 149.1 | 0.27 | 0.54 | 1 |
| 12_30580 | 5H | 149.1 | 0.02 | 0.97 | 1 |
| 11_20791 | 5H | 149.64 | 0.66 | 0.22 | 1 |
| 11_21297 | 5H | 149.64 | 0.99 | 0.10 | 1 |
| 11_20388 | 5H | 150.34 | 0.61 | 0.24 | 1 |
| 11_10080 | 5H | 151.36 | 0.02 | 0.96 | 1 |
| 11_10363 | 5H | 151.36 | 0.05 | 0.89 | 1 |
| 11_11441 | 5H | 151.36 | 0.51 | 0.31 | 1 |
| 11_20100 | 5H | 151.36 | 0.97 | 0.11 | 1 |
| 11_21360 | 5H | 151.36 | 0.22 | 0.60 | 1 |
| 12_10333 | 5H | 151.36 | 0.72 | 0.19 | 1 |
| 12_10904 | 5H | 151.36 | 0.10 | 0.79 | 1 |
| 12_21462 | 5H | 151.36 | 0.01 | 0.98 | 1 |
| 12_30062 | 5H | 151.36 | 0.05 | 0.89 | 1 |
| 12_30183 | 5H | 151.36 | 0.05 | 0.89 | 1 |
| 12_31050 | 5H | 151.36 | 1.02 | 0.10 | 1 |
| 12_31165 | 5H | 151.36 | 0.23 | 0.59 | 1 |
| 12_31206 | 5H | 151.36 | 0.02 | 0.95 | 1 |
| 12_30795 | 5H | 152.79 | 0.07 | 0.85 | 1 |
| 12_31221 | 5H | 152.79 | 0.07 | 0.86 | 1 |
| 11_10217 | 5H | 153.51 | 0.16 | 0.69 | 1 |
| 11_10589 | 5H | 153.51 | 0.16 | 0.69 | 1 |
| 11_20104 | 5H | 153.51 | 0.22 | 0.61 | 1 |
| 11_20573 | 5H | 153.51 | 0.06 | 0.87 | 1 |
| 11_21355 | 5H | 153.51 | 0.10 | 0.79 | 1 |
| 12_10016 | 5H | 153.51 | 0.26 | 0.55 | 1 |
| 12_30830 | 5H | 153.51 | 0.06 | 0.88 | 1 |
| 11_11490 | 5H | 153.6 | 1.39 | 0.04 | 1 |
| 12_20186 | 5H | 154.41 | 0.00 | 1.00 | 1 |
| 11_11497 | 5H | 155.13 | 1.51 | 0.03 | 1 |
| 11_10901 | 5H | 158.37 | 0.07 | 0.86 | 1 |
| 11_10820 | 5H | 159.09 | 0.09 | 0.82 | 1 |
| 11_11185 | 5H | 159.09 | 0.79 | 0.16 | 1 |
| 11_21041 | 5H | 159.09 | 0.08 | 0.84 | 1 |
| 11_10161 | 5H | 159.79 | 0.80 | 0.16 | 1 |
| 11_10528 | 5H | 159.79 | 0.57 | 0.27 | 1 |
| 11_10536 | 5H | 159.79 | 0.08 | 0.83 | 1 |
| 11_10582 | 5H | 159.79 | 0.24 | 0.57 | 1 |
| 11_10741 | 5H | 159.79 | 0.55 | 0.28 | 1 |
| 11_10902 | 5H | 159.79 | 0.51 | 0.31 | 1 |
| 11_20078 | 5H | 159.79 | 0.26 | 0.54 | 1 |
| 11_20545 | 5H | 159.79 | 0.61 | 0.24 | 1 |
| 11_21024 | 5H | 159.79 | 0.28 | 0.53 | 1 |
| 11_21452 | 5H | 159.79 | 0.55 | 0.28 | 1 |
| 12_20317 | 5H | 159.79 | 0.00 | 1.00 | 1 |
| 12_30165 | 5H | 159.79 | 0.48 | 0.33 | 1 |
| 12_30238 | 5H | 159.79 | 0.00 | 1.00 | 1 |
| 12_30759 | 5H | 159.79 | 0.69 | 0.20 | 1 |
| 11_20560 | 5H | 160.51 | 0.08 | 0.84 | 1 |
| 11_20876 | 5H | 160.51 | 0.08 | 0.84 | 1 |
| 11_10336 | 5H | 161.58 | 0.16 | 0.69 | 1 |
| 11_11464 | 5H | 161.58 | 0.16 | 0.69 | 1 |
| 11_20646 | 5H | 161.58 | 0.10 | 0.79 | 1 |
| 11_20988 | 5H | 161.58 | 0.01 | 0.97 | 1 |
| 11_21018 | 5H | 161.58 | 0.08 | 0.84 | 1 |
| 12_10273 | 5H | 161.58 | 0.59 | 0.26 | 1 |
| 12_11325 | 5H | 161.58 | 0.00 | 1.00 | 1 |
| 12_30162 | 5H | 161.58 | 0.26 | 0.55 | 1 |
| 12_30642 | 5H | 161.58 | 0.13 | 0.74 | 1 |
| 12_31375 | 5H | 161.58 | 0.33 | 0.47 | 1 |
| 11_20334 | 5H | 166.63 | 0.02 | 0.95 | 1 |
| 11_20934 | 5H | 166.63 | 0.15 | 0.71 | 1 |
| 11_20829 | 5H | 168.79 | 0.05 | 0.90 | 1 |
| 12_30666 | 5H | 169.51 | 0.11 | 0.78 | 1 |
| 11_11216 | 5H | 171.66 | 0.01 | 0.97 | 1 |
| 11_20546 | 5H | 172.38 | 0.23 | 0.58 | 1 |
| 11_20686 | 5H | 172.38 | 0.40 | 0.40 | 1 |
| 12_10769 | 5H | 172.38 | 0.07 | 0.85 | 1 |
| 12_30087 | 5H | 172.38 | 0.00 | 1.00 | 1 |
| 11_10869 | 5H | 173.08 | 0.56 | 0.28 | 1 |
| 11_10778 | 5H | 175.9 | 0.09 | 0.81 | 1 |
| 11_10600 | 5H | 176.62 | 0.10 | 0.80 | 1 |
| 11_21012 | 5H | 176.62 | 0.00 | 1.00 | 1 |
| 12_20867 | 5H | 176.62 | 0.09 | 0.81 | 1 |
| 12_21290 | 5H | 176.62 | 0.09 | 0.81 | 1 |
| 11_21141 | 5H | 177.07 | 0.18 | 0.65 | 1 |
| 11_20536 | 5H | 177.65 | 0.16 | 0.69 | 1 |
| 12_21009 | 5H | 177.65 | 0.09 | 0.81 | 1 |
| 12_31404 | 5H | 177.65 | 0.00 | 1.00 | 1 |
| 12_11010 | 5H | 178.43 | 0.74 | 0.18 | 1 |
| 12_11450 | 5H | 178.43 | 0.53 | 0.30 | 1 |
| 12_20816 | 5H | 178.43 | 0.07 | 0.84 | 1 |
| 11_10254 | 5H | 179.06 | 0.15 | 0.71 | 1 |
| 11_21138 | 5H | 179.64 | 0.24 | 0.58 | 1 |
| 12_30656 | 5H | 179.64 | 0.23 | 0.58 | 1 |
| 11_10736 | 5H | 180.71 | 0.07 | 0.85 | 1 |
| 12_30494 | 5H | 180.71 | 0.66 | 0.22 | 1 |
| 11_10236 | 5H | 181.43 | 0.42 | 0.38 | 1 |
| 11_20022 | 5H | 181.43 | 0.10 | 0.80 | 1 |
| 11_20189 | 5H | 181.43 | 0.27 | 0.54 | 1 |
| 12_30504 | 5H | 182.16 | 0.12 | 0.76 | 1 |
| 11_20897 | 5H | 182.88 | 0.04 | 0.90 | 1 |
| 12_30577 | 5H | 182.88 | 0.23 | 0.59 | 1 |
| 12_30769 | 5H | 182.88 | 0.00 | 0.99 | 1 |
| 12_31352 | 5H | 182.88 | 0.74 | 0.18 | 1 |
| 12_21393 | 5H | 186.48 | 0.00 | 1.00 | 1 |
| 11_21155 | 5H | 187.38 | 0.07 | 0.85 | 1 |
| 11_10310 | 5H | 187.96 | 0.06 | 0.86 | 1 |
| 11_11364 | 5H | 189.6 | 0.09 | 0.81 | 1 |
| 11_20786 | 5H | 189.6 | 0.51 | 0.31 | 1 |
| 11_21052 | 5H | 189.6 | 0.10 | 0.79 | 1 |
| 12_31292 | 5H | 189.6 | 0.12 | 0.76 | 1 |
| 12_11192 | 5H | 191.39 | 0.37 | 0.43 | 1 |
| 11_10401 | 5H | 191.97 | 0.38 | 0.42 | 1 |
| 12_10732 | 5H | 191.97 | 0.10 | 0.79 | 1 |
| 12_30360 | 5H | 191.97 | 0.25 | 0.56 | 1 |
| 12_31210 | 5H | 191.97 | 0.10 | 0.79 | 1 |
| 12_31481 | 5H | 191.97 | 0.03 | 0.93 | 1 |
| 12_10857 | 5H | 194.84 | 0.19 | 0.64 | 1 |
| 11_20402 | 5H | 195.42 | 0.13 | 0.74 | 1 |
| 11_20132 | 5H | 196.12 | 0.51 | 0.31 | 1 |
| 12_10322 | 5H | 196.12 | 0.34 | 0.46 | 1 |
| 12_21356 | 5H | 196.12 | 0.00 | 1.00 | 1 |
| 12_30958 | 5H | 196.12 | 0.20 | 0.63 | 1 |
| 12_31123 | 5H | 196.85 | 0.33 | 0.47 | 1 |
| 11_10496 | 6H | 0 | 0.66 | 0.22 | 1 |
| 11_11329 | 6H | 0 | 0.06 | 0.88 | 1 |
| 11_11406 | 6H | 0 | 0.16 | 0.70 | 1 |
| 11_20212 | 6H | 0 | 0.20 | 0.63 | 1 |
| 11_20232 | 6H | 0 | 0.06 | 0.88 | 1 |
| 11_20292 | 6H | 0 | 0.13 | 0.73 | 1 |
| 11_20336 | 6H | 0 | 0.56 | 0.27 | 1 |
| 11_20465 | 6H | 0 | 1.05 | 0.09 | 1 |
| 11_21425 | 6H | 0 | 0.00 | 1.00 | 1 |
| 12_30319 | 6H | 0 | 0.19 | 0.65 | 1 |
| 11_20493 | 6H | 1.34 | 0.17 | 0.67 | 1 |
| 11_20881 | 6H | 1.34 | 0.18 | 0.67 | 1 |
| 11_20886 | 6H | 1.34 | 0.19 | 0.64 | 1 |
| 11_10669 | 6H | 2.27 | 0.11 | 0.78 | 1 |
| 11_10120 | 6H | 3.11 | 0.07 | 0.85 | 1 |
| 11_21521 | 6H | 3.11 | 0.04 | 0.92 | 1 |
| 11_20882 | 6H | 3.23 | 0.04 | 0.91 | 1 |
| 12_30651 | 6H | 4.41 | 0.05 | 0.90 | 1 |
| 11_20294 | 6H | 5.4 | 0.10 | 0.79 | 1 |
| 11_21204 | 6H | 6.07 | 0.12 | 0.76 | 1 |
| 11_20262 | 6H | 8.07 | 0.07 | 0.85 | 1 |
| 11_21032 | 6H | 9.06 | 0.09 | 0.82 | 1 |
| 11_11479 | 6H | 12.54 | 0.07 | 0.85 | 1 |
| 11_20415 | 6H | 13.21 | 0.28 | 0.53 | 1 |
| 12_30010 | 6H | 15.63 | 0.00 | 1.00 | 1 |
| 11_10165 | 6H | 16.97 | 0.17 | 0.68 | 1 |
| 12_10554 | 6H | 16.97 | 0.09 | 0.81 | 1 |
| 12_30842 | 6H | 16.97 | 0.18 | 0.66 | 1 |
| 11_10064 | 6H | 21.69 | 0.03 | 0.94 | 1 |
| 11_10023 | 6H | 22.35 | 0.02 | 0.96 | 1 |
| 11_21246 | 6H | 22.35 | 0.05 | 0.89 | 1 |
| 12_30843 | 6H | 22.35 | 0.01 | 0.99 | 1 |
| 11_10136 | 6H | 24.36 | 0.01 | 0.97 | 1 |
| 11_10868 | 6H | 24.36 | 0.10 | 0.80 | 1 |
| 11_20315 | 6H | 24.36 | 0.02 | 0.97 | 1 |
| 11_10676 | 6H | 28.39 | 0.18 | 0.67 | 1 |
| 12_30697 | 6H | 29.05 | 0.04 | 0.91 | 1 |
| 12_30673 | 6H | 30.06 | 0.05 | 0.90 | 1 |
| 12_31485 | 6H | 30.06 | 0.03 | 0.94 | 1 |
| 12_31308 | 6H | 30.72 | 0.09 | 0.81 | 1 |
| 11_10799 | 6H | 31.73 | 0.28 | 0.52 | 1 |
| 11_10994 | 6H | 31.73 | 0.04 | 0.91 | 1 |
| 11_10939 | 6H | 33.74 | 0.39 | 0.41 | 1 |
| 11_10427 | 6H | 34.4 | 0.32 | 0.48 | 1 |
| 12_30358 | 6H | 35.07 | 0.81 | 0.15 | 1 |
| 12_30521 | 6H | 38.42 | 0.35 | 0.44 | 1 |
| 12_30361 | 6H | 40.79 | 0.71 | 0.20 | 1 |
| 11_10061 | 6H | 42.36 | 0.04 | 0.91 | 1 |
| 11_10129 | 6H | 42.36 | 0.72 | 0.19 | 1 |
| 11_10494 | 6H | 42.36 | 0.05 | 0.90 | 1 |
| 11_10882 | 6H | 42.36 | 0.09 | 0.81 | 1 |
| 11_20052 | 6H | 42.36 | 0.11 | 0.78 | 1 |
| 11_21030 | 6H | 42.36 | 0.33 | 0.47 | 1 |
| 12_11455 | 6H | 42.36 | 0.10 | 0.80 | 1 |
| 12_30665 | 6H | 42.36 | 0.00 | 1.00 | 1 |
| 12_30783 | 6H | 42.36 | 0.08 | 0.83 | 1 |
| 11_10244 | 6H | 43.15 | 0.12 | 0.76 | 1 |
| 11_20936 | 6H | 43.15 | 0.05 | 0.90 | 1 |
| 12_30516 | 6H | 43.15 | 0.06 | 0.88 | 1 |
| 11_21281 | 6H | 43.83 | 0.31 | 0.49 | 1 |
| 11_10462 | 6H | 44.77 | 0.04 | 0.92 | 1 |
| 11_20743 | 6H | 44.77 | 0.00 | 1.00 | 1 |
| 11_10013 | 6H | 45.44 | 0.02 | 0.94 | 1 |
| 11_10817 | 6H | 45.44 | 0.05 | 0.89 | 1 |
| 11_11097 | 6H | 45.44 | 0.03 | 0.94 | 1 |
| 11_20707 | 6H | 45.44 | 0.05 | 0.89 | 1 |
| 12_10199 | 6H | 45.44 | 0.15 | 0.70 | 1 |
| 12_10575 | 6H | 45.44 | 0.15 | 0.70 | 1 |
| 12_10811 | 6H | 45.44 | 0.06 | 0.87 | 1 |
| 12_10910 | 6H | 45.44 | 0.14 | 0.73 | 1 |
| 12_30317 | 6H | 45.44 | 0.01 | 0.99 | 1 |
| 11_10539 | 6H | 46.11 | 0.03 | 0.92 | 1 |
| 11_10461 | 6H | 48.74 | 0.02 | 0.95 | 1 |
| 12_30133 | 6H | 48.74 | 0.03 | 0.94 | 1 |
| 12_30316 | 6H | 48.74 | 0.04 | 0.91 | 1 |
| 12_30658 | 6H | 48.74 | 0.03 | 0.94 | 1 |
| 11_20291 | 6H | 49.4 | 0.01 | 0.97 | 1 |
| 12_30510 | 6H | 49.4 | 0.01 | 0.97 | 1 |
| 12_30751 | 6H | 49.4 | 0.04 | 0.91 | 1 |
| 12_31092 | 6H | 49.4 | 0.02 | 0.95 | 1 |
| 12_31274 | 6H | 49.4 | 0.09 | 0.82 | 1 |
| 12_31433 | 6H | 49.4 | 0.09 | 0.81 | 1 |
| 11_11205 | 6H | 50.07 | 0.04 | 0.92 | 1 |
| 11_20675 | 6H | 50.07 | 0.07 | 0.85 | 1 |
| 12_30782 | 6H | 50.07 | 0.15 | 0.71 | 1 |
| 12_21449 | 6H | 50.74 | 0.00 | 1.00 | 1 |
| 12_30569 | 6H | 51.41 | 0.07 | 0.85 | 1 |
| 11_10003 | 6H | 52.75 | 0.05 | 0.90 | 1 |
| 11_20720 | 6H | 52.75 | 0.43 | 0.37 | 1 |
| 12_10497 | 6H | 52.75 | 0.00 | 1.00 | 1 |
| 12_11104 | 6H | 52.75 | 0.00 | 1.00 | 1 |
| 12_11353 | 6H | 52.75 | 0.05 | 0.90 | 1 |
| 12_20381 | 6H | 52.75 | 0.00 | 1.00 | 1 |
| 12_30032 | 6H | 52.75 | 0.05 | 0.90 | 1 |
| 12_30120 | 6H | 52.75 | 0.65 | 0.22 | 1 |
| 12_30430 | 6H | 52.75 | 0.39 | 0.41 | 1 |
| 12_30473 | 6H | 52.75 | 0.06 | 0.87 | 1 |
| 11_21473 | 6H | 53.29 | 0.33 | 0.47 | 1 |
| 11_21158 | 6H | 53.95 | 0.41 | 0.39 | 1 |
| 11_10962 | 6H | 54.6 | 0.65 | 0.22 | 1 |
| 11_20651 | 6H | 54.6 | 0.71 | 0.20 | 1 |
| 11_20656 | 6H | 54.6 | 0.19 | 0.65 | 1 |
| 11_21014 | 6H | 54.6 | 0.65 | 0.22 | 1 |
| 12_30021 | 6H | 54.6 | 0.13 | 0.74 | 1 |
| 12_30441 | 6H | 54.6 | 0.39 | 0.41 | 1 |
| 12_30802 | 6H | 54.6 | 0.15 | 0.71 | 1 |
| 12_31004 | 6H | 54.6 | 0.61 | 0.24 | 1 |
| 12_31005 | 6H | 54.6 | 0.39 | 0.40 | 1 |
| 12_31007 | 6H | 54.6 | 0.08 | 0.84 | 1 |
| 11_10954 | 6H | 55 | 0.65 | 0.23 | 1 |
| 11_11312 | 6H | 55 | 0.67 | 0.21 | 1 |
| 11_20600 | 6H | 55 | 0.61 | 0.24 | 1 |
| 12_10591 | 6H | 55 | 0.00 | 1.00 | 1 |
| 12_30230 | 6H | 55 | 0.00 | 1.00 | 1 |
| 12_30567 | 6H | 55 | 0.00 | 1.00 | 1 |
| 12_31509 | 6H | 55 | 0.00 | 1.00 | 1 |
| 12_20142 | 6H | 55.36 | 0.00 | 1.00 | 1 |
| 12_30508 | 6H | 55.36 | 0.74 | 0.18 | 1 |
| 11_10323 | 6H | 55.65 | 0.73 | 0.19 | 1 |
| 11_10749 | 6H | 55.65 | 0.74 | 0.18 | 1 |
| 11_10848 | 6H | 55.65 | 0.74 | 0.18 | 1 |
| 11_11153 | 6H | 55.65 | 0.13 | 0.75 | 1 |
| 11_20329 | 6H | 55.65 | 0.67 | 0.21 | 1 |
| 11_20567 | 6H | 55.65 | 0.73 | 0.19 | 1 |
| 11_20572 | 6H | 55.65 | 0.73 | 0.19 | 1 |
| 11_20785 | 6H | 55.65 | 0.74 | 0.18 | 1 |
| 11_20799 | 6H | 55.65 | 0.72 | 0.19 | 1 |
| 11_20854 | 6H | 55.65 | 0.74 | 0.18 | 1 |
| 11_20946 | 6H | 55.65 | 0.73 | 0.19 | 1 |
| 11_21124 | 6H | 55.65 | 0.43 | 0.37 | 1 |
| 11_21216 | 6H | 55.65 | 0.74 | 0.18 | 1 |
| 12_11140 | 6H | 55.65 | 0.90 | 0.13 | 1 |
| 12_11442 | 6H | 55.65 | 0.90 | 0.13 | 1 |
| 12_11487 | 6H | 55.65 | 0.00 | 1.00 | 1 |
| 12_21482 | 6H | 55.65 | 0.04 | 0.90 | 1 |
| 12_30305 | 6H | 55.65 | 0.15 | 0.71 | 1 |
| 12_30765 | 6H | 55.65 | 0.74 | 0.18 | 1 |
| 12_30837 | 6H | 55.65 | 0.74 | 0.18 | 1 |
| 12_31187 | 6H | 55.65 | 0.74 | 0.18 | 1 |
| 12_31249 | 6H | 55.65 | 0.74 | 0.18 | 1 |
| 12_31443 | 6H | 55.65 | 0.73 | 0.18 | 1 |
| 11_10227 | 6H | 55.94 | 1.06 | 0.09 | 1 |
| 11_10377 | 6H | 55.94 | 0.30 | 0.50 | 1 |
| 11_10513 | 6H | 55.94 | 0.64 | 0.23 | 1 |
| 11_20835 | 6H | 55.94 | 1.04 | 0.09 | 1 |
| 12_10278 | 6H | 55.94 | 0.00 | 1.00 | 1 |
| 12_10345 | 6H | 55.94 | 0.00 | 1.00 | 1 |
| 12_11181 | 6H | 55.94 | 0.00 | 1.00 | 1 |
| 12_20463 | 6H | 55.94 | 0.29 | 0.52 | 1 |
| 12_21114 | 6H | 55.94 | 0.31 | 0.49 | 1 |
| 12_30311 | 6H | 55.94 | 0.43 | 0.37 | 1 |
| 12_30465 | 6H | 55.94 | 0.67 | 0.21 | 1 |
| 12_30511 | 6H | 55.94 | 0.68 | 0.21 | 1 |
| 12_30596 | 6H | 55.94 | 0.74 | 0.18 | 1 |
| 12_30681 | 6H | 55.94 | 0.99 | 0.10 | 1 |
| 12_30749 | 6H | 55.94 | 0.67 | 0.21 | 1 |
| 12_30856 | 6H | 55.94 | 0.68 | 0.21 | 1 |
| 12_31006 | 6H | 55.94 | 0.67 | 0.21 | 1 |
| 12_31178 | 6H | 55.94 | 0.28 | 0.52 | 1 |
| 11_20184 | 6H | 56.48 | 0.04 | 0.92 | 1 |
| 12_30144 | 6H | 56.48 | 0.04 | 0.92 | 1 |
| 12_30857 | 6H | 56.48 | 0.73 | 0.19 | 1 |
| 11_11067 | 6H | 58.01 | 0.55 | 0.28 | 1 |
| 12_11253 | 6H | 58.01 | 0.05 | 0.89 | 1 |
| 11_21339 | 6H | 58.55 | 0.72 | 0.19 | 1 |
| 12_10803 | 6H | 58.55 | 0.04 | 0.90 | 1 |
| 11_10964 | 6H | 59.56 | 0.78 | 0.17 | 1 |
| 11_20266 | 6H | 59.56 | 0.70 | 0.20 | 1 |
| 11_10189 | 6H | 60.23 | 0.01 | 0.98 | 1 |
| 11_10270 | 6H | 60.23 | 0.10 | 0.80 | 1 |
| 11_10635 | 6H | 60.23 | 0.02 | 0.96 | 1 |
| 11_20058 | 6H | 60.23 | 0.05 | 0.88 | 1 |
| 11_21310 | 6H | 60.23 | 0.06 | 0.88 | 1 |
| 12_10758 | 6H | 60.23 | 0.08 | 0.84 | 1 |
| 12_11321 | 6H | 60.23 | 0.00 | 1.00 | 1 |
| 12_30346 | 6H | 60.23 | 0.02 | 0.95 | 1 |
| 12_30804 | 6H | 60.23 | 0.02 | 0.95 | 1 |
| 12_30844 | 6H | 60.9 | 0.00 | 1.00 | 1 |
| 11_11483 | 6H | 63.27 | 0.07 | 0.85 | 1 |
| 11_21298 | 6H | 63.27 | 0.01 | 0.98 | 1 |
| 12_11475 | 6H | 63.27 | 0.05 | 0.90 | 1 |
| 11_21069 | 6H | 63.95 | 0.95 | 0.11 | 1 |
| 11_10455 | 6H | 64.36 | 0.69 | 0.20 | 1 |
| 11_10781 | 6H | 64.36 | 0.06 | 0.87 | 1 |
| 11_20287 | 6H | 64.36 | 0.24 | 0.58 | 1 |
| 11_20904 | 6H | 64.36 | 0.39 | 0.40 | 1 |
| 11_21225 | 6H | 64.36 | 0.21 | 0.62 | 1 |
| 11_21293 | 6H | 64.36 | 0.01 | 0.98 | 1 |
| 12_30637 | 6H | 64.36 | 0.01 | 0.99 | 1 |
| 11_10040 | 6H | 65.03 | 0.74 | 0.18 | 1 |
| 11_10124 | 6H | 65.03 | 0.01 | 0.98 | 1 |
| 11_11261 | 6H | 65.03 | 0.45 | 0.35 | 1 |
| 11_20015 | 6H | 65.03 | 0.14 | 0.72 | 1 |
| 11_20709 | 6H | 65.03 | 0.16 | 0.69 | 1 |
| 12_10392 | 6H | 65.03 | 0.00 | 1.00 | 1 |
| 12_20148 | 6H | 65.03 | 0.00 | 1.00 | 1 |
| 11_20714 | 6H | 67.04 | 0.03 | 0.93 | 1 |
| 12_10504 | 6H | 67.04 | 0.00 | 1.00 | 1 |
| 11_20468 | 6H | 67.7 | 0.20 | 0.63 | 1 |
| 11_20636 | 6H | 67.7 | 0.23 | 0.59 | 1 |
| 11_21469 | 6H | 67.7 | 0.25 | 0.56 | 1 |
| 12_31289 | 6H | 69.38 | 0.22 | 0.60 | 1 |
| 11_20620 | 6H | 70.04 | 0.22 | 0.61 | 1 |
| 11_20673 | 6H | 70.04 | 0.17 | 0.67 | 1 |
| 11_20892 | 6H | 70.04 | 0.16 | 0.69 | 1 |
| 12_10596 | 6H | 70.04 | 0.07 | 0.85 | 1 |
| 11_11349 | 6H | 71.08 | 0.26 | 0.55 | 1 |
| 11_20577 | 6H | 71.08 | 0.06 | 0.86 | 1 |
| 11_20784 | 6H | 71.08 | 0.65 | 0.22 | 1 |
| 12_31250 | 6H | 71.08 | 0.65 | 0.22 | 1 |
| 11_11459 | 6H | 71.87 | 0.22 | 0.61 | 1 |
| 11_21256 | 6H | 71.87 | 0.20 | 0.63 | 1 |
| 12_31101 | 6H | 71.87 | 0.77 | 0.17 | 1 |
| 11_10469 | 6H | 72.54 | 0.60 | 0.25 | 1 |
| 11_20053 | 6H | 72.54 | 0.00 | 0.99 | 1 |
| 11_20488 | 6H | 72.54 | 0.02 | 0.96 | 1 |
| 12_30236 | 6H | 72.54 | 0.00 | 1.00 | 1 |
| 12_30940 | 6H | 72.54 | 0.20 | 0.63 | 1 |
| 12_31111 | 6H | 72.54 | 0.64 | 0.23 | 1 |
| 12_31088 | 6H | 74.55 | 0.77 | 0.17 | 1 |
| 11_20682 | 6H | 75.21 | 0.08 | 0.82 | 1 |
| 11_20889 | 6H | 75.21 | 0.28 | 0.52 | 1 |
| 11_20969 | 6H | 75.21 | 0.05 | 0.90 | 1 |
| 11_20746 | 6H | 76.55 | 0.27 | 0.54 | 1 |
| 12_30573 | 6H | 76.55 | 0.07 | 0.85 | 1 |
| 12_30148 | 6H | 77.22 | 0.00 | 1.00 | 1 |
| 11_21224 | 6H | 77.89 | 0.59 | 0.26 | 1 |
| 11_10608 | 6H | 80.52 | 1.06 | 0.09 | 1 |
| 11_10220 | 6H | 81.17 | 0.58 | 0.26 | 1 |
| 11_11458 | 6H | 81.17 | 0.59 | 0.26 | 1 |
| 11_10331 | 6H | 81.22 | 0.03 | 0.93 | 1 |
| 11_10185 | 6H | 81.88 | 0.02 | 0.95 | 1 |
| 11_11246 | 6H | 81.88 | 0.02 | 0.96 | 1 |
| 11_20346 | 6H | 82.56 | 1.33 | 0.05 | 1 |
| 11_21404 | 6H | 82.56 | 0.85 | 0.14 | 1 |
| 11_11147 | 6H | 83.89 | 0.00 | 0.99 | 1 |
| 11_20654 | 6H | 84.51 | 0.06 | 0.87 | 1 |
| 11_10815 | 6H | 85.16 | 0.06 | 0.88 | 1 |
| 12_30698 | 6H | 86.88 | 0.02 | 0.95 | 1 |
| 11_10400 | 6H | 88.9 | 0.09 | 0.81 | 1 |
| 11_20783 | 6H | 88.9 | 0.51 | 0.31 | 1 |
| 11_21025 | 6H | 89.57 | 0.48 | 0.33 | 1 |
| 11_10202 | 6H | 90.15 | 0.71 | 0.19 | 1 |
| 12_30626 | 6H | 90.83 | 0.00 | 0.99 | 1 |
| 12_31235 | 6H | 91.79 | 0.85 | 0.14 | 1 |
| 11_20996 | 6H | 93.12 | 0.55 | 0.28 | 1 |
| 12_31225 | 6H | 93.12 | 0.30 | 0.50 | 1 |
| 11_11294 | 6H | 93.66 | 1.18 | 0.07 | 1 |
| 11_20728 | 6H | 93.66 | 0.89 | 0.13 | 1 |
| 11_10595 | 6H | 94.73 | 0.45 | 0.36 | 1 |
| 11_10978 | 6H | 94.73 | 0.06 | 0.87 | 1 |
| 11_20972 | 6H | 94.73 | 0.08 | 0.84 | 1 |
| 12_31432 | 6H | 94.73 | 0.38 | 0.42 | 1 |
| 11_10734 | 6H | 96.73 | 0.18 | 0.67 | 1 |
| 11_10015 | 6H | 97.39 | 1.14 | 0.07 | 1 |
| 11_10139 | 6H | 97.39 | 0.38 | 0.41 | 1 |
| 11_20118 | 6H | 97.39 | 0.66 | 0.22 | 1 |
| 11_20531 | 6H | 97.39 | 0.03 | 0.92 | 1 |
| 12_11494 | 6H | 97.39 | 0.00 | 1.00 | 1 |
| 12_30151 | 6H | 97.39 | 1.74 | 0.02 | 1 |
| 12_31042 | 6H | 97.39 | 0.00 | 1.00 | 1 |
| 12_31043 | 6H | 97.39 | 0.00 | 1.00 | 1 |
| 12_31044 | 6H | 97.39 | 0.87 | 0.13 | 1 |
| 12_31048 | 6H | 97.39 | 0.86 | 0.14 | 1 |
| 12_31049 | 6H | 97.39 | 0.87 | 0.13 | 1 |
| 12_31353 | 6H | 97.39 | 0.38 | 0.41 | 1 |
| 12_31115 | 6H | 100.1 | 0.06 | 0.88 | 1 |
| 11_20379 | 6H | 101.44 | 0.04 | 0.90 | 1 |
| 11_20036 | 6H | 105.6 | 0.42 | 0.38 | 1 |
| 11_20467 | 6H | 105.6 | 0.08 | 0.82 | 1 |
| 11_20725 | 6H | 105.6 | 0.08 | 0.82 | 1 |
| 11_21271 | 6H | 105.6 | 0.15 | 0.71 | 1 |
| 11_20355 | 6H | 110.32 | 0.68 | 0.21 | 1 |
| 12_30734 | 6H | 110.32 | 0.14 | 0.73 | 1 |
| 12_20448 | 6H | 110.99 | 2.36 | 0.00 | 1 |
| 11_10239 | 6H | 112.32 | 0.91 | 0.12 | 1 |
| 11_11534 | 6H | 112.32 | 0.06 | 0.87 | 1 |
| 11_20558 | 6H | 112.32 | 0.17 | 0.68 | 1 |
| 11_20733 | 6H | 112.32 | 0.06 | 0.87 | 1 |
| 12_31495 | 6H | 112.32 | 0.32 | 0.48 | 1 |
| 12_21477 | 6H | 117.01 | 0.00 | 1.00 | 1 |
| 11_10645 | 6H | 118.35 | 0.11 | 0.77 | 1 |
| 12_31277 | 6H | 118.35 | 0.20 | 0.64 | 1 |
| 11_10107 | 6H | 119.02 | 0.16 | 0.70 | 1 |
| 11_10175 | 6H | 119.02 | 0.05 | 0.90 | 1 |
| 12_10051 | 6H | 119.02 | 0.03 | 0.93 | 1 |
| 12_10071 | 6H | 119.02 | 0.00 | 1.00 | 1 |
| 12_31392 | 6H | 119.02 | 0.20 | 0.62 | 1 |
| 11_21455 | 6H | 119.67 | 0.21 | 0.61 | 1 |
| 11_21467 | 6H | 119.67 | 0.43 | 0.37 | 1 |
| 11_11187 | 6H | 121.22 | 0.52 | 0.30 | 1 |
| 12_30057 | 6H | 121.22 | 0.15 | 0.71 | 1 |
| 12_31126 | 6H | 121.22 | 0.10 | 0.79 | 1 |
| 11_20005 | 6H | 122.53 | 0.03 | 0.94 | 1 |
| 11_10748 | 6H | 123.84 | 0.03 | 0.93 | 1 |
| 11_11488 | 6H | 123.84 | 0.06 | 0.86 | 1 |
| 11_20211 | 6H | 123.84 | 0.01 | 0.97 | 1 |
| 11_10828 | 6H | 124.85 | 0.48 | 0.33 | 1 |
| 11_20687 | 6H | 124.85 | 0.24 | 0.57 | 1 |
| 11_20868 | 6H | 124.85 | 0.18 | 0.66 | 1 |
| 12_20907 | 6H | 124.85 | 0.00 | 1.00 | 1 |
| 12_30414 | 6H | 124.85 | 0.85 | 0.14 | 1 |
| 12_31283 | 6H | 124.85 | 0.17 | 0.68 | 1 |
| 12_31498 | 6H | 126.18 | 0.11 | 0.77 | 1 |
| 11_21112 | 6H | 126.85 | 0.07 | 0.85 | 1 |
| 11_10390 | 6H | 128.48 | 0.02 | 0.96 | 1 |
| 11_11111 | 6H | 128.48 | 0.25 | 0.56 | 1 |
| 11_20537 | 6H | 129.38 | 0.68 | 0.21 | 1 |
| 12_30627 | 6H | 129.38 | 1.00 | 0.10 | 1 |
| 12_30956 | 6H | 129.38 | 0.62 | 0.24 | 1 |
| 11_10209 | 7H | 0 | 0.30 | 0.50 | 1 |
| 11_10547 | 7H | 0 | 0.69 | 0.20 | 1 |
| 11_10700 | 7H | 0 | 0.25 | 0.56 | 1 |
| 11_10949 | 7H | 0 | 0.03 | 0.94 | 1 |
| 11_10956 | 7H | 0 | 0.38 | 0.42 | 1 |
| 11_10971 | 7H | 0 | 0.07 | 0.85 | 1 |
| 11_11222 | 7H | 0 | 0.11 | 0.78 | 1 |
| 11_11343 | 7H | 0 | 0.47 | 0.34 | 1 |
| 11_20076 | 7H | 0 | 0.05 | 0.90 | 1 |
| 11_20303 | 7H | 0 | 0.01 | 0.97 | 1 |
| 11_20311 | 7H | 0 | 0.02 | 0.96 | 1 |
| 11_20584 | 7H | 0 | 0.01 | 0.99 | 1 |
| 11_20623 | 7H | 0 | 0.12 | 0.76 | 1 |
| 11_20691 | 7H | 0 | 0.88 | 0.13 | 1 |
| 11_20998 | 7H | 0 | 0.12 | 0.75 | 1 |
| 11_21419 | 7H | 0 | 0.13 | 0.74 | 1 |
| 11_21516 | 7H | 0 | 0.04 | 0.90 | 1 |
| 12_20016 | 7H | 0 | 0.12 | 0.76 | 1 |
| 12_30296 | 7H | 0 | 0.13 | 0.73 | 1 |
| 12_30959 | 7H | 0 | 0.14 | 0.72 | 1 |
| 11_10682 | 7H | 0.62 | 0.05 | 0.90 | 1 |
| 11_11132 | 7H | 0.62 | 0.19 | 0.64 | 1 |
| 11_21443 | 7H | 0.62 | 0.07 | 0.84 | 1 |
| 12_30472 | 7H | 0.62 | 0.08 | 0.83 | 1 |
| 11_10121 | 7H | 1.89 | 0.34 | 0.46 | 1 |
| 11_10894 | 7H | 1.89 | 0.09 | 0.81 | 1 |
| 11_21307 | 7H | 1.89 | 0.02 | 0.95 | 1 |
| 12_20201 | 7H | 1.89 | 0.23 | 0.58 | 1 |
| 11_20710 | 7H | 3.34 | 0.04 | 0.91 | 1 |
| 12_31350 | 7H | 3.34 | 0.58 | 0.27 | 1 |
| 11_11179 | 7H | 4.12 | 0.00 | 1.00 | 1 |
| 11_20242 | 7H | 4.89 | 0.08 | 0.83 | 1 |
| 12_30836 | 7H | 4.89 | 0.34 | 0.46 | 1 |
| 12_31173 | 7H | 4.89 | 0.05 | 0.88 | 1 |
| 11_11495 | 7H | 6.14 | 0.56 | 0.28 | 1 |
| 12_10406 | 7H | 6.78 | 0.23 | 0.58 | 1 |
| 12_11433 | 7H | 6.78 | 0.30 | 0.50 | 1 |
| 11_20534 | 7H | 6.79 | 0.25 | 0.56 | 1 |
| 11_20307 | 7H | 9.84 | 0.01 | 0.99 | 1 |
| 12_11035 | 7H | 9.84 | 0.32 | 0.48 | 1 |
| 11_20245 | 7H | 12.42 | 0.47 | 0.34 | 1 |
| 12_31450 | 7H | 12.42 | 0.21 | 0.62 | 1 |
| 12_20227 | 7H | 12.51 | 0.00 | 0.99 | 1 |
| 11_10841 | 7H | 14.96 | 0.40 | 0.40 | 1 |
| 11_10851 | 7H | 14.96 | 0.01 | 0.97 | 1 |
| 12_30851 | 7H | 14.96 | 0.26 | 0.55 | 1 |
| 12_31285 | 7H | 14.96 | 0.01 | 0.97 | 1 |
| 11_20755 | 7H | 15.93 | 0.11 | 0.78 | 1 |
| 11_20014 | 7H | 17.2 | 0.38 | 0.42 | 1 |
| 11_21437 | 7H | 17.2 | 0.38 | 0.42 | 1 |
| 12_30902 | 7H | 17.56 | 0.00 | 1.00 | 1 |
| 12_30723 | 7H | 19.11 | 0.01 | 0.99 | 1 |
| 11_20722 | 7H | 19.25 | 0.47 | 0.34 | 1 |
| 11_21050 | 7H | 19.85 | 0.03 | 0.93 | 1 |
| 11_10025 | 7H | 21.13 | 0.31 | 0.49 | 1 |
| 11_20495 | 7H | 25.7 | 0.91 | 0.12 | 1 |
| 12_30530 | 7H | 25.93 | 1.35 | 0.04 | 1 |
| 12_30329 | 7H | 28.27 | 0.65 | 0.22 | 1 |
| 11_10965 | 7H | 29.82 | 0.39 | 0.41 | 1 |
| 12_30780 | 7H | 29.82 | 0.48 | 0.33 | 1 |
| 11_10920 | 7H | 31.75 | 0.25 | 0.56 | 1 |
| 11_20162 | 7H | 31.75 | 0.54 | 0.29 | 1 |
| 11_20507 | 7H | 31.75 | 0.39 | 0.40 | 1 |
| 12_30040 | 7H | 31.75 | 0.50 | 0.32 | 1 |
| 12_30063 | 7H | 31.75 | 0.50 | 0.32 | 1 |
| 11_10451 | 7H | 32.63 | 0.13 | 0.74 | 1 |
| 11_20758 | 7H | 32.63 | 0.12 | 0.76 | 1 |
| 12_30702 | 7H | 32.63 | 0.09 | 0.81 | 1 |
| 11_10232 | 7H | 34.82 | 0.32 | 0.48 | 1 |
| 11_20192 | 7H | 34.82 | 0.49 | 0.32 | 1 |
| 11_20993 | 7H | 34.82 | 0.11 | 0.77 | 1 |
| 12_30083 | 7H | 34.82 | 0.00 | 0.99 | 1 |
| 12_30141 | 7H | 34.82 | 0.12 | 0.76 | 1 |
| 12_30219 | 7H | 34.82 | 0.59 | 0.26 | 1 |
| 12_30242 | 7H | 36.77 | 0.09 | 0.81 | 1 |
| 11_20126 | 7H | 37.55 | 0.25 | 0.57 | 1 |
| 12_30893 | 7H | 37.55 | 0.08 | 0.84 | 1 |
| 12_30894 | 7H | 37.55 | 0.08 | 0.84 | 1 |
| 12_30895 | 7H | 37.55 | 0.07 | 0.85 | 1 |
| 11_10838 | 7H | 38.32 | 0.12 | 0.76 | 1 |
| 12_10218 | 7H | 39.04 | 0.53 | 0.29 | 1 |
| 11_10056 | 7H | 40.18 | 0.14 | 0.72 | 1 |
| 12_31305 | 7H | 40.96 | 0.29 | 0.52 | 1 |
| 11_10576 | 7H | 41.85 | 0.19 | 0.65 | 1 |
| 11_10327 | 7H | 42.6 | 0.14 | 0.73 | 1 |
| 12_30065 | 7H | 42.6 | 0.43 | 0.37 | 1 |
| 12_10979 | 7H | 43.38 | 0.19 | 0.64 | 1 |
| 12_10368 | 7H | 45.71 | 1.14 | 0.07 | 1 |
| 12_30143 | 7H | 45.71 | 0.49 | 0.33 | 1 |
| 11_21528 | 7H | 46.19 | 0.72 | 0.19 | 1 |
| 12_11339 | 7H | 48.13 | 0.00 | 1.00 | 1 |
| 11_21491 | 7H | 48.9 | 0.21 | 0.61 | 1 |
| 11_20357 | 7H | 49.68 | 2.13 | 0.01 | 1 |
| 11_21326 | 7H | 49.68 | 0.36 | 0.44 | 1 |
| 12_30528 | 7H | 49.68 | 0.24 | 0.57 | 1 |
| 11_20249 | 7H | 52.82 | 0.29 | 0.51 | 1 |
| 12_30545 | 7H | 53.6 | 0.49 | 0.33 | 1 |
| 12_30752 | 7H | 53.6 | 0.59 | 0.26 | 1 |
| 11_10772 | 7H | 54.37 | 0.26 | 0.56 | 1 |
| 11_20074 | 7H | 54.37 | 0.54 | 0.29 | 1 |
| 11_20790 | 7H | 55.63 | 0.05 | 0.89 | 1 |
| 12_10696 | 7H | 55.63 | 0.55 | 0.29 | 1 |
| 12_20031 | 7H | 55.63 | 0.00 | 1.00 | 1 |
| 11_10726 | 7H | 56.81 | 0.04 | 0.91 | 1 |
| 11_20113 | 7H | 56.81 | 0.94 | 0.12 | 1 |
| 12_30290 | 7H | 57.58 | 0.62 | 0.24 | 1 |
| 12_30181 | 7H | 58.36 | 0.25 | 0.57 | 1 |
| 12_10959 | 7H | 58.57 | 3.37 | 0.00 | 0.538343 |
| 12_30576 | 7H | 58.57 | 0.66 | 0.22 | 1 |
| 11_11014 | 7H | 60.69 | 1.18 | 0.07 | 1 |
| 11_10346 | 7H | 61.32 | 0.06 | 0.87 | 1 |
| 12_10403 | 7H | 61.32 | 0.03 | 0.92 | 1 |
| 12_30879 | 7H | 61.32 | 2.79 | 0.00 | 0.813054 |
| 12_30880 | 7H | 61.32 | 3.42 | 0.00 | 0.538343 |
| 11_10721 | 7H | 62.88 | 0.41 | 0.39 | 1 |
| 11_10050 | 7H | 63.66 | 0.04 | 0.91 | 1 |
| 11_20975 | 7H | 63.66 | 0.08 | 0.84 | 1 |
| 12_30149 | 7H | 63.66 | 0.64 | 0.23 | 1 |
| 12_10605 | 7H | 64.8 | 0.28 | 0.53 | 1 |
| 11_11098 | 7H | 68.46 | 0.47 | 0.34 | 1 |
| 11_20671 | 7H | 68.46 | 0.05 | 0.90 | 1 |
| 11_21270 | 7H | 68.46 | 0.59 | 0.25 | 1 |
| 12_10267 | 7H | 68.46 | 0.28 | 0.52 | 1 |
| 12_30639 | 7H | 68.46 | 0.14 | 0.72 | 1 |
| 12_31463 | 7H | 68.46 | 0.14 | 0.72 | 1 |
| 11_11348 | 7H | 70.4 | 0.06 | 0.87 | 1 |
| 12_31441 | 7H | 70.4 | 0.17 | 0.67 | 1 |
| 11_10431 | 7H | 71.1 | 0.03 | 0.93 | 1 |
| 11_11028 | 7H | 71.1 | 0.15 | 0.71 | 1 |
| 11_20060 | 7H | 71.1 | 0.17 | 0.67 | 1 |
| 11_20195 | 7H | 71.1 | 0.03 | 0.92 | 1 |
| 12_11242 | 7H | 71.1 | 0.00 | 1.00 | 1 |
| 12_30125 | 7H | 71.73 | 0.21 | 0.62 | 1 |
| 11_10153 | 7H | 73.75 | 0.61 | 0.24 | 1 |
| 11_10299 | 7H | 73.75 | 0.66 | 0.22 | 1 |
| 11_11122 | 7H | 73.75 | 0.11 | 0.77 | 1 |
| 12_11045 | 7H | 73.75 | 0.00 | 1.00 | 1 |
| 12_11103 | 7H | 73.75 | 0.14 | 0.72 | 1 |
| 12_11202 | 7H | 73.75 | 0.00 | 1.00 | 1 |
| 12_11257 | 7H | 73.75 | 0.25 | 0.56 | 1 |
| 12_21234 | 7H | 73.75 | 0.00 | 1.00 | 1 |
| 12_21492 | 7H | 73.75 | 0.00 | 1.00 | 1 |
| 12_30496 | 7H | 73.75 | 0.17 | 0.67 | 1 |
| 12_30832 | 7H | 73.75 | 0.14 | 0.72 | 1 |
| 12_30997 | 7H | 73.75 | 0.00 | 0.99 | 1 |
| 12_31452 | 7H | 73.75 | 0.31 | 0.49 | 1 |
| 11_10983 | 7H | 74.52 | 0.12 | 0.76 | 1 |
| 11_20885 | 7H | 74.52 | 0.41 | 0.39 | 1 |
| 12_21167 | 7H | 74.52 | 0.00 | 1.00 | 1 |
| 12_31120 | 7H | 74.52 | 0.39 | 0.41 | 1 |
| 12_30344 | 7H | 76.08 | 0.56 | 0.28 | 1 |
| 12_10655 | 7H | 76.17 | 0.15 | 0.71 | 1 |
| 12_30595 | 7H | 76.17 | 0.15 | 0.71 | 1 |
| 12_30581 | 7H | 77.45 | 0.00 | 1.00 | 1 |
| 11_10256 | 7H | 77.85 | 0.74 | 0.18 | 1 |
| 11_10394 | 7H | 77.85 | 0.56 | 0.28 | 1 |
| 11_10924 | 7H | 77.85 | 0.67 | 0.21 | 1 |
| 11_20828 | 7H | 77.85 | 0.55 | 0.28 | 1 |
| 11_20879 | 7H | 77.85 | 0.20 | 0.64 | 1 |
| 11_20893 | 7H | 77.85 | 0.55 | 0.28 | 1 |
| 11_20911 | 7H | 77.85 | 0.56 | 0.28 | 1 |
| 12_10222 | 7H | 77.85 | 0.56 | 0.27 | 1 |
| 12_10268 | 7H | 77.85 | 0.00 | 1.00 | 1 |
| 12_10459 | 7H | 77.85 | 0.13 | 0.74 | 1 |
| 12_10698 | 7H | 77.85 | 0.63 | 0.23 | 1 |
| 12_30053 | 7H | 77.85 | 0.56 | 0.28 | 1 |
| 12_30411 | 7H | 77.85 | 0.56 | 0.28 | 1 |
| 12_30486 | 7H | 77.85 | 0.56 | 0.28 | 1 |
| 12_30492 | 7H | 77.85 | 0.13 | 0.74 | 1 |
| 12_30544 | 7H | 77.85 | 0.56 | 0.28 | 1 |
| 12_30760 | 7H | 77.85 | 0.67 | 0.21 | 1 |
| 12_30794 | 7H | 77.85 | 0.67 | 0.21 | 1 |
| 12_31227 | 7H | 77.85 | 0.56 | 0.28 | 1 |
| 11_10073 | 7H | 78.22 | 0.09 | 0.82 | 1 |
| 12_11477 | 7H | 78.22 | 0.36 | 0.43 | 1 |
| 12_30004 | 7H | 78.22 | 0.13 | 0.74 | 1 |
| 12_30481 | 7H | 78.22 | 0.51 | 0.31 | 1 |
| 12_31000 | 7H | 78.22 | 0.36 | 0.43 | 1 |
| 11_10055 | 7H | 79.6 | 0.14 | 0.72 | 1 |
| 11_10370 | 7H | 79.6 | 0.55 | 0.28 | 1 |
| 11_10773 | 7H | 79.6 | 0.10 | 0.80 | 1 |
| 11_11145 | 7H | 79.6 | 0.09 | 0.81 | 1 |
| 11_11219 | 7H | 79.6 | 0.02 | 0.95 | 1 |
| 11_11352 | 7H | 79.6 | 0.09 | 0.82 | 1 |
| 11_20200 | 7H | 79.6 | 0.56 | 0.28 | 1 |
| 11_20460 | 7H | 79.6 | 0.19 | 0.65 | 1 |
| 11_21302 | 7H | 79.6 | 0.15 | 0.71 | 1 |
| 11_21335 | 7H | 79.6 | 0.08 | 0.84 | 1 |
| 11_21494 | 7H | 79.6 | 0.53 | 0.29 | 1 |
| 12_10581 | 7H | 79.6 | 0.14 | 0.73 | 1 |
| 12_10713 | 7H | 79.6 | 0.13 | 0.74 | 1 |
| 12_11146 | 7H | 79.6 | 0.18 | 0.67 | 1 |
| 12_11536 | 7H | 79.6 | 0.36 | 0.43 | 1 |
| 12_21319 | 7H | 79.6 | 0.00 | 1.00 | 1 |
| 12_30389 | 7H | 79.6 | 0.56 | 0.28 | 1 |
| 12_30445 | 7H | 79.6 | 0.56 | 0.28 | 1 |
| 12_30449 | 7H | 79.6 | 0.09 | 0.82 | 1 |
| 12_30475 | 7H | 79.6 | 0.17 | 0.67 | 1 |
| 12_30550 | 7H | 79.6 | 0.56 | 0.28 | 1 |
| 12_30563 | 7H | 79.6 | 0.13 | 0.73 | 1 |
| 12_30574 | 7H | 79.6 | 0.01 | 0.98 | 1 |
| 12_30589 | 7H | 79.6 | 0.14 | 0.73 | 1 |
| 12_30600 | 7H | 79.6 | 0.64 | 0.23 | 1 |
| 12_30621 | 7H | 79.6 | 0.56 | 0.28 | 1 |
| 12_30835 | 7H | 79.6 | 0.12 | 0.76 | 1 |
| 12_31140 | 7H | 79.6 | 0.09 | 0.80 | 1 |
| 12_31215 | 7H | 79.6 | 0.56 | 0.28 | 1 |
| 12_31345 | 7H | 79.6 | 0.09 | 0.82 | 1 |
| 12_31418 | 7H | 79.6 | 0.13 | 0.74 | 1 |
| 11_10534 | 7H | 80.94 | 0.15 | 0.71 | 1 |
| 12_30565 | 7H | 80.94 | 0.00 | 1.00 | 1 |
| 11_11461 | 7H | 82.34 | 0.01 | 0.99 | 1 |
| 11_10069 | 7H | 83.44 | 0.39 | 0.41 | 1 |
| 11_10673 | 7H | 83.44 | 0.40 | 0.40 | 1 |
| 11_20205 | 7H | 83.44 | 0.10 | 0.79 | 1 |
| 11_20349 | 7H | 83.44 | 0.52 | 0.30 | 1 |
| 11_21079 | 7H | 83.44 | 0.22 | 0.60 | 1 |
| 12_10125 | 7H | 83.44 | 0.07 | 0.86 | 1 |
| 12_10369 | 7H | 83.44 | 0.16 | 0.69 | 1 |
| 12_10982 | 7H | 83.44 | 0.07 | 0.86 | 1 |
| 12_11091 | 7H | 83.44 | 0.04 | 0.91 | 1 |
| 12_30213 | 7H | 83.44 | 0.36 | 0.44 | 1 |
| 12_30506 | 7H | 83.44 | 0.12 | 0.77 | 1 |
| 12_11377 | 7H | 84.07 | 0.05 | 0.90 | 1 |
| 12_30645 | 7H | 84.07 | 0.45 | 0.35 | 1 |
| 11_10442 | 7H | 84.92 | 0.38 | 0.41 | 1 |
| 11_10531 | 7H | 84.92 | 0.10 | 0.80 | 1 |
| 11_11239 | 7H | 84.92 | 0.10 | 0.79 | 1 |
| 11_11445 | 7H | 84.92 | 0.01 | 0.98 | 1 |
| 11_20880 | 7H | 84.92 | 0.08 | 0.83 | 1 |
| 12_11055 | 7H | 84.92 | 0.06 | 0.87 | 1 |
| 12_11499 | 7H | 84.92 | 0.24 | 0.57 | 1 |
| 12_11529 | 7H | 84.92 | 0.04 | 0.91 | 1 |
| 12_30998 | 7H | 84.92 | 0.08 | 0.83 | 1 |
| 11_20042 | 7H | 86.44 | 0.06 | 0.87 | 1 |
| 11_20230 | 7H | 86.44 | 0.52 | 0.30 | 1 |
| 11_20896 | 7H | 86.44 | 0.13 | 0.74 | 1 |
| 11_21330 | 7H | 86.44 | 0.12 | 0.75 | 1 |
| 12_30199 | 7H | 86.44 | 0.25 | 0.56 | 1 |
| 12_31137 | 7H | 86.44 | 0.14 | 0.73 | 1 |
| 12_31199 | 7H | 86.44 | 0.18 | 0.66 | 1 |
| 11_21409 | 7H | 87.21 | 0.27 | 0.53 | 1 |
| 11_10143 | 7H | 87.97 | 0.47 | 0.34 | 1 |
| 11_10303 | 7H | 87.97 | 0.26 | 0.55 | 1 |
| 11_20083 | 7H | 87.97 | 0.04 | 0.92 | 1 |
| 12_10089 | 7H | 87.97 | 0.07 | 0.85 | 1 |
| 12_30419 | 7H | 88.65 | 0.01 | 0.97 | 1 |
| 12_11437 | 7H | 91.79 | 0.15 | 0.71 | 1 |
| 12_20685 | 7H | 91.79 | 0.18 | 0.66 | 1 |
| 12_30026 | 7H | 91.79 | 0.12 | 0.77 | 1 |
| 12_30301 | 7H | 91.79 | 0.22 | 0.60 | 1 |
| 12_30996 | 7H | 91.79 | 0.07 | 0.85 | 1 |
| 11_21201 | 7H | 98.5 | 0.02 | 0.96 | 1 |
| 11_21448 | 7H | 98.5 | 0.09 | 0.81 | 1 |
| 12_30806 | 7H | 99.67 | 0.14 | 0.72 | 1 |
| 12_31395 | 7H | 99.67 | 0.07 | 0.86 | 1 |
| 12_31188 | 7H | 100.54 | 0.00 | 1.00 | 1 |
| 11_20808 | 7H | 101.32 | 0.11 | 0.77 | 1 |
| 12_30335 | 7H | 101.32 | 0.01 | 0.97 | 1 |
| 11_20103 | 7H | 102.85 | 0.75 | 0.18 | 1 |
| 12_20611 | 7H | 102.85 | 0.00 | 1.00 | 1 |
| 12_30168 | 7H | 102.85 | 0.00 | 1.00 | 1 |
| 12_11051 | 7H | 103.62 | 0.93 | 0.12 | 1 |
| 12_30630 | 7H | 103.62 | 0.63 | 0.23 | 1 |
| 12_31294 | 7H | 103.62 | 0.61 | 0.24 | 1 |
| 12_31440 | 7H | 103.62 | 0.61 | 0.25 | 1 |
| 11_10169 | 7H | 104.78 | 0.01 | 0.97 | 1 |
| 12_31261 | 7H | 107.11 | 0.26 | 0.54 | 1 |
| 11_20824 | 7H | 107.9 | 0.12 | 0.77 | 1 |
| 12_10362 | 7H | 108.67 | 0.37 | 0.43 | 1 |
| 12_20684 | 7H | 109.73 | 0.17 | 0.68 | 1 |
| 11_10853 | 7H | 110.99 | 0.14 | 0.72 | 1 |
| 11_20092 | 7H | 110.99 | 0.01 | 0.98 | 1 |
| 11_20385 | 7H | 110.99 | 0.13 | 0.74 | 1 |
| 11_20652 | 7H | 110.99 | 0.01 | 0.97 | 1 |
| 11_10563 | 7H | 112.46 | 0.10 | 0.79 | 1 |
| 11_20570 | 7H | 112.46 | 0.05 | 0.88 | 1 |
| 12_10241 | 7H | 112.46 | 0.27 | 0.53 | 1 |
| 12_10652 | 7H | 112.46 | 0.11 | 0.77 | 1 |
| 12_30362 | 7H | 114.78 | 0.04 | 0.90 | 1 |
| 11_20247 | 7H | 116.33 | 0.45 | 0.36 | 1 |
| 12_21208 | 7H | 116.33 | 0.06 | 0.87 | 1 |
| 12_30797 | 7H | 116.95 | 0.42 | 0.38 | 1 |
| 12_30368 | 7H | 118.9 | 0.35 | 0.45 | 1 |
| 12_11184 | 7H | 119.54 | 0.00 | 1.00 | 1 |
| 12_30164 | 7H | 119.54 | 0.16 | 0.69 | 1 |
| 12_20217 | 7H | 121.09 | 0.04 | 0.92 | 1 |
| 11_11243 | 7H | 122.07 | 0.21 | 0.61 | 1 |
| 12_10543 | 7H | 122.07 | 1.82 | 0.02 | 1 |
| 11_20354 | 7H | 125.17 | 1.15 | 0.07 | 1 |
| 12_31535 | 7H | 125.17 | 0.11 | 0.77 | 1 |
| 12_31374 | 7H | 126.68 | 0.51 | 0.31 | 1 |
| 11_11521 | 7H | 127.45 | 0.00 | 1.00 | 1 |
| 11_10182 | 7H | 128.36 | 0.99 | 0.10 | 1 |
| 11_21229 | 7H | 128.36 | 1.09 | 0.08 | 1 |
| 12_11279 | 7H | 128.36 | 0.82 | 0.15 | 1 |
| 12_20832 | 7H | 129.14 | 0.01 | 0.97 | 1 |
| 11_21209 | 7H | 129.91 | 0.74 | 0.18 | 1 |
| 11_10861 | 7H | 133.79 | 1.01 | 0.10 | 1 |
| 11_21104 | 7H | 133.79 | 0.62 | 0.24 | 1 |
| 11_10078 | 7H | 136.62 | 0.67 | 0.21 | 1 |
| 11_10797 | 7H | 136.62 | 0.00 | 0.99 | 1 |
| 11_21160 | 7H | 136.62 | 0.03 | 0.93 | 1 |
| 12_30380 | 7H | 138.17 | 0.16 | 0.70 | 1 |
| 11_10885 | 7H | 139.72 | 0.38 | 0.42 | 1 |
| 11_10454 | 7H | 140.21 | 0.15 | 0.71 | 1 |
| 11_20847 | 7H | 140.21 | 0.99 | 0.10 | 1 |
| 12_10973 | 7H | 140.21 | 1.01 | 0.10 | 1 |
| 11_10687 | 7H | 140.99 | 1.10 | 0.08 | 1 |
| 11_20139 | 7H | 141.76 | 0.78 | 0.16 | 1 |
| 11_21280 | 7H | 141.76 | 0.93 | 0.12 | 1 |
| 12_30761 | 7H | 141.76 | 0.78 | 0.16 | 1 |
| 12_20241 | 7H | 143.68 | 0.00 | 1.00 | 1 |
| 12_20949 | 7H | 143.68 | 0.00 | 1.00 | 1 |
| 12_31325 | 7H | 143.68 | 1.82 | 0.02 | 1 |
| 11_11440 | 7H | 144.45 | 0.79 | 0.16 | 1 |
| 11_20414 | 7H | 144.45 | 0.17 | 0.68 | 1 |
| 11_20452 | 7H | 144.45 | 0.76 | 0.17 | 1 |
| 11_21363 | 7H | 144.45 | 0.99 | 0.10 | 1 |
| 12_30593 | 7H | 144.45 | 0.66 | 0.22 | 1 |
| 12_31166 | 7H | 144.45 | 1.68 | 0.02 | 1 |
| 12_20640 | 7H | 145.23 | 0.29 | 0.51 | 1 |
| 11_11012 | 7H | 147.47 | 0.36 | 0.44 | 1 |
| 11_10130 | 7H | 147.48 | 0.11 | 0.78 | 1 |
| 12_21016 | 7H | 147.48 | 0.01 | 0.98 | 1 |
| 11_10896 | 7H | 148.25 | 0.31 | 0.49 | 1 |
| 12_11309 | 7H | 148.25 | 0.00 | 1.00 | 1 |
| 11_11275 | 7H | 149.03 | 0.05 | 0.89 | 1 |
| 12_21328 | 7H | 149.03 | 0.01 | 0.98 | 1 |
| 11_20962 | 7H | 149.8 | 0.18 | 0.66 | 1 |
| 12_20926 | 7H | 149.8 | 0.00 | 1.00 | 1 |
| 12_30244 | 7H | 151.35 | 0.04 | 0.91 | 1 |
| 11_20117 | 7H | 157 | 0.59 | 0.26 | 1 |
| 12_20079 | 7H | 157 | 0.59 | 0.26 | 1 |
| 11_20504 | 7H | 157.76 | 0.05 | 0.90 | 1 |
| 11_21086 | 7H | 159.27 | 0.01 | 0.97 | 1 |
| 11_10999 | 7H | 161.43 | 0.23 | 0.59 | 1 |
| 12_30974 | 7H | 161.43 | 0.23 | 0.59 | 1 |
| 11_20170 | 7H | 161.54 | 0.22 | 0.61 | 1 |
| 11_10174 | 7H | 166.56 | 0.00 | 1.00 | 1 |
| 11_20365 | 7H | 166.56 | 0.01 | 0.98 | 1 |
| 12_30826 | 7H | 166.56 | 0.20 | 0.63 | 1 |
